# Synthesis and evaluation of novel 3,5-dinitrobenzoyl substituted amino acid hydrazides against *Mycobacterium tuberculosis*

**DOI:** 10.1039/d6md00338a

**Published:** 2026-09-10

**Authors:** Fatimah M. Alsalem, Alistair K. Brown, Andrew K. Crowhurst, Charlotte A. Taylor, Luka G. Larkin, Jason H. Gill, Jonathan D. Sellars

**Affiliations:** a Biosciences Institute, Faculty of Medical Sciences, Newcastle University Catherine Cookson Building, Framlington Place Newcastle upon Tyne NE2 4HH UK jon.sellars@newcastle.ac.uk +44 (191)2082357; b Chemistry, School of Natural & Environmental Sciences, Newcastle University Newcastle upon Tyne NE1 7RU UK; c School of Pharmacy, Faculty of Medical Sciences, Newcastle University King George VI Building Newcastle upon Tyne NE1 7RU UK; d Translational and Clinical Research Institute, Faculty of Medical Sciences, Newcastle University Newcastle upon Tyne NE2 4HH UK

## Abstract

Multidrug-resistant tuberculosis (MDR-TB) represents an urgent unmet clinical need, with current regimens limited by prolonged duration, toxicity, and escalating resistance. We report the design, synthesis, and biological evaluation of 43 novel 3,5-dinitrobenzoyl amino acid hydrazide analogues against wild-type and multidrug-resistant strains of *Mycobacterium tuberculosis*. Systematic variation of both the amino acid side chain and the aryl hydrazide substituent revealed that hydrophobic, extended side chains confer superior antimycobacterial potency, whilst the influence of the hydrazide substituent appears context-dependent, modulated by the steric demands of the amino acid side chain. Cytotoxicity against mammalian cells was observed for several potent analogues, representing an early-stage optimisation challenge with clearly identified structure-dependent separability. Molecular docking against decaprenylphosphoryl-β-d-ribose 2′-epimerase (DprE1) is presented as a hypothesis-generating framework to rationalise key SAR trends. These findings establish a refined SAR landscape that will guide future structural optimisation of this promising antitubercular scaffold.

## Abstract 2

Systematic SAR study of 3,5-dinitrobenzoyl amino acid hydrazides identifies analogues with MIC_95_ values as low as 2 μM against drug-susceptible and multidrug-resistant *Mycobacterium tuberculosis*.

## Graphical abstract

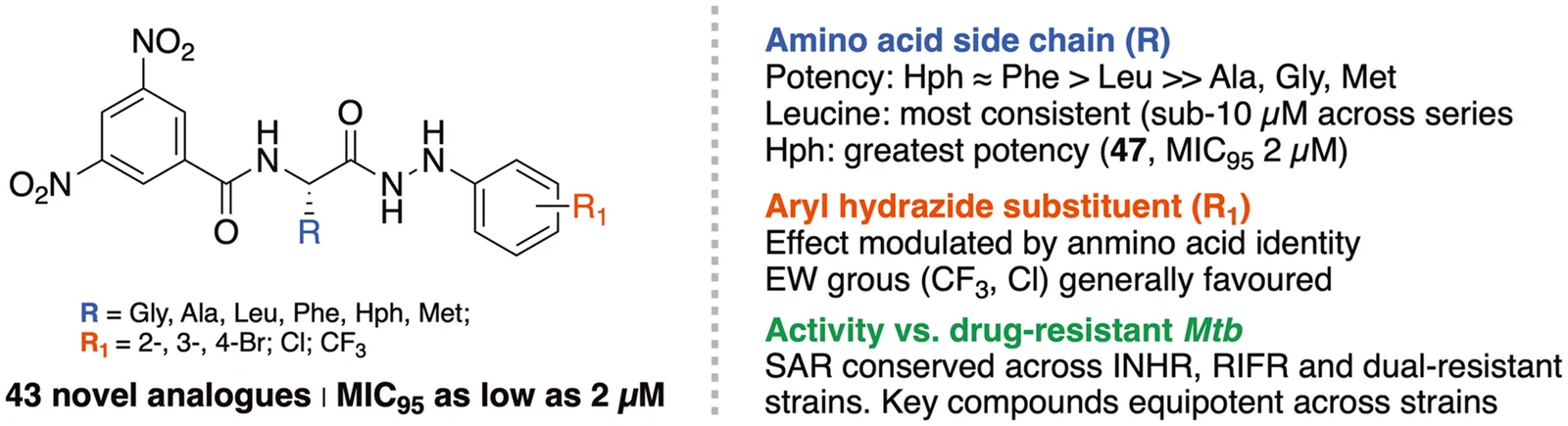

## 1. Introduction

Antibiotics have played a pivotal role in treating bacterial infections, revolutionising healthcare by enabling the successful management of diseases once considered untreatable.^1,2^ Nonetheless, the widespread and often inappropriate use of these agents, combined with a scarcity of new therapeutic options, has contributed to the emergence of bacteria resistant to multiple antibiotics, posing a significant global health threat.^3^ Among these, *Mycobacterium tuberculosis* (*Mtb*), the causative agent of Tuberculosis (TB), remains a major public health concern. The World Health Organisation (WHO) recently reported that although global TB cases declined in 2024 for the first time since the COVID-19 pandemic, most regions are still falling short of international TB control targets. The report also noted a rise in TB cases in the UK, with 6700 reported in 2024. This corresponds to an estimated incidence rate of 9.7 per 100 000, just under the WHO's low-incidence threshold of 10 per 100 000, suggesting that while progress has been made, sustained and targeted efforts are still required to bring TB under control globally and within high-income countries like the UK.^4,5^

Current treatment protocols for tuberculosis (TB) infections necessitate that patients consistently follow a prescribed regimen of multiple drugs over an extended period, typically lasting a minimum of six months.^6^ This prolonged therapy duration not only incurs significant financial costs but also yields inconsistent success rates across populations.^4^ The complexity and length of the treatment schedule pose considerable obstacles to ensuring patient compliance and sustained adherence to the medication regimen.^7^ Such challenges are particularly concerning as they play a pivotal role in the escalating incidence of drug-resistant TB strains.^7–10^ The emergence and spread of resistance to standard anti-TB drugs severely undermines the efficacy of existing treatments, leading to poorer health outcomes and more complicated clinical management of the disease.^4^ Considering these pressing issues, there is an urgent and critical demand for the development of novel therapeutic approaches and innovative strategies aimed at effectively combating TB, with a particular focus on addressing the growing threat posed by its drug-resistant variants.^11^

Recently, several new therapeutic approaches have been developed or entered clinical trials, including bedaquiline, clofazimine, and combination therapies such as bedaquiline–linezolid–pretomanid.^12^ Nonetheless, instances of resistance to several of these new treatments have already been identified.^13–18^ Consequently, to enhance the effectiveness of tuberculosis treatments, particularly in cases of drug resistance, it is vital to exploit new drug targets, develop unique therapeutic agents and thus identify novel treatment approaches to circumvent resistance and advance treatment options.

Compounds containing nitro groups are one such drug class gaining significant attention.^19^ Nitroimidazoles are a recently added new class of antitubercular agents with efficacy against *Mtb*, exemplified by the clinically active agent pretomanid 1 (Fig. 1), which targets both wild-type and multidrug-resistant strains of *Mtb*.^20–22^ The primary mechanism of action of this prodrug (1) involves bioactivation within *Mtb* by deazaflavin-dependent nitroreductase (Ddn) to its nitroso form, leading to inhibition of the biosynthesis of mycolic acids and subsequent cell wall biosynthesis.^23^ Another novel class of nitro-containing agents with significant promise for improved treatment of *Mtb* are electron-deficient nitroaromatic compounds, such as BTZ043, 2 and dinitroaromatics 3 (Fig. 1), which also exhibit efficacy against multidrug-resistant strains of TB.^24–26^ These compounds specifically inhibit decaprenylphosphoryl-β-d-ribose 2′-epimerase (DprE1), a critical enzyme in the biosynthesis of arabinogalactan, an essential component of the mycobacterial cell wall.^27,28^ This leads to a unique mode of action that does not overlap with existing TB therapies, effectively addressing the need for new treatment options.^24,29–35^

**Fig. 1:**
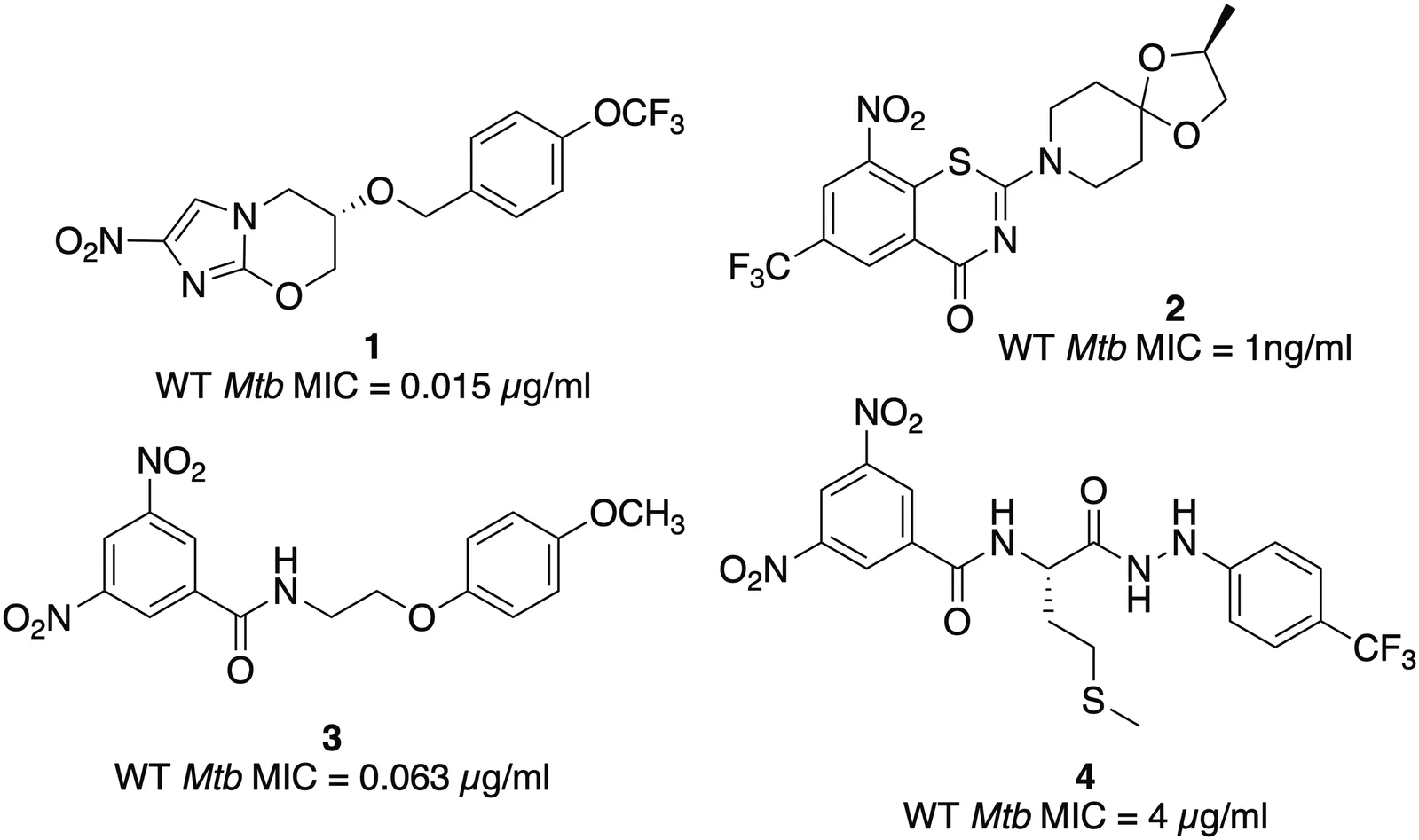
Selected nitro-containing compounds effective against *Mtb*; minimum inhibitory concentration against wild-type *Mtb* (WT *Mtb* MIC).

The relative success of these approaches has thus led to the exploration of structural modifications to enhance the activity of nitro-containing compounds, employing scaffold simplification and electronic modification strategies to optimise their therapeutic potential. For example, the incorporation of electron-withdrawing groups on nitroaromatic scaffolds has been shown to enhance antitubercular activity.^36,37^ In our previous studies focusing on a scaffold-hopping approach to identify key features of our amino acid hydrazide compounds, we demonstrated that attachment of 3,5-dinitrobenzoic acid to the amino acid hydrazide moiety yielded an inhibitor of mycobacterial growth in both susceptible and drug-resistant strains.^38^ This led to the identification of dinitroaryl amino acid hydrazide 4 as a potent inhibitor of mycobacterial growth in both susceptible and drug-resistant strains.^38^ Beyond the identification of dinitroaryl amino acid hydrazide 4, however, the structural determinants underpinning this activity have not been systematically explored, and it remains unclear whether antitubercular potency is governed principally by the amino acid side chain, the aryl hydrazide substituent, or an interplay between the two. Herein, we address this gap through the design, synthesis and biological evaluation of a 43-membered library that independently varies both structural elements, representing, to our knowledge, the most comprehensive SAR analysis of the 3,5-dinitrobenzoyl amino acid hydrazide scaffold 5 (Fig. 2). Screening against both wild-type and multidrug-resistant *Mtb* strains establishes whether these SAR trends translate across clinically relevant resistance backgrounds, while molecular docking against DprE1 is applied for the first time to this compound class to generate a testable hypothesis for its mechanism of action. Together, these data provide a refined structural framework to guide the rational optimisation of this promising antitubercular scaffold.

**Fig. 2:**
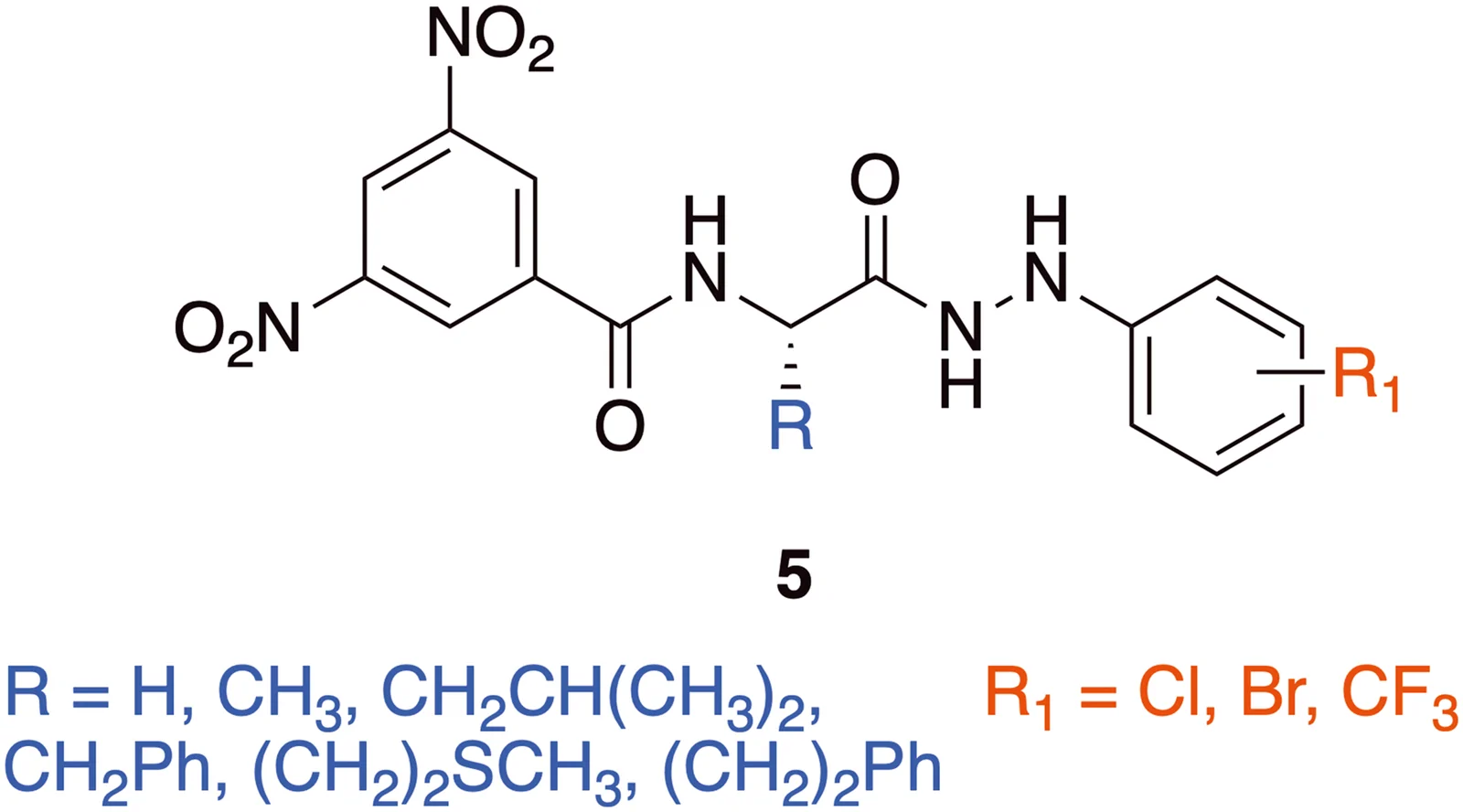
Regions of 3,5-dinitrobenzoyl amino acid hydrazides being explored for the structure–activity relationship between the amino acid and hydrazine within the compound architecture.

## 2. Results and discussion

### 2.1 Chemistry – synthesis of 3,5-dinitrobenzoyl amino acid hydrazides

Our previous studies outlined the concept for synthesising a wide array of substituted amino acid hydrazides.^38,39^ This involved a two-step approach, creating the protected amino acid hydrazide 8 from *N*-protected l-amino acids 6 and a substituted aryl hydrazine 7. Once amino acid hydrazide 8 was obtained, deprotection and coupling of the resulting free amine with 3,5-dinitrobenzoic acid 9 yielded the desired compound 10 (Fig. 3).

**Fig. 3:**
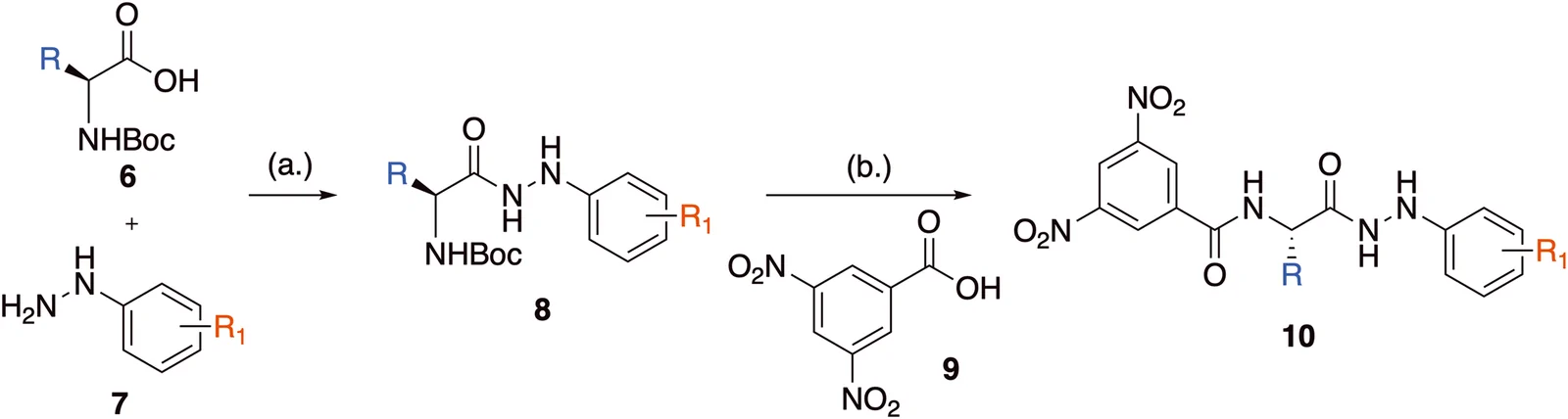
Reagents and conditions: (a.) HBTU, DIPEA, THF, 4 h, r.t.; (b.) 4N HCl in dioxane, 90 min, r.t. then 10, HBTU, DIPEA, THF, 4 h, r.t.

With a robust synthetic route established, a series of amino acid hydrazides 8 was prepared to investigate the contributions of both the hydrazine and the amino acid upon antimycobacterial activity. Guided by insight from prior SAR studies, the structural modifications described herein focused upon improving potency and selectivity through varying the hydrophobicity and steric bulk of amino acid side-chains, alongside electron-withdrawing and donating groups, such as halogens and alkyl groups, on the aryl hydrazine scaffold. Consequently, forty-three amino acid hydrazides were synthesised and subsequently converted to the desired 3,5-dinitrobenzoyl amino acid hydrazides for biological evaluation (Table 1).

**Table 1:** REMA assay results showing MIC_95_ values against wild-type and drug-resistant strains of *Mtb* for the 3,5-dinitrobenzoyl amino acid hydrazides grouped by amino acid; abbreviations: *Mtb* WT mc^2^7902 (wild-type *Mtb*), *Mtb* INH^R^ mc^2^8245 (isoniazid-resistant *Mtb*), *Mtb* RIF^R^ mc^2^8247 (rifampicin-resistant *Mtb*), *Mtb* INH^R^ RIF^R^ mc^2^8050 (*Mtb* resistant to both isoniazid and rifampicin)

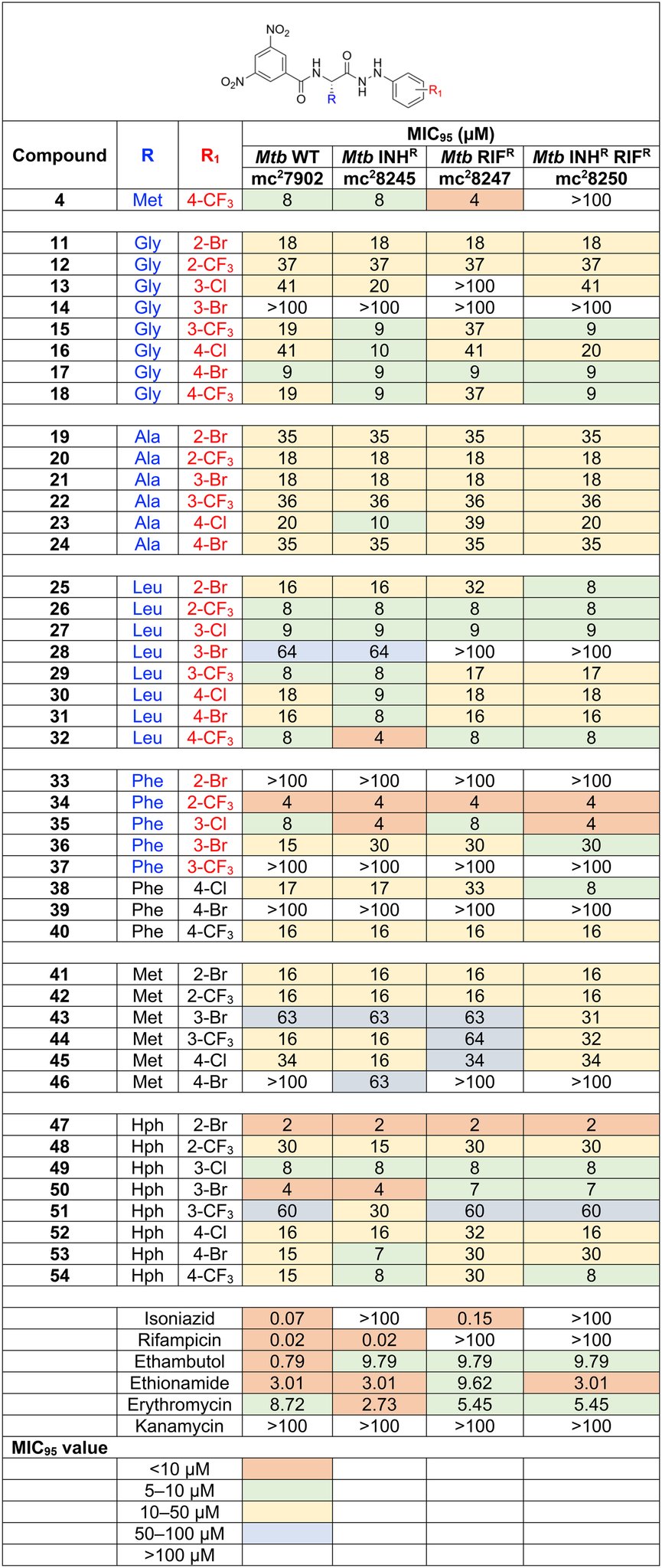

#### Structure–activity relationship analysis

Screening of compounds 11–54 against wild-type *Mtb* revealed a diverse range of growth inhibitory activities, with MIC_95_ (concentration of drug at which 95% of growth is inhibited compared to drug-vehicle control) from 2 to >100 μM (Table 1). This spread demonstrates that both the amino acid and aryl hydrazide regions of the scaffold exert substantial, independent control over antimycobacterial potency with modification at either position sufficient to shift activity across almost the full range observed. Critically, because the library was designed to vary each region systematically while holding the other constant, this represents, to our knowledge, the first study to deconvolute the contributions of these two structural variables at this scale, allowing robust, region-specific SAR conclusions to be drawn independently rather than inferred from a smaller or less systematically varied compound set.

Within the amino acid region, a clear trend emerged: MIC_95_ against *Mtb* improved progressively as side chain size and lipophilicity increased, from alanine to leucine to phenylalanine. Relative to alanine analogue 20 (bearing an *ortho*-trifluoromethyl hydrazide substituent), the corresponding leucine 26 and phenylalanine 34 analogues showed 2- and 4-fold increases in potency, respectively. Although these results support a relationship between side chain length and enhanced antibacterial activity, this trend is not maintained for compounds incorporating methionine. While methionine-containing analogues retained antimicrobial activity, they are comparable (or in some cases worse) than the corresponding alanine analogues. Considering the improved activities observed for phenylalanine, which has a side-chain length comparable to that of methionine, as well as the previously observed activity of compound 4, this lack of correlation is unexpected. It is therefore likely that the reduction in activity observed with these analogues is a consequence of the reactive sulphur within the side chain rather than from the side chain length alone.

To further probe the relationship between the amino acid side-chain length and antibacterial activity, phenylalanine analogues were compared with their homophenylalanine counterparts. A greater effect was observed between phenylalanine and homophenylalanine homologues, with a 3–4-fold improvement between 36 and 50, a >10-fold improvement between 39 and 53, and a markedly greater (>50-fold) increase in activity observed between 33*versus*47 (Table 1). However, this trend is not uniformly maintained across the compound series. For example, an inverse relationship is noted between the phenylalanine analogue 34 and the corresponding homophenylalanine analogue 48, which exhibited a 3–7-fold reduction in growth inhibition. This discrepancy appears to be the exception and is therefore likely attributed to differences in the respective hydrazide substitution rather than side-chain length alone. Despite these promising trends, antibacterial activities with phenylalanine (and homophenylalanine) containing compounds were noted to be inconsistent across the panel of compounds evaluated compared to those containing leucine. This is likely a reflection of the bulkier aromatic group, which may influence ligand orientation within the drug target active site, particularly when in combination with the varying aryl hydrazide aspect of the molecule.

Whilst complementarity to the as-yet unexplored binding site cannot be ignored, the data suggest that both the position and type of substituent present on the hydrazide substituent play a significant role in the antibacterial activity of the compounds. Notably, antibacterial activity was observed irrespective of the position of the substituent. For example, leucine-containing analogues bearing *ortho*-26, *meta*-29, and *para*-trifluoromethyl 32 substituents all exhibited sub-10 μM antibacterial activities, suggesting that the electronic properties of the substituents exert less influence over inhibitory activity than might be expected. Similarly, homophenylalanine analogues with *ortho*-, 47, *meta*-, 50, and *para*-bromide, 53, substitutions demonstrated exquisite inhibitory activities of 2, 4 and 15 μM against wild-type *Mtb*, respectively. However, a different response was observed for the corresponding brominated phenylalanine analogues (33, 36, 39). Both the *ortho*-33 and *para*-39 analogues lacked measurable antibacterial activity (>100 μM), while the *meta*-bromide 36 retained moderate activity (15 μM) (Table 1). In the case of the leucine bromide analogues, the reverse was observed, with *ortho*-25 and *para*-31 analogues showing sub-20 μM activity, whereas the *meta*-28 substitution exhibited very poor (>50 μM) activity. This lack of consistent SAR, coupled with the variable nature of the amino acid, makes the prediction of a robust pharmacophore particularly challenging. Despite this variability, there were some very impressive improvements in antibacterial activities with the *meta*-chloro substituent with either leucine 27, phenylalanine 35 or homophenylalanine 49 amino acid, delivering sub-10 μM antibacterial activities. Similarly, the *ortho*-trifluoromethyl substituent resulted in excellent activities (<10 μM) when combined with leucine 26 or phenylalanine 34, but less so with homophenylalanine 48 (15–30 μM). The precise rationale for these positional asymmetries remains unclear; they likely reflect a complex interplay between amino acid side chain geometry and the spatial constraints of the hydrazide binding region, and are acknowledged as exceptions rather than general trends.

#### Activity and selectivity against drug-resistant *Mtb*

Having established key SAR trends against wild-type *Mtb*, MIC_95_ data were obtained across isoniazid-resistant (INH^R^), rifampicin-resistant (RIF^R^), and dual INH^R^/RIF^R^ strains to determine whether potency was retained across clinically relevant resistance backgrounds (Table 1).

For the majority of compounds, MIC_95_ values against the resistant strains mirrored those obtained against wild-type *Mtb*, consistent with a mechanism of action that is distinct from those targeted by isoniazid and rifampicin. Leucine-containing analogues were particularly robust in this regard: compounds 26, 27, and 29 each maintained sub-10 μM activity across all four *Mtb* strains evaluated, while 32 achieved an improved MIC_95_ of 4 μM against INH^R^*Mtb*. Similarly, the most potent homophenylalanine compounds retained their activity: 47 (2 μM) and 50 (4 μM) were equipotent across all strains, and phenylalanine analogues 34 and 35 maintained 4 μM and sub-8 μM potency, respectively, across all resistance phenotypes. Notably, several compounds showed modest improvements against one or more resistant strains relative to wild-type: for example, 35 (Phe, 3-Cl) improved from 8 μM against wild-type to 4 μM against both INH^R^ and dual-resistant strains, and 32 (Leu, 4-CF_3_) improved to 4 μM against INH^R^*Mtb*. In contrast, where wild-type activity was already poor (MIC_95_ >50 μM), activity was generally not recovered against resistant strains, as seen across the glycine, alanine, and methionine series. Taken together, these data show that potency against wild-type *Mtb* is a reliable predictor of potency against isoniazid- and rifampicin-resistant strains within this scaffold, with leucine and homophenylalanine analogues representing the most promising candidates for further development. To our knowledge, this is the first systematic evidence that SAR trends across a 3,5-dinitrobenzoyl amino acid hydrazide-type scaffold hold across clinically relevant MDR-TB resistance phenotypes; a finding with direct practical value, since it means the wild-type-guided optimisation already carried out on this series does not need to be independently repeated against resistant strains, and can instead be used to prioritise candidates for MDR-TB-focused development.

#### Cytotoxicity analysis

The safety profile of nitro-containing compounds as therapeutics, including their use as antitubercular agents, is a critical area of investigation due to the inherent risk of cytotoxicity associated with this chemical class, particularly due to oxidative stress.^40,41^ Despite these potential risks, numerous studies have indicated that nitro-containing derivatives may possess acceptable safety profiles, whilst delivering robust therapeutic activity against *Mtb*.^42^ For instance, Karabanovich *et al.* noted that 3,5-dinitrobenzylsulfanyl-substituted heterocycles exhibit a good therapeutic index, with excellent antitubercular activity coupled with reduced human cell toxicity, which challenges the notion that nitro groups should be avoided in therapeutics.^42^ Consequently, cytotoxicity of a representative panel of the 3,5-dinitrobenzoyl amino acid hydrazide derivatives was evaluated to address any influence of both the amino acid (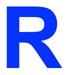) and aromatic substituents (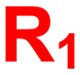) on cytotoxicity and subsequent therapeutic index.

As previously indicated, compounds bearing the leucine side chain consistently deliver very good antibacterial potency. However, a relationship between antibacterial potency and cytotoxicity appears to exist, indicating a poorer than desired therapeutic index (Table 2). Despite this, there are some promising observations within this series. For instance, moving the trifluoromethyl substituent from the *ortho*-26 to *meta*-29 position, whilst retaining excellent antibacterial activity (8 μM), reduces human cell cytotoxicity from >90% to almost 50%. Similarly, the size of the substituent also impacts this differential response, as evidenced by the *para*-chloro 30*versus para*-bromide 31 substituent, which have similar antibacterial activities but quite different cytotoxicity against human cells. This increase in substituent size from chloro to bromide increases the level of cytotoxicity from <30% and relatively safe to >50% and demonstrably cytotoxic. This shows how small positional changes in substituents can strongly influence a compound's therapeutic index, without dramatically altering desired therapeutic activity, offering significant scope for delivery of compounds with defined clinical potential.

**Table 2:** MTS assay results for compounds tested at 50 μM against the RAW 264.7 cell line

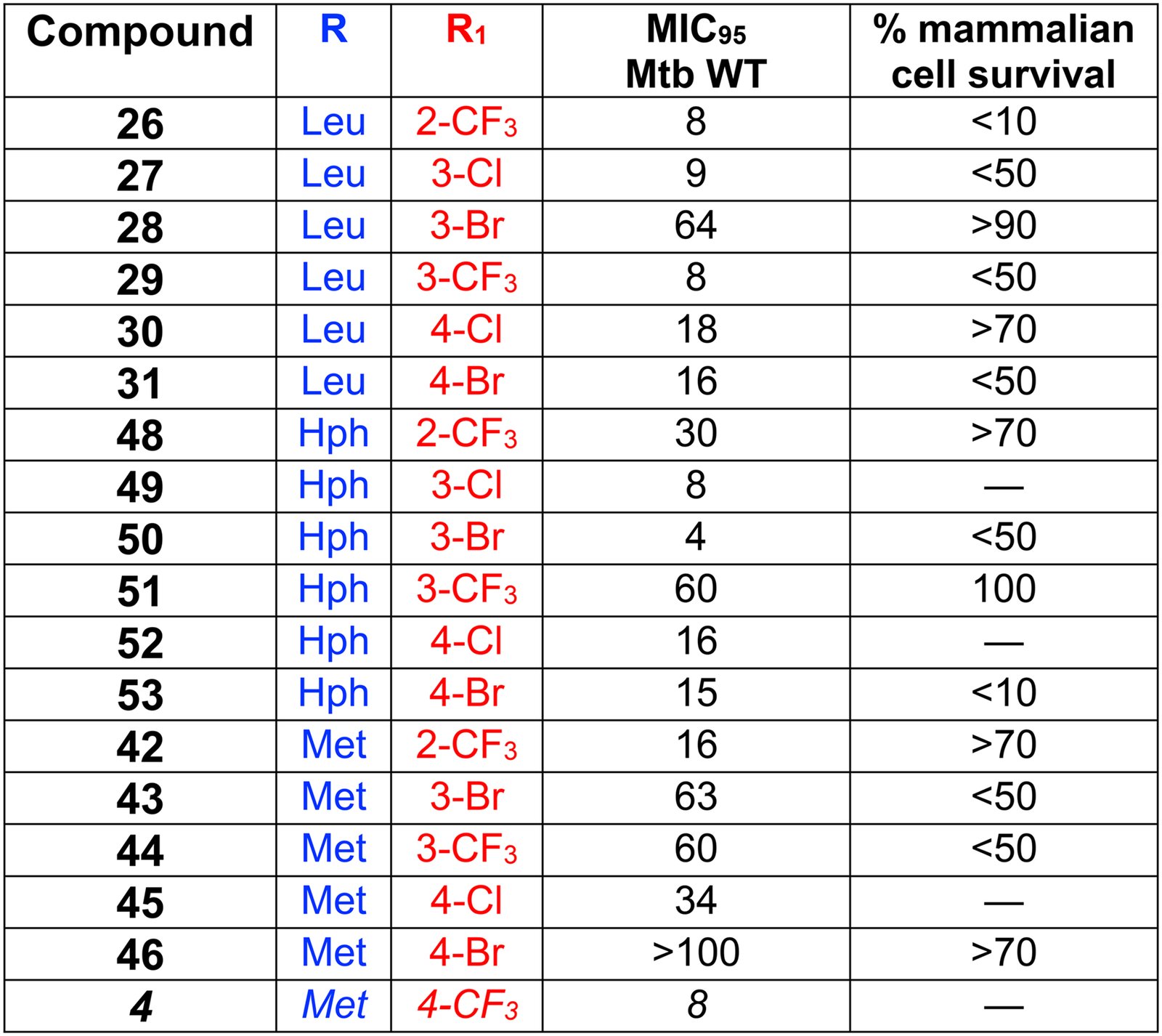

In addition to the aryl hydrazide substituent having a clear effect upon human cell cytotoxicity (and in parallel, potency), the amino acid can be seen to play a significant role. As above, the *ortho*-trifluoromethyl substituent resulted in excellent antibacterial activity when combined with leucine (MIC_95_ 8 μM) but less so with homophenylalanine (MIC_95_ 30 μM) and methionine (MIC_95_ 16 μM). When analysed in terms of cytotoxicity, this somewhat mirrored the antibacterial activity, resulting in >90%, <30% and <30% cytotoxicity, respectively.

Taken together, these data demonstrate that both the amino acid side chain and the substituent on the aryl hydrazide exert a strong influence on biological outcome, therapeutic index and longer-term potential for clinical translation. It is highly likely that the positioning of the compound within the drug target site in *Mtb* and within the respective site in human cells, as a consequence of the interaction of the amino acid at one side of the molecule and the accommodation of the hydrazide substituent at the other, is somewhat mirrored, although they are highly likely to be distinct protein families. Teasing out these interactions and the positioning of the molecules is therefore essential to identifying and developing these compounds as viable antibacterial agents. Gratifyingly, the diversity of this series of compounds underlines the interplay between amino acid identity and aromatic substitution in shaping antibacterial efficacy and human tolerability, providing a nuanced SAR landscape for further optimisation. Importantly, the observed cytotoxicity should be viewed as an early-stage optimisation challenge inherent to this class rather than a fundamental scaffold liability. The clear structure-dependent separability of antibacterial activity and cytotoxicity demonstrated within this series, for example, the contrasting profiles of analogues 27 and 29, provides a clear rationale for targeted structural modifications in future optimisation campaigns aimed at widening the therapeutic index.

#### Molecular modelling with the mycobacterial protein DprE1

*Mtb* DprE1 is a crucial enzyme in mycobacterial cell wall biosynthesis, playing a key role in the formation of arabinogalactan, an essential structural component of the cell wall. DprE1 is a flavoprotein that catalyses the epimerisation of decaprenylphosphoryl-β-d-ribose to decaprenylphosphoryl-β-d-arabinose, a precursor for the synthesis of both arabinogalactan and lipoarabinomannan, which are vital for maintaining mycobacterial cell wall integrity.^43,44^ The enzyme has emerged as a significant target for both benzothiazinones and dinitrobenzamides; however, the binding orientation of compounds within the enzymatic site remains to be fully determined.^24–26,45–47^ An in-depth analysis of the active site has revealed three key regions responsible for the binding of DprE1 inhibitors. The head region requires hydrophobic functional groups, whilst the trunk region, parallel to the isoalloxazine ring of FAD, accommodates generally flat heterocyclic fragments, and the tail region tolerates a wide variety of functional groups and is solvent accessible.^46^ Additionally, a recent structural study examining multiple crystal forms of DprE1 revealed considerable levels of structural flexibility within two surface loops that govern the accessibility of the active site. Interestingly, when DprE1 is complexed covalently with a BTZ-derived nitroso derivative, the flexible surface loops in the tail region remain disordered. In contrast, formation of a noncovalent complex with an analogous compound containing an inert nitro group induces ordering of the flexible loop region. This effectively closes the active site tail region and generates another hydrophobic pocket-like structure. These findings suggest that binding of BTZ-class inhibitors to DprE1 is not strictly dependent on the formation of the covalent link to Cys387, and that potential inhibitors can be designed to operate by either a covalent or noncovalent mechanism.^48^

Interestingly, a structural relationship between the substituted aryl hydrazides described herein and the previously defined nitro-containing benzothiazinones and dinitrobenzamides implicated DprE1 as a putative target for these compounds. No mechanistic hypothesis has previously been proposed to explain the biological activity of dinitrobenzoyl amino acid hydrazides, so this structural analogy provided a rational starting point for target identification. To investigate this hypothesis and gain insight into the potential mechanism of action, molecular docking studies were conducted with the *Mtb* DprE1 crystal structure (PDB: 4FDO), with compounds modelled to bind in a non-covalent manner. It is important to note that static, non-covalent docking against a single crystallographic structure carries inherent limitations: protein flexibility, solvent effects, and induced-fit contributions are not captured. Accordingly, the docking studies presented here should be interpreted as a hypothesis-generating framework that rationalises observed SAR trends, rather than as definitive mechanistic evidence of DprE1 engagement.

Using the SeeSAR molecular docking program (BioSolveIT GmbH, Sankt Augustin, Germany, SeeSAR version 14.1.2 2025), *in silico* docking studies with compounds 26, 34, 42 and 48 were conducted, representing the *ortho*-trifluoromethyl substituent on the hydrazide with leucine, phenylalanine, methionine and homophenylalanine, respectively. To assess the impact of the hydrazide substituent, compounds 26, 29 and 32 representing *ortho*-, *meta*- and *para*-trifluoromethyl substituents, respectively, in combination with a leucine side chain, were selected for docking studies. Inclusion of these variations enables the systematic evaluation of how both amino acid identity and substituent positioning influence binding interactions and activity.

Initially, the DprE1 crystal structure was prepared by removing the co-crystallised ligand from the active site, thereby defining the binding region for subsequent analysis. All compounds were then imported into SeeSAR and docked using the bound molecule as a template in the standard docking mode, generating up to 300 poses per compound. Optimising the binding and triaging the remaining poses produced a concise set of docked poses, with the lowest possible estimated binding affinities, alongside acceptable ligand lipophilic efficiencies (LLE) and statistic profiles.

Analysis of the non-covalent binding poses to DprE1 indicated that all the molecules 26, 29, 32, 34, 42, and 48 consistently orient the dinitro aromatic group toward the hydrophobic head region of the protein. Although this orientation might appear unfavourable, the amino acid hydrazide is directed towards the now closed hydrophobic tail end of the active site. Ultimately, this alignment more closely resembles that of the cocrystal-bound inhibitor, providing a better rationale for the observed inhibitory activity. With the orientation of the docked compounds established, attention was directed toward the amino acids of 26, 34, 42 and 48. Analysis of the docking poses reveals that all compounds position their amino acid side chains towards the prominent hydrophobic pocket, formed by Leu363 and Val365, along with Leu317 and Phe320 from the structured loop region (indicated in red, Fig. 4). This arrangement highlights the importance of aromatic or hydrophobic side chains, as evidenced by the correlation between the amino acids present in 26, 34, 42 and 48 and their associated potency. For instance, phenylalanine 34, the most potent of the selected analogues, aligns closely with the docked BTZ inhibitor and exploits the greatest number of van der Waals interactions with the flexible loop region. In contrast, the extended length of homophenylalanine 48 hindered good residency within this pocket, with leucine 26 showing greater binding than methionine 42 due to lower potential for repulsive van der Waals interactions with the flexible loop region (Fig. 4).

**Fig. 4:**
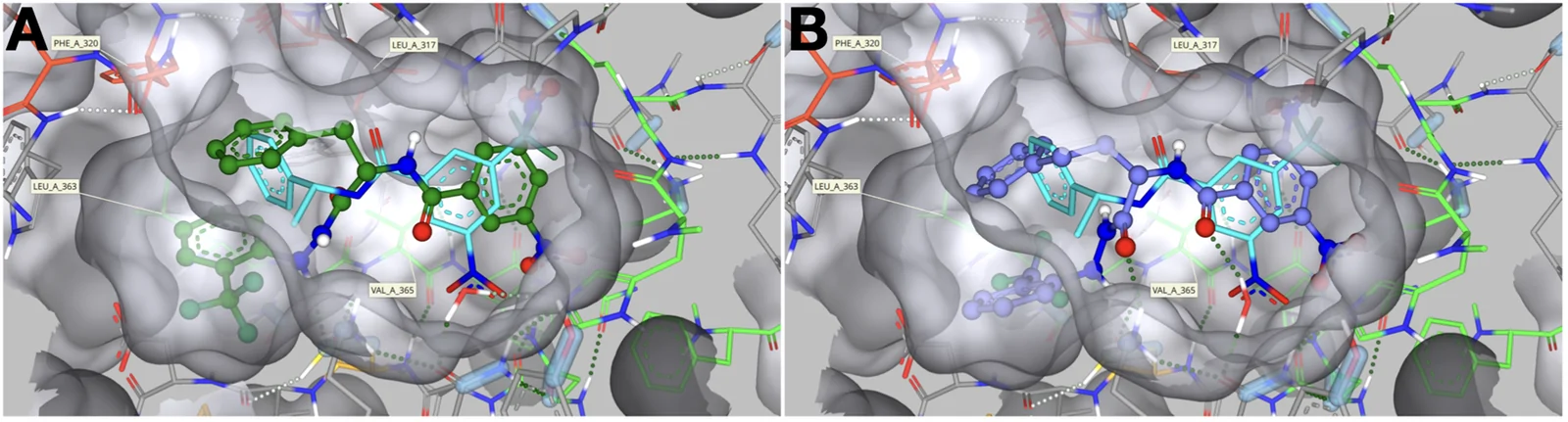
A. Docked inhibitor, 34 (green) B. Docked inhibitor, 48 (blue); both are present in DprE1 with the docked BTZ inhibitor (cyan), looking down through FAD (removed for clarity); hydrophobic head end of protein; red residues (top left) are now the ordered loop region closing the tail end of the active site.

The influence of substituent position on the hydrazide aromatic ring within the other analogues was further evaluated for analogues 26, 29, and 32. The position of the hydrazide within the closed hydrophobic tail of the active site is predicted to have little effect on the activity in this context. The experimental results support this prediction, with all three analogues exhibiting similar activities (MIC_95_ 8 μM) against wild-type *Mtb*.

The influence of substituent size was also evaluated through comparison of 35, 36 and 37, representing the phenylalanine analogue with *meta*-substituted chloro-, bromo-, and trifluoromethyl groups, respectively. It was anticipated that a more compact substituent arrangement would enhance inhibitory activity. Modelling of these analogues supports this hypothesis, with chloro and bromo analogues occupying the site more efficiently than the trifluoromethyl group, which reorientates itself as a consequence of steric clashes with the closed loop region (Fig. 5).

**Fig. 5:**
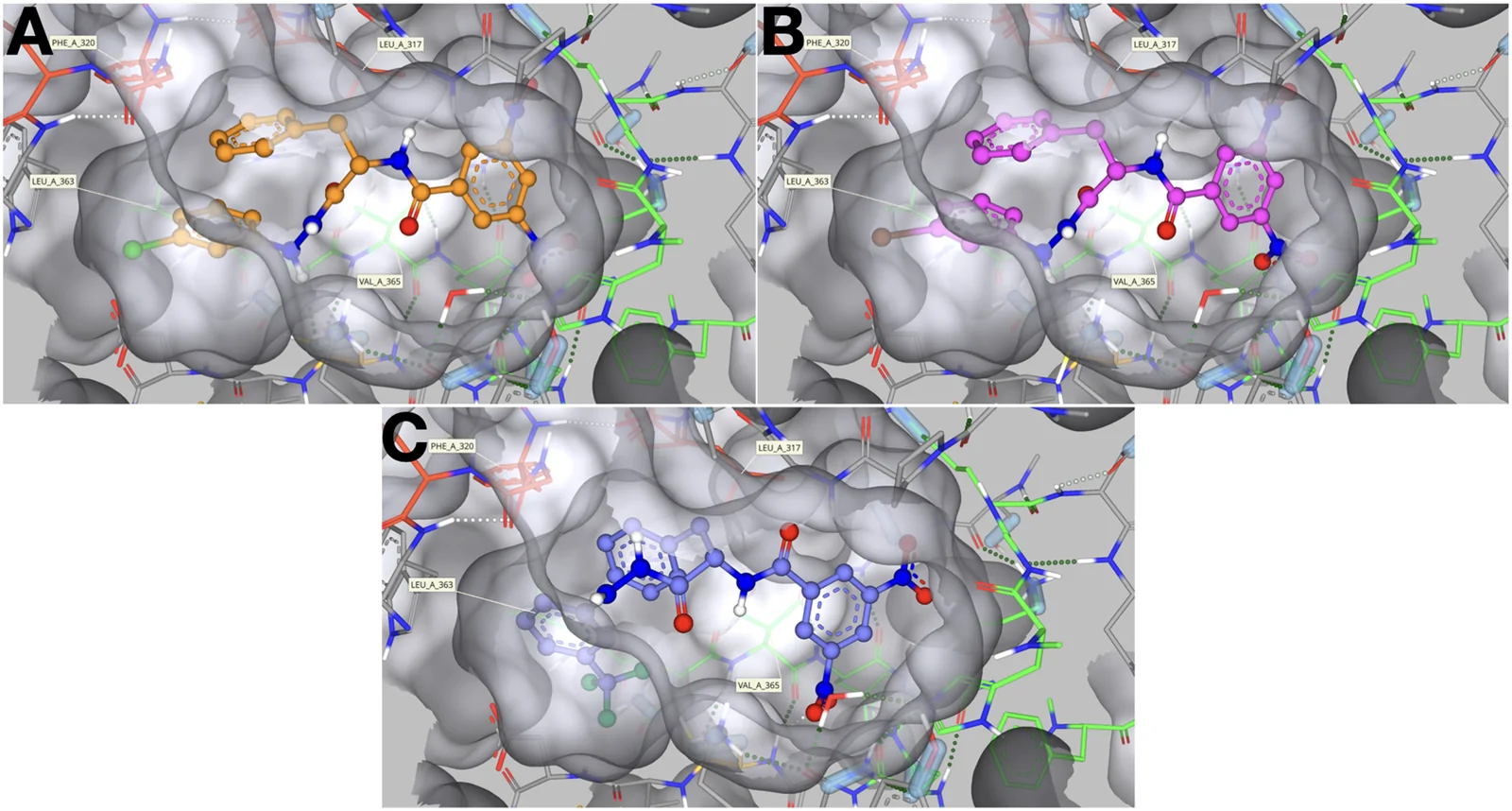
A. Docked inhibitor, 35 (orange), B. Docked inhibitor, 36 (pink), C. Docked inhibitor, 37 (blue); all three into DprE1, looking down through FAD (removed for clarity); hydrophobic head end of protein; red residues (top left) are now the ordered loop region closing the tail end of the active site.

Nevertheless, analysis of the full compound library reveals a variable and often inconsistent relationship between hydrazide substituent position and antibacterial activity. For example, in homophenylalanine analogues 47, 50, and 53, a clear positional trend is observed: inhibitory potency decreases as the halogen substituent shifts from *ortho* (2 μM) to *meta* (4 μM) to *para* (15 μM). In contrast, leucine analogues 26, 29, and 32 maintain consistent low MIC values (all ∼8 μM), regardless of the substituent's position. This uniform activity may reflect the leucine side chain's limited interaction with the flexible loop region, likely permitting more space for a stable interaction of the hydrazide. By comparison, analogues with bulkier side chains, such as phenylalanine and homophenylalanine, often display greater variability in activity. It is plausible that these larger, more rigid side chains possess greater occupancy of the flexible loop region, therefore inducing subtle conformational changes or steric hindrance within the binding pocket. As a result the position and nature of the aryl hydrazide substituent may play a more pronounced role, either compensating for or amplifying these effects. This provides a rationale for why identical hydrazide substitution patterns can produce divergent outcomes across different amino acid backbones. Overall, these findings suggest that hydrazide substituent effects are context-dependent, becoming more or less influential with respect to the side chain environment.

## 3. Conclusion

This study reports a comprehensive, two-variable SAR analysis of the 3,5-dinitrobenzoyl amino acid hydrazide scaffold, encompassing forty-three novel analogues with activity against wild-type and drug-resistant *Mtb* strains. The principal SAR findings are: (i) hydrophobic, extended side chains confer superior potency, with leucine analogues delivering the most consistent sub-10 μM activity and homophenylalanine analogues the greatest maximum potency (MIC_95_ 2 μM, 47); (ii) the influence of the aryl hydrazide substituent on activity is context-dependent, modulated by the steric bulk of the amino acid side chain; and (iii) these SAR trends are largely conserved across clinically relevant drug-resistant backgrounds, indicating that wild-type-guided optimisation translates directly to MDR-relevant potency.

Beyond potency, cytotoxicity against mammalian cells remains an early-stage optimisation challenge; however, we show that antibacterial potency and cytotoxicity are structurally separable within this scaffold (*e.g.* leucine analogues 27 and 29), giving a clear starting point for selectivity-focused optimisation. To begin addressing the mechanism, molecular docking against DprE1 is advanced as a hypothesis-generating framework rather than definitive evidence with future work incorporating molecular dynamics simulations and *in vitro* target engagement assays needed to test this hypothesis and guide rational design.

Taken together, by independently resolving the contributions of the amino acid and aryl hydrazide substituents across this large library, this work establishes a structural framework for the 3,5-dinitrobenzoyl amino acid hydrazide class that was previously unavailable; one that, combined with the DprE1 engagement hypothesis proposed here, provides a validated starting point for the rational design of next-generation dinitrobenzoyl antitubercular agents.

## Author contributions

Conceptualization: JDS; data curation: FMA, JHG, AKB, JDS; formal analysis: JHG, AKB, JDS; investigation: FMA, AKB, CAT, LGL, AKB; project administration: AKB, JDS; supervision: JDS; validation: JDS; writing – original draft: JDS; writing – review & editing: AKB, JHG, JDS.

## Conflicts of interest

There are no conflicts to declare.

## Supplementary Material

MD-017-D6MD00338A-s001

## Data Availability

The data supporting this article have been included as part of the supplementary information (SI). Supplementary information: S1. Reordered Table 1; S2. Methods and reported data for all compounds; S3 and S4. Biological data and experimental procedures for assays; S5. Methods and approach for molecular modelling; S6. Copies of ^1^H and ^13^C NMR spectra for compounds assayed in this study. See DOI: https://doi.org/10.1039/d6md00338a.
