# Supplementary material for: Synthesis and evaluation of novel 3,5-dinitrobenzoyl substituted amino acid hydrazides against Mycobacterium tuberculosis
Source: RSC Med Chem. 2026 Sep 10;17(10):4913–22. doi: 10.1039/d6md00338a (PMC13587841; doi:10.1039/d6md00338a)

Supplementary Information (SI) for RSC Medicinal Chemistry.
This journal is © The Royal Society of Chemistry 2026

Synthesis and Evaluation of Novel 3,5-dinitrobenzoyl Substituted Amino Acid Hydrazides Against *Mycobacterium tuberculosis*

Fatimah M. Alsalem[1], Alistair K. Brown[1], Andrew K. Crowhurst[2], Charlotte A. Taylor[3], Luka G. Larkin[3], Jason H. Gill[3,4], Jonathan D. Sellars[1,3*]

1 Biosciences Institute, Faculty of Medical Sciences, Newcastle University, Catherine Cookson Building, Framlington Place, Newcastle upon Tyne, NE2 4HH, UK.

2 Chemistry, School of Natural & Environmental Sciences, Newcastle University, Newcastle upon Tyne, NE1 7RU, UK

3 School of Pharmacy, Faculty of Medical Sciences, Newcastle University, King George VI Building, Newcastle upon Tyne, NE1 7RU, UK.

4 Translational and Clinical Research Institute, Faculty of Medical Sciences, Newcastle University, Newcastle upon Tyne NE2 4HH, UK

* Correspondence: jon.sellars@newcastle.ac.uk; Tel: +44 (191) 2082357

Table of Contents

S1. Reordered Table 1 2

S2. Experimental Procedures 4

Synthesis of Amino acid hydrazides 4

Synthesis of 3,5-dinitro benzoyl amino acid hydrazides 4

S3. Biological Data and Experimental Procedures 32

Mycobacterial culture 32

Minimum inhibition concentration (MIC_{95}). 32

S4. Cytotoxicity testing 33

S5. Molecular modelling with *Mycobacterium tuberculosis* DprE1 33

S6. ^{1}H and ^{13}C NMR for all compounds 35

S1. Reordered Table 1

Reordered Table 1 to show REMA assay results showing MIC_{95} values against wild-type and drug-resistant strains of Mtb for the 3,5-dinitrobenzoyl amino acid hydrazides grouped by substitution on the amino acid hydrazide.

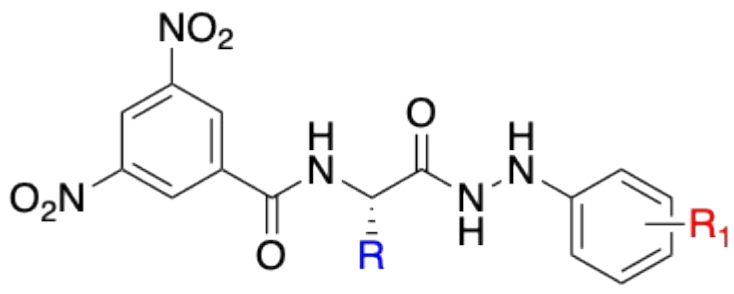

Compound	R	R_1	MIC_{95} (μM)			
			Mtb WT $mc^2 7902$	*Mtb* INH^R $mc^2 8245$	*Mtb* RIF^R $mc^2 8247$	*Mtb* INH^R RIF^R $mc^2 8250$
**4**	Met	4-CF_3	8	8	4	-
**11**	Gly	2-Br	18	18	18	18
**19**	Ala	2-Br	35	35	35	35
**25**	Leu	2-Br	16	16	32	8
**33**	Phe	2-Br	>100	>100	>100	>100
**47**	Hph	2-Br	2	2	2	2
**41**	Met	2-Br	16	16	16	16
**12**	Gly	2-CF_3	37	37	37	37
**20**	Ala	2-CF_3	18	18	18	18
**26**	Leu	2-CF_3	8	8	8	8
**34**	Phe	2-CF_3	4	4	4	4
**48**	Hph	2-CF_3	30	15	30	30
**42**	Met	2-CF_3	16	16	16	16
**13**	Gly	3-Cl	41	20	>100	41
**27**	Leu	3-Cl	9	9	9	9
**35**	Phe	3-Cl	8	4	8	4
**49**	Hph	3-Cl	8	8	8	8
**14**	Gly	3-Br	>100	>100	>100	>100
**21**	Ala	3-Br	18	18	18	18
**28**	Leu	3-Br	64	64	>100	>100
**36**	Phe	3-Br	15	30	30	30
**50**	Hph	3-Br	4	4	7	7
**43**	Met	3-Br	63	63	63	31
**15**	Gly	3-CF_3	19	9	37	9
**22**	Ala	3-CF_3	36	36	36	36
**29**	Leu	3-CF_3	8	8	17	17
**37**	Phe	3-CF_3	>100	>100	>100	>100
**51**	Hph	3-CF_3	60	30	60	60
**44**	Met	3-CF_3	16	16	64	32
**16**	Gly	4-Cl	41	10	41	20

**23**	Ala	4-Cl	20	10	39	20
**30**	Leu	4-Cl	18	9	18	18
**38**	Phe	4-Cl	17	17	33	8
**52**	Hph	4-Cl	16	16	32	16
**45**	Met	4-Cl	34	16	34	34
**17**	Gly	4-Br	9	9	9	9
**24**	Ala	4-Br	35	35	35	35
**31**	Leu	4-Br	16	8	16	16
**39**	Phe	4-Br	>100	>100	>100	>100
**53**	Hph	4-Br	15	7	30	30
**46**	Met	4-Br	>100	63	>100	>100
**18**	Gly	4-CF_3	19	9	37	9
**32**	Leu	4-CF_3	8	4	8	8
**40**	Phe	4-CF_3	16	16	16	16
**54**	Hph	4-CF_3	15	8	30	8
4	*Met*	*4-CF_3*	*8*	*8*	*4*	-

Isoniazid	0.07	>100	0.15	>100
Rifampicin	0.02	0.02	>100	>100
Ethambutol	0.79	9.79	9.79	9.79
Ethionamide	3.01	3.01	9.62	3.01
Erythromycin	8.72	2.73	5.45	5.45
Kanamycin	>100	>100	>100	>100

MIC_{95} value

<10 μM	
5-10 μM	
10-50 μM	
50-100 μM	
>100 μM	

S2. Experimental Procedures

Synthesis of Amino acid hydrazides

Under a nitrogen atmosphere, *N*-protected amino acid (1 equiv.) dissolved in THF (5 mL) at room temperature and treated with DIPEA (1.5 equiv.) followed by HBTU (1.2 equiv.). After stirring for 10 minutes at room temperature addition of N-Aryl hydrazine (1.1 equiv.) continued stirring for another 5 hours at room temperature. The reaction was mixed with ethyl acetate (8 mL) and distilled water (5 mL). After separating the two phases, the organic layer was washed again with distilled water (5 mL x 3 mL) followed by a wash with sat. aq. NH_4Cl (6 mL) then sat. aq. $NaHCO_3$ (6 mL) followed by brine (8 mL). The organic layer dried over $MgSO_4$, filtered, evaporated and dried in *vacuo* achieving the desired *N*-Boc amino acid hydrazides.

Synthesis of 3,5-dinitro benzoyl amino acid hydrazides

The *N*-Boc amino acid hydrazides were dissolved in 4 N HCl solution in dioxane (2 mL) and stirred at room temperature for 90 minutes. Afterwards, the solvent was evaporated, dried *in vacuo* and the hydrochloride salt precipitated via using (EtOH/Et_2O). The precipitate was used in the next step without further purification. The amino acid hydrazide salt (1 equiv.) was dissolved in THF (3 mL) and treated with diisopropylethylamine (3 equiv.) and HBTU (1.2 equiv.). The mixture was stirred for 10 minutes at room temperature, then the solution was treated with 3,5-dinitrobenzoic acid (1.2 equiv.) and continued stirring for 4 hours. The reaction was mixed with ethyl acetate (10 mL) and distilled water (5 mL). After separating the two phases the organic layer was washed again with distilled water (5 mL x 3 mL) followed by sat. aq. NH_4Cl (6 mL) then sat. aq. $NaHCO_3$ (6 mL) and finally with brine (10 mL). The organic layer was dried over $MgSO_4$, filtered, evaporated and dried in *vacuo*. Purification by trituration with (Et_2O or DCM) or through flash chromatography (*n*-hexane/EtOAc [2:1]); (DCM/EtOH/NH_3 [600:8:1], [200:6:1]) afforded the desired compounds

***N*-(2-(2-(2-bromophenyl)hydrazineyl)-2-oxoethyl)-3,5-dinitrobenzamide, 11**

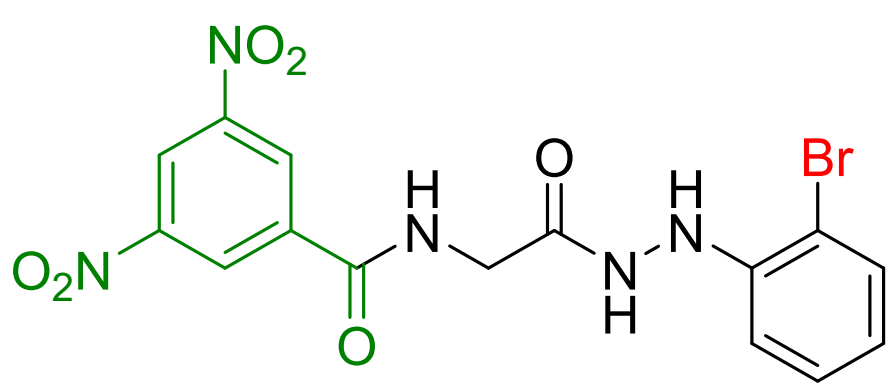

Following the general standard procedure*, tert*-butyl (2-(2-(2-bromophenyl)hydrazineyl)-2-oxoethyl)carbamate (0.10 g, 0.29 mmol) was transformed following trituration with Et_2O to produce the title compound as a yellow solid (0.07 g, 58 %); R_f 0.53 (DCM/EtOH/NH_3 [200:6:1]); m.p. 91 -94 °C; ν_{max} 3290, 1667, 1531, 1395, 1333, 1218, 1159, 1067, 810 cm^{-1}; δ_H (300 MHz, DMSO-d_6) 10.13 (1H, d, *J* 2, CON*H*NH), 9.68 (1H, t, *J* 6, CON*H*), 9.10 (2H, d, *J*

2, Ar-*H*), 8.99 (1H, t, *J* 2, Ar-*H*), 7.44 (1H, dd, *J* 8, 2, Ar-*H*), 7.25 (1H, s, CONHN*H*), 7.20 (1H, m, Ar-*H*), 6.87 (1H, dd, *J* 8., 2, Ar-*H*), 6.71 (1H, td, *J* 8, 2, Ar-*H*), 4.08 (2H, d, *J* 6, CONHC*H*$_2$). δ_C (75 MHz, DMSO-d$_6$) 168.8 (*C*ONHNH), 163.5 (*C*ONH), 148.6 (*ipso*-Ar-*C*), 145.9 (*ipso*-Ar-*C*), 136.7 (*ipso*-Ar-*C*), 132.8 (Ar-*C*), 128.7 (Ar-*C*), 128.2 (Ar-*C*), 121.5 (Ar-*C*) 120.7 (Ar-*C*), 113.8 (Ar-*C*), 107.4 (*ipso*-Ar-*C*), 42.4 (CONH*C*H$_2$); *m/z* (ES$^+$) 438 ([^{79}Br]MH$^+$), 440 ([^{81}Br]MH$^+$); HRMS (ES$^+$) Found [^{79}Br]MH$^+$, 438.0034 ($C_{15}H_{13}{}^{79}BrN_5O_6$ require 438.0049).

3,5-dinitro-*N*-(2-oxo-2-(2-(2-(trifluoromethyl)phenyl)hydrazineyl)ethyl)benzamide, 12

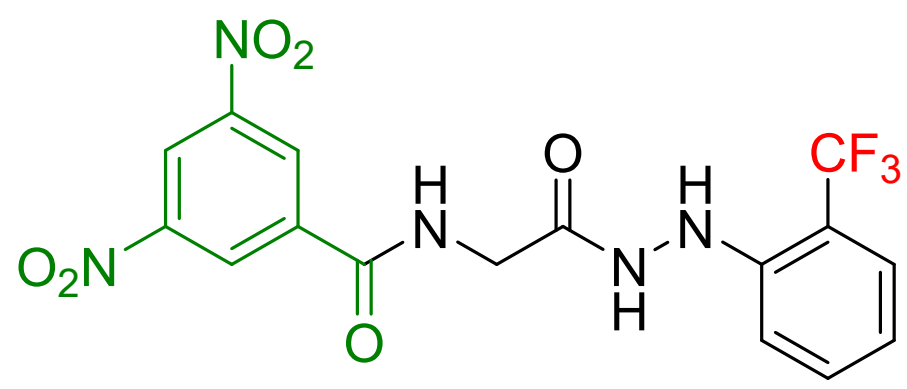

Following the general standard procedure, *tert*-butyl (2-oxo-2-(2-(2-(trifluoromethyl)phenyl)hydrazineyl)ethyl)carbamate (0.10 g, 0.30 mmol) was transformed following trituration with Et$_2$O to produce the title compound as a light yellow solid (0.06 g, 49 %); R$_f$ 0.34 (*n*-hexane/EtOAc) [3:1]); m.p. 89 - 91 °C; v_{max} 3087, 1664, 1529, 1345, 1271, 1095, 748 cm^{-1}; δ_H (300 MHz, DMSO-d$_6$) 10.14 (1H, d, *J* 2, CON*H*NH), 9.70 (1H, t, *J* 6, CON*H*), 9.10 (2H, d, *J* 2, Ar-*H*), 8.98 (1H, t, *J* 2, Ar-*H*), 7.53 (1H, s, CONHN*H*), 7.46 (2H, t, *J* 8, Ar-*H*), 7.03 (1H, d, *J* 8, Ar-*H*), 6.87 (1H, t, *J* 8, Ar-*H*), 4.08 (2H, d, *J* 6, CONHC*H$_2$*); δ_C (75 MHz, DMSO-d$_6$) 168.9 (*C*ONHNH), 163.5 (*C*ONH), 148.6 (*ipso*-Ar-*C*),146.5 (*ipso*-Ar-*C*), 137.1 (*ipso*-Ar-*C*), 133.8 (Ar-*C*), 128.2 (Ar-*C*),126.5 (*ipso*-Ar-C*CF$_3$*), 121.5 (Ar-*C*), 118.7 (Ar-*C*), 113.7 (Ar-*C*) 112.3 (*ipso*-Ar-*C*). 42.5 (CONH*C*H$_2$); δ_F (282 MHz, DMSO-d$_6$) -59.30 (3F, s, CF$_3$); *m/z* (ES$^+$) 427 (MH$^+$); HRMS (ES$^+$) found MH$^+$, 428.0579 ($C_{16}H_{13}F_3N_5O_6$ requires 428.0818).

***N*-(2-(2-(3-chlorophenyl)hydrazineyl)-2-oxoethyl)-3,5-dinitrobenzamide, 13**

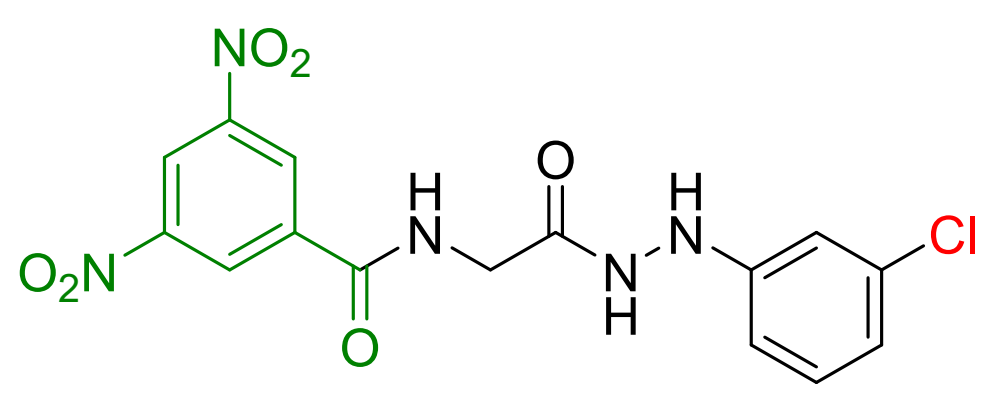

Following the general standard procedure, *tert*-butyl (2-(2-(3-chlorophenyl)hydrazineyl)-2-oxoethyl)carbamate (0.11 g, 0.37 mmol) was transformed following flash column chromatography (*n*-hexane/EtOAc) [4:1] to produce the title product as yellow solid (0.07 g, 46 %); R$_f$ 0.31 (DCM/EtOH/NH$_3$ [200:6:1]); m.p. 90 - 91 °C; v_{max} 3303, 3101, 1594, 1488, 1249,1092, 1010, 1092, 918, 822; δ_H (300 MHz, DMSO-d$_6$) 9.98 (1H, d, *J* 2, CON*H*NH), 9.68 (1H, t, *J* 6, CON*H*), 9.11 (2H, d, *J* 2, Ar-*H*), 8.99 (1H, t, *J* 2, Ar-*H*), 8.06 (1H, d, *J* 2, CONHN*H*), 7.09 (1H, t, *J* 8.0, Ar-*H*), 6.92 (1H, t, *J* 2, Ar-*H*), 6.85 (1H, dt, *J* 8, 1, Ar-*H*), 6.74 (1H, dt, *J* 8, Ar-

H), 4.06 (1H, d, *J* 6,CONHC*H*$_2$); δ_C (75 MHz, DMSO-d$_6$) 168.9 (*C*ONHNH), 163.5 (*C*ONH), 151.4 (*ipso*-Ar-*C*), 148.6 (*ipso*-Ar-*C*), 137.1 (*ipso*-Ar-*C*), 131.1 (Ar-*C*), 128.9 (Ar-*C*), 122.6 (Ar-*C*), 121.5 (Ar-*C*), 121.3 (Ar-*C*), 114.8 (Ar-*C*), 111.7 (Ar-*C*), 42.5 (*C*ONHCH$_2$); *m/z* (ES$^+$) 394 ([^{35}Cl]MH$^+$), 396 ([^{37}Cl]MH$^+$); HRMS (ES$^+$) Found [^{35}Cl]MH$^+$, 394.0536 ($C_{15}H_{13}{}^{35}ClN_5O_6$ require 394.0555).

N-(2-(2-(3-bromophenyl)hydrazineyl)-2-oxoethyl)-3,5-dinitrobenzamide, 14

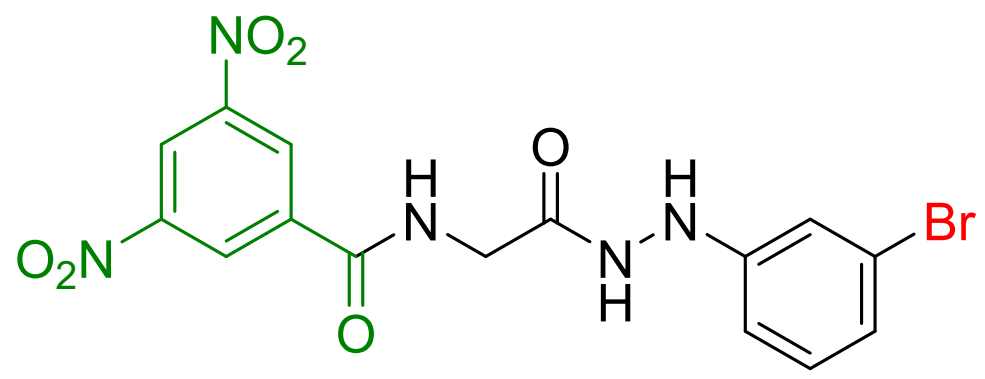

Following the general standard procedure, *tert*-butyl (2-(2-(3-bromophenyl)hydrazineyl)-2-oxoethyl)carbamate (0.10 g, 0.29 mmol) was transformed following flash column chromatography (*n*-hexane/EtOAc) [4:1] to produce the title compound as a yellow gummy solid (0.07 g, 57 %); R$_f$ 0.31 (DCM/EtOH [200:6]); m.p. 94 -95 °C; v_{max} 3282, 1682, 1599, 1477, 1248, 1151, 1044, 992, 864, 768, 434 cm^{-1}; δ_H (300 MHz, DMSO-d$_6$) 9.98 (1H, d, *J* 2, CON*H*NH), 9.68 (1H, t, *J* 6, CON*H*), 9.11 (2H, d, *J* 2, Ar-*H*), 8.99 (1H, t, *J* 2, Ar-*H*), 8.06 (1H, d, *J* 2, CONHN*H*), 7.09 (1H, t, *J* 8, Ar-*H*), 6.92 (1H, t, *J* 2, Ar-*H*), 6.85 (1H, dt, *J* 8, 1, Ar-*H*), 6.74 (1H, dt, *J* 8, 1, Ar-*H*), 4.06 (1H, d, *J* 6,CONHC*H*$_2$); δ_C (75 MHz, DMSO-d$_6$) 168.9 (*C*ONHNH), 163.5 (*C*ONH), 151.4 (*ipso*-Ar-*C*), 148.6 (*ipso*-Ar-*C*), 137.1 (*ipso*-Ar-*C*), 131.1 (Ar-*C*), 128.9 (Ar-*C*), 122.6 (*ipso*-Ar-*C*), 121.5 (Ar-*C*), 121.3 (Ar-*C*), 114.8 (Ar-*C*), 111.7 (Ar-*C*), 42.5 (CONH*C*H$_2$); *m/z* (ES$^+$) 438 ([^{79}Br]MH$^+$), 440 ([^{81}Br]MH$^+$); HRMS (ES$^+$) Found [^{79}Br]MH$^+$, 438.0804 ($C_{15}H_{13}{}^{79}BrN_5O_6$ require 438.0049).

3,5-dinitro-*N*-(2-oxo-2-(2-(3-(trifluoromethyl)phenyl)hydrazineyl)ethyl)benzamide, 15

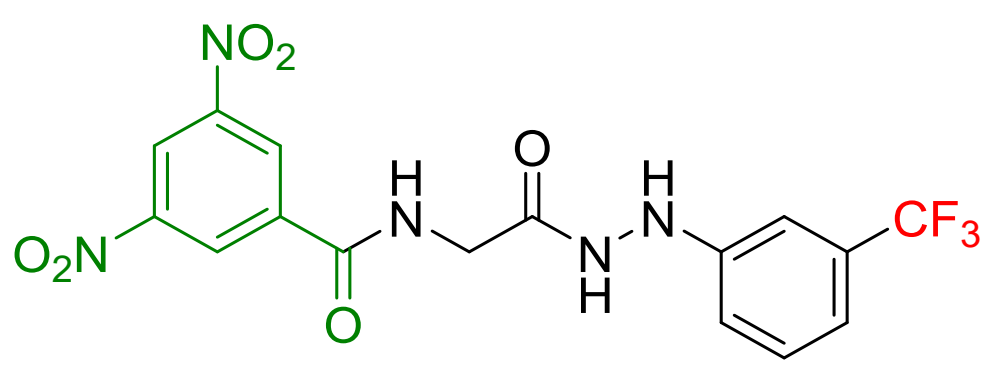

Following the general standard procedure, *tert*-butyl (2-oxo-2-(2-(3-(trifluoromethyl)phenyl)hydrazineyl)ethyl)carbamate (0.10 g, 0.30 mmol) was transformed following flash column chromatography (DCM/EtOAc [3:1]) to produce the title compound as a yellow solid (0.06 g, 49 %); R$_f$ 0.36 (DCM/EtOH/NH$_3$ [200:6:1]); m.p. 96 – 97 °C; v_{max} 3322, 1629, 1533, 1344, 1167, 1065, 1109, 748, 699 cm^{-1}; δ_H (300 MHz, DMSO-d$_6$) 10.05 (1H, d, *J* 2, CON*H*NH), 9.70 (1H, t, *J* 6, CON*H*), 9.10 (2H, d, *J* 2, Ar-*H*), 8.98 (1H, t, *J* 2, Ar-*H*), 8.23 (1H, d, *J* 2, CONHN*H*), 7.36 (1H, t, *J* 8, Ar-*H*), 7.03 (2H, d, *J* 5, Ar-*H*), 7.00 – 6.98 (1H, m,

Ar-*H*), 4.06 (2H, d, *J* 6, CONHC*H*$_2$) (75 MHz, DMSO-d$_6$) 169 (*C*ONHNH), 163.5 (*C*ONH), 150.33 (*ipso*-Ar-*C*),148.6 (*ipso*-Ar-*C*), 137.1 (*ipso*-Ar-*C*), 130.2 (Ar-*C*), 128.2 (Ar-*C*), 121.4 (Ar-*C*), 116.3 (Ar-*C*) 114.9 (*ipso*-Ar-*C*), 108.4 (Ar-*C*) 42.5 (CONHC*H*$_2$); δ_F (282 MHz, DMSO-d$_6$) -59.29 (3F, s, CF$_3$); *m/z* (ES+) 427 (MH$^+$); HRMS (ES$^+$) found MH$^+$, 428.0796 ($C_{16}H_{13}F_3N_5O_6$ requires 428.0818).

***N*-(2-(2-(4-chlorophenyl)hydrazineyl)-2-oxoethyl)-3,5-dinitrobenzamide, 16**

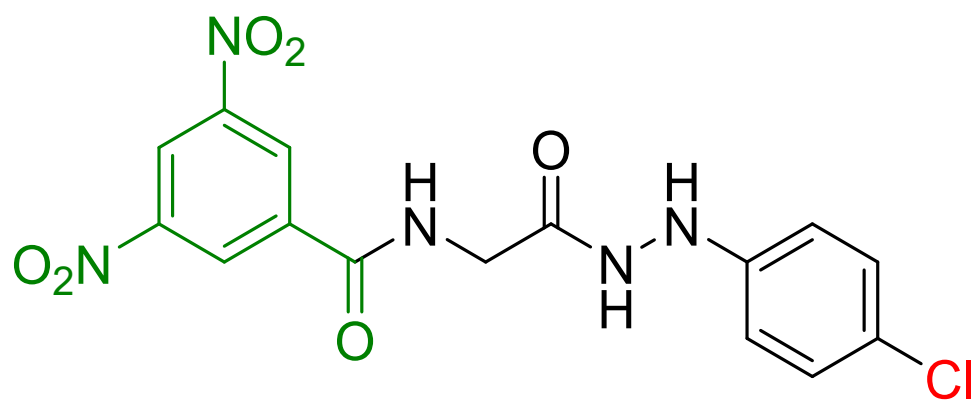

Following the general standard procedure, *tert*-butyl (2-(2-(4-chlorophenyl)hydrazineyl)-2-oxoethyl)carbamate (0.10 g, 0.33 mmol) was transformed following flash column chromatography (*n*-hexane/EtOAc) [3:1] to produce the title product as a light yellow solid (0.03 g, 26 %); R$_f$ 0.47 (DCM/EtOH/NH$_2$ [200:6:1]); m.p. 90 - 92 °C; ν_{max} 3272, 1630, 1549, 1423, 1249, 1097, 1010, 748, 699 cm^{-1}; δ_H (700 MHz, DMSO-d$_6$) 9.98 (1H, d, *J* 2, CON*H*NH), 9.67 (1H, t, *J* 6, CON*H*), 9.08 (2H, d, *J* 2, Ar-*H*), 8.98 (1H, d, *J* 2, Ar-*H*), 7.95 (1H, d, *J* 2, CONHN*H*), 7.20 (2H, d, *J* 9, Ar-*H*), 6.75 (2H, d, *J* 9, Ar-*H*), 4.04 (2H, d, *J* 6, CONHC*H*$_2$); δ_C (700 MHz, DMSO-d$_6$) 168.3 (*C*ONHNH), 162.9 (*C*ONH), 148.6 (*ipso*-Ar-*C*), 136.5 (*ipso*-Ar-*C*), 128.3.1 (Ar-*C*), 127.6 (Ar-*C*), 121.7 (Ar-*C*), 120.9 (Ar-*C*), 113.6 (Ar-*C*), 42.5 (*C*ONHCH$_2$) 168.8 (*C*ONHNH), 163.4 (*C*ONH), 148.6 (*ipso*-Ar-*C*), 148.6 (*ipso*-Ar-*C*), 137.1 (*ipso*-Ar-*C*), 128.9 (Ar-*C*), 128.2 (Ar-*C*), 122.2 (Ar-*C*), 121.5 (Ar-*C*), 114.1 (Ar-*C*), 42.4 (CONHC*H*$_2$).; *m/z* (ES$^+$) 394 ([^{35}Cl]MH$^+$), 396 ([^{37}Cl]MH$^+$); HRMS (ES$^+$) Found [^{35}Cl]MH$^+$, 394.0536 ($C_{15}H_{13}{}^{35}ClN_5O_6$ require 394.0554).

***N*-(2-(2-(4-bromophenyl)hydrazineyl)-2-oxoethyl)-3,5-dinitrobenzamide, 17**

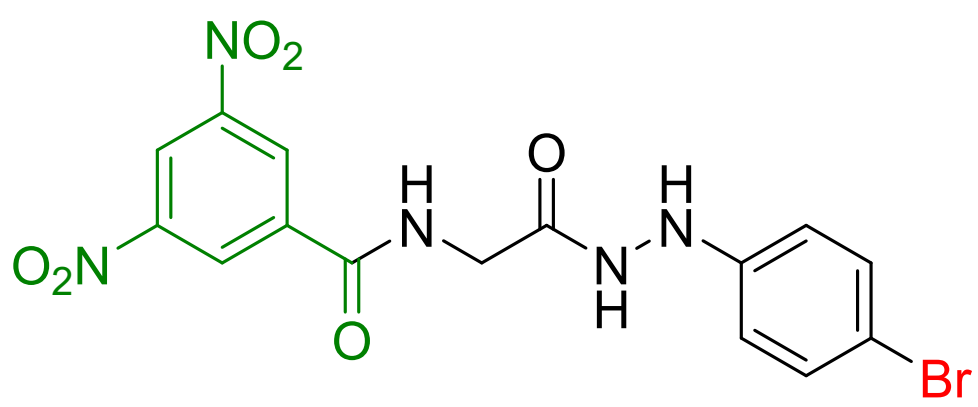

Following the general standard procedure, *tert*-butyl (2-(2-(4-bromophenyl)hydrazineyl)-2-oxoethyl)carbamate (0.09 g, 0.26 mmol) was transformed following flash column chromatography (*n*-hexane/EtOAc) [3:1] to produce the title product as a light yellow solid (0.04 g, 37 %); R$_f$ 0.32 (DCM/EtOH/NH$_2$ [200:6:1]); m.p. 90 - 92 °C; ν_{max} 3272, 1639, 1538, 1323, 1277, 1097, 1034, 748, 699 cm^{-1}; δ_H (300 MHz, DMSO-d$_6$) 9.97 (1H, d, *J* 2, CON*H*NH), 9.66 (1H, t, *J* 6, CON*H*), 9.11 (2H, d, *J* 2, Ar-*H*), 8.99 (1H, d, *J* 2, Ar-*H*), 7.97 (1H, d, *J* 2,

CONHN*H*), 7.19 (2H, d, *J* 9, Ar-*H*), 6.77 (2H, d, *J* 9, Ar-*H*), 4.06 (2H, d, *J* 6, CONHC*H*$_2$); δ_C (75 MHz, DMSO-d$_6$) 168.8 (*C*ONHNH), 163.4 (*C*ONH), 148.6 (*ipso*-Ar-*C*), 137.1 (*ipso*-Ar-*C*), 128.9 (Ar-*C*), 128.2 (Ar-*C*), 122.2 (*ipso*-Ar-*C*), 121.5 (Ar-*C*), 114.1 (Ar-*C*), 42.4 (CONH*C*H$_2$).; *m/z* (ES$^+$) 438 ([^{79}Br]MH$^+$), 440 ([^{81}Br]MH$^+$); HRMS (ES$^+$) Found [^{79}Br]MH$^+$, 438.0020 ($C_{15}H_{13}{}^{79}BrN_5O_6$ require 438.0049).

3,5-dinitro-*N*-(2-oxo-2-(2-(4-(trifluoromethyl)phenyl)hydrazineyl)ethyl)benzamide, 18

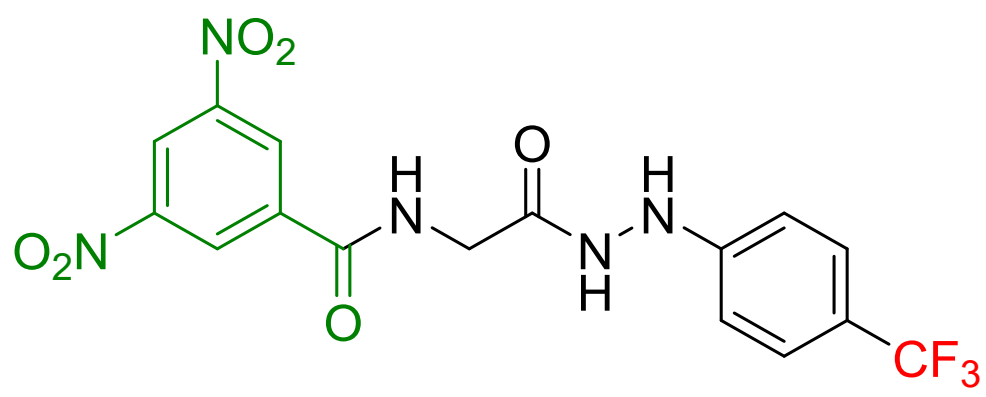

Following the general standard procedure, *tert*-butyl (2-oxo-2-(2-(4-(trifluoromethyl)phenyl)hydrazineyl)ethyl)carbamate (0.10 g, 0.30 mmol) was transformed following trituration with Et_2O to produce the title compound as a yellow solid (0.06 g, 49 %); R$_f$ 0.36 (DCM/EtOH/NH_3 [200:6:1]); m.p. 95 – 96 °C; v_{max} 3272, 1639, 1538, 1323, 1277, 1097, 1034, 748, 699 cm^{-1}; δ_H (300 MHz, DMSO-d$_6$) 10.09 – 10.06 (1H, m, CON*H*NH), 9.69 (1H, t, *J* 6, CON*H*), 9.10 (2H, d, *J* 2., Ar-*H*), 8.99 (1H, t, *J* 2, Ar-*H*), 8.42 (1H, s, CONHN*H*), 7.47 (2H, d, *J* 9, Ar-*H*), 6.86 (2H, d, *J* 9 Ar-*H*), 4.08 (1H, d, *J* 6, CONHC*H*$_2$). δ_C (75 MHz, DMSO-d$_6$) 168.9 (*C*ONHNH), 163.5 (*C*ONH), 152.8 (*ipso*-Ar-*C*)148.6 (*ipso*-Ar-*C*),137.1 (*ipso*-Ar-*C*), 128.2 (Ar-*C*),126.6 (Ar-*C*), 121.5 (Ar-*C*), 118.9 (*ipso*-Ar-C*C*F$_3$), 111.0 (Ar-*C*). 42.4 (CONH*C*H$_2$); δ_F (282 MHz, DMSO-d$_6$) -61.30 (3F, s, CF_3); *m/z* (ES$^+$) 428 (MH$^+$); HRMS (ES$^+$) found MH$^+$, 428.0808 ($C_{16}H_{13}F_3N_5O_6$ requires 427.0812).

(S)-*N*-(1-(2-(2-bromophenyl)hydrazineyl)-1-oxopropan-2-yl)-3,5-dinitrobenzamide, 19

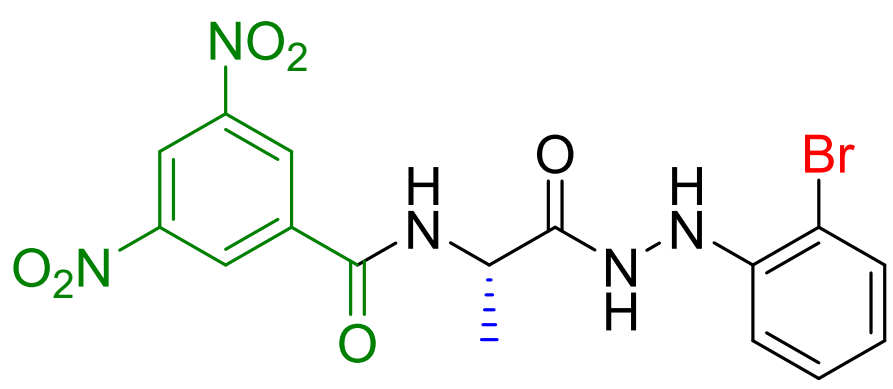

Following the general standard procedure, *tert*-butyl (S)-(1-(2-(2-bromophenyl)hydrazineyl)-1-oxopropan-2-yl)carbamate (0.10 g, 0.28 mmol) was transformed to produce the title compound as a yellow solid, (0.09 g, 68 %); R$_f$ 0.65 (*n*-hexane/EtOAc) [2:1]; m.p. 101 - 102 °C; v_{max} 2155, 1638,1344, 1165, 1095, 728 cm^{-1}; δ_H (300 MHz, DMSO-d$_6$) 10.22 (1H, d, *J* 2, CON*H*NH), 9.52 (1H, d, *J* 7 , CON*H*), 9.19 (2H, d, *J* 2, Ar-*H*), 9.03 (1H, t, *J* 2., Ar-*H*), 7.49 (1H, dd, *J* 8, 1., Ar-*H*), 7.32 – 7.20 (1H, m, Ar-*H*), 6.91 (1H, dd, *J* 8., 2, Ar-*H*), 6.75 (1H, td, *J* 8, 2 , Ar-*H*), 4.67 (1H, q, *J* 7, CONHC*H*CH$_3$), 1.54 (3H, d, *J* 7, CONHCHC*H*$_3$); δ_C (75 MHz, DMSO-d$_6$) 172.3 CONHNH), 163 (*C*ONH), 148.6 (*ipso*-Ar-*C*), 146.(*ipso*-Ar-*C*), 137.1 (*ipso*-Ar-*C*), 132.8

(Ar-*C*), 128.8 (Ar-*C*), 128.4 (*ipso*-Ar-*C*), 121.5 (Ar-*C*), 120.7 (*ipso*-Ar-*C*), 107.4 (Ar-*C*), 49.2 (CONH*C*HCH$_3$), 18.0 (CONHCH*C*H$_3$); *m/z* (ES$^+$) 452 ([^{79}Br]MH$^+$) 454 ([^{81}Br]MH$^+$); HRMS (ES$^+$) found [^{79}Br] MH$^+$, 452.2200 ($C_{16}H_{15}{}^{79}BrN_5O_6$ requires 452.0206).

(S)-3,5-dinitro-*N*-(1-oxo-1-(2-(2-(trifluoromethyl)phenyl)hydrazineyl)propan-2-yl)benzamide, 20

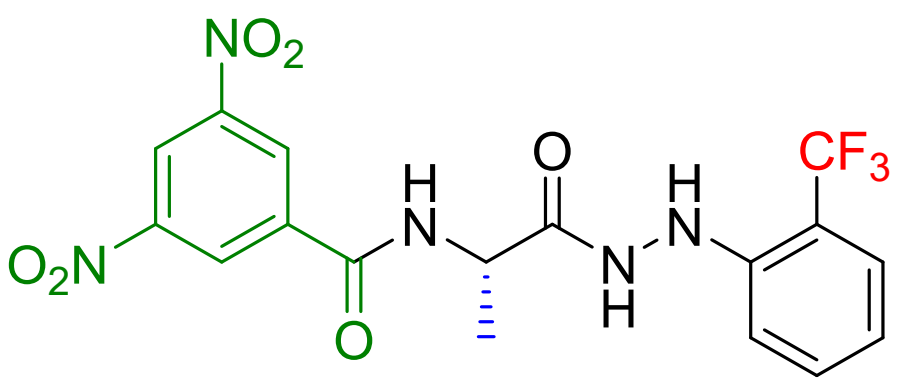

Following the general standard procedure, *tert*-butyl (S)-(1-oxo-1-(2-(2-(trifluoromethyl)phenyl)hydrazineyl)propan-2-yl)carbamate (0.10 g, 0.29 mmol) was transformed following trituration with Et$_2$O to produce the title compound as an off white solid (0.03 g, 21 %); R$_f$ 0.29 (*n*-hexane/EtOAc) [4:1]; m.p. 98 - 100 °C; ν_{max} 3272, 1639, 1503, 1459, 1342, 1286, 1127,1035, 940, 755, 645, cm^{-1}; δ_H (300 MHz, DMSO-d$_6$) 10.18 (1H, d, *J* 2 ,CON*H*NH), 9.48 (1H, d, *J* 7,CON*H*), 9.15 (2H, d, *J* 2 ,Ar-*H*), 8.99 (1H, t, *J* 2, Ar-*H*), 7.35 (1H, bs, CONHN*H*), 7.49 – 7.43 (2H, m, Ar-*H*), 7.03 (1H, d, *J* 8, Ar-*H*), 6.87 (1H, t, *J* 8, Ar-*H*), 4.62 (1H, q, *J* 7,CONHC*H*(CH$_3$)), 1.50 (3H, d, *J* 7,CONHCH(C*H*$_3$)).; δ_C (75 MHz, DMSO-d$_6$) 172.2 (*C*ONHNH), 163.0 (*C*ONH), 148.5 (*ipso*-Ar-*C*), 146.6 (*ipso*-Ar-*C*), 137.1 (*ipso*-Ar-*C*), 133.8 (Ar-*C*), 128.4 (Ar-*C*), 126.4 (Ar-*C*), 123.0 (*ipso*-Ar-C*C*F$_3$), 121.4 (Ar-*C*), 118.6 (Ar-*C*), 113.6 (*ipso*-Ar-*C*), 49.2 (CONH*C*H(CH$_3$)), 17.9 (CONHCH(*C*H$_3$)); δ_F (282 MHz, DMSO-d$_6$) -59.30 (3F, s, CF$_3$); *m/z* (ES$^+$) 442 (MH$^+$); HRMS (ES$^+$) found MH$^+$, 442.0731 ($C_{17}H_{15}F_3N_5O_6$ requires 442.0974).

(S)-*N*-(1-(2-(3-bromophenyl)hydrazineyl)-1-oxopropan-2-yl)-3,5-dinitrobenzamide, 21

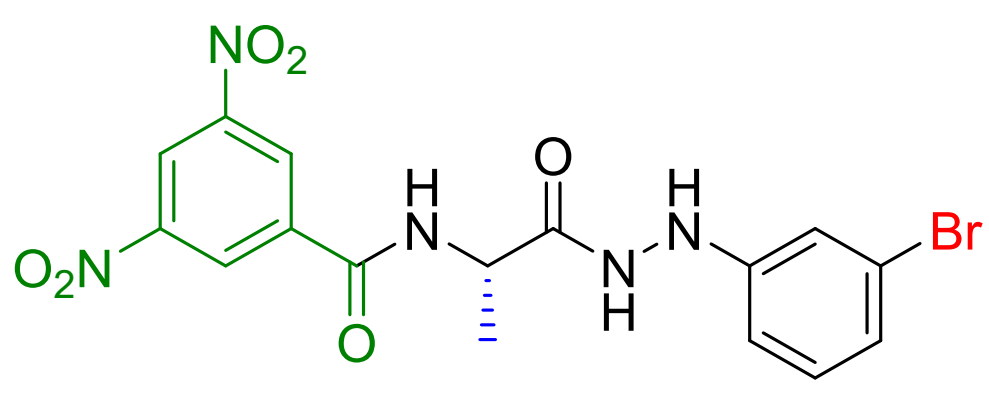

Following the general standard procedure, *tert*-butyl (S)-(1-(2-(3-bromophenyl)hydrazineyl)-1-oxopropan-2-yl)carbamate (0.10g, 0.28 mmol) was transformed to produce the title compound as a pale yellow solid, (0.06 g, 36 %); R$_f$ 0.24 (*n*-hexane/EtOAc) [4:1]; m.p. 98 - 100 °C; ν_{max} 3267, 1683, 1642, 1536, 1476, 1341, 1283, 1224, 1069, 898, cm^{-1}; δ_H (300 MHz, DMSO-d$_6$) 10.03 (1H, s, CON*H*NH), 9.47 (1H, d, *J* 7, CON*H*), 9.17 (2H, d, *J* 2, Ar-*H*), 8.99 (1H, m, Ar-*H*), 8.07 (1H, bs, CONHN*H*), 7.11 (1H, t, *J* 8, Ar-*H*), 6.76 (1H, t, *J* 2, Ar-H), 6.73-6.66 (2H, m, Ar-*H*), 4.58 (1H, q, *J* 7 CONHC*H*(CH$_3$)), 1.48 (3H, d, *J* 7, CONHCH(C*H*$_3$)); δ_C (75 MHz, DMSO-

d_6) 171.9 (*C*ONHNH), 162.5 (*C*ONH), 150.9 (*ipso*-Ar–*C*), 148.1 (*ipso*-Ar-*C*), 136.6 (*ipso*-Ar-*C*), 130.6 (Ar-*C*), 127.9 (Ar-*C*), 122.1 (Ar-*C*), 121.0 (Ar-*C*), 120.7 (Ar-*C*), 114.9 (Ar-*C*), 111.2 (Ar-*C*), 49.2 (CONH*C*H(CH_3)), 17.4 (CONHCH(*C*H_3); *m/z* (ES^+) 452 ([^{79}Br]MH^+) 454 ([^{81}Br]MH^+); HRMS (ES^+) found [^{79}Br] MH^+, 452.2200 ($C_{16}H_{15}{}^{79}BrN_5O_6$ requires 452.0260).

(S)-3,5-dinitro-*N*-(1-oxo-1-(2-(3-(trifluoromethyl)phenyl)hydrazineyl)propan-2-yl)benzamide, 22

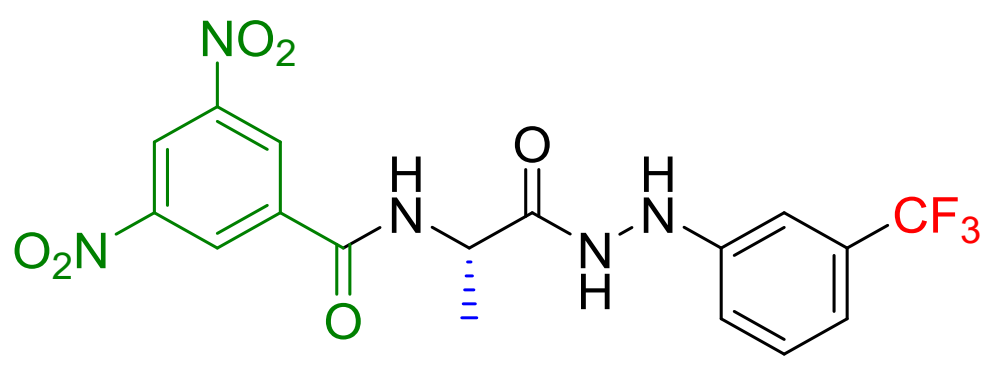

Following the general standard procedure, *tert*-butyl (S)-(1-oxo-1-(2-(3-(trifluoromethyl)phenyl)hydrazineyl)propan-2-yl)carbamate (0.10 g, 0.29 mmol) was transformed following trituration with Et_2O to produce the title compound as a pale yellow solid (0.06 g, 43 %); R_f 0.32 (*n*-hexane/EtOAc) [4:1]; m.p. 103 - 105 °C; v_{max} 3280, 1638, 1540, 1344, 1127, 1070, 920, 785 cm^{-1}; δ_H (300 MHz, DMSO-d_6) 10.11 (1H, d, *J* 2, CON*H*NH), 9.48 (1H, d, *J* 7, CON*H*), 9.15 (2H, d, *J* 2, Ar-*H*), 8.99 (1H, t, *J* 2, Ar-*H*), 8.25 (1H, d, *J* 2, CONHN*H*), 7.36 (1H, t, *J* 8, Ar-*H*), 7.06 – 6.94 (3H, m, Ar-*H*), 4.57 (1H, q, *J* 7, CONHC*H*(CH_3)), 1.49 (3H, d, *J* 7, CONHCH(C*H*$_3$)). δ_C (75 MHz, DMSO-d_6) 172.5 (*C*ONHNH), 163.0 (*C*ONH), 150.4 (*ipso*-Ar-*C*), 148.6 (*ipso*-Ar-*C*), 137.0 (*ipso*-Ar-*C*), 130.2 (Ar-*C*), 128.4 (Ar-*C*), 121.5 (Ar-*C*), 115.3 (*ipso*-Ar-C*C*F_3), 108 (Ar-*C*), 49.3 (CONH*C*H(CH_3)), 17.8 (CONHCH(C*H*$_3$)); δ_F (282 MHz, DMSO-d_6) -61.35 (3F, s, CF_3); *m/z* (ES^+) 442 (MH^+); HRMS (ES^+) found MH^+, 442.0734 ($C_{17}H_{15}F_3N_5O_6$requires 442.0930).

(S)-*N*-(1-(2-(4-chlorophenyl)hydrazineyl)-1-oxopropan-2-yl)-3,5-dinitrobenzamide, 23

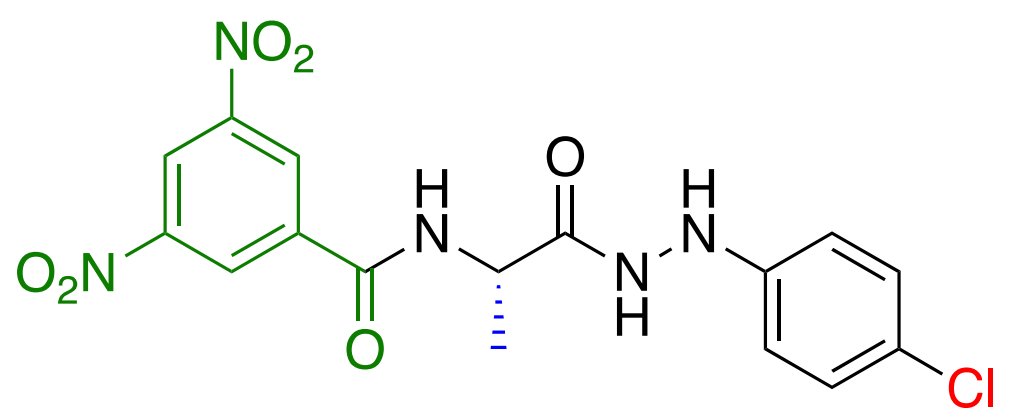

Following the general standard procedure, *tert*-butyl (S)-(1-(2-(4-chlorophenyl)hydrazineyl)-1-oxopropan-2-yl)carbamate (0.12 g, 0.38 mmol) was transformed following trituration with Et_2O to produce the title compound as an off white solid (0.09 g, 56 %); R_f 0.34 (*n*-hexane/EtOAc) [3:1]; m.p. 100 - 102 °C; v_{max} 3302, 1635, 1536, 1487, 1341, 1284, 1230,1055, 959 cm^{-1}; δ_H (300 MHz, DMSO-d_6) 10.00 (1H, d, *J* 3 CON*H*NH), 9.44 (1H, d, *J* 7,CON*H*), 9.13 (2H, d, *J* 2, Ar-*H*), 8.99 (1H, d, *J* 2, Ar-*H*), 7.98 (1H, d, *J* 2, CONHN*H*), 7.29 (2H, d, *J* 9, Ar-*H*), 6.70 (2H, d, *J* 8.8, Ar-*H*), 4.58 (1H, q, *J* 8, CONHC*H*(CH_3)), 1.47 (3H, d, *J* 7, CONHCH(C*H*$_3$)); δ_C (75

MHz, DMSO-d_6) 171.9 (*C*ONHNH), 162.5 (*C*ONH), 149.1 (*ipso*-Ar–*C*), 148.5 (*ipso*-Ar-*C*), 136.7 (*ipso*-Ar-*C*), 131.7 (Ar-*C*), 128.4 (Ar-*C*) 127.9 (Ar-C), 121.7 (Ar-*C*), 121.0 (Ar-C), 113.6 (*ipso*-Ar-*C*), 48.7 (CONH*C*H(CH_3)), 17.6 (CONHCH(*C*H_3)), *m/z* (ES^+) 408 ([^{35}Cl]MH^+), 410 ([^{37}Cl]MH^+); HRMS (ES^+) Found [^{35}Cl]MH^+, 408.0680 ($C_{16}H_{15}{}^{35}ClN_5O_6$ require 408.0711).

(S)-*N*-(1-(2-(4-bromophenyl)hydrazineyl)-1-oxopropan-2-yl)-3,5-dinitrobenzamide, 24

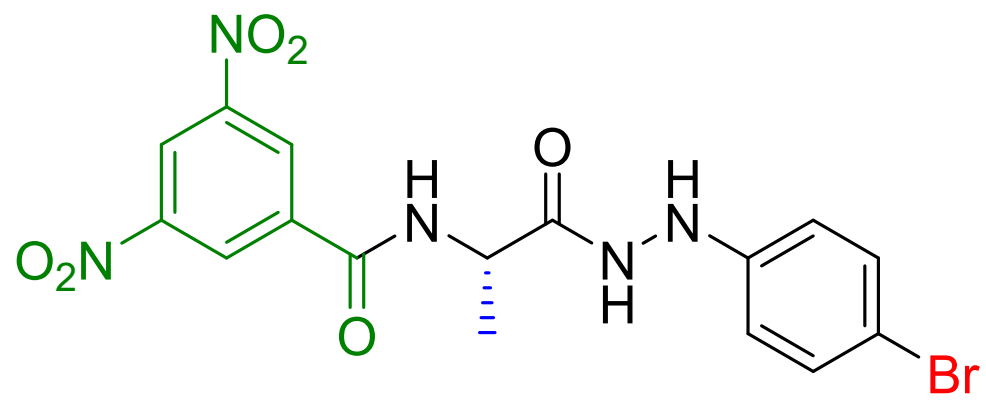

Following the general standard procedure, *tert*-butyl (S)-(1-(2-(4-bromophenyl)hydrazineyl)-1-oxopropan-2-yl)carbamate (0.10 g, 0.27 mmol) was transformed following trituration with Et_2O to produce the title compound as a white solid (0.04 g, 37 %); R_f 0.56 (*n*-hexane/EtOAc) [2:1]; m.p. 107 - 109 °C; ν_{max} 3238, 1640, 1593, 1487, 1342, 1284, 1230,1055, 959, cm^{-1}; δ_H (300 MHz, DMSO-d_6) 10.01 (1H, d, *J* 3 CON*H*NH), 9.44 (1H, d, *J* 7,CON*H*), 9.13 (2H, d, *J* 2, Ar-*H*), 8.99 (1H, d, *J* 2, Ar-*H*), 7.98 (1H, d, *J* 2, CONHN*H*), 7.29 (2H, d, *J* 9, Ar-*H*), 6.70 (2H, d, *J* 8.8, Ar-*H*), 4.58 (1H, q, *J* 8, CONHC*H*(CH_3)), 1.47 (3H, d, *J* 7, CONHCH(C*H*$_3$)); δ_C (75 MHz, DMSO-d_6) 171.8 (*C*ONHNH), 161.1 (*C*ONH), 148.7 (*ipso*-Ar–*C*), 148.1 (*ipso*-Ar-*C*), 136.7 (*ipso*-Ar-*C*), 131.3 (Ar-*C*), 128.0 (Ar-*C*), 121.0 (Ar-*C*), 114.2 (Ar-*C*),109.3 (*ipso*-Ar-*C*), 48.7 (CONH*C*H(CH_3)), 17.6 (CONHCH(*C*H_3)); *m/z* (ES^+) 452 ([^{79}Br]MH^+) 454 ([^{81}Br]MH^+); HRMS (ES^+) found [^{79}Br]MH^+, 452.2200 ($C_{16}H_{15}{}^{79}BrN_5O_6$ requires 452.0206).

(S)-*N*-(1-(2-(2-bromophenyl)hydrazineyl)-4-methyl-1-oxopentan-2-yl)-3,5-dinitrobenzamide, 25

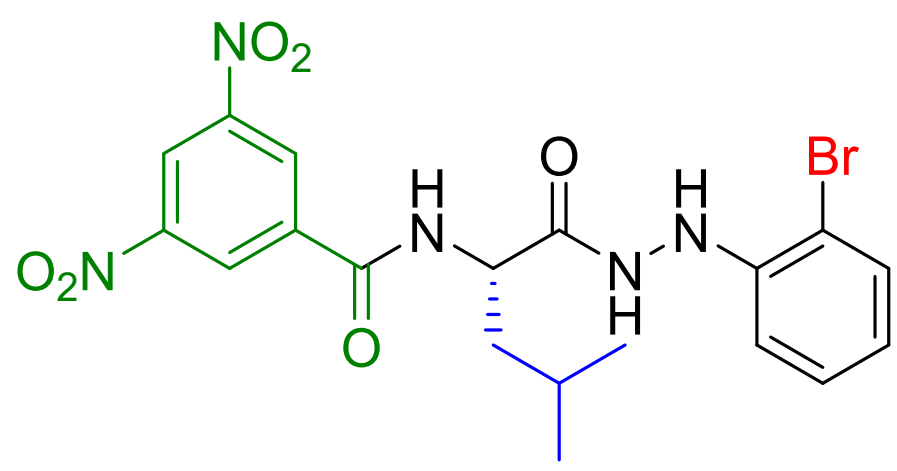

Following the general standard procedure, *tert*-butyl (S)-(1-(2-(2-bromophenyl)hydrazineyl)-4-methyl-1-oxopentan-2-yl)carbamate (0.10 g, 0.25 mmol) was transformed following flash column chromatography to produce the title compound as a pale yellow solid (0.09 g, 63 %); R_f 0.53 (DCM/EtOH/NH_3) [200/6/0.01]; m.p. 90 - 93 °C; ν_{max} 3259, 1644, 1536, 1341, 1076, 1022, 915, 747 cm^{-1}; δ_H (300 MHz, DMSO-d_6) 10.27 (1H, d, *J* 3,CON*H*NH), 9.42 (1H, d, *J* 8, CON*H*), 9.16 (2H, d, *J* 2, Ar-*H*), 8.98 (1H, t, *J* 2, Ar-*H*), 7.44 (1H, dd, *J* 8, 1, Ar-*H*), 7.26 (1H, d, *J* 3,CONHN*H*), 7.21 (1H, t, *J* 7, Ar-*H*), 6.82 (1H, dd, *J* 8, 2, Ar-*H*), 6.70 (1H, td, *J* 8, 2, Ar-*H*),

4.75 – 4.60 (1H, m, CONHC*H*(CH$_2$C*H*(CH$_3$)$_2$)), 1.91 – 1.60 (3H, m, CONHCH(C*H*$_2$C*H*(CH$_3$)$_2$)), 0.96 (6H, dd, *J* 16, 6, CONHCH(CH$_2$CH(C*H*$_3$)$_2$)); δ_C (75 MHz, DMSO-d$_6$) 171.9 (*C*ONHNH), 163.2 (*C*ONH), 148.6 (*ipso*-Ar-*C*), 146.0 (*ipso*-Ar-*C*), 137.0 (*ipso*-Ar-*C*), 132.8 (Ar-*C*), 128.8 (Ar-*C*), 128.4 (Ar-*C*), 121.5 (Ar-*C*), 120.7 (*ipso*-Ar-*C*), 113.6 (Ar-*C*), 107.4 (Ar-*C*), 51.7 (CONH*C*H(CH$_2$CH(CH$_3$)$_2$)), 24.8 (CONHCH(*C*H$_2$CH(CH$_3$)$_2$)), 23.4 (CONHCH(CH$_2$*C*H(CH$_3$)$_2$)), 21.9 (CONHCH(CH$_2$CH(*C*H$_3$)$_2$)); *m/z* (ES$^+$) 494 ([^{79}Br]MH$^+$) 496 ([^{81}Br]MH$^+$); HRMS (ES$^+$) found [^{79} Br] MH$^+$, 494.0652 ($C_{19}H_{21}{}^{79}BrN_5O_6$ requires 494.0675).

(S)-*N*-(4-methyl-1-oxo-1-(2-(2-(trifluoromethyl)phenyl)hydrazineyl)pentan-2-yl)-3,5-dinitrobenzamide, 26

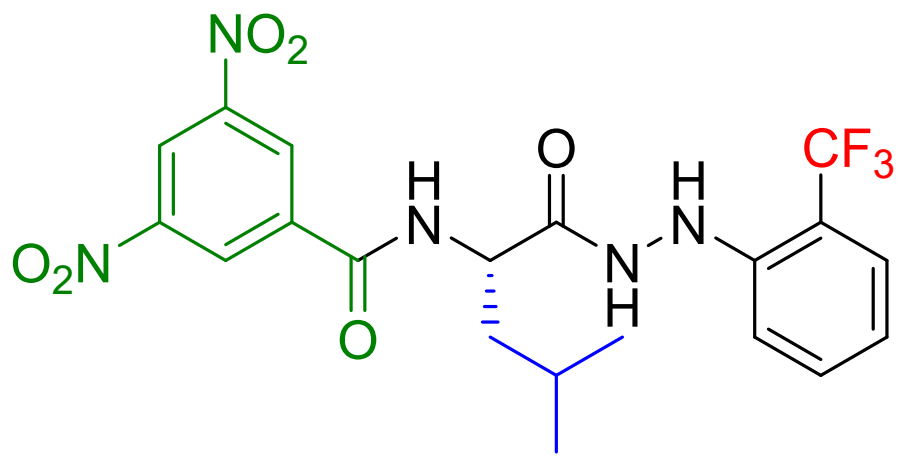

Following the general standard procedure, *tert*-butyl (S)-(4-methyl-1-oxo-1-(2-(2-(trifluoromethyl)phenyl)hydrazineyl)pentan-2-yl)carbamate (0.10 g, 0.26 mmol) was transformed following trituration with DCM to produce the title compound as a white yellowish solid (0.09 g, 70 %); R$_f$ 0.56 (DCM⁄EtOH) [200:8]; m.p. 93 - 94 °C; ν_{max} 3276, 1668, 1593, 1455, 1323, 1155, 1075, 921, 743 cm^{-1}; δ_H (300 MHz, DMSO-d$_6$) 10.20 (1H, d, *J* 2, CON*H*NH), 9.36 (1H, d, *J* 8, CON*H*), 9.09 (2H, d, *J* 2, Ar-*H*), 8.92 (1H, t, *J* 2, Ar-*H*), 7.49 (1H, s, CONHN*H*), 7.44 – 7.36 (2H, m, Ar-*H*), 4.68 – 4.54 (1H, m, CONHC*H*(CH$_2$CH(CH$_3$)$_2$)), 1.87 – 1.55 (3H, m, CONHCH(C*H*$_2$C*H*(CH$_3$)$_2$))), 0.89 (6H, dd, *J* 16, 6, CONH*C*H(CH$_2$CH(C*H*$_3$)$_2$)); δ_C (75 MHz, DMSO-d$_6$) 171.9 (*C*ONHNH), 163.2 (*C*ONH), 148.6 (*ipso*-Ar-*C*), 146.5 (*ipso*-Ar-*C*), 137.0 (*ipso*-Ar-*C*), 133.8 (Ar-*C*), 128.4 (Ar-*C*), 121.5 (*ipso*-Ar-C*CF$_3$*), 118.6 (Ar-*C*), 113.6 (*ipso*-Ar-*C*), 51.8 (CONH*C*H(CH$_2$CH(CH$_3$)$_2$)), 24.8 (CONHCH(*C*H$_2$CH(CH$_3$)$_2$)), 23.4 (CONHCH(CH$_2$*C*H(CH$_3$)$_2$)), 21.8 (CONHCH(CH$_2$CH(*C*H$_3$)$_2$)); δ_F (282 MHz, DMSO-d$_6$) -60.77 (3F, s, CF$_3$); *m/z* (ES$^+$) 484 (MH$^+$); HRMS (ES$^+$) found MH$^+$, 484.1451 ($C_{20}H_{21}F_3N_5O_6$ requires 484.1444).

(S)-N-(1-(2-(3-chlorophenyl)hydrazineyl)-4-methyl-1-oxopentan-2-yl)-3,5-dinitrobenzamide, 27

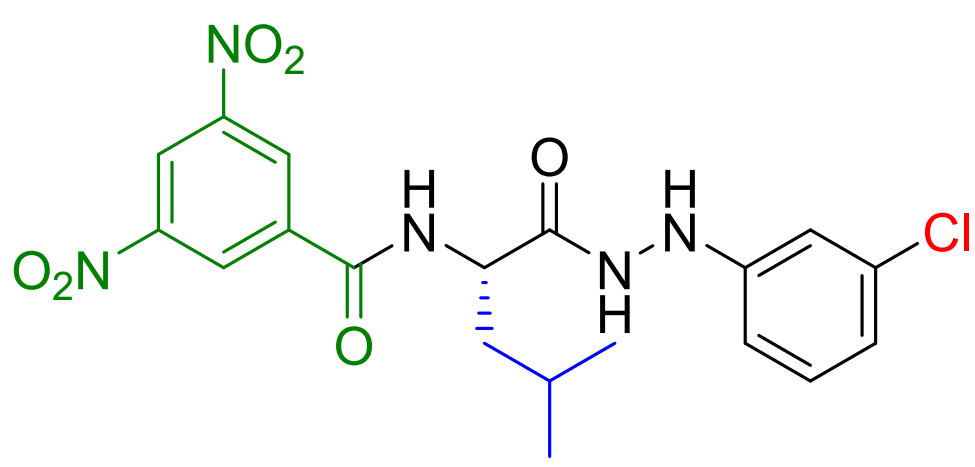

Following the general standard procedure, *tert*-butyl (S)-(1-(2-(3-chlorophenyl)hydrazineyl)-4-methyl-1-oxopentan-2-yl)carbamate (0.13 g, 0.37 mmol) was transformed following trituration with Et_2O produce the title compound as white yellowish solid (0.08 g, 46 %); R_f 0.56 (*n*-hexane/EtOAc) [2:1]; m.p. 90 - 92 °C; ν_{max} 3247, 1665, 1537, 1470, 1340, 1320, 1082, 916 cm^{-1}; δ_H (300 MHz, DMSO-d_6) 10.23 (1H, d, *J* 2, CON*H*NH), 9.42 (1H, d, *J* 7, CON*H*), 9.17 (2H, d, *J* 2, Ar-*H*), 8.99 (1H, t, *J* 2, Ar-*H*), 8.10 (1H, d, *J* 2, CONHN*H*), 7.08 (1H, t, *J* 8, Ar-*H*), 6.90 (1H, t, *J* 2, Ar-*H*), 6.89 – 6.79 (1H, m, Ar-*H*), 6.76 – 6.65 (1H, m, Ar-*H*), 4.68 – 4.55 (1H, m, CONHC*H*($CH_2CHCH_3)_2$)), 1.87 – 1.61 (2H, m, CONHCH(C*H*$_2$CHCH$_2$)$_3$)), 0.99 (3H, d, *J* 6, CONHCH(CH$_2$CHC*H*$_2$)$_3$)), 0.93 (3H, d, *J* 6, CONHCH(CH$_2$CHC*H*$_2$)$_3$)); δ_C (75 MHz, DMSO-d_6) 172.1 (*C*ONHNH) 163.1 (*C*ONH), 148.6 (*ipso*-Ar-*C*), 136.9 (*ipso*-Ar-*C*), 130.4 (Ar-*C*), 130.1 (*ipso*-Ar-*C*), 129.0 (Ar-*C*), 128.4 (Ar-*C*), 126.7 (Ar-*C*), 121.5 (Ar-*C*), 116.4 (Ar-*C*), 115.0 (Ar-*C*), 108.3 (*ipso*-Ar-*C*), 51.9 (CONH*C*H(CH$_2$CH(CH$_3$)$_2$)), 24.8 ((CONHCH(*C*H$_2$CH(CH$_3$)$_2$)), 23.2 ((CONHCH(CH$_2$*C*H(CH$_3$)$_2$)), 22.0 ((CONH*C*H(CH$_2$CH(*C*H$_3$)$_2$)); *m/z* (ES$^+$) 450 ([^{35}Cl]MH$^+$) 452 ([^{37}Cl]MH$^+$); HRMS (ES$^+$) found [^{35}Cl] MH$^+$, 450.1165 ($C_{19}H_{21}{}^{35}ClN_5O_6$ requires 450.1180).

(S)-*N*-(1-(2-(3-bromophenyl)hydrazineyl)-4-methyl-1-oxopentan-2-yl)-3,5-dinitrobenzamide, 28

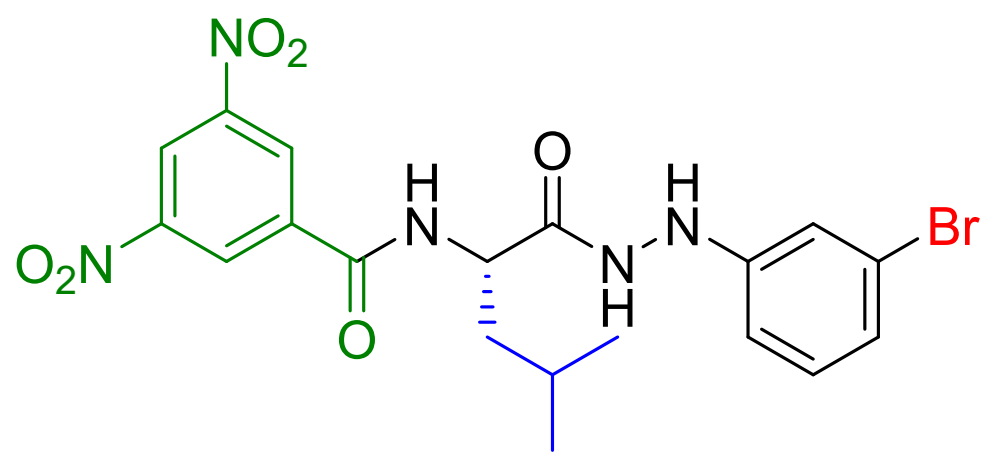

Following the general standard procedure, *tert*-butyl (S)-(1-(2-(3-bromophenyl)hydrazineyl)-4-methyl-1-oxopentan-2-yl)carbamate (0.10 g, 0.25 mmol) was transformed following trituration with Et_2O produce the title compound as a white yellowish solid (0.06 g, 52 %); R_f 0.56 (DCM/EtOH/NH_3) [200:3:1]; m.p. 100 - 102 °C; ν_{max} 3247, 1665, 1537, 1470, 1340, 1320, 1082, 916 cm^{-1}; δ_H (300 MHz, DMSO-d_6) 10.14 (1H, d, *J* 3, CON*H*NH), 9.42 (1H, d, *J* 7, CON*H*), 9.17 (2H, d, *J* 2, Ar-*H*), 8.99 (1H, t, *J* 2, Ar-*H*), 8.10 (1H, d, *J* 3, CONHN*H*), 7.08 (1H, t, *J* 8, Ar-*H*), 6.90 (1H, t, *J* 2, Ar-*H*), 6.89 – 6.79 (1H, m, Ar-*H*), 6.76 – 6.65 (1H, m, Ar-*H*), 4.68 – 4.55 (1H, m, CONHC*H*(CH$_2$CHCH$_3$)$_2$)), 1.87 – 1.61 (2H, m, CONHCH(C*H*$_2$CHCH$_3$)$_2$)), 0.99 (3H, d, *J* 6, CONHCH(CH$_2$CHC*H*$_3$)$_2$)), 0.93 (3H, d, *J* 6, CONHCH(CH$_2$CHC*H*$_3$)$_2$)); δ_C (75 MHz, DMSO-d_6) 172.1 (*C*ONHNH) 163.1 (*C*ONH), 148.6 (*ipso*-Ar-*C*), 136.9 (ipso-Ar-*C*), 130.4 (Ar-*C*), 130.1 (*ipso*-Ar-*C*), 129.0 (Ar-*C*), 128.4 (Ar-*C*), 126.7 (Ar-*C*), 121.5 (Ar-*C*), 116.4 (Ar-*C*), 115.0 (Ar-*C*), 108.3 (*ipso*-Ar-*C*), 51.9 (CONH*C*H(CH$_2$CH(CH$_3$)$_2$)), 24.8 ((CONHCH(*C*H$_2$CH(CH$_3$)$_2$)), 23.2 ((CONHCH(CH$_2$*C*H(CH$_3$)$_2$)), 22.0 ((CONH*C*H(CH$_2$CH(*C*H$_3$)$_2$)); *m/z* (ES$^+$) 494 ([^{79}Br]MH$^+$) 496 ([^{81}Br]MH$^+$); HRMS (ES$^+$) found [^{79}Br] MH$^+$, 494.0652 ($C_{19}H_{21}{}^{79}BrN_5O_6$ requires 494.0675).

(S)-*N*-(4-methyl-1-oxo-1-(2-(3-(trifluoromethyl)phenyl)hydrazineyl)pentan-2-yl)-3,5-dinitrobenzamide, 29

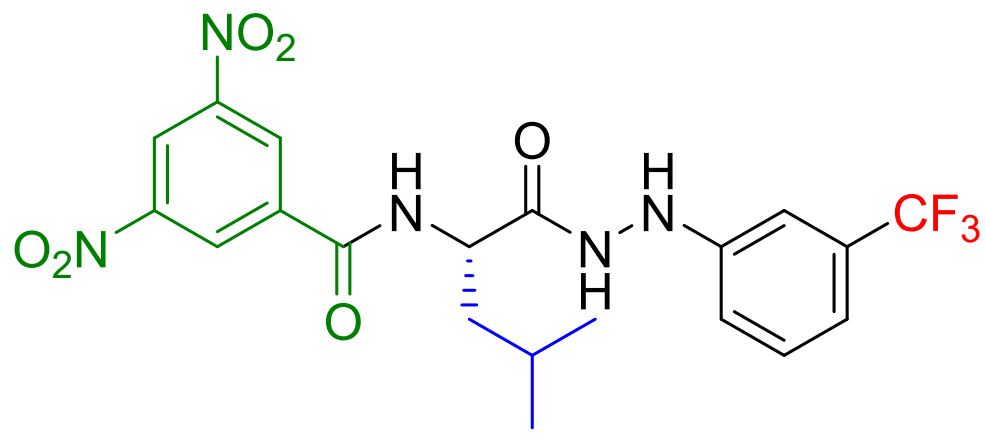

Following the general standard procedure, *tert*-butyl (S)-(4-methyl-1-oxo-1-(2-(3-(trifluoromethyl)phenyl)hydrazineyl)pentan-2-yl)carbamate (0.10 g, 0.26 mmol) was transformed following trituration with Et_2O produce the title compound as a white yellowish solid (0.08 g, 46 %); R_f 0.43 (*n*-hexane/EtOAc) [1:2]; m.p. 90 - 92 °C; ν_{max} 3247, 1665, 1537, 1470, 1340, 1320, 1082, 916 cm^{-1}; δ_H (300 MHz, DMSO-d$_6$) 10.14 (1H, d, *J* 2., CONHN*H*), 9.42 (1H, d, *J* 7, CON*H*), 9.16 (2H, d, *J* 2, Ar-*H*), 8.98 (1H, t, *J* 2, Ar-*H*), 8.11 (1H, d, *J* 2, CON*H*NH), 7.13 (1H, t, *J* 8, Ar-*H*), 6.74 (1H, t, *J* 2, Ar-*H*), 6.72 – 6.63 (2H, m, Ar-*H*), 4.69 – 4.56 (1H, m, CONHC*H*(CH$_2$CHCH$_3$)$_2$)), 1.85 – 1.63 (3H, m, CONHCH(C*H*$_2$C*H*CH$_3$)$_2$)), 0.96 (6H, dd, *J* 18, 6, CONHCH(CH$_2$CHC*H*$_3$)$_2$)); δ_C (75 MHz, DMSO-d$_6$) 172.0 (*C*ONHNH) 163.2 (*C*ONH), 151.3 (ipso-Ar-*C*)148.6 (ipso-Ar-*C*), 137.0 (ipso-Ar-*C*), 133.0 (Ar-*C*), 130.7 (*ipso*-Ar-*C*), 128. 4 (Ar-*C*), 121.5 (Ar-*C*), 118.3 (Ar-*C*), 111.9 (Ar-*C*), 111.30 (*ipso*-Ar-*C*), 51.8 (CONH*C*H(CH$_2$CH(CH$_3$)$_2$)), 24.8 (CONHCH(*C*H$_2$CH(CH$_3$)$_2$)), 23.3 (CONHCH(CH$_2$*C*H(CH$_3$)$_2$)), 22.0 (CONH*C*H(CH$_2$CH(*C*H$_3$)$_2$)); *m/z* (ES$^+$) 506 (M$^+$Na$^+$) 506; HRMS (ES$^+$) found MNa$^+$, 506.1256 ($C_{18}H_{19}{}^{79}BrN_5O_6S$ requires 506.1258).

(S)-*N*-(1-(2-(4-chlorophenyl)hydrazineyl)-4-methyl-1-oxopentan-2-yl)-3,5-dinitrobenzamide, 30

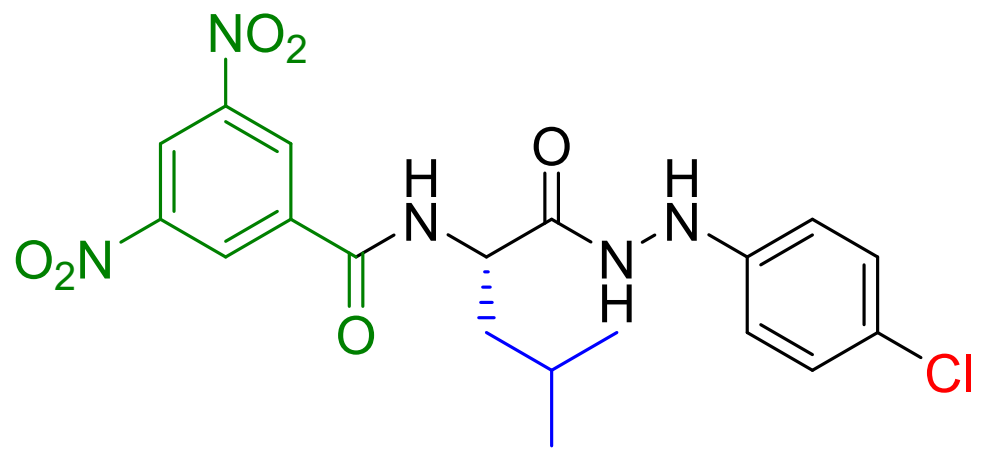

Following the general standard procedure, *tert*-butyl (S)-(1-(2-(4-chlorophenyl)hydrazineyl)-4-methyl-1-oxopentan-2-yl)carbamate (0.09 g, 0.23 mmol) was transformed following trituration with Et_2O to afforded the title compound as a faint yellow solid (0.07 g, 62 %); R_f 0.34 (DCM/EtOH) [200:6]; m.p. 90 - 92 °C; ν_{max} 3081, 1639 1530, 1490, 1320, 1087, 916, 818 cm^{-1}; δ_H (300 MHz, DMSO-d$_6$) 10.17 (1H, d, *J* 3, CON*H*NH), 9.46 (1H, d, *J* 8, CON*H*), 9.21 (2H, d, *J* 2, Ar-*H*), 9.05 (1H, t, *J* 2, Ar-*H*), 8.05 (1H, d, *J* 3, Ar-*H*), 7.29 (2H, d, *J* 8, Ar-*H*), 6.89 – 6.73 (2H, d *J* 8, Ar-*H*), 4.75 – 4.62 (1H, m, CONHC*H*(CH$_2$CH(CH$_3$)$_2$)), 1.95 – 1.64 (3H, m,

CONHCH(CH_2CH(CH_3)$_2$)), 1.04 (3H, d, *J* 6, CONHCH(CH_2CH(CH_3)$_2$)), 0.98 (3H, d, *J* 6.0, CONHCH(CH_2CH(CH_3)$_2$)); δC (75 MHz, DMSO-d_6) 172 (*C*ONHNH), 163.1 (*C*ONH), 148.7 (*ipso*-Ar-*H*), 148.6 (*ipso*-Ar-*H)*, 137.2 (*ipso*-Ar-*H)*, 128.9 (Ar-*H*), 128.4 (Ar-*H*), 122.2 (*ipso*-Ar-*H*), 121.5 (Ar-*H*), 114.1 (Ar-*H*), 51.7 (CONH*C*H(CH_2CH(CH_3)$_2$)), 24.8 (CONHCH(*C*H_2CH(CH_3)$_2$)), 23.4 (CONHCH(CH_2*C*H(CH_3)$_2$)), 21.9 (CONHCH(CH_2CH(*C*H_3)$_2$)); *m/z* (ES^+) 450 ([^{35} Cl]MH^+) 452 ([^{37} Cl]MH^+); HRMS (ES^+) found [^{35} Cl] MH^+, 450.1165 ($C_{19}H_{21}{}^{35}ClN_5O_6$ requires 450.1180).

(S)-N-(1-(2-(4-bromophenyl)hydrazineyl)-4-methyl-1-oxopentan-2-yl)-3,5-dinitrobenzamide, 31

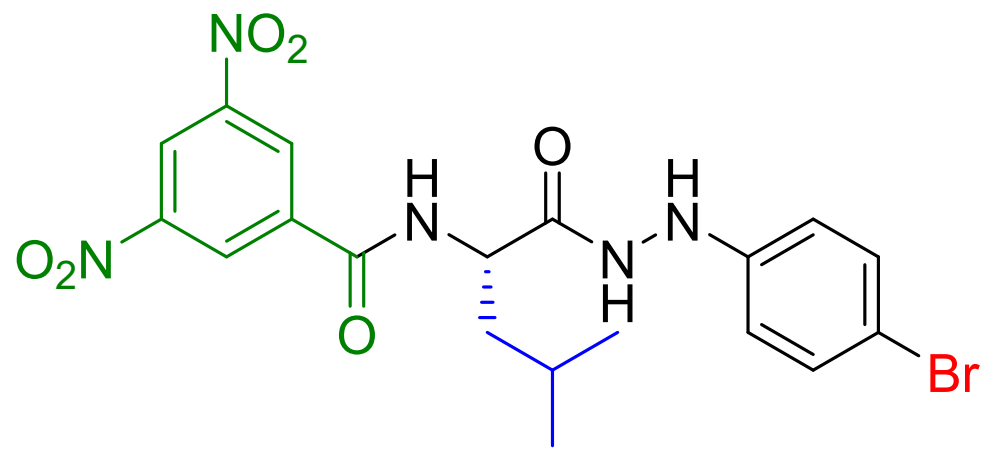

Following the general standard procedure, *tert*-butyl (S)-(1-(2-(4-bromophenyl)hydrazineyl)-4-methyl-1-oxopentan-2-yl)carbamate (0.09 g, 0.23 mmol) was transformed following trituration with Et_2O to produce the title compound as a pale yellow solid (0.07 g, 62 %); R_f 0.34 (DCM/EtOH) [200:6]; m.p. 90 - 92 °C; v_{max} 3266, 1643, 1533, 1354, 1170, 1095, 765, 646 cm^{-1}; δ_H (300 MHz, DMSO-d_6) 10.17 (1H, d, *J* 3, CON*H*NH), 9.46 (1H, d, *J* 8, CON*H*), 9.21 (2H, d, *J* 2, Ar-*H*), 9.05 (1H, t, *J* 2, Ar-*H*), 8.05 (1H, d, *J* 3, Ar-*H*), 7.29 (2H, d, *J* 8, Ar-*H*), 6.89 – 6.73 (2H, d *J* 8, Ar-*H*), 4.75 – 4.62 (1H, m, CONHC*H*(CH_2CH(CH_3)$_2$)), 1.95 – 1.64 (3H, m, CONHCH(CH_2CH(CH_3)$_2$)), 1.04 (3H, d, *J* 6, CONHCH(CH_2CH(CH_3)$_2$)), 0.98 (3H, d, *J* 6.0, CONHCH(CH_2CH(CH_3)$_2$)); δ_C (75 MHz, DMSO-d_6) 172 (*C*ONHNH), 163.1 (*C*ONH), 148.7 (*ipso*-Ar-*H*), 148.6 (*ipso*-Ar-*H)*, 137.2 (*ipso*-Ar-*H)*, 128.9 (Ar-*H*), 128.4 (Ar-*H*), 122.2 (*ipso*-Ar-*H*), 121.5 (Ar-*H*), 114.1 (Ar-*H*), 51.7 (CONH*C*H(CH_2CH(CH_3)$_2$)), 24.8 (CONHCH(*C*H_2CH(CH_3)$_2$)), 23.4 (CONHCH(CH_2*C*H(CH_3)$_2$)), 21.9 (CONHCH(CH_2CH(*C*H_3)$_2$)); *m/z* (ES^+) 494 ([^{79} Br]MH^+), 496 ([^{81} Br]MH^+); HRMS (ES^+) found [^{79} Br] MH^+, 494.0652 ($C_{19}H_{21}{}^{79}BrN_5O_6$ requires 494.0675).

(S)-*N*-(4-methyl-1-oxo-1-(2-(4-(trifluoromethyl)phenyl)hydrazineyl)pentan-2-yl)-3,5-dinitrobenzamide, 32

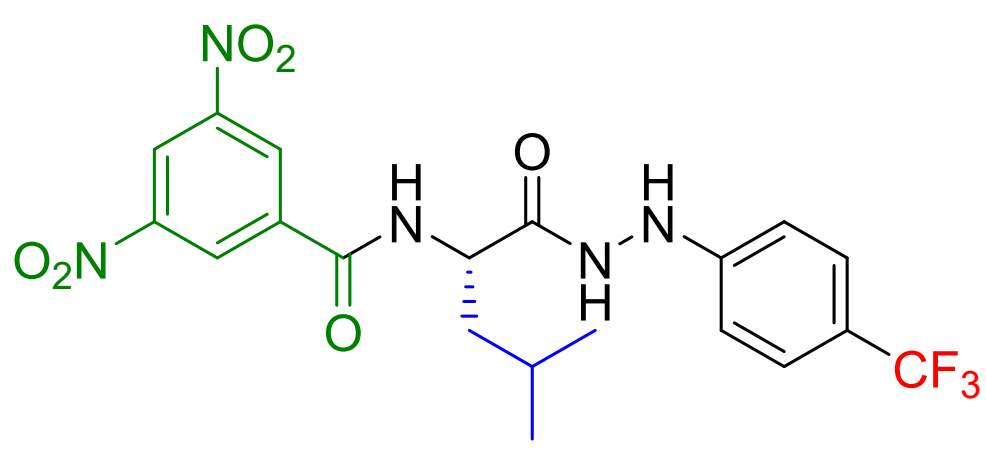

Following the general standard procedure, *tert*-butyl (S)-(4-methyl-1-oxo-1-(2-(3-(trifluoromethyl)phenyl)hydrazineyl)pentan-2-yl)carbamate (0.10 g, 0.26 mmol) was transformed following trituration with Et_2O produce the title compound as a white yellowish solid (0.08 g, 47 %); R_f 0.56 (DCM/EtOH) [200:6]; m.p. 93 - 94 °C; v_{max} 1640, 1536, 1486, 1342, 1282, 749, 719, 699, 510 cm^{-1}; δ_H (300 MHz, DMSO-d_6) 10.23 (1H, s, CON*H*NH), 9.43 (1H, d, *J* 8, CON*H*), 9.17 (2H, d, *J* 2,Ar-*H*), 8.99 (1H, t, *J* 2.,Ar-*H*), 8.46 (1H, s, CONHN*H*), 7.47 (2H, d, *J* 9, Ar-*H*), 6.83 (2H, d, *J* 9, Ar-*H*), 4.67 (1H, m, CONHC*H*($CH_2CHCH_3)_2$)), 1.90 – 1.62 (3H, m, CONHC*H*(C*H*$_2$C*H*$CH_3)_2$)), 0.97 (6H, dd, *J* 16, 6, CONHCH($CH_2CHCH_3)_2$)); δC (75 MHz, DMSO-d_6) 172.1 (*C*ONHNH), 163.1 (*C*ONH), 152.8 (*ipso*-Ar-*C*), 148.6 (*ipso*-Ar-*C*), 137.0 (*ipso*-Ar-*C*), 128.4 (Ar-*C*), 127.2 (Ar-*C*), 126.6 (Ar-*C*), 121.4 (Ar-*C*), 118.8 (Ar-*C*), 118.5 (Ar-*C*), 111.9 (Ar-*C*), 51.8 (CONH*C*H($CH_2CH(CH_3)_2$)), 24.8 (CONHCH(*C*$H_2CH(CH_3)_2$)), 23.4 CONHCH(CH_2*C*H($CH_3)_2$)), 21.83 CONHCH(*C*H_2CH(*C*$H_3)_2$)); *m/z* (ES^+) 484 (MH^+); HRMS (ES^+) found MH^+, 848.1423 ($C_{20}H_{21}F_3N_5O_6$ requires 848.1444).

(S)-*N*-(1-(2-(2-bromophenyl)hydrazineyl)-1-oxo-3-phenylpropan-2-yl)-3,5-dinitrobenzamide, 33

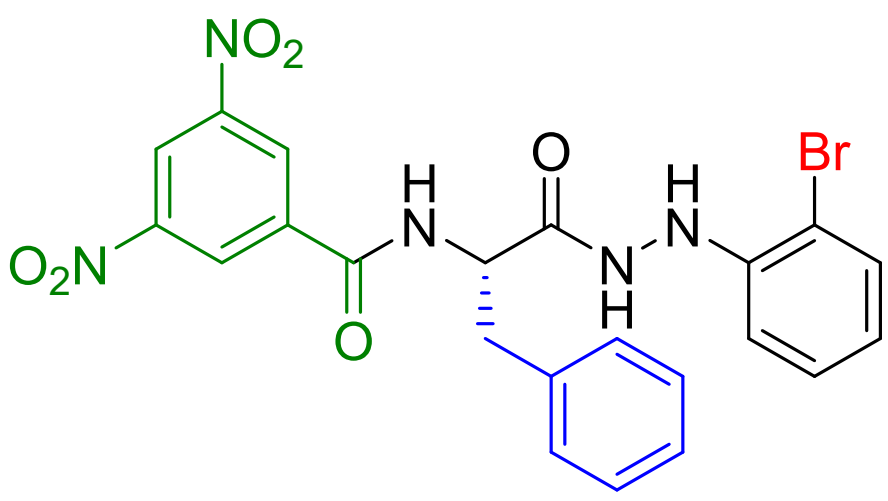

Following the general standard procedure, *tert*-butyl (S)-(1-(2-(2-bromophenyl)hydrazineyl)-1-oxo-3-phenylpropan-2-yl)carbamate (0.10 g, 0.23 mmol) was transformed following trituration with DCM to produce the title compound as a white yellowish solid (0.05 g, 44 %); R_f 0.56 (DCM/EtOH) [200: 6]; m.p. 93 - 94 °C; v_{max} 1640, 1536, 1486, 1342, 1282, 749, 719, 699, 510 cm^{-1}; δ_H (500 MHz, DMSO-d_6) 10.30 (1H, d, *J* 2, CON*H*NH), 9.60 (1H, d, *J* 8, CON*H*), 9.04 (3H, d, *J* 2, Ar-H), 8.94 (1H, t, *J* 2, Ar-*H*), 7.49 – 7.34 (6H, m, Ar-*H*), 7.27 (5H, dd, *J* 10, 8, Ar-*H*), 7.22 – 7.07 (3H, m, Ar-*H*), 6.73 – 6.62 (1H, m, Ar-*H*), 6.53 (2H, dd, *J* 8, 2, Ar-*H*), 4.99 – 4.85 (1H, m, CONHC*H*(CH_2Phe)), 3.25 (1H, dd, *J* 12, 5, CONHCH(C*H*$_2$Phe)), 3.10 (1H, dd, *J* 13,10, CONHCH(C*H*$_2$Phe)); δ_C (125 MHz, DMSO-d_6) 171 (*C*ONHNH), 163 (*C*ONH), 148.6 (*ipso*-Ar-*C*), 145.8 (*ipso*-Ar-*C*), 138.1 (*ipso*-Ar-*C*), 136.9 (*ipso*-Ar-*C*), 132.8 (*ipso*-Ar-*C*), 129.7 (Ar-*C*), 128.8 (Ar-*C*), 128.8 (Ar-*C*), 128.2 (Ar-*C*), 127.0 (Ar-*C*), 121.5 (Ar-C), 120.7 (Ar-*C*), 113.6 (Ar-*C*), 107.4 (Ar-*C*), 54.5 (CONH*C*H(CH_2Phe)), 37.4 CONH*C*H(*C*H_2Phe)); *m/z* (ES^+) *m/z* (ES^+) 528 ([^{79}Br]MH^+) 528 ([^{81}Br]MH^+); HRMS (ES^+) found [^{79}Br] MH^+, 528.0504, ($C_{22}H_{19}{}^{81}BrN_5O_6$ requires 528.0519).

(S)-3,5-dinitro-*N*-(1-oxo-3-phenyl-1-(2-(2-(trifluoromethyl)phenyl)hydrazineyl)propan-2-yl)benzamide, 34

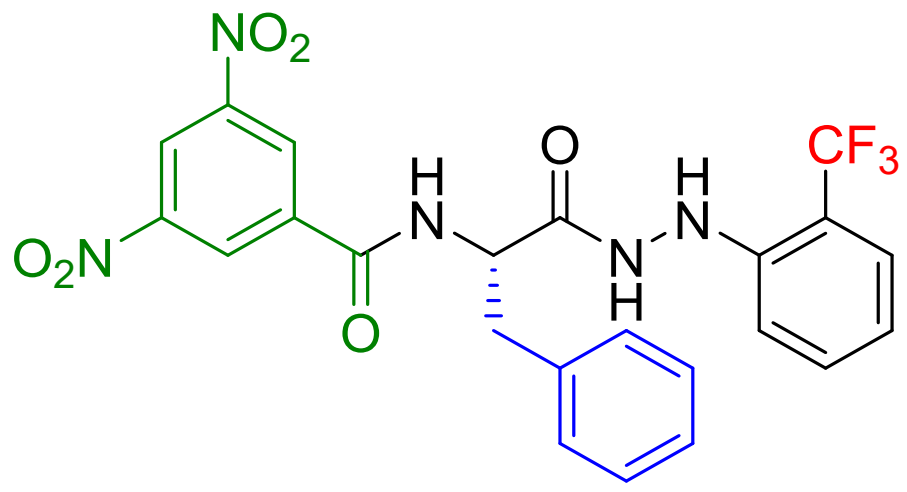

Following the general standard procedure, *tert*-butyl (S)-(1-oxo-3-phenyl-1-(2-(2-(trifluoromethyl)phenyl)hydrazineyl)propan-2-yl)carbamate (0.10 g, 0.26 mmol) was transformed following trituration with Et_2O to produce the title compound as a light orange solid (0.03 g, 45 %); R_f 0.56 (DCM/EtOH) [2:0.6]; m.p. 93 - 94 °C; ν_{max} 3260, 1629, 1540, 1343, 1181, 1034, 916 cm^{-1}; δ_H (300 MHz, DMSO-d_6) 10.33 (1H, d, *J* 2, CON*H*NH), 9.62 (1H, d, *J* 8, CON*H*), 9.05 (2H, d, *J* 2, Ar-*H*), 8.95 (1H, q, *J* 3, CONHN*H*), 7.56 (1H, s, Ar-*H*), 7.51 – 7.45 (1H, m, Ar-*H*), 7.44 – 7.21 (7H, m, Ar-*H*), 6.85 (1H, t, *J* 8, Ar-*H*), 6.69 (1H, d, *J* 8, Ar-*H*), 4.93 (1H, m, CONHC*H*(CH_2Ph)), 3.31 – 3.07 (2H, m, CONHCH(CH_2Ph)); δ_C (75 MHz, DMSO-d_6) 171.0 (*C*ONHNH), 163.0 (*C*ONH), 148.6 (*ipso*-Ar-*C*), 146.3 (*ipso*-Ar-*C)*, 138.1 (*ipso*-Ar-*C*), 136.9 (*ipso*-Ar-*C*), 133.9 (*ipso*-Ar-*C*), 129.7 (*ipso*-Ar-*C*), 128.8 (Ar-*C*), 128.2 (Ar-*C*), 127.0 (Ar-*C*), 121.5 (Ar-*C*), 118.7 (Ar-*C*), 113.4 (Ar-*C*), 112.4 (*ipso*-Ar-*C*), 54.6 (CONH*C*H(CH_2 Phe)), 37.4 (CONHCH(*C*H_2 Phe)); *m/z* (ES^+) 518 (MH^+); HRMS (ES^+) found MH^+, 518.1290 ($C_{23}H_{19}F_3N_5O_6$ requires 518.1278).

(S)-*N*-(1-(2-(3-chlorophenyl)hydrazineyl)-1-oxo-3-phenylpropan-2-yl)-3,5-dinitrobenzamide, 35

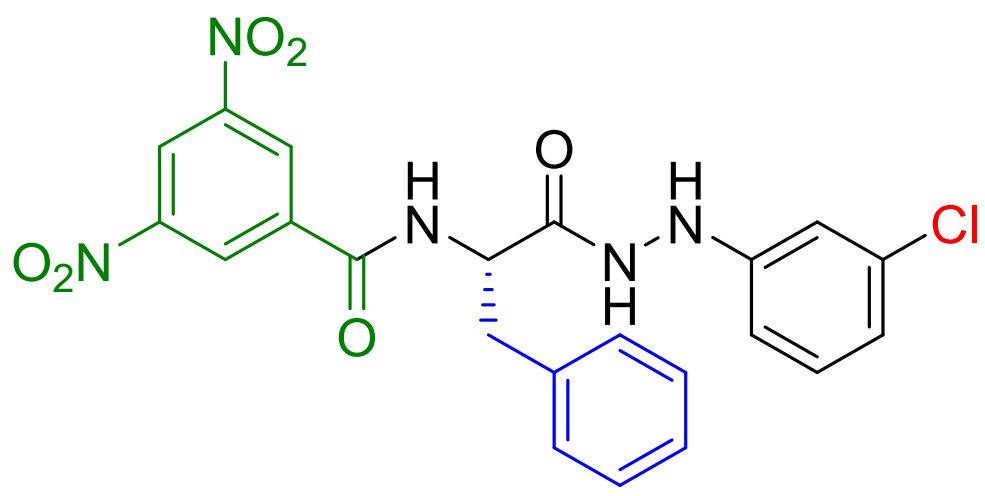

Following the general standard procedure, *tert*-butyl (S)-(1-(2-(3-chlorophenyl)hydrazineyl)-1-oxo-3-phenylpropan-2-yl)carbamate (0.12 g, 0.31 mmol) was transformed following flash column chromatography (DCM;EtOAc [3:1]) to produce the title compound as a yellow solid (0.08 g, 54 %); R_f 0.50 (DCM/EtOH/NH_3 [200:6:1]); m.p. 110 - 112 °C; ν_{max} 3227, 1672, 1641, 1538, 1341, 1076, 767, 699, 460 cm^{-1}; δ_H (300 MHz, DMSO-d_6) 10.16 (1H, s, CON*H*NH), 9.61 (1H, d, *J* 8, CON*H*), 9.06 (2H, d, *J* 2, Ar-*H*), 8.96 (1H, d, *J* 2, Ar-*H*), 7.31 (6H, m, Ar-*H*), 7.11 (1H, t, *J* 8, Ar-*H*), 6.74 – 6.66 (2H, m, Ar-*H*), 6.55 (1H, m, Ar-*H*), 4.93 – 4.82 (1H, m,

CONHC*H*(CH_2Phe)), 3.23 – 3.11 (2H, m, CONHCH(C*H*$_2$Phe)); δ_C (75 MHz, DMSO-d_6) 171.1 (*C*ONHNH), 163.1 (*C*ONH), 151.1 (*ipso*-Ar-*C*), 148.6 (*ipso*-Ar-*C*), 138.0 (*ipso*-Ar-*C*), 136.9 (*ipso*-Ar-*C*), 133.9 (*ipso*-Ar-*C*), 130.8 (Ar-*C*), 129.6 (Ar-*C*), 128.8 (Ar-*C*), 128.2 (Ar-*C*), 127.0 (*ipso*-Ar-*C*), 121.5 (Ar-C), 118.4 (Ar-*C*), 111.9 (Ar-*C*), 111.2 (Ar-*C*), 54.7 (CONH*C*H(CH_2Phe)), 37.3 (CONHCH(*C*H$_2$Phe)); *m/z* (ES^+) 484 ([^{35} Cl]MH^+), 486 ([^{37} Cl]MH^+); HRMS (ES^+) found ([^{35}Cl]MH^+) MH^+, 484.1027($C_{22}H_{20}{}^{35}ClN_5O_6$ requires 484.1024).

(S)-*N*-(1-(2-(3-bromophenyl)hydrazineyl)-1-oxo-3-phenylpropan-2-yl)-3,5-dinitrobenzamide, 36

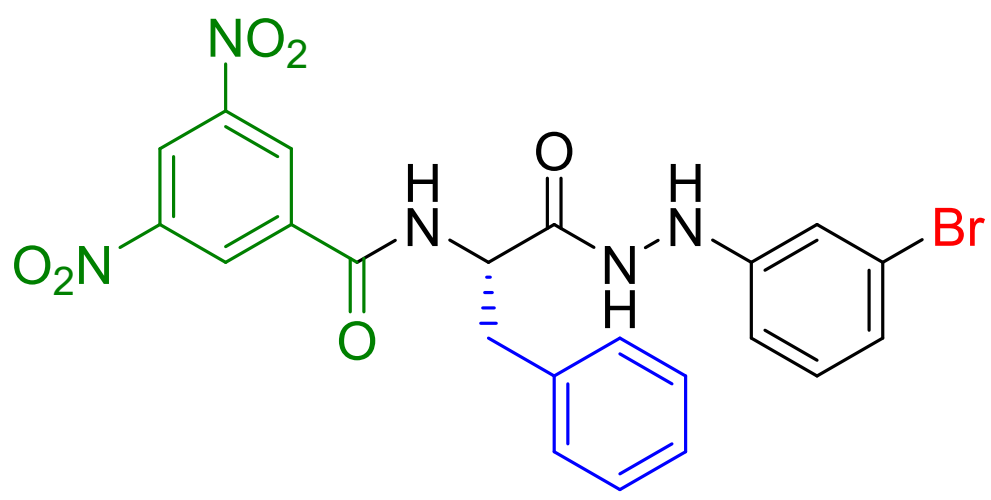

Following the general standard procedure, *tert*-butyl (S)-(1-(2-(3-bromophenyl)hydrazineyl)-1-oxo-3-phenylpropan-2-yl)carbamate (0.11 g, 0.25 mmol) was transformed following flash column chromatography (DCM;EtOH:NH_3 [200:6:1]) to produce the title compound as a white yellowish solid (0.04 g, 31 %); R_f 0.63 (DCM/EtOH/NH_3 [200:6:1]); m.p. 107-109 °C; v_{max} 3264, 1728, 1627, 1537, 1472, 1341, 1155, 1077, 921, 765 cm^{-1}; δ_H (300 MHz, DMSO-d_6) 10.16 (1H, s, CON*H*NH), 9.61 (1H, d, *J* 8, CON*H*), 9.07 (2H, d, *J* 2, Ar-*H*), 8.95 (H, t, *J* 2, CONHN*H*), 8.11 (1H, s, Ar-*H*), 7.30 (7H, m, Ar-*H*), 7.04 (1H, t, *J* 8, Ar-*H*), 6.83 (2H, d, *J* 8, Ar-*H*), 6.58 (1H, d, *J* 7,Ar-*H*), 4.94 – 4.80 (1H, m, CONHC*H*(CH_2Phe)), 3.29 – 3.04 (2H, m, CONHCH(C*H*$_2$Phe)); δ_C (75 MHz, DMSO-d_6) 171.1 (*C*ONHNH), 163.0 (*C*ONH), 151.2 (*ipso*-Ar-*C*), 148.6 (*ipso*-Ar-*C*), 138.0 (*ipso*-Ar-*C*), 136.9 (*ipso*-Ar-*C*), 131.1 (Ar-*C*), 129.6 (Ar-C), 128.8 (Ar-*C*), 128.2 (Ar-*C*), 127.0 (Ar-C), 122.5 (*ipso*-Ar-*C*), 121.5 (*ipso*-Ar-*C*), 121.3 (Ar-*C*), 114.8 (Ar-*C*), 111.6 (Ar-*C*), 54.7 (CONH*C*H(CH_2Phe)), 37.3 (CONHCH(*C*H$_2$Phe)); *m/z* (ES^+) 528 ([^{79}Br]MH^+) 530 ([^{81}Br]MH^+); HRMS (ES^+) found [^{79}Br] MH^+, 528.0522, ($C_{22}H_{19}{}^{81}BrN_5O_6$ requires 528.0522).

(S)-3,5-dinitro-*N*-(1-oxo-3-phenyl-1-(2-(3-(trifluoromethyl)phenyl)hydrazineyl)propan-2-yl)benzamide, 37

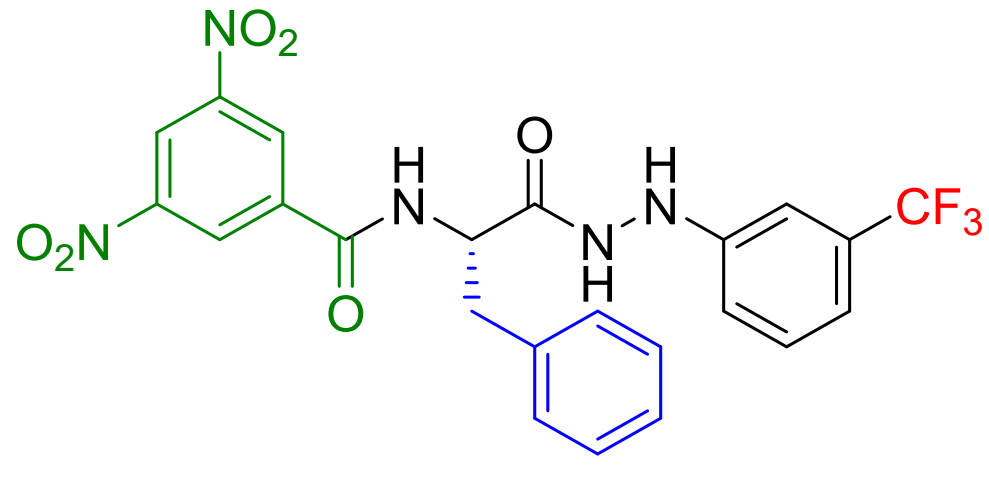

Following the general standard procedure, *tert*-butyl (S)-(1-oxo-3-phenyl-1-(2-(3-(trifluoromethyl)phenyl)hydrazineyl)propan-2-yl)carbamate (0.10 g, 0.24 mmol) was transformed following flash column chromatography (DCM;EtOH:NH_3 [200:6:1]) to produce the title compound as white yellowish solid (0.09 g, 72 %); R_f 0.27 (DCM/EtOH/NH_3 [200:6:1]); m.p. 102-105 °C; ν_{max} 3276, 1668, 1593, 1455, 1323, 1155, 1075, 921, 743 cm^{-1}; δ_H (300 MHz, DMSO-d_6) 10.25 (1H, d, *J* 2, CON*H*NH), 9.63 (1H, d, *J* 8, CON*H*), 9.06 (2H, d, *J* 2, Ar-*H*), 8.96 (1H, t, *J* 2, Ar-*H*), 8.28 (1H, d, *J* 2, CONHN*H*), 7.42 – 7.37 (2H, m,Ar-*H*), 7.35 – 7.19 (5H, m, Ar-*H*), 7.10 – 6.96 (3H, m, Ar-*H*), 6.84 (1H, d, *J* 8, Ar-*H*), 4.95 – 4.81 (1H, m, CONHC*H*(CH_2Ph)), 3.28 – 3.08 (2H,m, CONHCH(CH_2Ph)); δ_C (75 MHz, DMSO-d_6) 171.2 (*C*ONHNH), 163.1 (*C*ONH), 150.2 (*ipso*-Ar-*C*), 148.6 (*ipso*-Ar-*C*), 138.1 (*ipso*-Ar-*C*), 136.9 (*ipso*-Ar-*C*), 129.6 (Ar-*C*), 128.8 (Ar-*C*), 128.2 (Ar-*C*), 127.0 (Ar-*C*), 123.0 (Ar-*C*), 121.6 (*ipso*-Ar-*C*), 116.2 (Ar-*C*), 115.0 (*ipso*-Ar-*C*), 108.5 (Ar-*C*), 54.7 (CONH*C*H(CH_2Ph)), 37.3 (CONHCH(*C*H_2Ph)); δ_F (282 MHz, DMSO-d_6) -59.30 (3F, s, CF_3); *m/z* (ES^+) 518 (MH^+); HRMS (ES^+) found MH^+, 518.1290 ($C_{23}H_{19}F_3N_5O_6$ requires 518.1287).

(S)-*N*-(1-(2-(4-chlorophenyl)hydrazineyl)-1-oxo-3-phenylpropan-2-yl)-3,5-dinitrobenzamide, 38

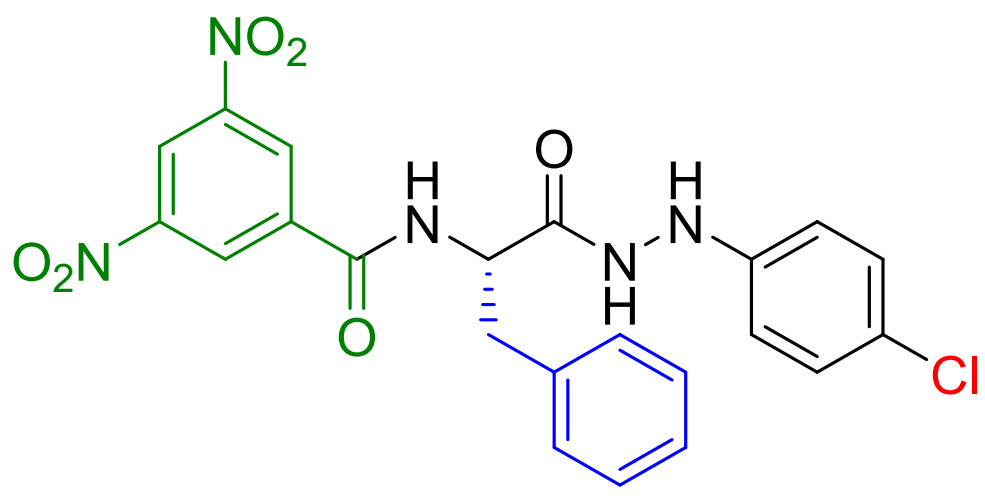

Following the general standard procedure, *tert*-butyl (S)-(1-(2-(4-chlorophenyl)hydrazineyl)-1-oxo-3-phenylpropan-2-yl)carbamate (0.05 g, 0.13 mmol) was transformed following trituration with DCM to produce the title compound as a pale yellow solid (0.04 g, 60 %); R_f 0.54 (DCM/EtOH/NH_3 [200:6:1]); m.p. 106 - 108 °C; ν_{max} 3264, 1728, 1627, 1537, 1472, 1341, 1155, 1077, 921, 765 cm^{-1}; δ_H (300 MHz, DMSO-d_6) 10.14 (1H, d, *J* 3, CON*H*NH), 9.59 (1H, d, *J* 8, CON*H*), 9.05 (2H, d, *J* 2, Ar-*H*), 8.96 (1H, t, *J* 2, Ar-*H*), 8.00 (1H, d, *J* 2, CONHN*H*), 7.43 – 7.23 (4H, m, Ar-*H*), 7.29 – 7.18 (1H, m, Ar-*H*), 7.23 – 7.06 (2H, m, Ar-*H*), 6.63 – 6.52 (2H, m, Ar-*H*), 4.96 – 4.83 (1H, m, CONHC*H*(CH_2Phe)), 3.23 (1H, dd, *J* 14, 6, CONHCH(C*H_2*Phe)), 3.11 (1H, dd, *J* 14, 10, CONHCH(C*H_2*Phe)); δ_C (75 MHz, DMSO-d_6) 171.0 (*C*ONHNH), 163.0 (*C*ONH), 148.6 (*ipso*-Ar-*C*), 148.5 (*ipso*-Ar-*C*) , 138.0 (*ipso*-Ar-*C*), 136.9 (*ipso*-Ar-*C*), 129.7 (Ar-*C*), 128.9 (Ar-*C*), 128.8 (Ar-*C*), 128.2 (Ar-*C*), 127.0 (Ar-*C*), 122.2 (*ipso*-Ar-*C*), 121.5 (Ar-*C*), 114.1 (Ar-*C*), 54.5 (CONH*C*H(CH_2Ph), 37.5 (CONHCH(*C*H_2Phe); *m/z* (ES^+) 484 ([^{35}Cl]MH^+), 486 ([^{37}Cl]MH^+); HRMS (ES^+) found ([^{35}Cl]MH^+) MH^+, 484.1027($C_{22}H_{20}{}^{35}ClN_5O_6$ requires 484.1024).

(S)-*N*-(1-(2-(4-bromophenyl)hydrazineyl)-1-oxo-3-phenylpropan-2-yl)-3,5-dinitrobenzamide, 39

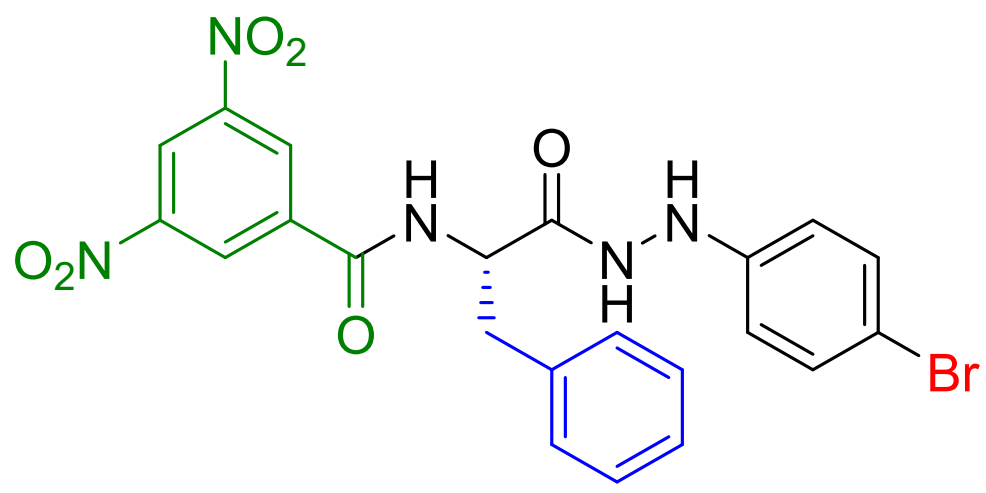

Following the general standard procedure, *tert*-butyl (S)-(1-(2-(4-bromophenyl)hydrazineyl)-1-oxo-3-phenylpropan-2-yl)carbamate (0.12 g, 0.31 mmol) was transformed following flash column chromatography (DCM:EtOAc [3:1]) to produce the title compound as a yellow solid (0.08 g, 54 %); R_f 0.50 (DCM/EtOH/NH_3 [200:6:1]); m.p. 99 - 101 °C; ν_{max} 3282, 1728, 1627, 1537, 1472, 1341, 1155, 1077, 921, 765 cm^{-1}; δ_H (300 MHz, DMSO-d_6) 10.14 (1H, d, *J* 3, CON*H*NH), 9.59 (1H, d, *J* 8, CON*H*), 9.05 (2H, d, *J* 2, Ar-*H*), 8.96 (1H, t, *J* 2, Ar-*H*), 8.00 (1H, d, *J* 2, CONHN*H*), 7.43 – 7.23 (4H, m, Ar-*H*), 7.29 – 7.18 (1H, m, Ar-*H*), 7.23 – 7.06 (2H, m, Ar-*H*), 6.63 – 6.52 (2H, m, Ar-*H*), 4.96 – 4.83 (1H, m, CONHC*H*(CH_2Phe)), 3.23 (1H, dd, *J* 14, 6, CONHCH(CH_2Phe)), 3.11 (1H, dd, *J* 14, 10, CONHCH(CH_2Phe)); δ_C (75 MHz, DMSO-d_6) 171.0 (*C*ONHNH), 163.0 (*C*ONH), 148.6 (*ipso*-Ar-C), 148.5 (*ipso*-Ar-*C*) , 138.0 (*ipso*-Ar-*C*), 136.9 (*ipso*-Ar-*C*), 129.7 (*ipso*-Ar-C), 128.9 (Ar-C), 128.8 (Ar-C), 128.2 (Ar-C), 127.0 (Ar-C), 122.2 (*ipso*-Ar-C), 121.5 (*ipso*-Ar-C), 114.1 (Ar-C), 54.5 (CONH*C*H(CH_2Ph), 37.5 (CONHCH(*C*H_2Phe); *m/z* (ES^+) 528 ([^{79}Br]MH^+) 530 ([^{81}Br]MH^+); HRMS (ES^+) found [^{79}Br] MH^+, 528.0507, ($C_{22}H_{19}{}^{81}BrN_5O_6$ requires 528.0519).

(S)-3,5-dinitro-*N*-(1-oxo-3-phenyl-1-(2-(4-(trifluoromethyl)phenyl)hydrazineyl)propan-2-yl)benzamide, 40

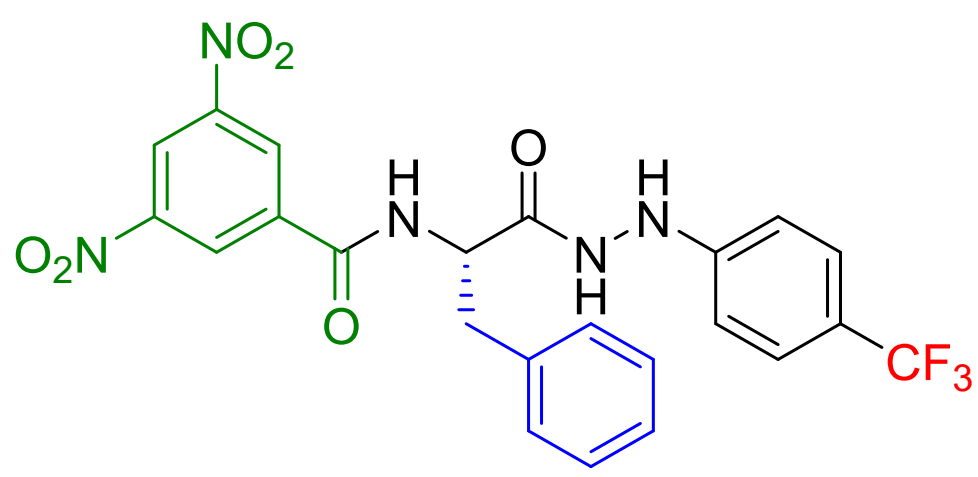

Following the general standard procedure, *tert*-butyl (S)-(1-oxo-3-phenyl-1-(2-(4-(trifluoromethyl)phenyl)hydrazineyl)propan-2-yl)carbamate (0.10 g, 0.24 mmol) was transformed following trituration with DCM to produce the title compound as a white yellowish solid (0.05 g, 43 %); R_f 0.53 (DCM/EtOH) [200:6]; m.p. 93 - 94 °C; ν_{max} 3276, 1668, 1593, 1455, 1323, 1155, 1075, 921, 743 cm^{-1}; δ_H (300 MHz, DMSO-d_6) 10.24 (1H, d, *J* 2, CON*H*NH), 9.05 (2H, d, *J* 2.1, Ar-*H*), 8.97 – 8.92 (1H, m, Ar-*H*), 8.46 (1H, d, *J* 2, CONHN*H*), 7.42 – 7.23 (7H, m,, Ar-*H*), 6.66 (2H, d, *J* 9, Ar-*H*), 4.95 – 4.85 (1H, m, CONHC*H*(CH_2Phe)), 3.26 – 3.04

(2H, m, CONHCH(CH_2Phe)). δ_C (75 MHz, DMSO-d$_6$) 170.6 (*C*ONHNH), 162.5 (*C*ONH), 152.2 (*ipso*-Ar-*C*), 148.1 (*ipso*-Ar-*C*), 137.5 (*ipso*-Ar-*C*), 136.4 (*ipso*-Ar-*C*), 129.1 (Ar-*C*), 128.3 (Ar-*C*), 127.8 (*ipso*-Ar-*CCF$_3$*), 126.5 (Ar-*C*), 126.1 (Ar-*C*), 121.1 (*ipso*-Ar-*C*), 111.4 (Ar-*C*), 54.1 (CONHCH(CH$_2$Phe)), 37.0 CONHCH(*C*H$_2$Phe); δ_F (282 MHz, DMSO-d$_6$) -59.29 (3F, s, CF$_3$); *m/z* (ES$^+$) 518 (MH$^+$); HRMS (ES$^+$) found MH$^+$, 518.1290 ($C_{23}H_{19}F_3N_5O_6$ requires 518.1287).

(S)-N-(1-(2-(2-bromophenyl)hydrazineyl)-4-(methylthio)-1-oxobutan-2-yl)-3,5-dinitrobenzamide, 41

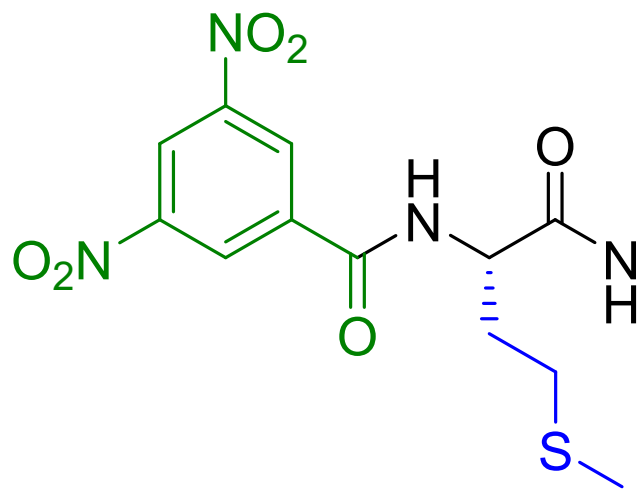

Following the general standard procedure, *tert*-butyl (S)-(1-(2-(2-bromophenyl)hydrazineyl)-4-(methylthio)-1-oxobutan-2-yl)carbamate (0.20 g, 0.48 mmol) was transformed following trituration with Et$_2$O to produce the title compound as a light orange solid (0.12 g, 49 %); R$_f$ 0.23 (DCM/EtOH) [200:3]; m.p. 125 - 126 °C; ν_{max} 3260, 1644, 1593, 1430, 1312, 1226, 1163, 1023, 915 cm^{-1}; δ_H (300 MHz, DMSO-d$_6$) 10.24 (1H, d, *J* 2, CON*H*NH), 9.44 (1H, d, *J* 7, CON*H*), 9.15 (2H, d, *J* 2, Ar-*H*), 8.98 (1H, d, *J* 2, Ar-*H*), 7.44 (1H, d, *J* 8, CONHN*H*), 7.28 (1H, s, Ar-*H*), 7.21 (1H, t, *J* 8, Ar-*H*), 6.85 – 6.81 (1H, m, Ar-*H*), 6.70 (1H, t, *J* 8, Ar-*H*), 4.69 (1H, q, *J* 8, CONHC*H*(CH$_2$CH$_2$SCH$_3$)), 2.62 (2H, m, CONHCH(CH$_2$C*H$_2$*SCH$_3$)), 2.10 (5H, m, CONHCH(C*H$_2$*CH$_2$SC*H$_3$*); d$_C$ (75 MHz, DMSO-d$_6$) 170.8 (*C*ONHNH), 162.9 (*C*ONH), 148.1 (*ipso*-Ar-*C*), 145.5 (*ipso*-Ar-*C*), 136.6 (*ipso*-Ar-*C*), 132.3 (Ar-*C*) 128.3 (Ar-*C*), 128.0 (Ar-*C*), 121.0 (Ar-*C*), 120.2 (Ar-*C*), 113.0 (Ar-C), 107.0 (*ipso*-Ar-*C*), 52.3 (CONH*C*H(CH$_2$CH$_2$SCH$_3$)), 30.8 (CONHCH(CH$_2$*C*H$_2$SCH$_3$)), 29.8 (CONHCH(*C*H$_2$CH$_2$SCH$_3$)), 14.1 (CONHCH(CH$_2$CH$_2$S*C*H$_3$)); *m/z* (ES$^+$) 512 ([^{79}Br]MH$^+$) 514 ([^{81}Br]MH$^+$); HRMS (ES$^+$) found [^{79}Br] MH$^+$ 512.0204, ($C_{18}H_{19}{}^{79}BrN_5O_6S$ requires 512.0239).

(*S*)-*N*-(4-(methylthio)-1-oxo-1-(2-(2-(trifluoromethyl)phenyl)hydrazineyl)butan-2-yl)-3,5-dinitrobenzamide, 42

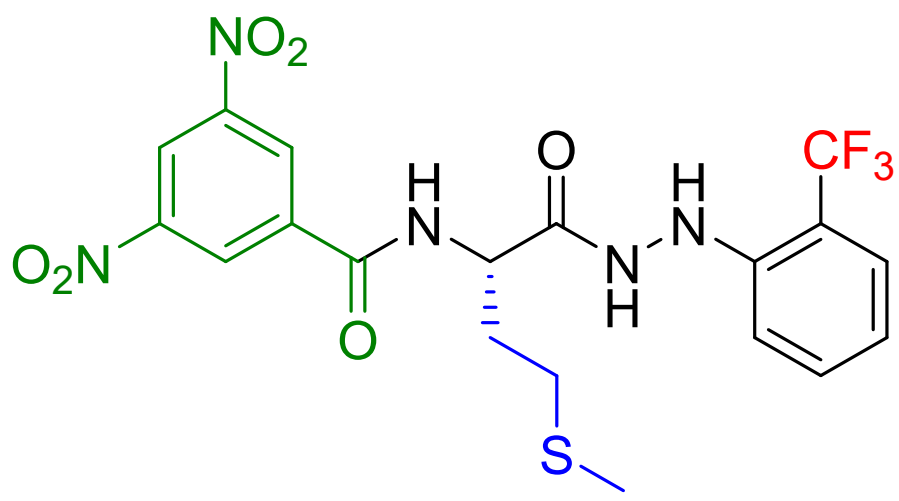

Following the general standard procedure, *tert*-butyl (S)-(4-(methylthio)-1-oxo-1-(2-(2-(trifluoromethyl)phenyl)hydrazineyl)butan-2-yl)carbamate (0.13 g, 0.31 mmol) was transformed following trituration with Et_2O to produce the title compound as a light yellow solid (0.05 g, 35 %); R_f 0.34 (DCM/EtOH) [200:3]; m.p. 128 - 130 °C; ν_{max} 3281, 1640, 1592, 1485, 1071, 914, 820, 700 cm^{-1}; δ_H (300 MHz, DMSO-d_6) 10.25 (1H, d, *J* 2., CON*H*NH), 9.45 (1H, d, *J* 7, CON*H*), 9.16 (2H, d, *J* 2. Ar-*H*), 8.99 (1H, t, *J* 2, Ar-*H*), 7.57 (1H, d, *J* 2, CONHN*H*), 7.53 – 7.40 (1H, m, Ar-*H*), 7.02 (1H, d, *J* 8, Ar-*H*), 6.87 (1H, t, *J* 8, Ar-*H*), 4.70 (1H, m, CONHC*H*$CH_2CH_2(SCH_3)$), 2.76 – 2.51 (2H, m, CONHCHC$H_2CH_2(SCH_3)$), 2.21 – 2.13 (5H, m, CONHCHCH_2CH_2(SCH_3)); δ_C (75 MHz, DMSO-d_6) 171.3 (*C*ONHNH), 163.5 (CON*H*), 148.5 (*ipso*-Ar-*C*), 146.5 (*ipso*-Ar-*C*), 137.1 (*ipso*-Ar-*C*), 133.8 (Ar-*C*), 128.5 (Ar-*C*), 126.8 (Ar-*C*), 126.0 (*ipso*-Ar-CCF_3), 121.5 (Ar-*C*), 118.6 (Ar-*C*), 113.5 (Ar-*C*), 112.4 (*ipso*-Ar-*C*), 52.8 (NH*C*H($CH_2CH_2SCH_3$)), 31.2 (NHCH(CH_2CH_2S*C*H_3)), 30.3 (CONHCH(CH_2*C*H_2SCH_3)), 15.0 (NHCH(*C*$H_2CH_2SCH_3$)); δ_F (282 MHz, DMSO-d_6) -60.75 (3F, s, CF_3); *m/z* (ES^+) 502 (MH^+); HRMS (ES^+) found MH^+, 502.1000 ($C_{19}H_{19}F_3N_5O_6S$ requires 502.1008).

(S)-*N*-(1-(2-(3-bromophenyl)hydrazineyl)-4-(methylthio)-1-oxobutan-2-yl)-3,5-dinitrobenzamide, 43

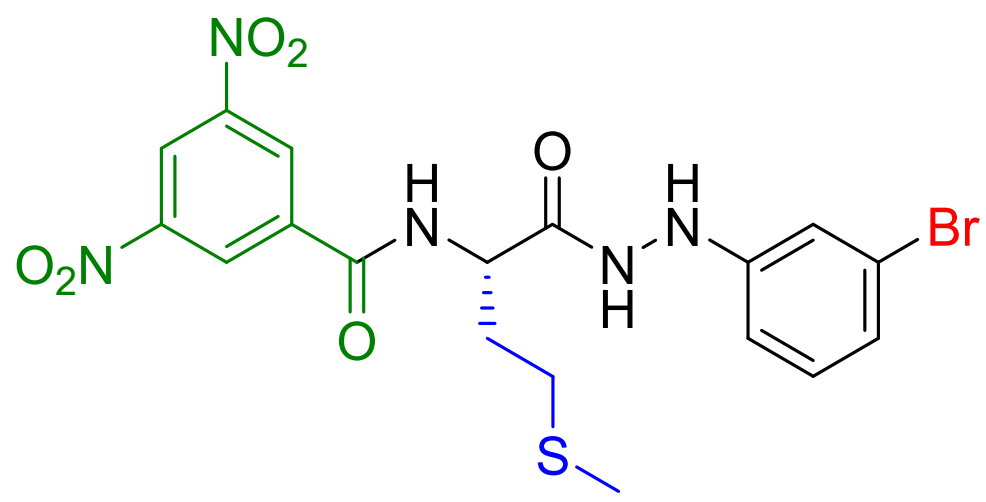

Following the general standard procedure, *tert*-butyl (S)-(1-(2-(3-bromophenyl)hydrazineyl)-4-(methylthio)-1-oxobutan-2-yl)carbamate (0.10 g, 0.25 mmol) was transformed following trituration with Et_2O to produce the title compound as a light orange solid (0.09 g, 69 %); R_f 0.32 (DCM/EtOH/NH_3) [200:3:1]; m.p. 127 - 129 °C; ν_{max} 3259, 3071, 1649, 1640, 1596, 1536, 1476, 1341, 1340, 1320, 1076, 1041, 918, 847 cm^{-1}; δ_H (300 MHz, DMSO-d_6) 10.12 (1H, s, CON*H*NH), 9.44 (1H, d, *J* 7, CONH), 9.15 (2H, d, *J* 2, Ar-*H*), 8.99 (1H, dd, *J* 2, Ar-*H*), 8.10 (1H, s, CONHN*H*), 7.15 (1H, t, *J* 8, Ar-*H*), 6.78–6.62 (3H, m, Ar-*H*), 4.68 (1H, m, CONHC*H*($CH_2CH_2SCH_3$)), 2.74–2.53 (2H, m, CONHCH(CH2CH_2SCH_3)), 2.19–2.04 (5H, m, CONHCH(CH_2CH_2SCH_3)); δC (75 MHz, DMSO-d_6) 171.8 (*C*ONHNH), 170.0 (*C*ONH), 151.4 (*ipso*-Ar-*C*), 131.1 (Ar-*C*), 128.5 (Ar-*C*), 122.5 (Ar-*C*), 121.2 (Ar-C), 114.8 (Ar-*C*), 111.6 (Ar-*C*), , 51.8 (CONHC*H*($CH_2CH_2SCH_3$)), 30.1 (CONHCH(CH_2*C*H_2SCH_3)), 23.0 (CONHCH(*C*$H_2CH_2SCH_3$)), 15.1 (CONHCH(CH_2CH_2S*C*H_3)); *m/z* (ES^+) 512 ([^{79}Br]MH^+) 514 ([^{81}Br]MH^+); HRMS (ES^+) found [^{79}Br] MH^+ 512.0204, ($C_{18}H_{19}{}^{79}BrN_5O_6S$ requires 512.0239).

(S)-*N*-(4-(methylthio)-1-oxo-1-(2-(3-(trifluoromethyl)phenyl)hydrazineyl)butan-2-yl)-3,5-dinitrobenzamide, 44

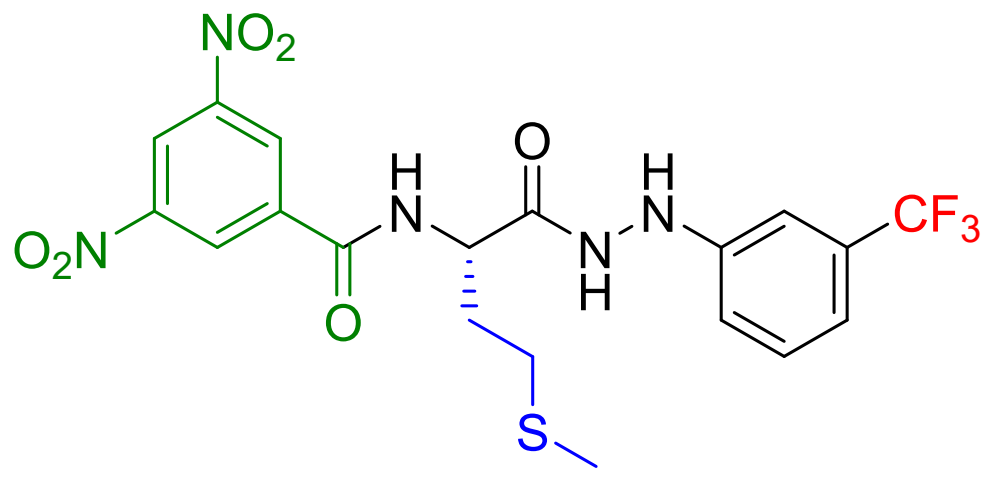

Following the general standard procedure, *tert*-butyl (S)-(4-(methylthio)-1-oxo-1-(2-(3-(trifluoromethyl)phenyl)hydrazineyl)butan-2-yl)carbamate (0.12 g, 0.28 mmol) was transformed following trituration with DCM to produce the title compound as a yellowish white solid (0.09 g, 62 %); R_f 0.45 (n-hexane/EtOAc) [4:1]; m.p. 128 - 130 °C; ν_{max} 3281, 1640, 1592, 1485, 1071, 914, 820, 700 cm^{-1}; δ_H (300 MHz, DMSO-d_6) 10.20 (1H, d, *J* 3, CONHNH), 9.46 (1H, d, *J* 7, CON*H*), 9.15 (2H, d, *J* 2, Ar-*H*), 8.99 (1H, t, *J* 2, Ar-*H*), 8.26 (1H, s, CONHN*H*), 7.36 (1H, t, *J* 8, Ar-*H*), 7.01 (3H, d, *J* 7, Ar-*H*), 4.66 (1H, q, *J* 7, CONHC*H*($CH_2CH_2SCH_3$)), 2.70 – 2.55 (2H, m, CONHCH(C$H_2$$CH_2SCH_3$)), 2.10 (5H, m, CONHCH($CH_2CH_2SCH_3$)). δ_C (75 MHz, DMSO-d_6) 171.5 (*C*ONHNH), 163.4 (*C*ONH), 150.3 (*ipso*-Ar-*C*), 148.6 (*ipso*-Ar-*C*), 136.9 (*ipso*-Ar-*C*), 130.2 (Ar-*C*), 128.4 (Ar-*C*), 121.5 (Ar-*C*), 116.3 (Ar-*C*), 52.8 (CONH*C*H($CH_2CH_2SCH_3$)), 31.1 ((CONHCH(CH_2*C*H_2SCH_3)), 30.3 (CONHCH(*C*$H_2CH_2SCH_3$)), 15.02 ((CONHCH(CH_2CH_2S*C*H_3)); δ_F (282 MHz, DMSO-d_6) -59.29 (3F, s, CF_3); *m/z* (ES^+) 502 (MH^+); HRMS (ES^+) found MH^+, 502.1000 ($C_{19}H_{19}F_3N_5O_6S$ requires 502.1008).

(S)-*N*-(1-(2-(4-chlorophenyl)hydrazineyl)-4-(methylthio)-1-oxobutan-2-yl)-3,5-dinitrobenzamide, 45

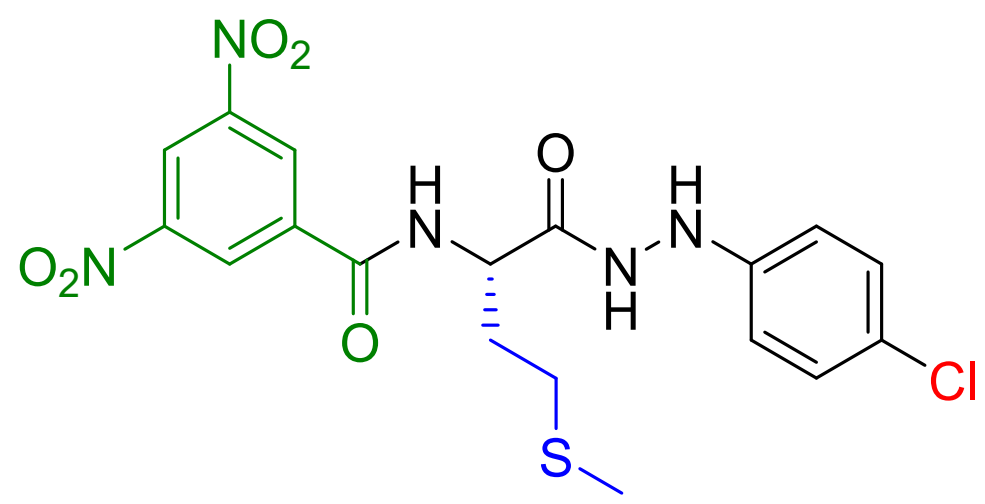

Following the general standard procedure, *tert*-butyl (S)-(1-(2-(4-chlorophenyl)hydrazineyl)-4-(methylthio)-1-oxobutan-2-yl)carbamate (0.10 g, 0.28 mmol) was transformed following flash column chromatography using DCM/EtOH [200:4] produce the title compound as a light yellow solid (0.09 g, 68 %); R_f 0.56 (DCM/EtOH/NH_3) [200:3:1]; m.p. 90 - 92°C; ν_{max} 3269, 1654, 1537, 1478, 1341, 1320, 1073, 915, 822 cm^{-1}; δ_H (300 MHz, DMSO-d_6) 10.10 (1H, d, *J* 3, CON*H*NH), 9.43 (1H, d, *J* 7, CON*H*), 9.15 (2H, d, *J* 2, Ar-*H*), 8.99 (1H, t, *J* 2, Ar-*H*), 8.00 (1H,

d, *J* 3, CONHN*H*), 7.28 (2H, d, *J* 9, Ar-*H*), 6.69 (2H, d, *J* 9, Ar-*H*), 4.72 – 4.63 (1H, m, CONHC*H*($CH_2CH_2SCH_3$)) – 2.51 (2H, m, CONHC*H*($CH_2CH_2SCH_3$)), 2.10 (5H, m, CONHC*H*($CH_2CH_2SCH_3$)). δ_C (75 MHz, DMSO-d_6) 171.3 (*C*ONHNH), 163.3 (*C*ONH), 149.1 (*ipso*-Ar-*C*), 148.5 (*ipso*-Ar-*C*), 137.1 (*ipso*-Ar-C), 131.7 (ipso-Ar-C), 128.5 (Ar-C), 121.4 (Ar-C), 114.6 (Ar-C), 109.8 (*ipso*-Ar-*C*), 52.7, (CONHCH($CH_2CH_2SCH_3$)), 31.3 ((CONHCH($CH_2CH_2SCH_3$)), 30.4 (CONHCH($CH_2CH_2SCH_3$)), 15.1 (CONHCH($CH_2CH_2SCH_3$)); *m/z* (ES$^+$) 468 ([^{35}Cl]MH$^+$) 470 ([^{37}Cl]MH$^+$); HRMS (ES$^+$) found [^{35}Cl] MH$^+$, 468.0760 ($C_{18}H_{19}{}^{35}ClN_5O_6S$ requires 468.0745).

(S)-*N*-(1-(2-(4-bromophenyl)hydrazineyl)-4-(methylthio)-1-oxobutan-2-yl)-3,5-dinitrobenzamide, 46

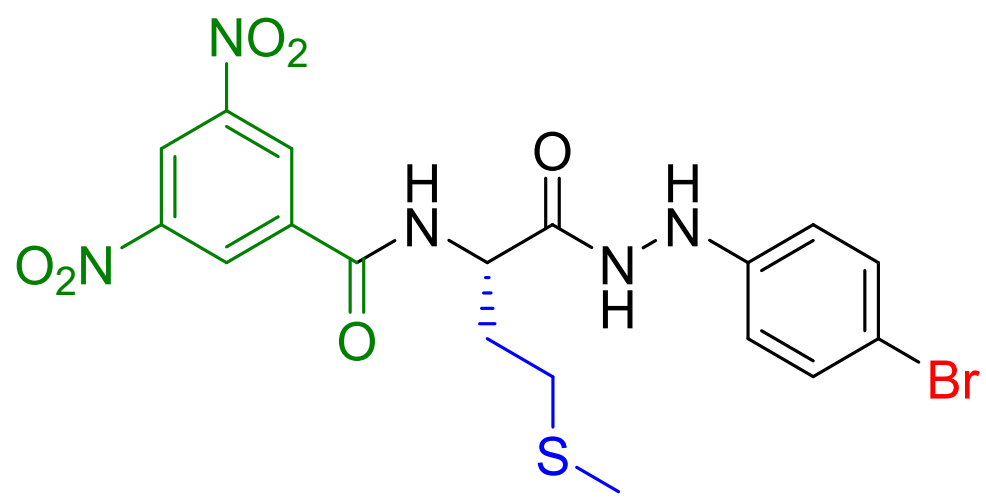

Following the general standard procedure, *tert*-butyl (S)-(1-(2-(3-bromophenyl)hydrazineyl)-4-(methylthio)-1-oxobutan-2-yl)carbamate (0.10 g, 0.24 mmol) was transformed following trituration with Et_2O to produce the title compound as a light orange solid (0.09 g, 69 %); R_f 0.32 (DCM/EtOH/NH_3) [200:3:1]; m.p. 90 - 92°C; v_{max} 3269, 1654, 1537, 1478, 1341, 1320, 1073, 915, 822 cm^{-1}; δ_H (300 MHz, DMSO-d_6) 10.10 (1H, d, *J* 3, CON*H*NH), 9.43 (1H, d, *J* 7, CON*H*), 9.15 (2H, d, *J* 2, Ar-*H*), 8.99 (1H, t, *J* 2, Ar-*H*), 8.00 (1H, d, *J* 3, CONHN*H*), 7.28 (2H, d, *J* 9, Ar-*H*), 6.69 (2H, d, *J* 9, Ar-*H*), 4.72 – 4.63 (1H, m, CONHC*H*($CH_2CH_2SCH_3$)) – 2.51 (2H,m, CONHC*H*($CH_2CH_2SCH_3$)), 2.10 (5H, m, CONHC*H*($CH_2CH_2SCH_3$)). δC (75 MHz, DMSO-d_6) 171.3 (*C*ONHNH), 163.3 (*C*ONH), 149.1 (*ipso*-Ar-*C*), 148.5 (*ipso*-Ar-*C*), 137.1 (*ipso*-Ar-C), 131.7 (*ipso*-Ar-C), 128.5 (Ar-C), 121.4 (Ar-C), 114.6 (Ar-C), 109.8 (*ipso*-Ar-*C*), 52.7, (CONHCH($CH_2CH_2SCH_3$)), 31.3 ((CONHCH($CH_2CH_2SCH_3$)), 30.4 (CONHCH($CH_2CH_2SCH_3$)), 15.1 (CONHCH($CH_2CH_2SCH_3$)), *m/z* (ES$^+$) 512 ([^{79}Br]MH$^+$) 512 ([^{81}Br]MH$^+$) 514; HRMS (ES$^+$) found [^{79}Br] MH$^+$ 512.0249, ($C_{18}H_{19}{}^{79}BrN_5O_6S$ requires 512.0239).

(S)-N-(1-(2-(2-bromophenyl)hydrazineyl)-1-oxo-4-phenylbutan-2-yl)-3,5-dinitrobenzamide, 47

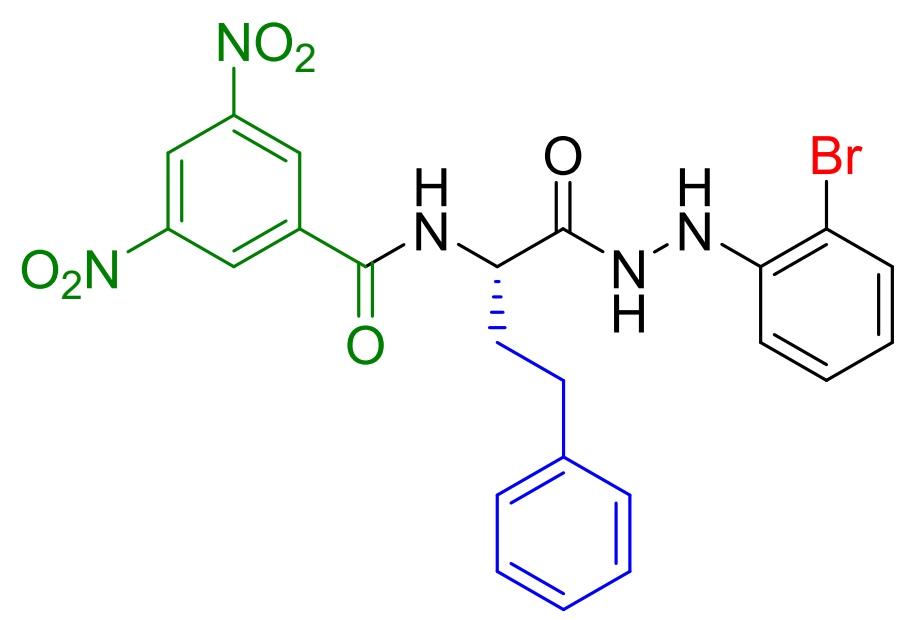

Following the general standard procedure, *tert*-butyl (S)-(1-(2-(2-bromophenyl)hydrazineyl)-1-oxo-4-phenylbutan-2-yl)carbamate (0.10 g, 0.22 mmol) was transformed following flash column chromatography (*n*-hexane/EtOAc) [3:1] to produce the title compound as a white solid (0.08 g, 63 %); R_f 0.56 (DCM/EtOH) [200: 6]; m.p. 93 - 94 °C; v_{max} 3268, 1655, 1537, 1488, 1342, 744, 649, 494 cm^{-1}; δ_H (300 MHz, DMSO-d_6) 10.24 (1H, d, *J* 2, CON*H*NH), 9.50 (1H, d, *J* 7,CON*H*), 9.16 (2H, d, *J* 2.,Ar-*H*), 9.00 (1H, t, *J* 2, Ar-*H*), 7.44 (1H, dd, *J* 8, 1, Ar-*H*), 7.35 – 7.31 (5H, m, Ar-*H*), 7.24 (1H, m, CONHN*H*) 6.83 (1H, dd, *J* 8, 2, Ar-*H*), 6.69 (1H, ddd, *J* 8, 7, 2 , Ar-*H*), 4.58 (1H, m, CONHC*H*(CH_2CH_2Phe)), 2.83 – 2.67 (2H, m, CONHCH(CH_2CH_2Phe)), 2.23 – 2.11 (2H, m, CONHCH(CH_2CH_2Phe)); δC (75 MHz, DMSO-d_6) 171.5 (*C*ONHNH), 163.4 (*C*ONH), 148.6 (*ipso*-Ar-*C*), 145.98 (*ipso*-Ar-*C*), 141.5 (*ipso*-Ar-*C*), 137.1 (*ipso*-Ar-*C*), 132.8 (Ar-*C*), 128.9 (Ar-*C*), 128.8 (*ipso*-Ar-*C*), 128.5 (Ar-*C*), 126.4 (Ar-*C*), 121.5 (Ar-*C*), 120.7 (Ar-*C*), 113.7 (Ar-*C*), 107.4 (*ipso*-Ar-*C*), 53.3 (CONH*C*H(CH_2CH_2Phe)), 33.4 (CONH*C*H(CH_2CH_2Phe)), 32.2 (CONHCH(CH_2CH_2Phe)); *m/z* (ES^+) 564 ([^{79}Br]MNa^+) 566 ([^{81}Br]MNa^+); HRMS (ES^+) found [^{79}Br] MNa^+, 564.0491 ($C_{23}H_{21}{}^{79}BrN_5O_6$ requires 564.0489).

(S)-3,5-dinitro-*N*-(1-oxo-4-phenyl-1-(2-(2-(trifluoromethyl)phenyl)hydrazineyl)butan-2-yl)benzamide, 48

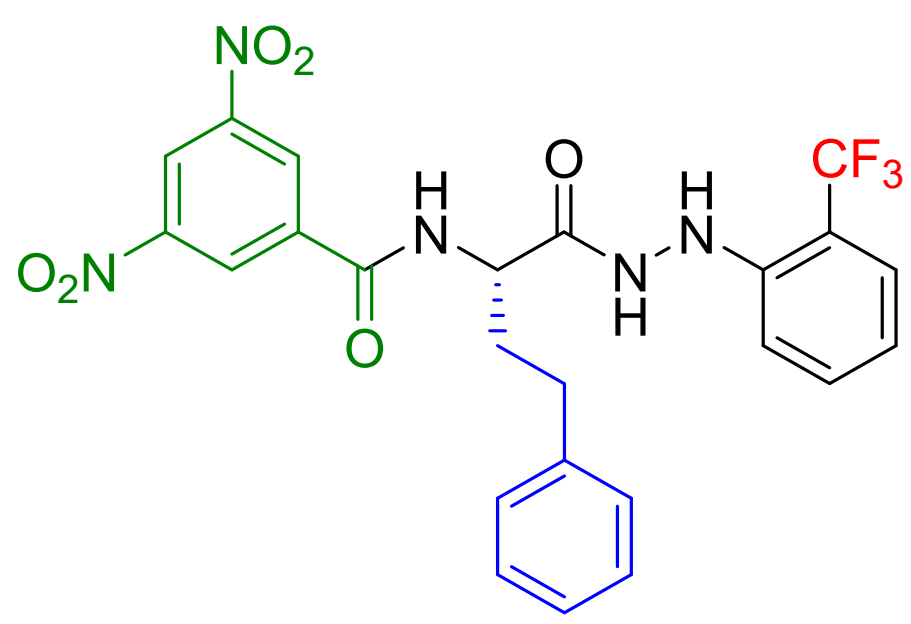

Following the general standard procedure, *tert*-butyl (S)-(1-oxo-4-phenyl-1-(2-(2-(trifluoromethyl)phenyl)hydrazineyl)butan-2-yl)carbamate (0.10 g, 0.23 mmol) was transformed following trituration with Et_2O to produce the produce the title compound as a white solid (0.08 g, 62 %); R_f 0.56 (DCM/EtOH) [200:6]; m.p. 106 - 108 °C; v_{max} 3278, 1645, 1552, 1343, 1297, 1102, 820 cm^{-1}; δ_H (300 MHz, DMSO-d_6) 10.25 (1H, d, *J* 2, CON*H*NH), 9.51 (1H, d, *J* 7, CON*H*), 9.17 (2H, d, *J* 2, Ar-*H*), 9.00 (1H, t, *J* 2, Ar-H), 7.56 (1H, s, CONHN*H*),

7.46 (2H, dd, *J* 13, 8, Ar-*H*), 7.35 – 7.17 (5H, m, Ar-*H*), 7.01 (1H, d, *J* 8, Ar-*H*), 6.86 (1H, t, *J* 8, Ar-*H*), 4.64 – 4.53 (1H, m, CONHC*H*(CH_2CH_2Phe)), 2.86 – 2.69 (1H, m, CONHCH(CH_2CH_2Phe), 2.30 – 2.12 (2H, m, CONHCH(CH_2CH_2Phe)); δ_C (75 MHz, DMSO-d_6) 171.5 (*C*ONHNH) 163.4 (*C*ONH), 148.6 (*ipso*-Ar-*C*), 146.5 (*ipso*-Ar-*C*), 141.5 (*ipso*-Ar-*C*), 137.1 (*ipso*-Ar-*C*), 133.8 (Ar-*C*), 128.8 (Ar-*C*), 128.5 (Ar-*C*), 126.4 (ipso-Ar-C*CF₃*), 121.5 (Ar-*C*), 118.6 (Ar-*C*), 113.5 (Ar-*C*), 112.4 (*ipso*-Ar-*C*), 53.4 (CONH*C*H(CH_2CH_2Phe)), 33.3 (CONHCH(CH_2CH_2Phe)), 32.2 (CONHCH(CH_2CH_2Phe)); *m/z* (ES^+) 532 (MH^+); HRMS (ES^+) found MH^+, 532.1432 ($C_{24}H_{21}F_3N_5O_6$ requires 532.1444).

(S)-*N*-(1-(2-(3-chlorophenyl)hydrazineyl)-1-oxo-4-phenylbutan-2-yl)-3,5-dinitrobenzamide, 49

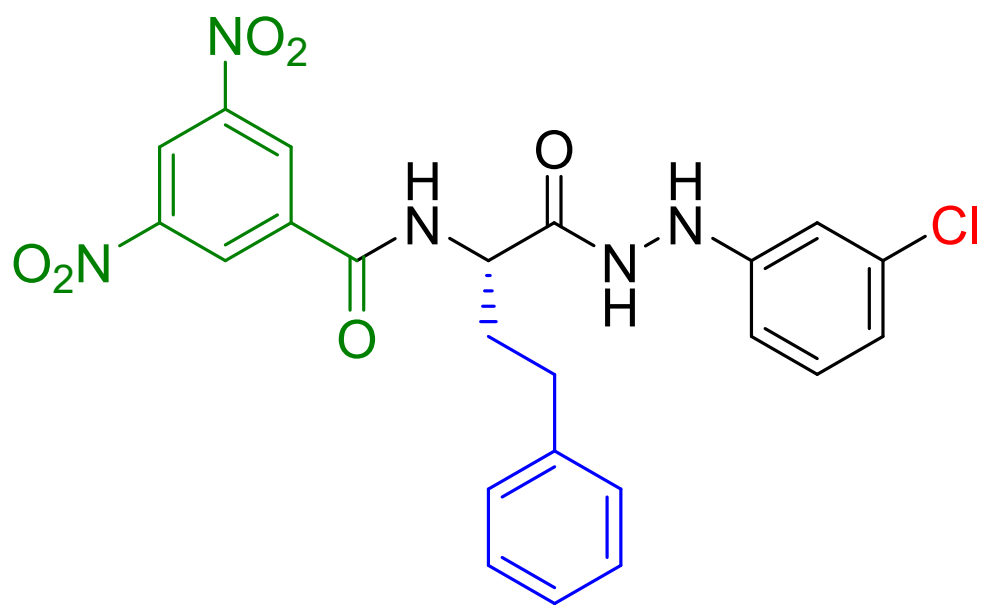

Following the general standard procedure, *tert*-butyl (S)-(1-(2-(3-chlorophenyl)hydrazineyl)-1-oxo-4-phenylbutan-2-yl)carbamate (0.10 g, 0.25 mmol) was transformed to produce the title compound as a yellow white solid (0.10 g, 83 %); R_f 0.48 (n-hexane/EtOAc) [2:1]; m.p.111 - 113 °C; v_{max} 3265, 1628, 1538, 1442, 1309, 1221, 1140, 1072, 991, 729, 570 cm^{-1}; δ_H (300 MHz, DMSO-d_6) 10.11 (1H, d, *J* 2., CON*H*NH), 9.50 (1H, d, *J* 7, CONH), 9.17 (2H, d, *J* 2, Ar-*H*), 8.99 (1H, t, *J* 2, Ar-*H*), 8.11 (1H, d, *J* 2.,CONHN*H*), 7.34 – 7.16 (6H, m, Ar-*H*), 7.07 (1H, t, *J* 8., Ar-*H*), 6.90 (1H, t, *J* 2.,Ar-*H*), 6.86 – 6.81 (2H, m, Ar-*H*), 6.76 – 6.66 (2H, m, Ar-*H*), 4.53 (1H, q, *J* 7,CONHC*H*(CH_2CH_2Phe)), 2.87 – 2.61 (2H, m, CONHCH(CH_2CH_2Phe)), 2.16 (2H, q, *J* 8, CONHCH(CH_2CH_2Phe)); δ_C (75 MHz, DMSO-d_6) 171.6 (*C*ONHNH), 163.4 (*C*ONH), 151.4 (*ipso*-Ar-*C*), 148.6 (*ipso*-Ar-*C*), 137.1 (*ipso*-Ar-*C*), 131.1 (*ipso*-Ar-*C*), 128.9 (Ar-*C*), 128.5 (Ar-*C*), 126.5 (Ar-*C*), 122.6 (Ar-*C*), 121.5 (Ar-*C*), 121.2 (Ar-*C*), 114.8 (Ar-*C*), 111.7 (Ar-*C*), 79.6 (Ar-*C*), 53.4 (CONH*C*H(CH_2CH_2Phe)) 32.8 (CONHCH(CH_2CH_2Phe)); 31.8 (CONHCH(CH_2CH_2Phe)); *m/z* (ES^+) 498 ([^{35}Cl]MH^+) 500 ([^{37}Cl]MH^+); HRMS (ES^+) found [^{35}Cl] MH^+, 498.0875 ($C_{23}H_{20}{}^{35}ClN_5O_6$ requires 498.1180).

(S)-*N*-(1-(2-(3-bromophenyl)hydrazineyl)-1-oxo-4-phenylbutan-2-yl)-3,5-dinitrobenzamide, 50

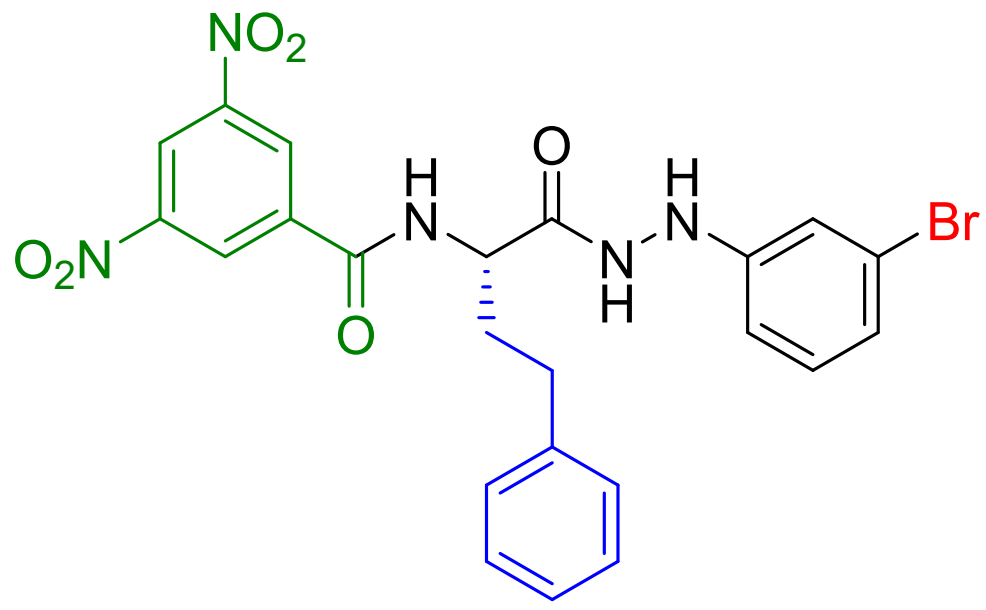

Following the general standard procedure, *tert*-butyl (S)-(1-(2-(3-bromophenyl)hydrazineyl)-1-oxo-4-phenylbutan-2-yl)carbamate (0.13 g, 0.28 mmol) was transformed following flash column chromatography (*n*-hexane/EtOAc) [3:1] to produce the title compound as a white solid (0.08 g, 44 %); R_f 0.56 (DCM/EtOH) [200:6]; m.p. 112 - 114 °C; v_{max} 3279, 1642, 1595, 1490, 1341, 1077, 915, 861, 769 cm^{-1}; δ_H (300 MHz, DMSO-d_6) 10.11 (1H, d, *J* 2., CON*H*NH), 9.50 (1H, d, *J* 7, CONH), 9.17 (2H, d, *J* 2, Ar-*H*), 8.99 (1H, t, *J* 2, Ar-*H*), 8.11 (1H, d, *J* 2.,CONHN*H*), 7.34 – 7.16 (6H, m, Ar-*H*), 7.07 (1H, t, *J* 8., Ar-*H*), 6.90 (1H, t, *J* 2.,Ar-*H*), 6.86 – 6.81 (2H, m, Ar-*H*), 6.76 – 6.66 (2H, m, Ar-*H*), 4.53 (1H, q, *J* 7,CONHC*H*(CH_2CH_2Phe)), 2.87 – 2.61 (2H, m, CONHCH($CH_2C\mathit{H_2}$Phe)), 2.16 (2H, q, *J* 8, CONHCH($C\mathit{H_2}CH_2$Phe)). δ_C (75 MHz, DMSO-d_6) 171.6 (*C*ONHNH), 163.4 (*C*ONH), 151.4 (*ipso*-Ar-*C*), 148.6 (*ipso*-Ar-*C*), 137.1 (*ipso*-Ar-*C*), 131.1 (*ipso*-Ar-*C*), 128.9 (Ar-*C*), 128.5 (Ar-*C*), 126.5 (Ar-*C*), 122.6 (Ar-*C*), 121.5 (Ar-*C*), 121.2 (Ar-*C*), 114.8 (Ar-*C*), 111.7 (Ar-*C*), 79.6 (Ar-*C*), 53.4 (CONH*C*H(CH_2CH_2Phe)), 33.3 (CONHCH($\mathit{C}H_2CH_2$Phe), 32.3 (CONHCH($CH_2\mathit{C}H_2$Phe)); *m/z* (ES^+) 542 ([^{79}Br]MH^+) 544 ([^{81}Br]MH^+), HRMS (ES^+) found [^{79}Br] MH^+, 544.0645, ($C_{23}H_{20}{}^{79}BrN_5O_6$ requires 544.0675).

(S)-3,5-dinitro-*N*-(1-oxo-4-phenyl-1-(2-(3-(trifluoromethyl)phenyl)hydrazineyl)butan-2-yl)benzamide, 51

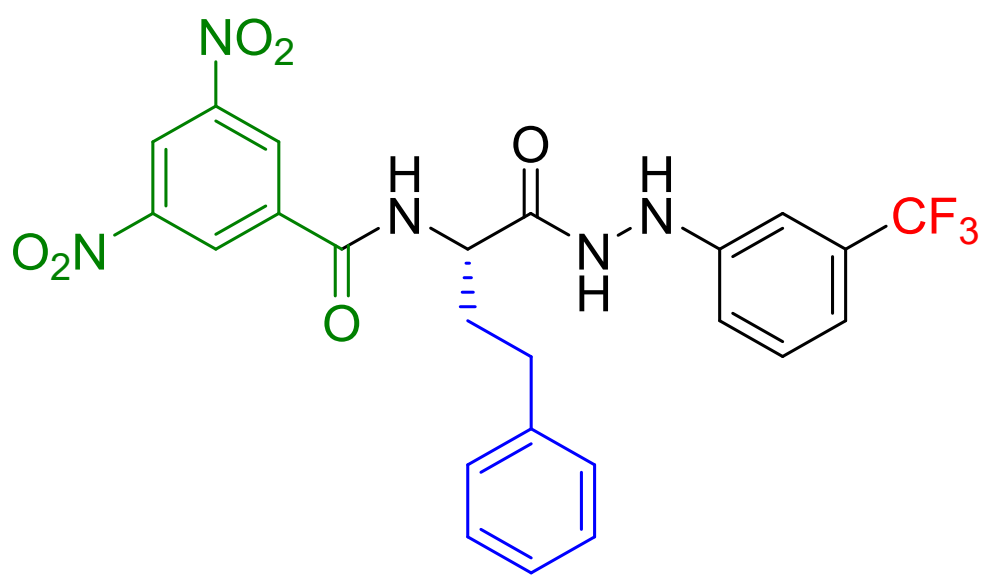

Following the general standard procedure, *tert*-butyl (S)-(1-oxo-4-phenyl-1-(2-(3-(trifluoromethyl)phenyl)hydrazineyl)butan-2-yl)carbamate (0.10 g, 0.23 mmol) was transformed following trituration with diethyl ether to produce the title compound as alight yellow solid (0.05 g, 44 %); R_f 0.45 (DCM/EtOH) [100:3]; m.p 113- 115 °C; v_{max} 3275, 1651, 1538, 1445, 1275, 1119, 1067, 918, 718 cm^{-1}; δ_H (300 MHz, DMSO-d_6) 10.20 (1H, d, *J* 2,CON*H*NH), 9.52 (1H, d, *J* 7, CON*H*), 9.17 (2H, d, *J* 2, Ar-*H*), 9.00 (1H, t, *J* 2. Ar-*H*), 8.28 (1H, d, *J* 2, CONHN*H*), 7.38 – 7.16 (7H, m, Ar-*H*), 7.04 – 6.96 (2H, m, Ar-*H*), 4.54 (1H, q, *J* 7,

CONHC*H*(CH_2CH_2Phe)), 2.99 – 2.62 (2H, m, CONHCH(CH_2C*H*$_2$Phe)), 2.17 (2H, q, *J* 8, CONHCH(C*H*$_2$$CH_2$Phe)); δ_C (75 MHz, DMSO-d_6) 171.7 (*C*ONHNH), 163.4 (*C*ONH), 150.4 (*ipso*-Ar-C), 148.6 (*ipso*-Ar-*C*), 141.4 (*ipso*-Ar-*C*), 137.0 (*ipso*-Ar-*C*), 128.9 (Ar-*C*), 128.8 (Ar-*C*), 128.4 (Ar-*C*), 126.5 (*ipso*-Ar-C*CF*$_3$),121.5 (Ar-*C*), 116.4 (Ar-*C*), 115.0 (Ar-*C*), 53.4 (CONH*C*H(CH_2CH_2Phe)), 33.2 (CONHCH(*C*H$_2$$CH_2$Phe)), 32.3 (CONHCH($CH_2$*C*H$_2$Phe)); *m/z* ($ES^+$) 532 ($MH^+$); HRMS ($ES^+$) found MH^+, 532.1436 ($C_{24}H_{21}F_3N_5O_6$ requires 532.1444).

(S)-*N*-(1-(2-(4-chlorophenyl)hydrazineyl)-1-oxo-4-phenylbutan-2-yl)-3,5-dinitrobenzamide, 52

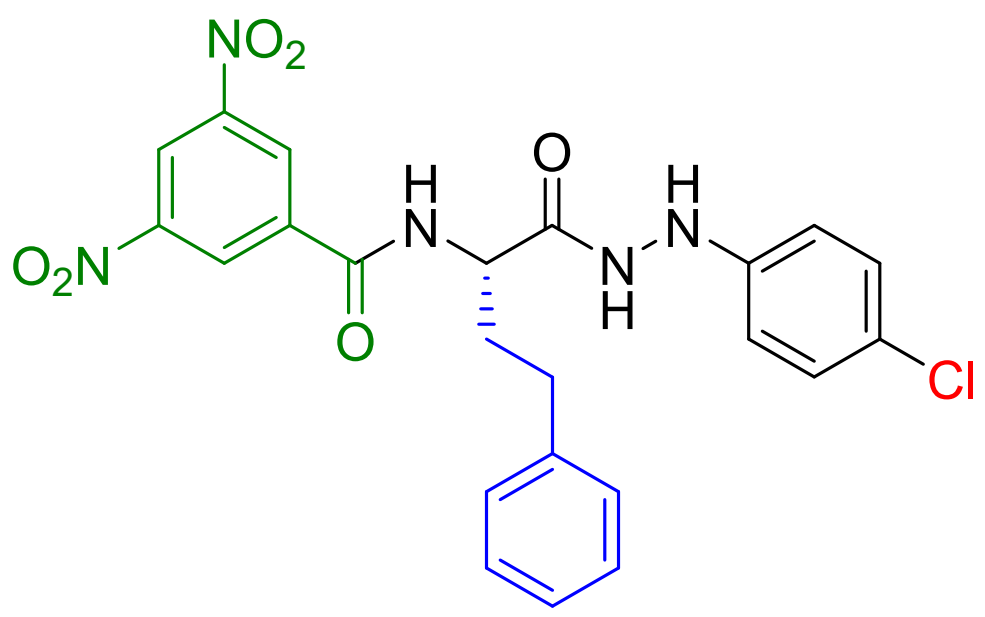

Following the general standard procedure, *tert*-butyl (S)-(1-(2-(4-chlorophenyl)hydrazineyl)-1-oxo-4-phenylbutan-2-yl)carbamate (0.10 g, 0.25 mmol) was transformed following flash column chromatography (DCM/EtOAc) [3:1] to produce the title compound as a white solid (0.08 g, 63 %); R_f 0.60 (*n*-hexane/EtOAc) [3:1]; m.p. 106 - 108 °C; ν_{max} 3639, 1644, 1540, 1324, 1157, 1099, 917, 833 cm^{-1}; δ_H (300 MHz, DMSO-d_6) 10.08 (1H, d, *J* 3 CON*H*NH), 9.47 (1H, d, *J* 8 , CON*H*), 9.15 (2H, d, *J* 2, Ar-*H*), 8.99 (1H, t, *J* 2, Ar-*H*), 7.99 (1H, d, *J* 3, CONHN*H*), 7.33 – 7.18 (5H, m, Ar-*H*), 7.16 (2H, d, *J* 9, Ar-*H*), 6.72 (2H, d, *J* 9, Ar-*H*), 4.55 (1H, q, *J* 8, CONHC*H*(CH_2CH_2Phe)), 2.86 – 2.59 (2H, m, CONHCH(CH_2C*H*$_2$Phe)), 2.17 (2H, t, *J* 8, CONHCH(C*H*$_2$$CH_2$Phe)); δ_C (75 MHz, DMSO-d_6) 171.1 (*C*ONHNH), 162.9; (*C*ONH), 148.1 (*ipso*-Ar-*C*), 141.0 (*ipso*-Ar-*C*), 136.7 (*ipso*-Ar-*C*), 128.5 (Ar-*C*), 128.0 (Ar-*C*), 125.9 (Ar-*C*), 121.8 (Ar-*C*), 121.0 (*ipso*-Ar-*C*), 113.7 (Ar-*C*), 52.9 (CONH*C*H(CH_2CH_2Phe)), 33.0 (CONHCH(*C*H$_2$$CH_2$Phe)), 31.9 CONHCH($CH_2$*C*H$_2$Phe)); *m/z* ($ES^+$) 498 ([^{35}Cl]$MH^+$) 500 ([^{37}Cl]$MH^+$); HRMS ($ES^+$) found [^{35}Cl] MH^+, 498.1177 ($C_{23}H_{20}{}^{35}ClN_5O_6$ requires 498.1180).

(S)-*N*-(1-(2-(4-bromophenyl)hydrazineyl)-1-oxo-4-phenylbutan-2-yl)-3,5-dinitrobenzamide, 53

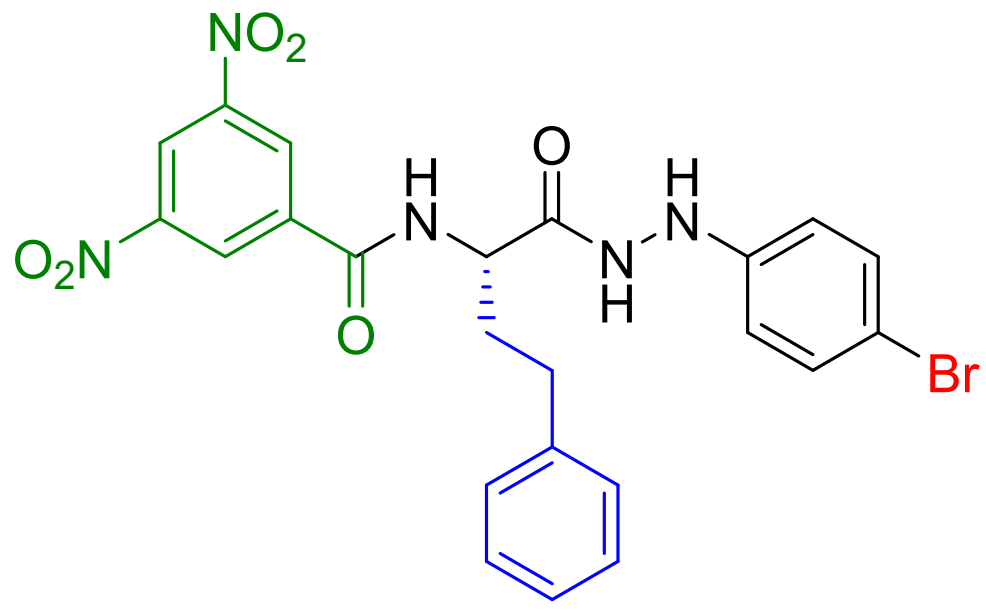

Following the general standard procedure, *tert*-butyl (S)-(1-(2-(4-bromophenyl)hydrazineyl)-1-oxo-4-phenylbutan-2-yl)carbamate (0.10 g, 0.22 mmol) was transformed following flash column chromatography (*n*-hexane/EtOAc) [3:1] to produce the title compound as a white solid (0.08 g, 63 %); R_f 0.56 (DCM/EtOH) [200:6]; m.p. 112 - 114 °C; ν_{max} 3283, 1640, 1537, 1485, 1342, 1070, 914, 820, 686 cm^{-1}; δ_H (300 MHz, DMSO-d$_6$) 10.11 (1H, d, *J* 2., CON*H*NH), 9.50 (1H, d, *J* 7, CONH), 9.17 (2H, d, *J* 2, Ar-*H*), 8.99 (1H, t, *J* 2, Ar-*H*), 8.11 (1H, d, *J* 2.,CONHN*H*), 7.34 – 7.16 (6H, m, Ar-*H*), 7.07 (1H, t, *J* 8., Ar-*H*), 6.90 (1H, t, *J* 2.,Ar-*H*), 6.86 – 6.81 (2H, m, Ar-*H*), 6.76 – 6.66 (2H, m, Ar-*H*), 4.53 (1H, q, *J* 7,CONHC*H*(CH_2CH_2Phe)), 2.87 – 2.61 (2H, m, CONHCH(CH_2CH_2Phe)), 2.16 (2H, q, *J* 8, CONHCH(CH_2CH_2Phe)). δC (75 MHz, DMSO-d$_6$) 171.6 (*C*ONHNH), 163.4 (*C*ONH), 151.4 (*ipso*-Ar-*C*), 148.6 (*ipso*-Ar-*C*), 137.1 (*ipso*-Ar-*C*), 131.1 (*ipso*-Ar-*C*), 128.9 (Ar-*C*), 128.5 (Ar-*C*), 126.5 (Ar-*C*), 122.6 (Ar-*C*), 121.5 (Ar-*C*), 121.2 (Ar-*C*), 114.8 (Ar-*C*), 111.7 (Ar-*C*), 79.6 (Ar-*C*), 53.4 (CONH*C*H(CH_2CH_2Phe)), 33.3 (CONHCH(*C*H_2CH_2Phe)); 32.3 (CONHCH(CH_2*C*H_2Phe)); *m/z* (ES$^+$) 542 ([^{79} Br]MH$^+$) 543 ([^{81} Br]MH$^+$); HRMS (ES$^+$) found [^{81} Br]MH$^+$, 544.0645 ($C_{23}H_{20}{}^{81}BrN_5O_6$ requires 544.0659).

(S)-3,5-dinitro-*N*-(1-oxo-4-phenyl-1-(2-(4-(trifluoromethyl)phenyl)hydrazineyl)butan-2-yl)benzamide, 54

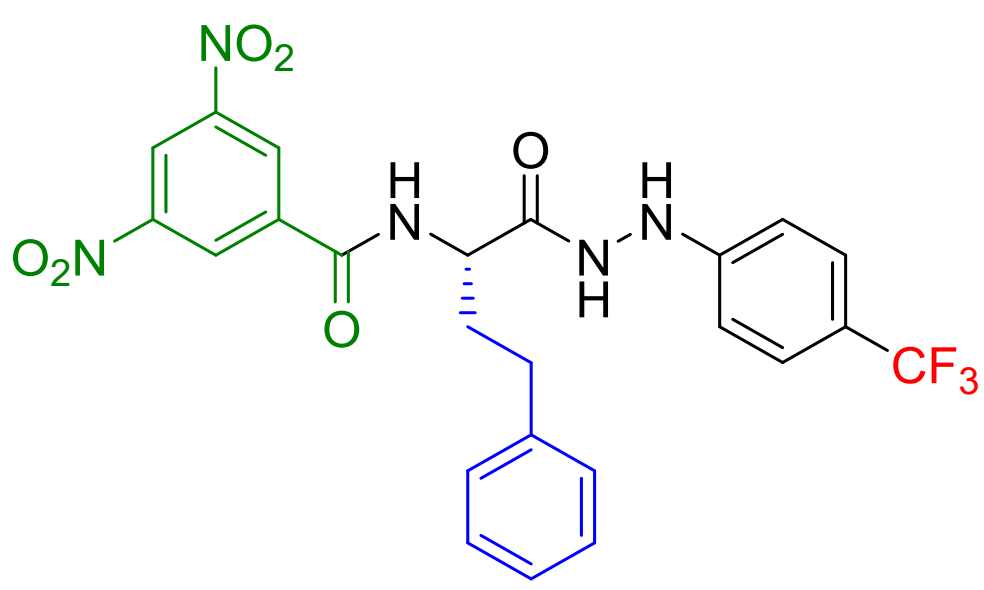

Following the general standard procedure, *tert*-butyl (S)-(1-oxo-4-phenyl-1-(2-(4-(trifluoromethyl)phenyl)hydrazineyl)butan-2-yl)carbamate (0.09 g, 0.20 mmol) was transformed following trituration with diethyl ether to produce the title compound as a yellow solid (0.05 g, 26 %); R_f 0.50 (*n*-hexane/EtOAc) [3:1]; m.p 113 - 115 °C; ν_{max} 3275, 1644, 1537, 1342, 1158, 1066, 697 cm^{-1}; δ_H (300 MHz, DMSO-d$_6$) 10.20 (1H, d, *J* 2,CON*H*NH), 9.52 (1H, d, *J* 7, CON*H*), 9.17 (2H, d, *J* 2, Ar-*H*), 9.00 (1H, t, *J* 2. Ar-*H*), 8.28 (1H, d, *J* 2, CONHN*H*), 7.38 – 7.16 (7H, m, Ar-*H*), 7.04 – 6.96 (2H, m, Ar-*H*), 4.54 (1H, q, *J* 7, CONHC*H*(CH_2CH_2Phe)),

2.99 – 2.62 (2H, m, CONHCH(CH_2C*H_2*Phe)), 2.17 (2H, q, *J* 8, CONHCH(C*H_2*CH_2Phe)); δ_C (75 MHz, DMSO-d_6) 171.7 (*C*ONHNH), 163.4 (*C*ONH), 152.8 (*ipso*-Ar-*C*), 148.6 (*ipso*-Ar-*C*), 141.4 (*ipso*-Ar-*C*) 137.1 (*ipso*-Ar-*C*), 128.9 (*ipso*-Ar-C*C*F_3), 128.8 (Ar-*C*), 128.5 (Ar-*C*), 126.6 (Ar-*C*), 126.6 (Ar-*C*), 126.5 (Ar-*C*), 123.8 (Ar-*C*), 121.5 (Ar-*C*), 111.9 (*ipso*-Ar-*C*), 53.3 (CONH*C*H(CH_2CH_2Phe)), 33.4 (CONHCH(*C*H_2CH_2Phe)), 32.3 (CONHCH(CH_2*C*H_2Phe)); δ_F (282 MHz, DMSO-d_6) -59.29 (3F, s, C*F_3*); *m/z* (ES$^+$) 554 (MNa^+), HRMS (ES$^+$) found MNa^+, 554.1261 ($C_{24}H_{21}F_3N_5O_6$ requires 554.1258).

S3. Biological Data and Experimental Procedures

Materials. All reagents were purchased from Sigma-Aldrich unless stated otherwise. BSL2 bacterial species used in this study were all kindly supplied by Prof William R. Jacobs Jr.[1]

Table Strains and plasmids used in this study

**Plasmid and Strain**	**Description**	**Source or reference**
**Plasmids**		
pMV261	*E. coli*-mycobcaterial shuttle plasmid, *hsp60* promoter, KanR	Stover *et al*., 1991
**Strains**		
Mtb mc²7902	*ΔleuCD ΔpanCD ΔargB,* Leucine, pantothenate and arginine triple auxotroph	Vilcheze *et al*., 2018
Mtb mc²8245 (INH^R)	mc²7902 derived Δ2116169–2162530; Δ2116169–2162530 genome deletion, INH^R	Vilcheze *et al*., 2018
Mtb mc²8247 (RIF^R)	mc²7902 derived, *rpoB* (H445Y); *rpoB* His445 → Lys, RIF^R	Vilcheze *et al*., 2018
Mtb mc²8250 (RIF^R & INH^R)	mc²8247 derived, *rpoB* (H445Y) Δ2122397–2170320; *rpoB* His445 → Lys, RIF^R, Δ2122397–2170320 genome deletion, INH^R	Vilcheze *et al*., 2018
Mtb mc²7902 pMV261	*Mtb* mc²7902 containing pMV261, Kan^R	Yu *et al.*, 2025
Mtb mc²8245 pMV261	*Mtb* mc²8245 containing pMV261, Kan^R	Yu *et al.*, 2025
Mtb mc²8247 pMV261	*Mtb* mc²8247 containing pMV261, Kan^R	Yu *et al.*, 2025
Mtb mc²8250 pMV261	*Mtb* mc²8250 containing pMV261, Kan^R	Yu *et al.*, 2025
Mtb mc²8258 pMV261	*Mtb* mc²8258 containing pMV261, Kan^R	Yu *et al.*, 2025

Mycobacterial culture

Mycobacterial species were cultured in either Middlebrook 7H9 broth or Middlebrook 7H11 agar media supplemented with albumin–dextrose–catalase (ADC) or oleic acid–albumin–dextrose–catalase (OADC) enrichments (BD Biosciences) and 0.2 % glycerol, 0.1 % w/v casamino acids, 24 μg mL^{-1} pantothenate, 200 μg mL^{-1} arginine, 50 μg mL^{-1} leucine, 1 μg mL^{-1} penicillin G, and 10 μg mL^{-1} cyclohexamide (7H9OPALPen^{1}Cyc^{10}, broth or 7H11OPALPen^{1}Cyc^{10}, agar).

Minimum inhibition concentration (MIC$_{95}$).

Bacterial minimum inhibition concentrations (MIC) were determined using the Resazurin Microtiter Assay (REMA) method according to Palomino *et al.*[3] Stock solutions of the tested compounds were prepared in sterile dimethyl sulfoxide (DMSO), then diluted in

7H9OPALPen[1]Cyc[10] to obtain a final drug concentration range of 0.032 – 32 µg mL^{-1}. A suspension of the test *Mtb* was cultured in 7H9OPALPen[1]Cyc[10] and 0.05 % v/v Tween® 80 for one week at 37 °C in an atmosphere of 5 % CO_2. The concentration was adjusted to 1 × 10^6 CFU mL^{-1} and diluted in 7H9OPALPen[1]Cyc[10] 1:50. 100 µL of the inoculum was added to each well of a 96-well microplate together with 100 µL of the compounds. The plate was incubated at 37 °C in an atmosphere of 5 % CO_2. After 5 days, 10.5 µL 0.1 % (w/v) resazurin (solubilized in sterile PBS containing 0.02 % v/v Tween® 80) was added. The resazurin assay protocol is based on the reduction of oxidized non-fluorescent blue resazurin to a red fluorescent dye (resorufin) by the respiratory chain in live cells. The amount of resorufin produced is directly proportional to the number of living cells.

S4. Cytotoxicity testing

RAW 264.7 cell lines were purchased from the American Type Culture Collection, Middlesex, UK(ATCC1 TIB-71™). Cells were cultured in DMEM high glucose medium, supplemented with 10 % FBS, 2 mM L-Glutamine and 1 % penicillin/streptomycin, at 37 °C in a humidified incubator containing 5 % CO_2. Cells were passaged after reaching 90 % confluence. Cells, in logarithmic growth, were plated in 96-well plates at a density of 1 ×104 cells/well in 200 µL of media and incubated for 24 h. The media on the cells was removed and replaced with 180 µL of fresh media. To each respective well, 10 µL of test compound at a concentration of 1 mM was added, resulting in a final compound concentration of 50 µM. To control wells, DMSO drug solvent was added. In all wells, the final concentration of DMSO did not exceed 0.1 %. Following incubation for a further 96 h at 37 °C in a humidified incubator containing 5 % CO_2, all media was removed and replaced with 100 µL of fresh media. To each well, 20 µL of 3-(4,5-dimethylthiazol-2-yl)-5-(3-carboxymethoxyphenyl)-2-(4-sulfophenyl)-2H-tetrazolium, inner salt (MTS) assay solution (Promega, Madison, WI, USA) was added, and the plate was incubated in the dark at 37 ◦C for 4 h. Finally, the absorbance of each well was recorded at 490 nm using a microplate reader. The percentage of cell viability was calculated relative to the compound solvent (DMSO) control.

S5. Molecular modelling with *Mycobacterium tuberculosis* DprE1

The docking of compounds was performed using the SeeSAR molecular docking programme. The protein crystal structure *Mycobacterium tuberculosis* DprE1 protein crystal structure (PDB:4FDN) was utilised in this docking study. For clarity during docking, the whole protein was restricted to provide the active site only, along with the removal of the associated ligand. Water and other metals present outside of the region of interest were also excluded.

The binding site in DprE1 was identified using the binding site module in SeeSAR utilising the covalent inhibitor for reference to determine the active site to contain 28 residues, including the Cys-387 residue. Compounds were entered into the SeeSAR docking module and non-covalently bound poses were generated, with parameters set to produce 100 poses for each molecule, a medium clash tolerance and flexibility in aliphatic ring structures.

References

1. C. Vilcheze, J. Copeland, T. L. Keiser, T. Weisbrod, J. Washington, P. Jain, A. Malek, B. Weinrick and W. R. Jacobs, Jr., *mBio*, 2018, **9**, 9:e00938-00918.
2. C. K. Stover, V. F. de la Cruz, T. R. Fuerst, J. E. Burlein, L. A. Benson, L. T. Bennett, G. P. Bansal, J. F. Young, M. H. Lee, G. F. Hatfull, S. B. Snapper, R. G. Barletta, W. R. Jacobs and B. R. Bloom, *Nature*, 1991, **351**, 456-460.
3. J. C. Palomino, A. Martin, M. Camacho, H. Guerra, J. Swings and F. Portaels, *Antimicrob Agents Chemother*, 2002, **46**, 2720-2722.
4. Y. Lu, D. Partleton, F. M. Gugu, A. Y. G. Alhejaili, S. Norris, J. J. Harburn, J. H. Gill, J. D. Sellars and A. K. Brown, *J. Enzyme Inhib. Med. Chem.*, 2025, 40.

S6. ^{1}H and ^{13}C NMR for all compounds

***N*-(2-(2-(2-bromophenyl)hydrazineyl)-2-oxoethyl)-3,5-dinitrobenzamide, 11**

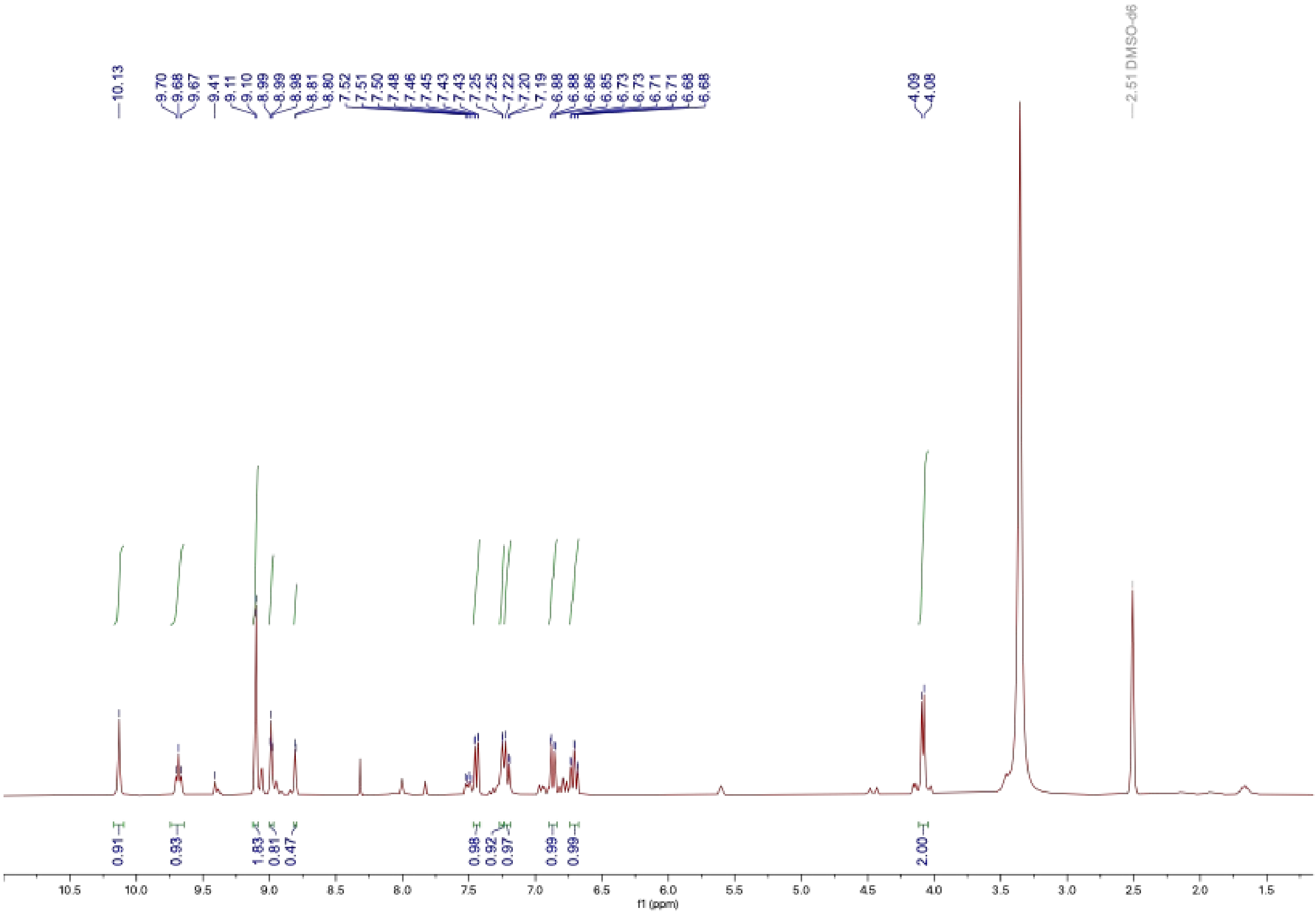

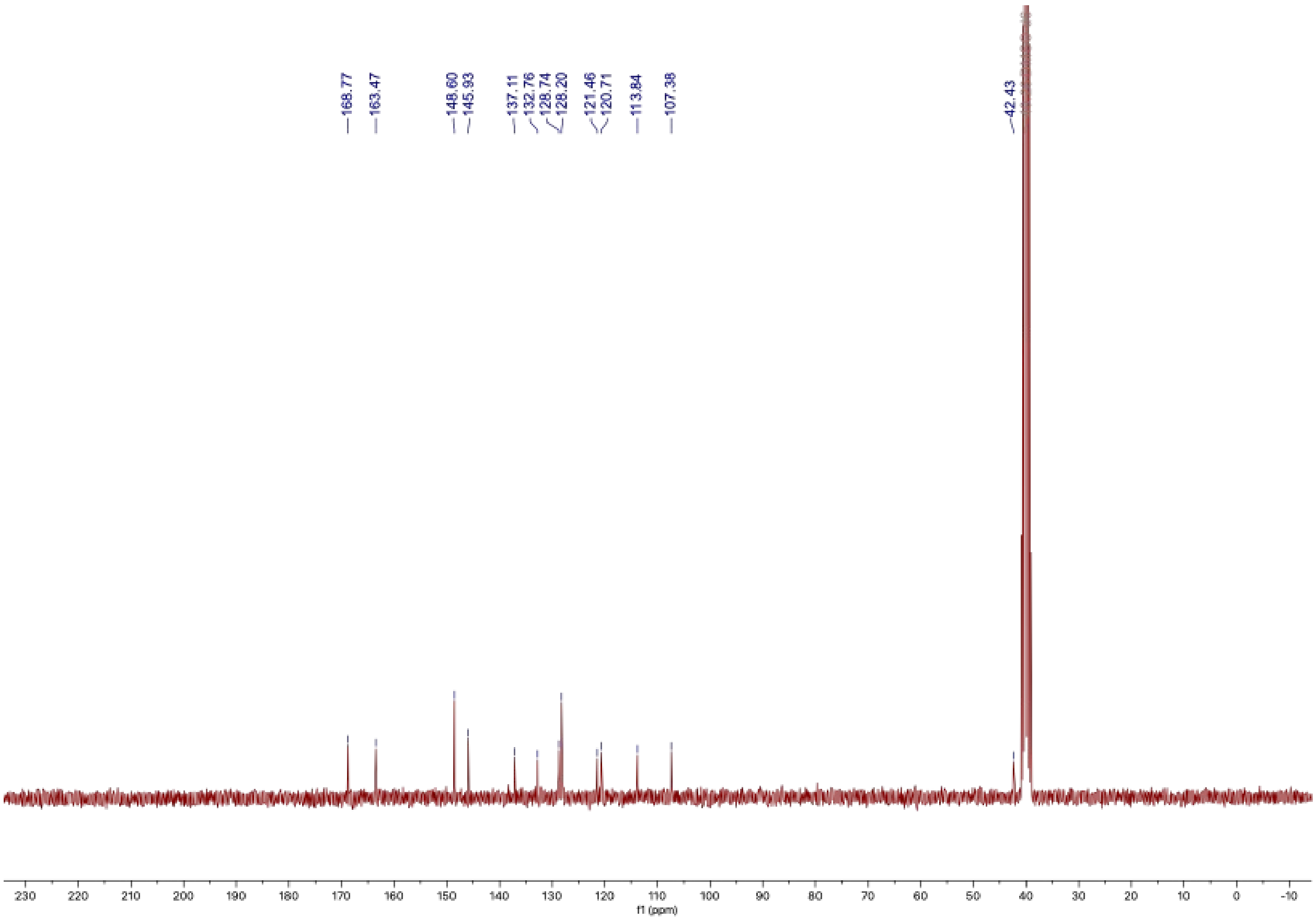

3,5-dinitro-*N*-(2-oxo-2-(2-(2-(trifluoromethyl)phenyl)hydrazineyl)ethyl)benzamide, 12

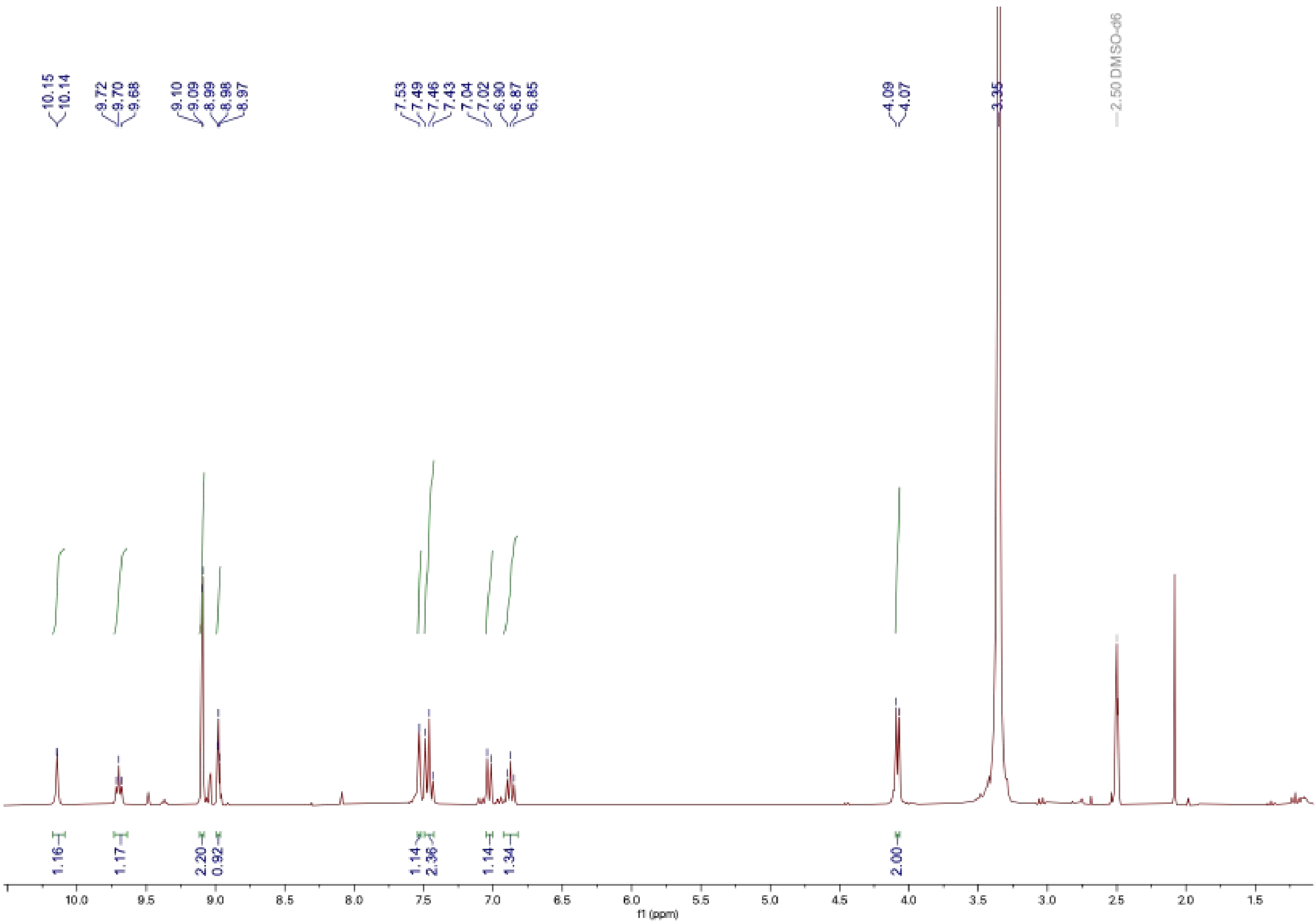

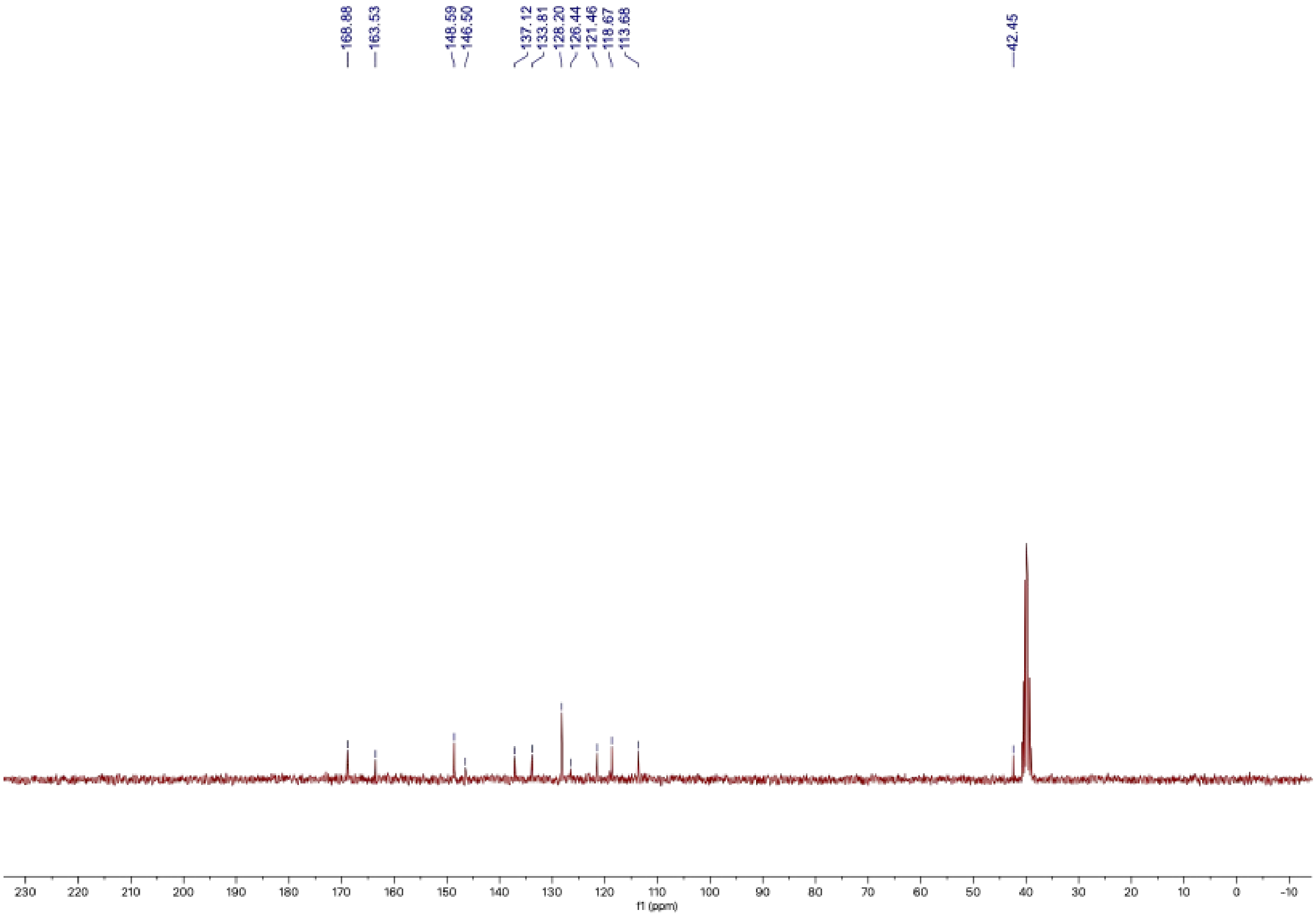

***N*-(2-(2-(3-chlorophenyl)hydrazineyl)-2-oxoethyl)-3,5-dinitrobenzamide, 13**

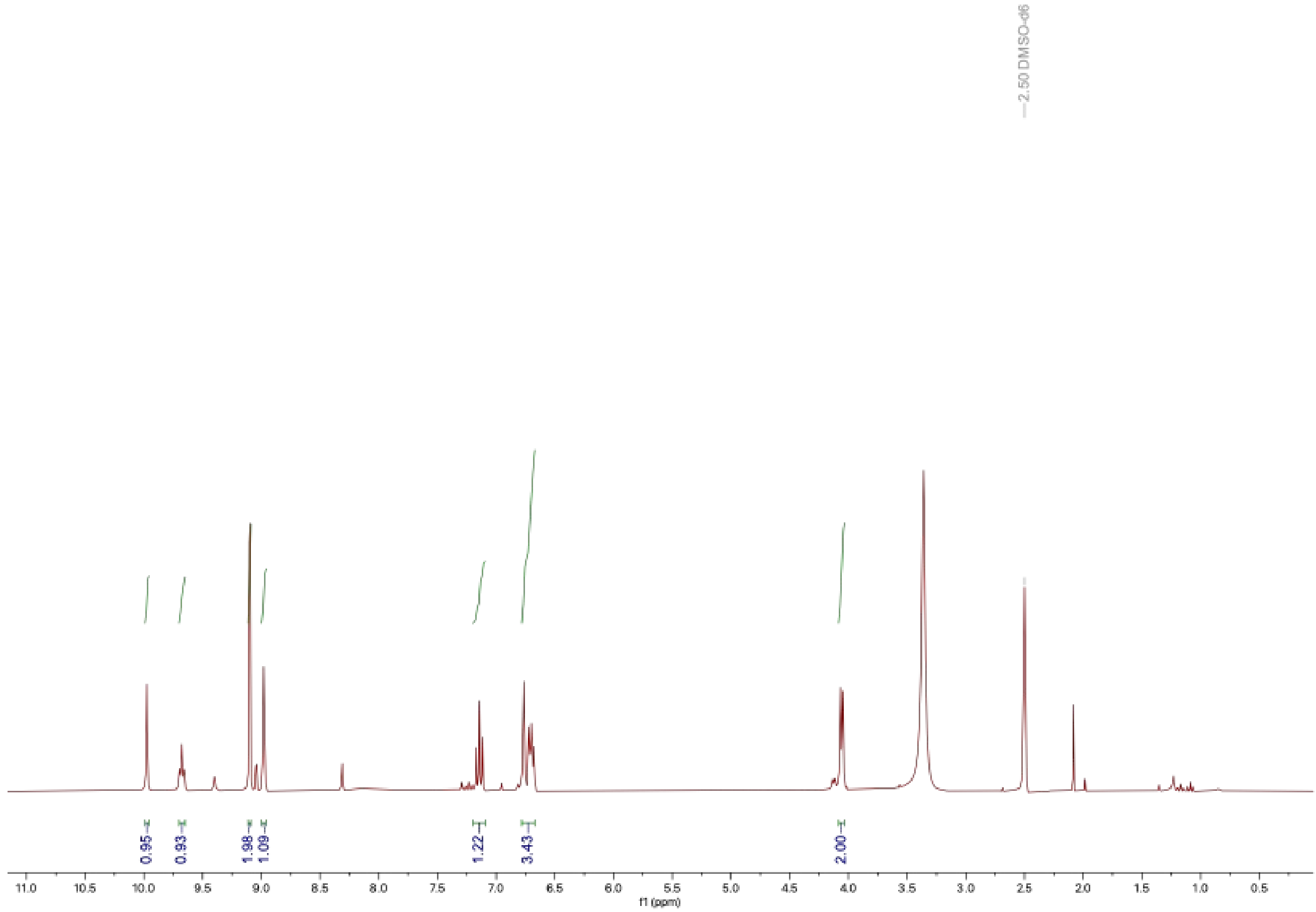

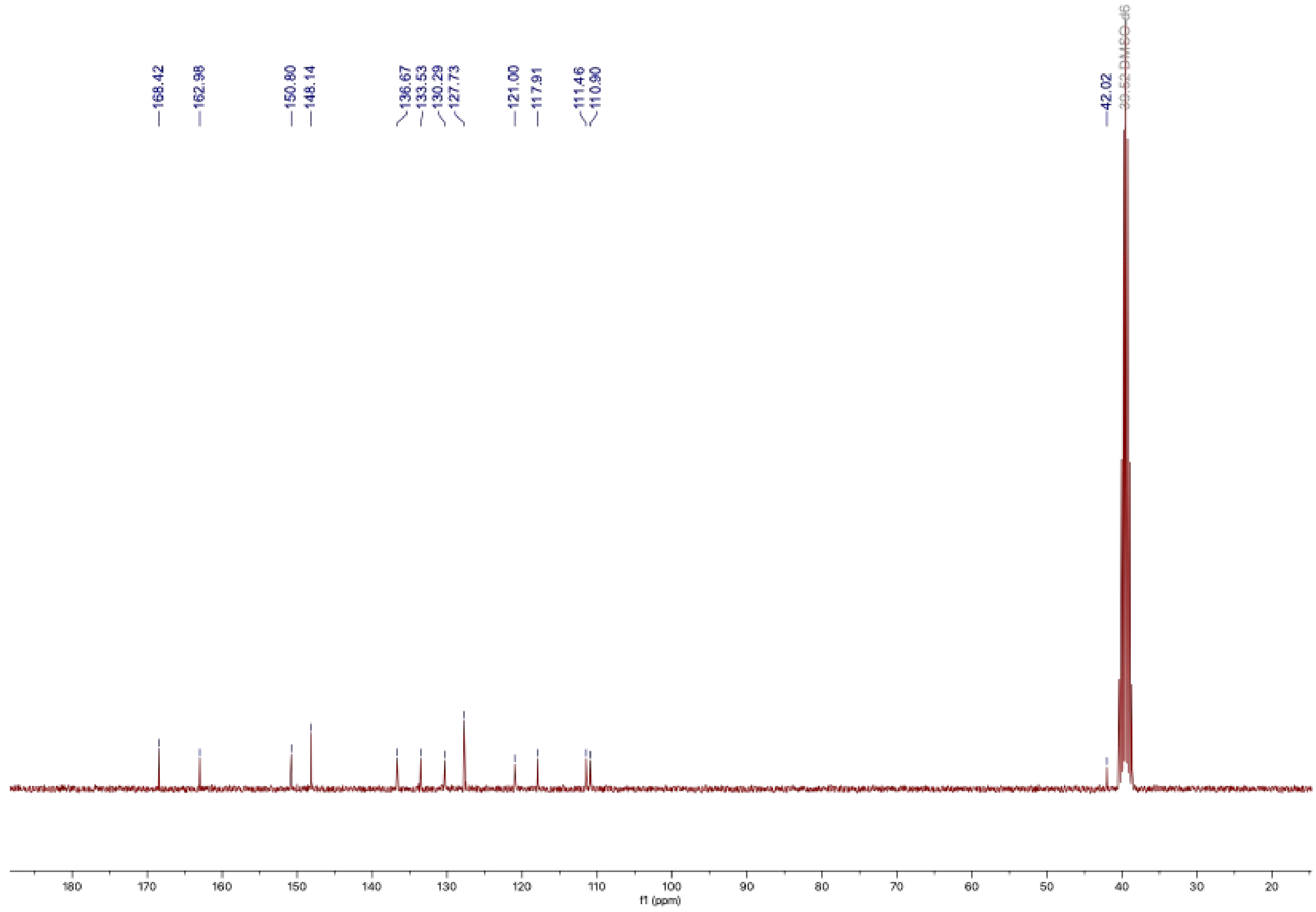

N-(2-(2-(3-bromophenyl)hydrazineyl)-2-oxoethyl)-3,5-dinitrobenzamide, 14

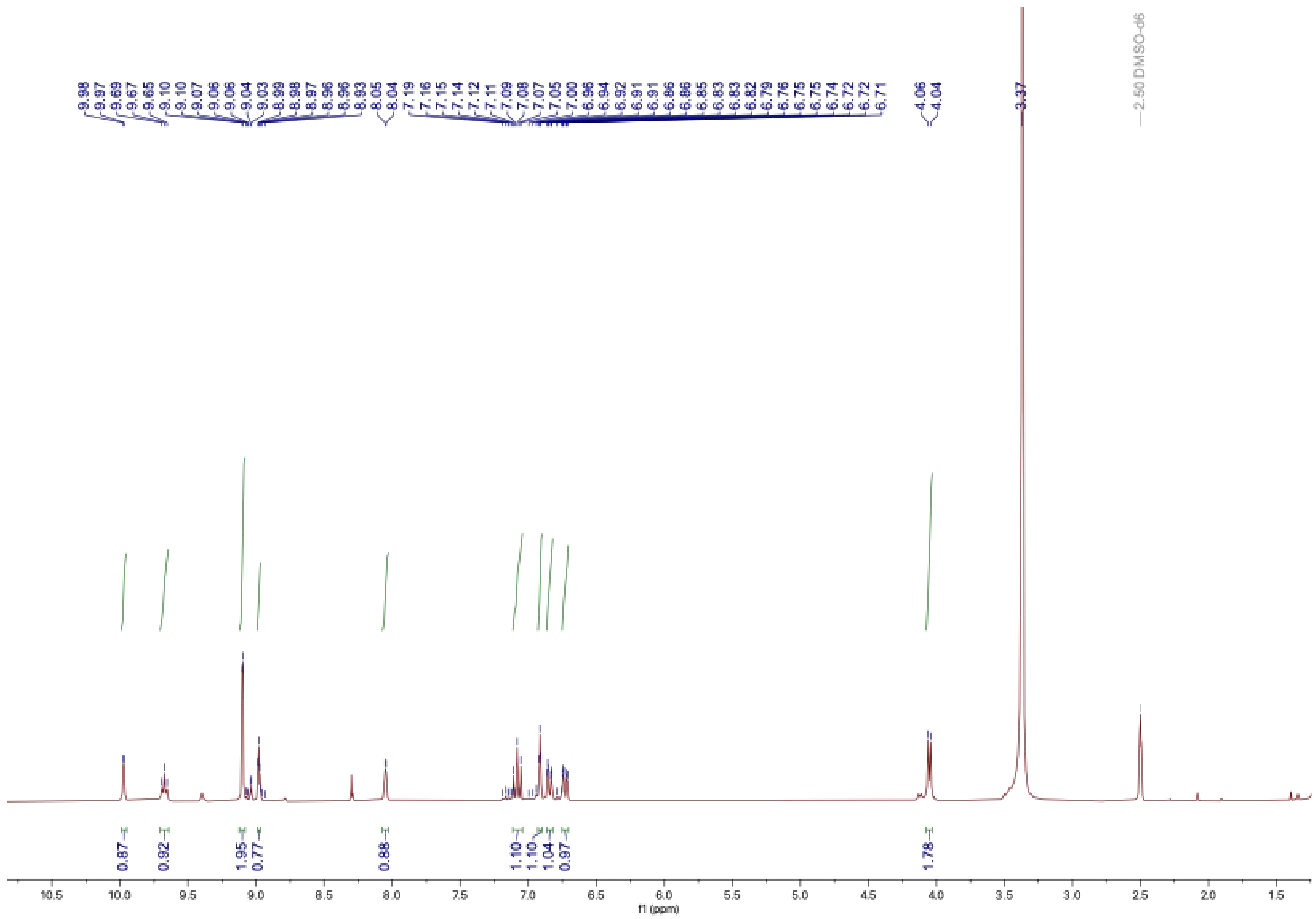

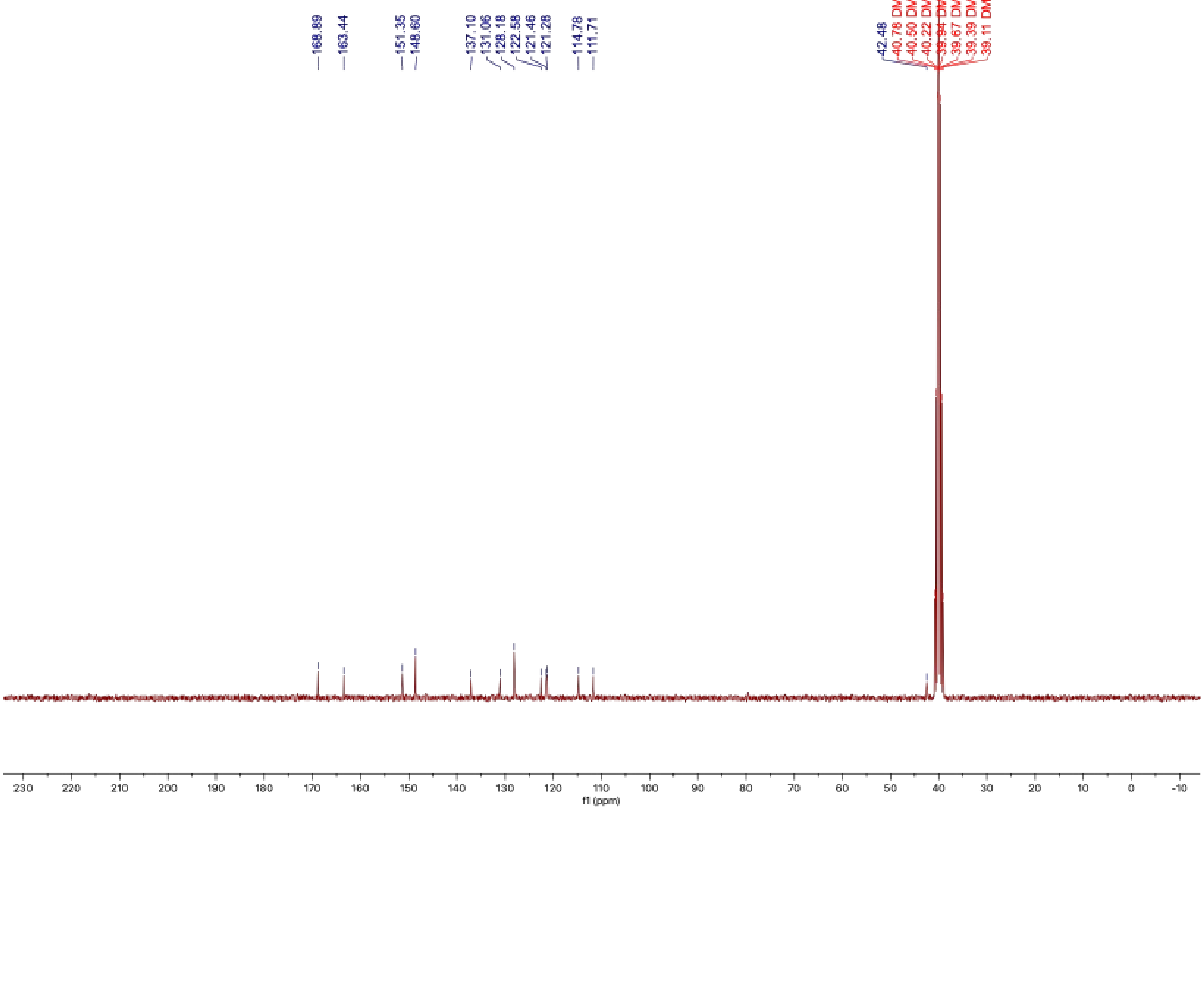

3,5-dinitro-*N*-(2-oxo-2-(2-(3-(trifluoromethyl)phenyl)hydrazineyl)ethyl)benzamide, 15

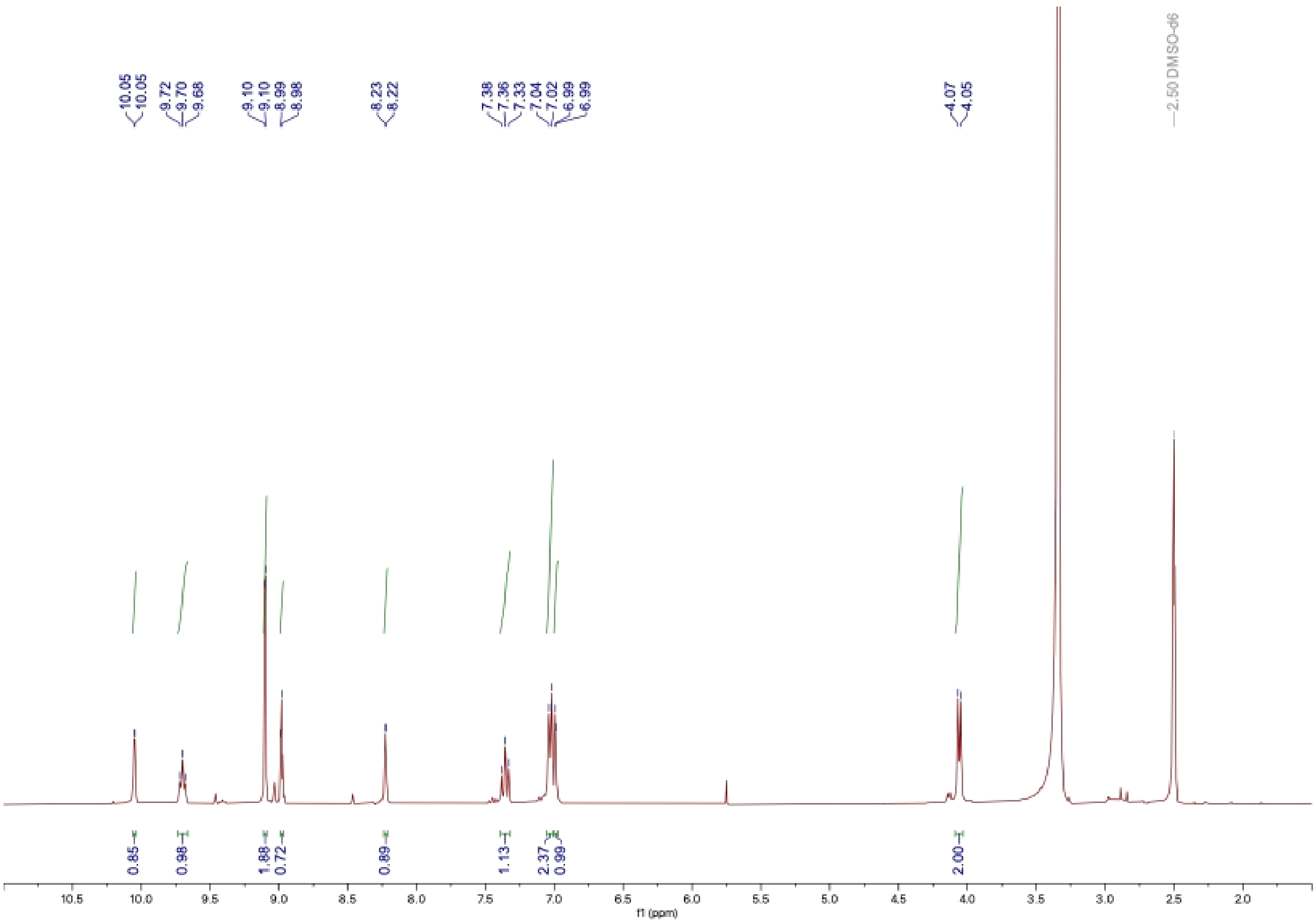

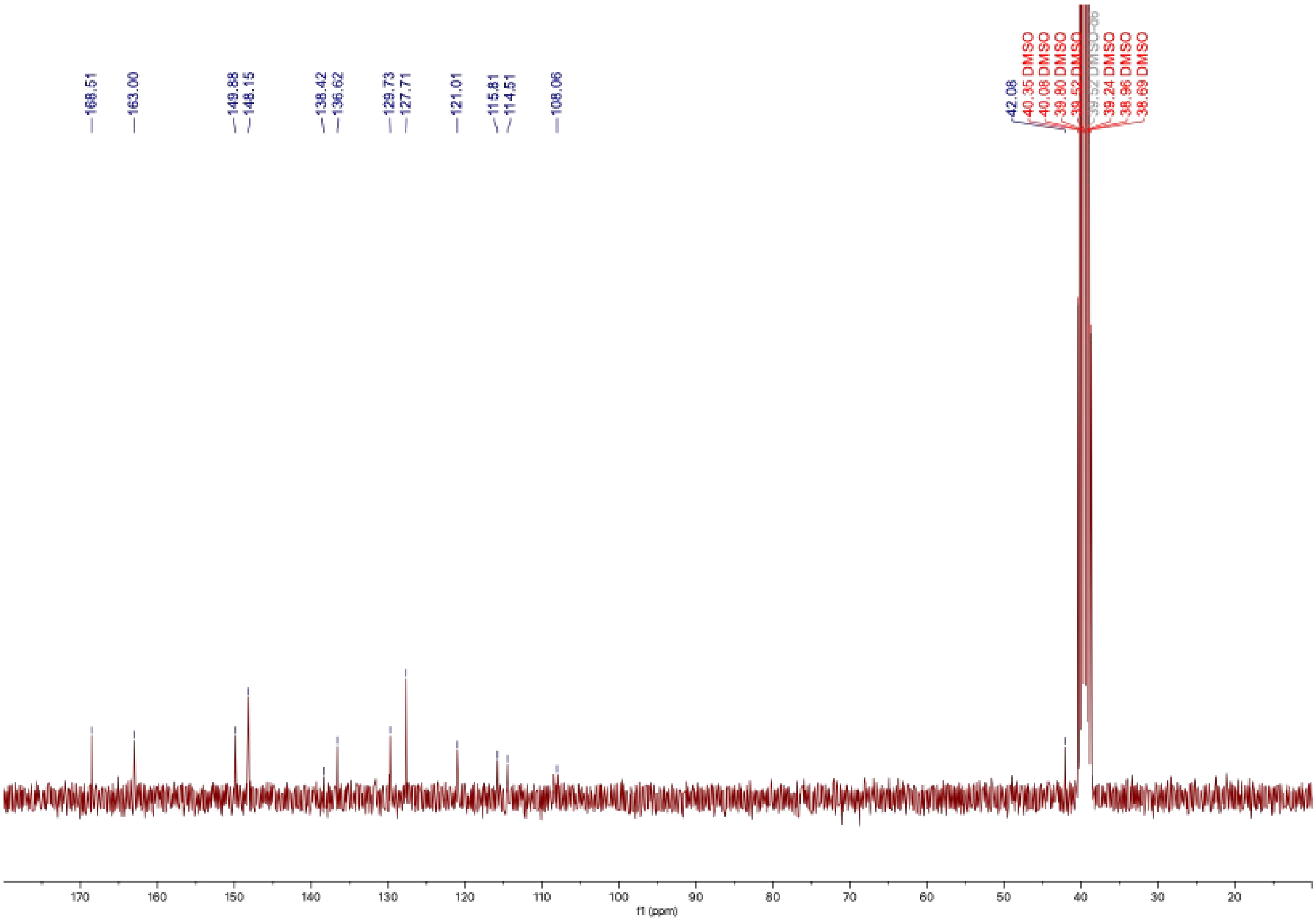

***N*-(2-(2-(4-chlorophenyl)hydrazineyl)-2-oxoethyl)-3,5-dinitrobenzamide, 16**

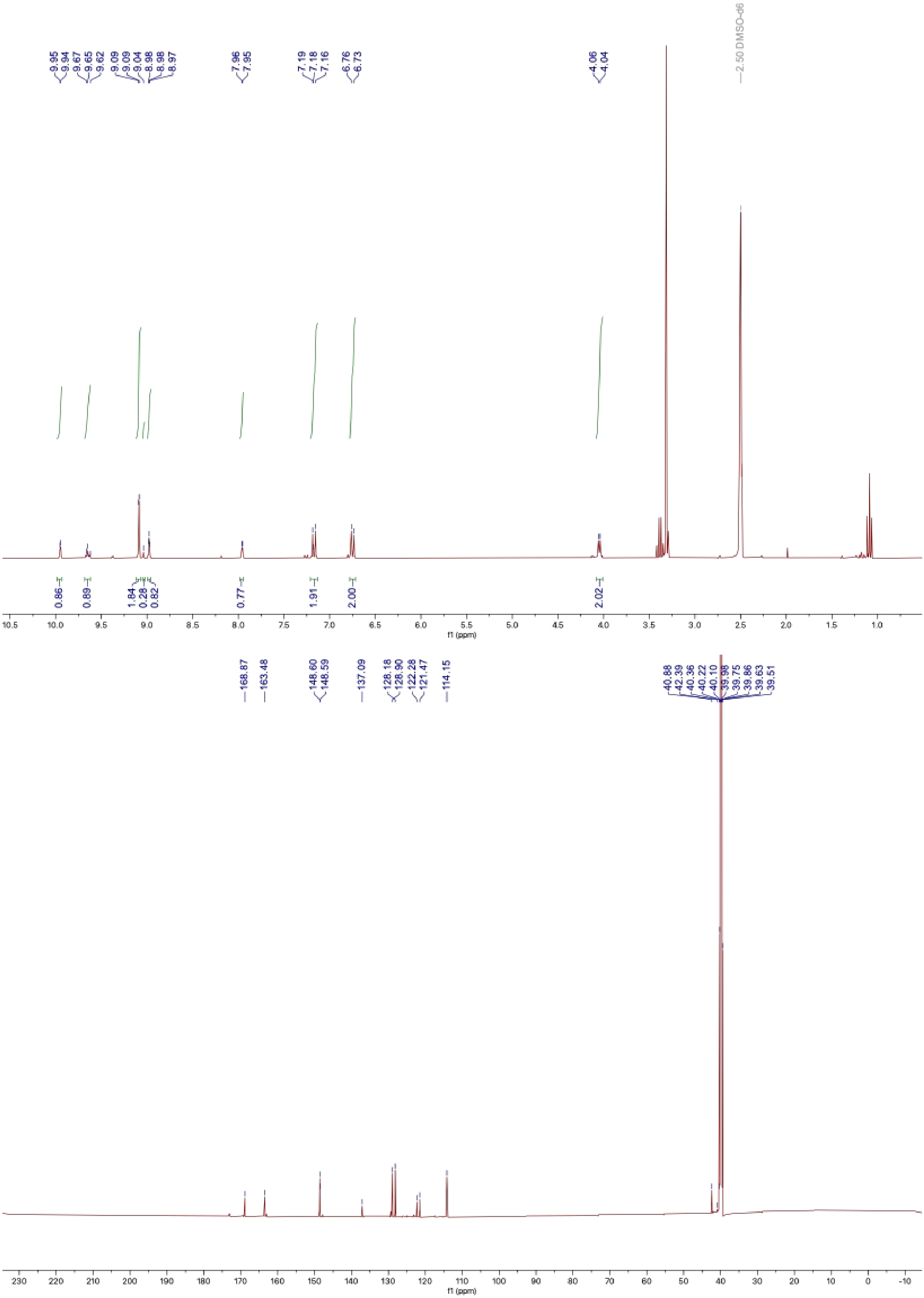

***N*-(2-(2-(4-bromophenyl)hydrazineyl)-2-oxoethyl)-3,5-dinitrobenzamide, 17**

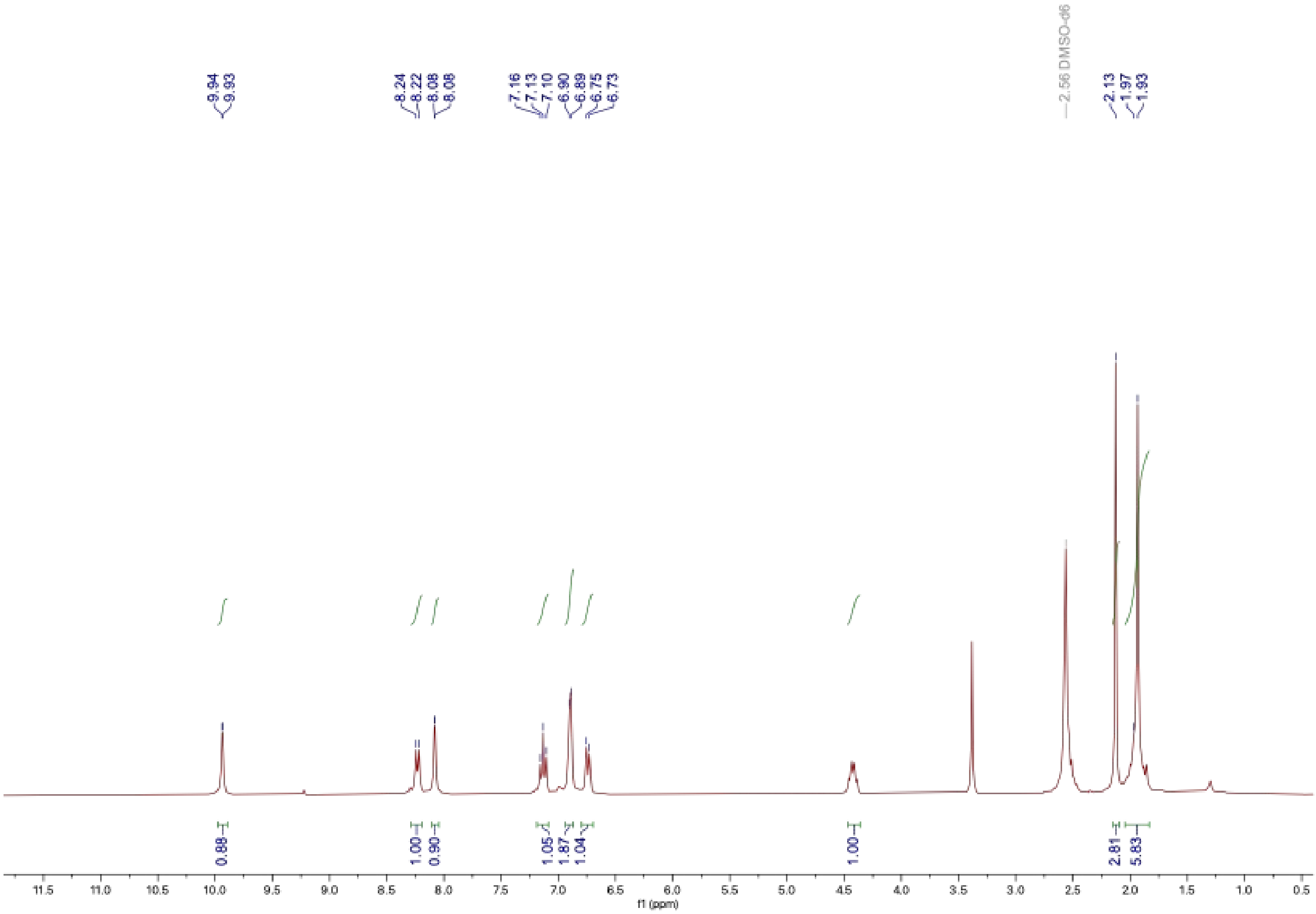

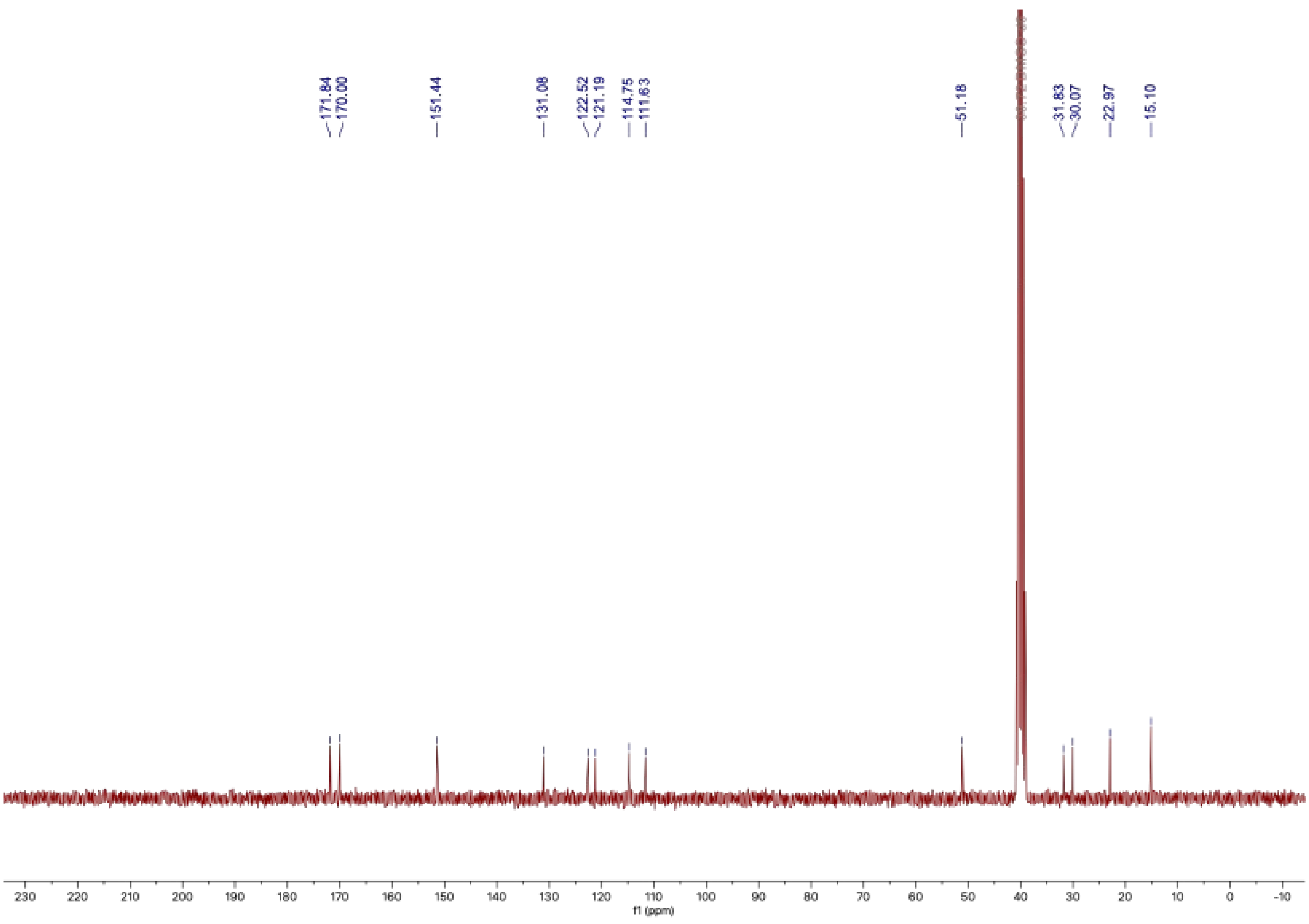

3,5-dinitro-*N*-(2-oxo-2-(2-(4-(trifluoromethyl)phenyl)hydrazineyl)ethyl)benzamide, 18

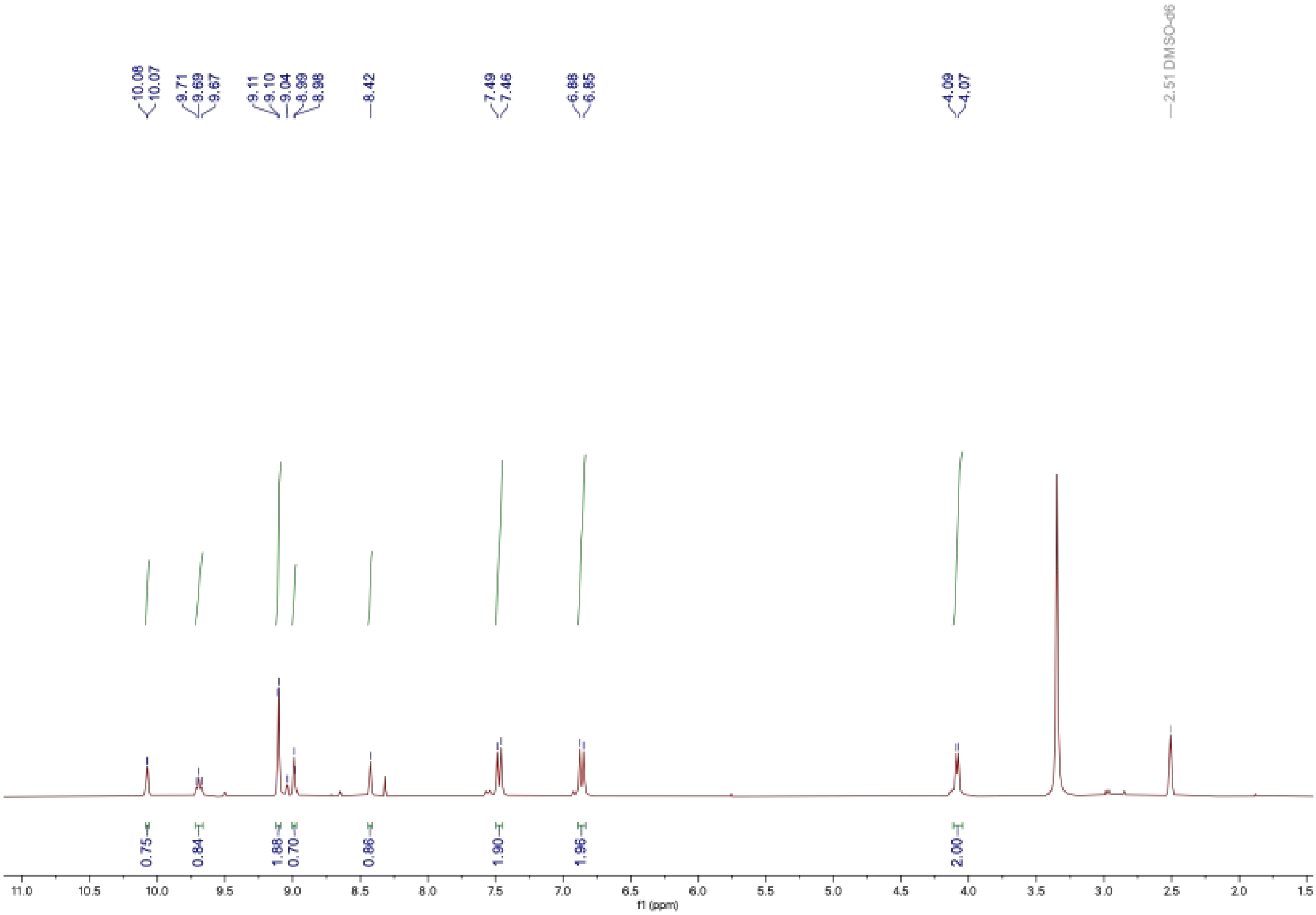

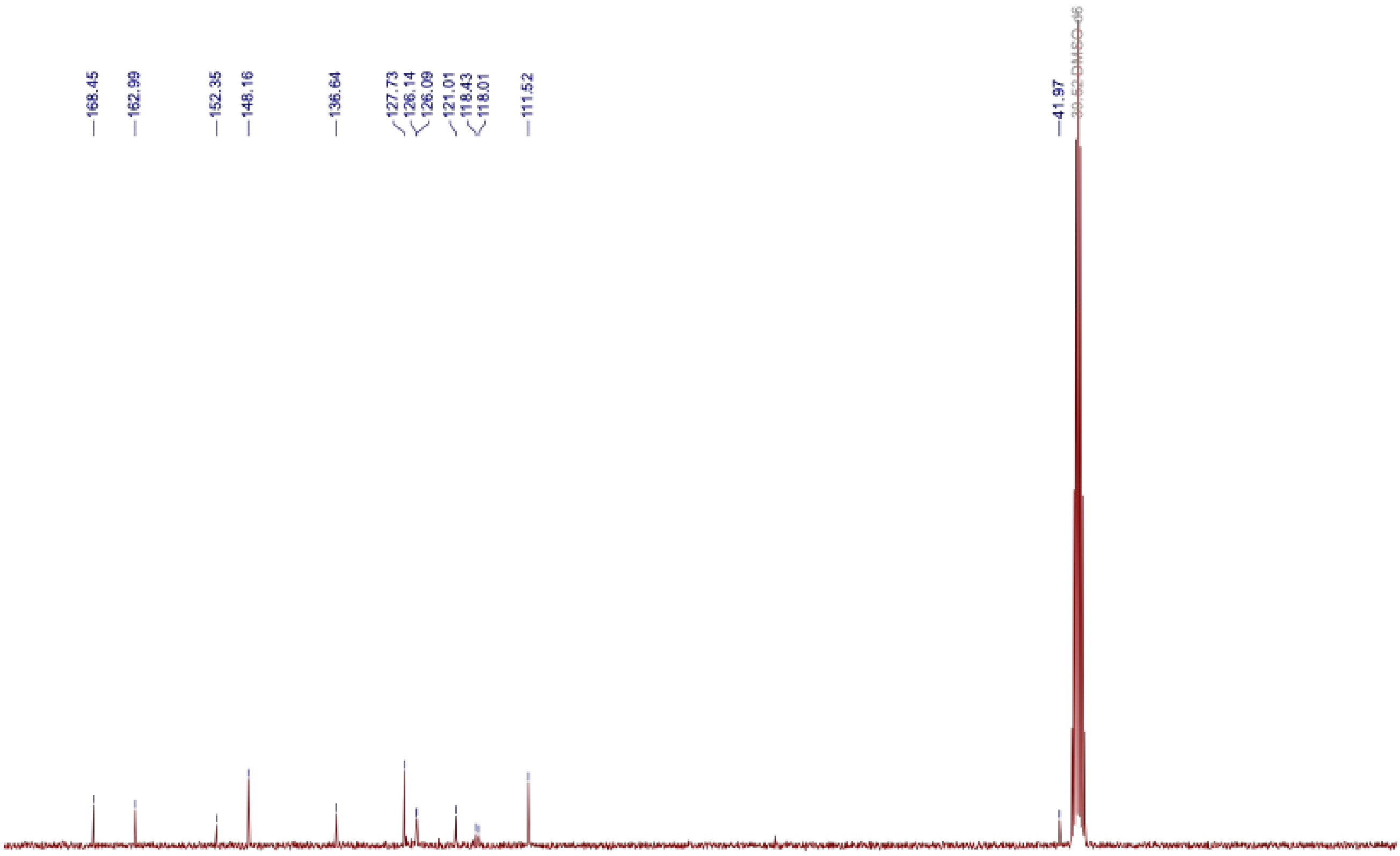

(S)-*N*-(1-(2-(2-bromophenyl)hydrazineyl)-1-oxopropan-2-yl)-3,5-dinitrobenzamide, 19

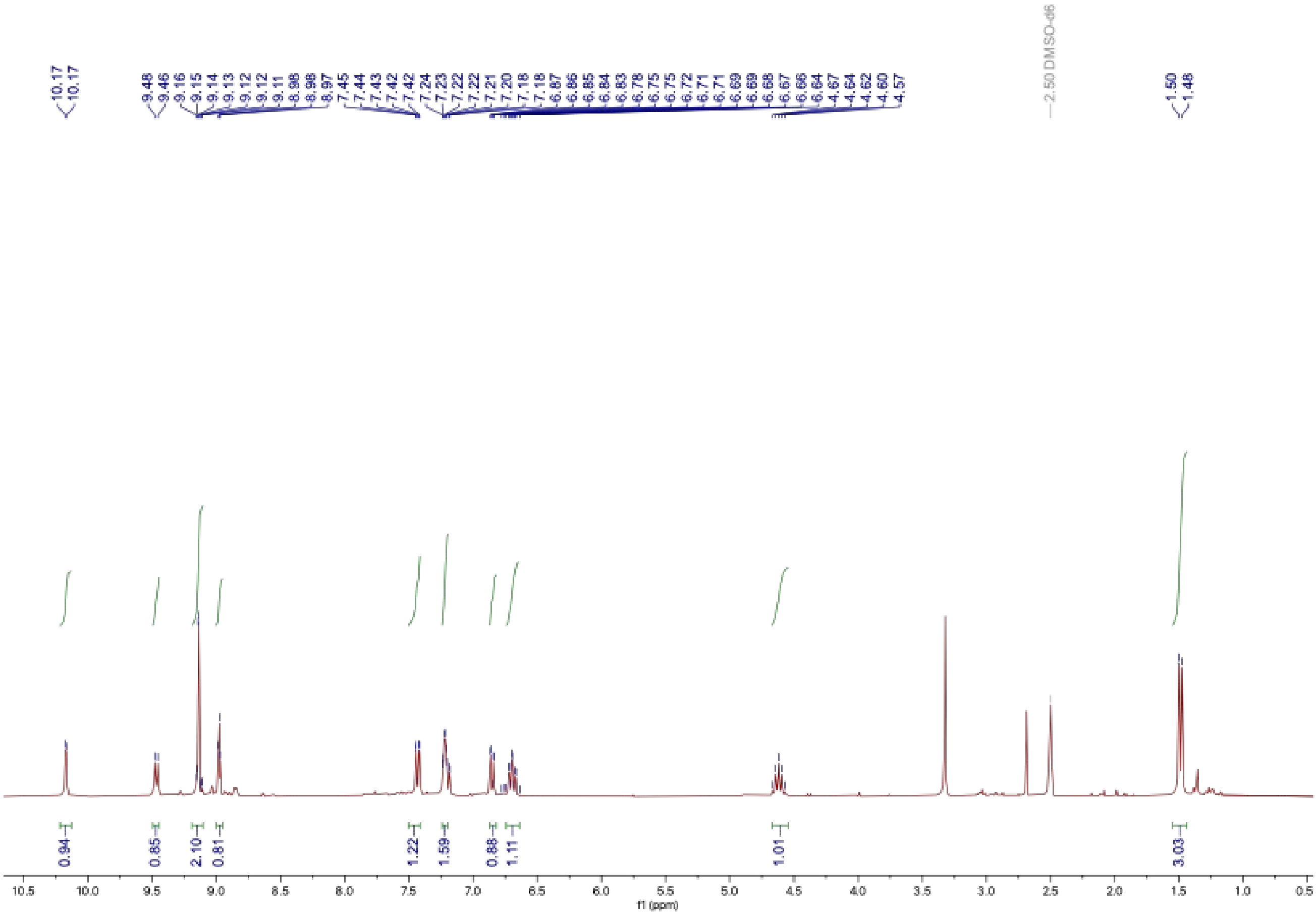

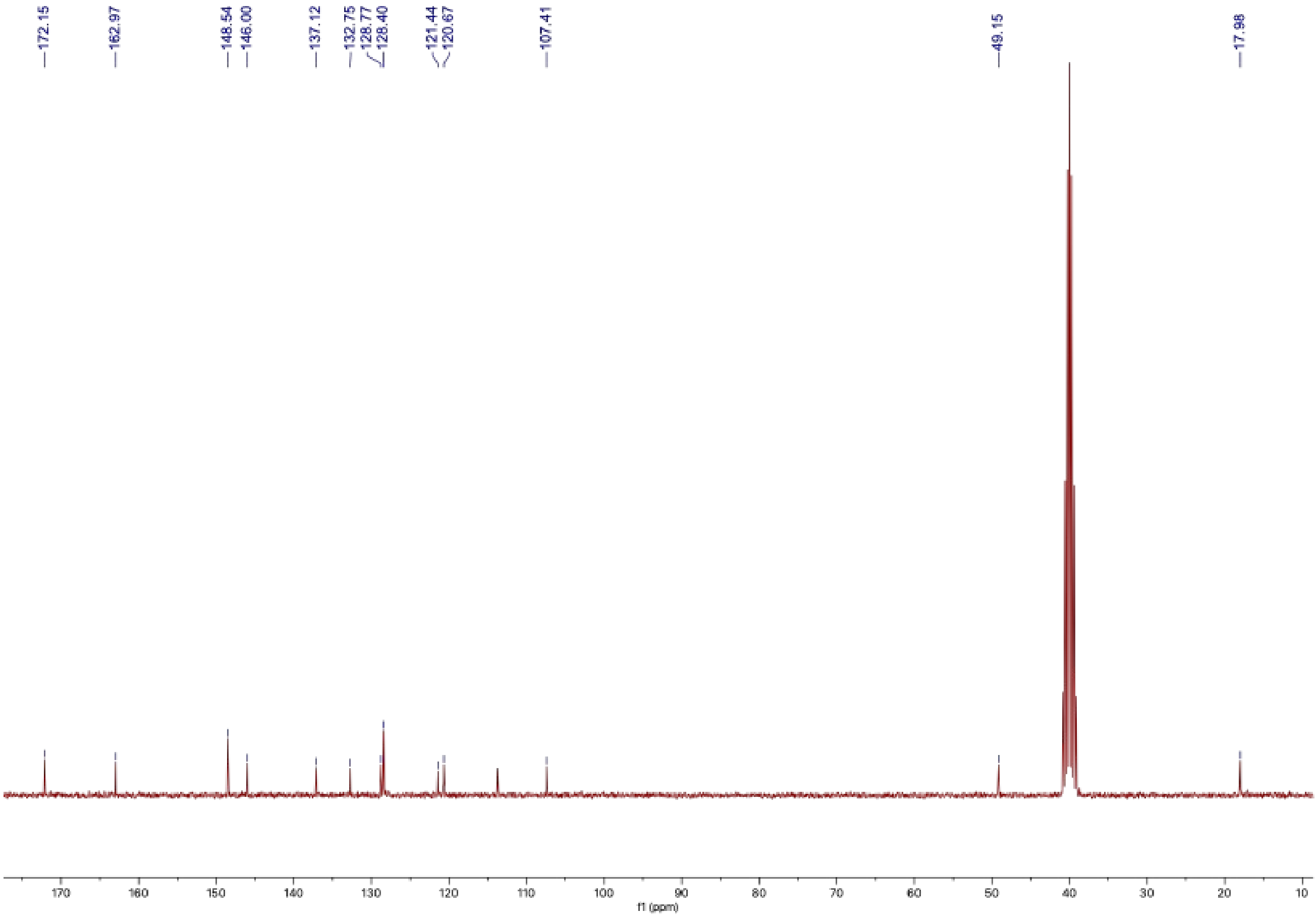

(S)-3,5-dinitro-*N*-(1-oxo-1-(2-(2-(trifluoromethyl)phenyl)hydrazineyl)propan-2-yl)benzamide, 20

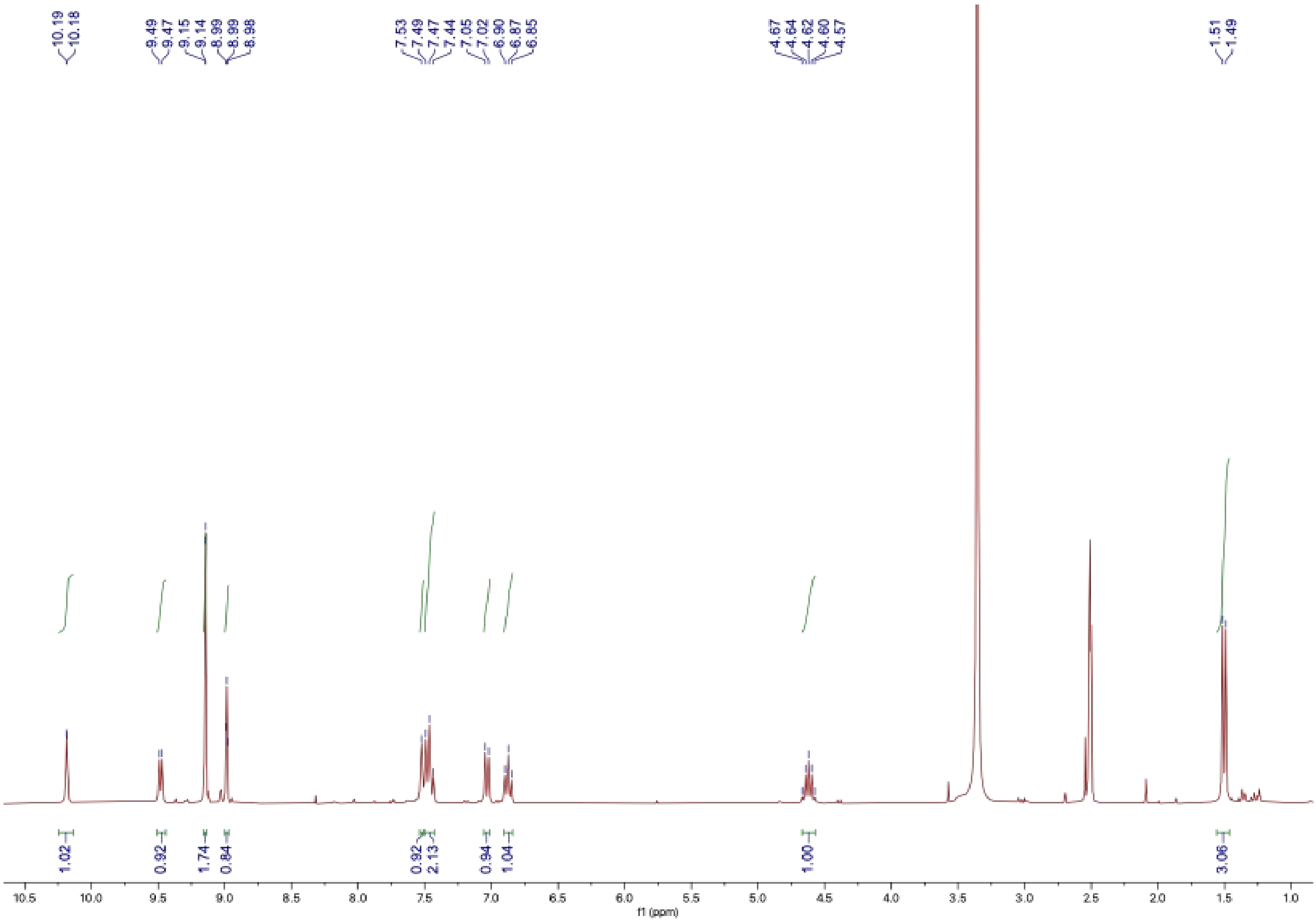

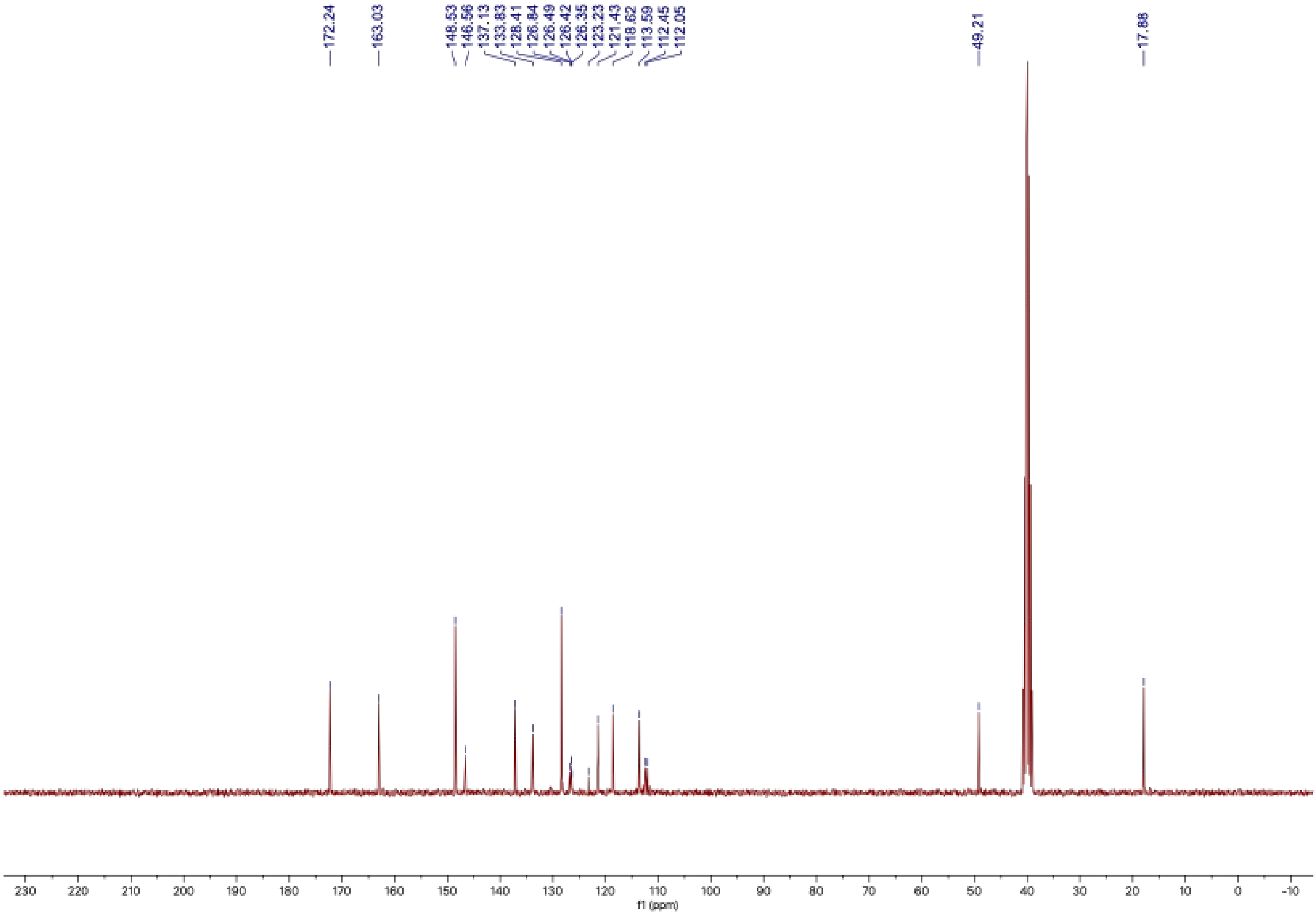

(S)-*N*-(1-(2-(3-bromophenyl)hydrazineyl)-1-oxopropan-2-yl)-3,5-dinitrobenzamide, 21

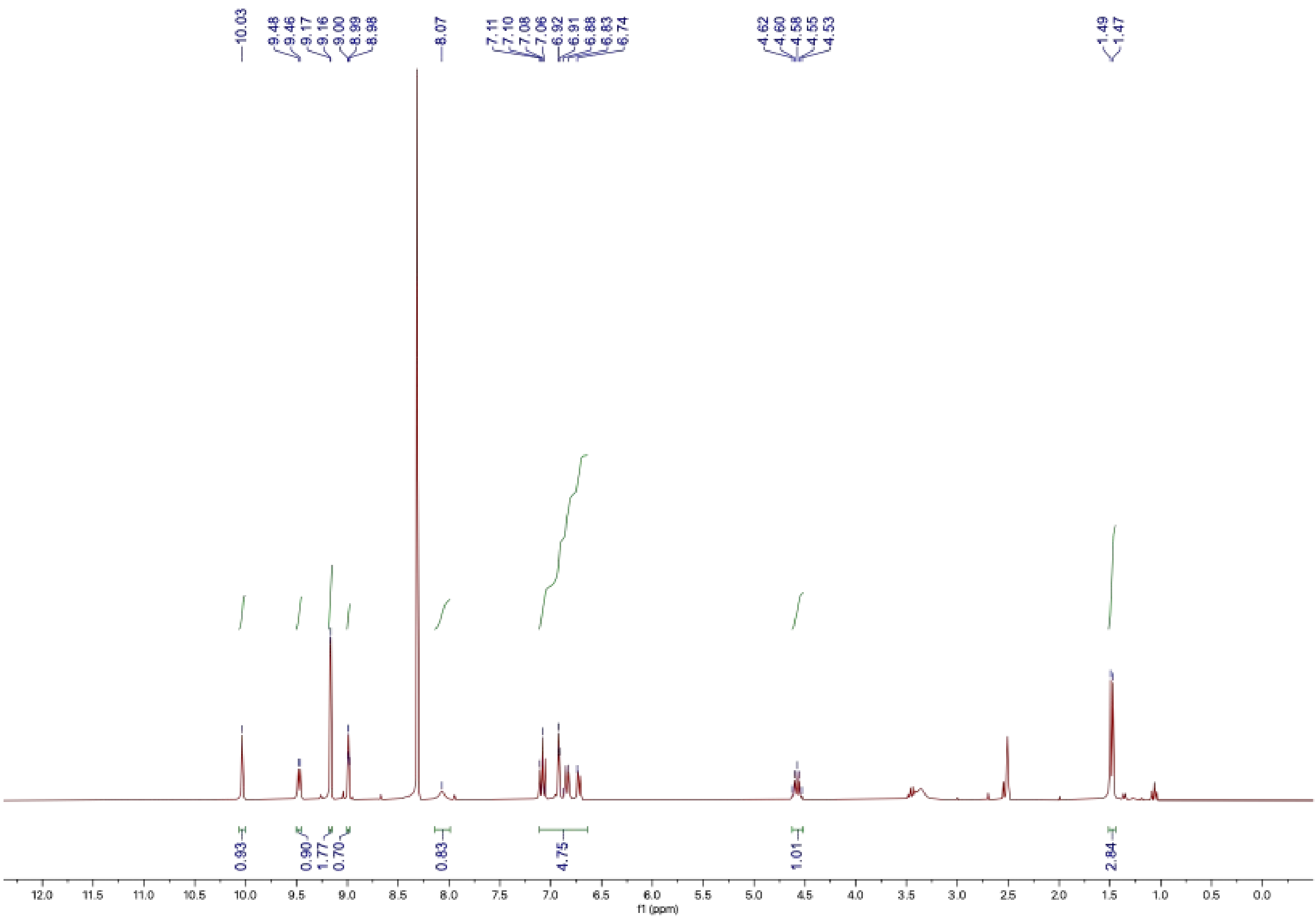

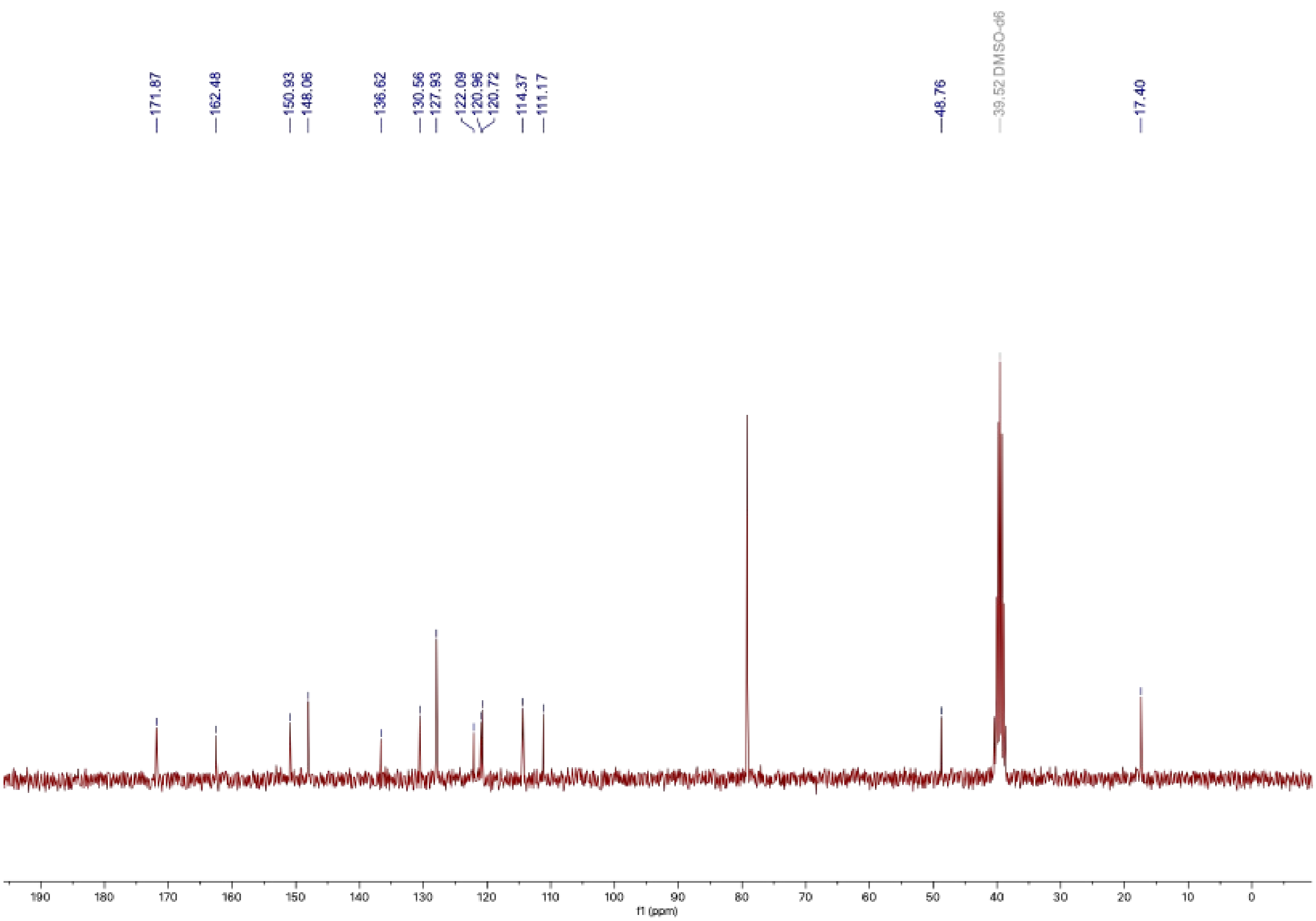

(S)-3,5-dinitro-*N*-(1-oxo-1-(2-(3-(trifluoromethyl)phenyl)hydrazineyl)propan-2-yl)benzamide, 22

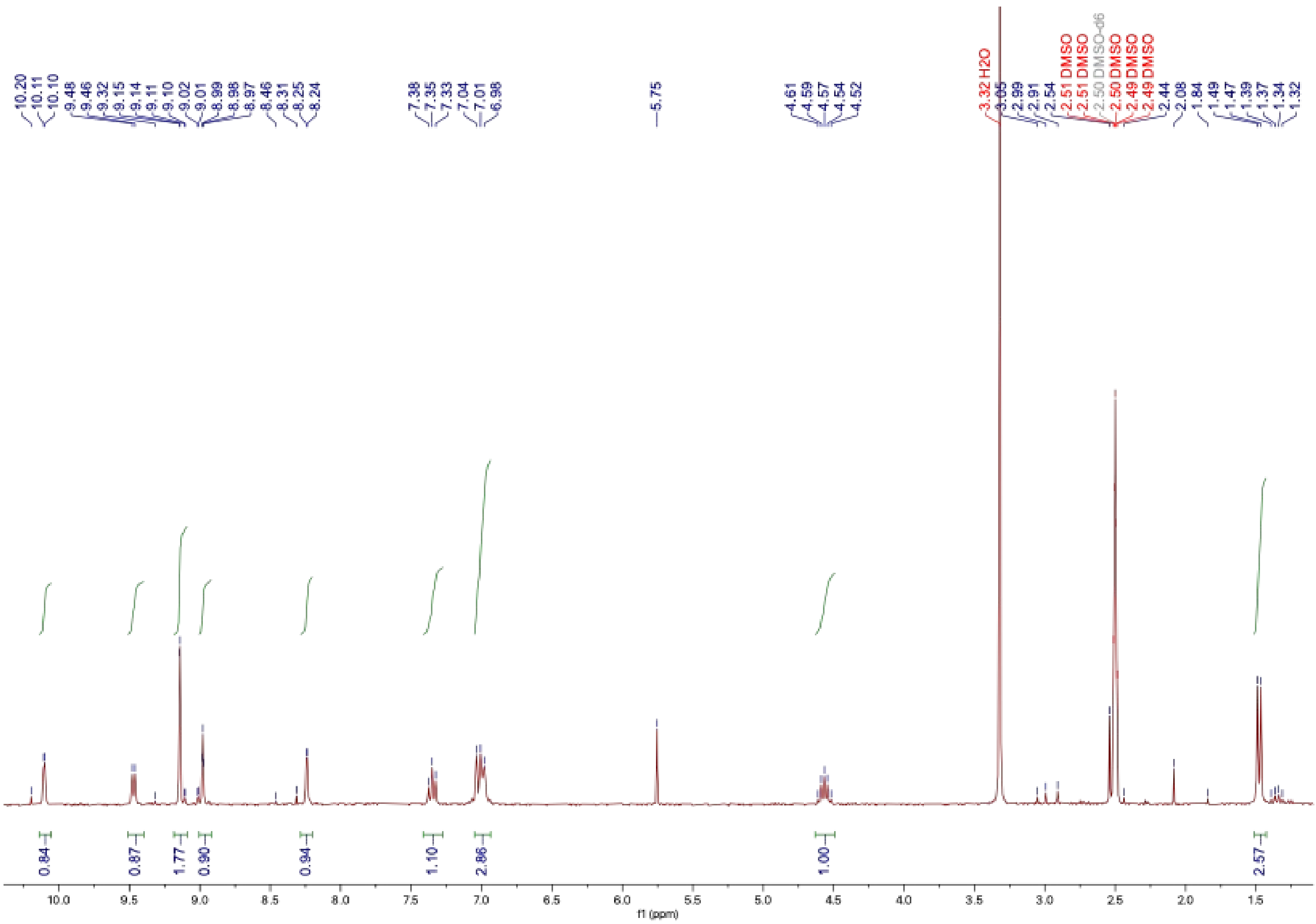

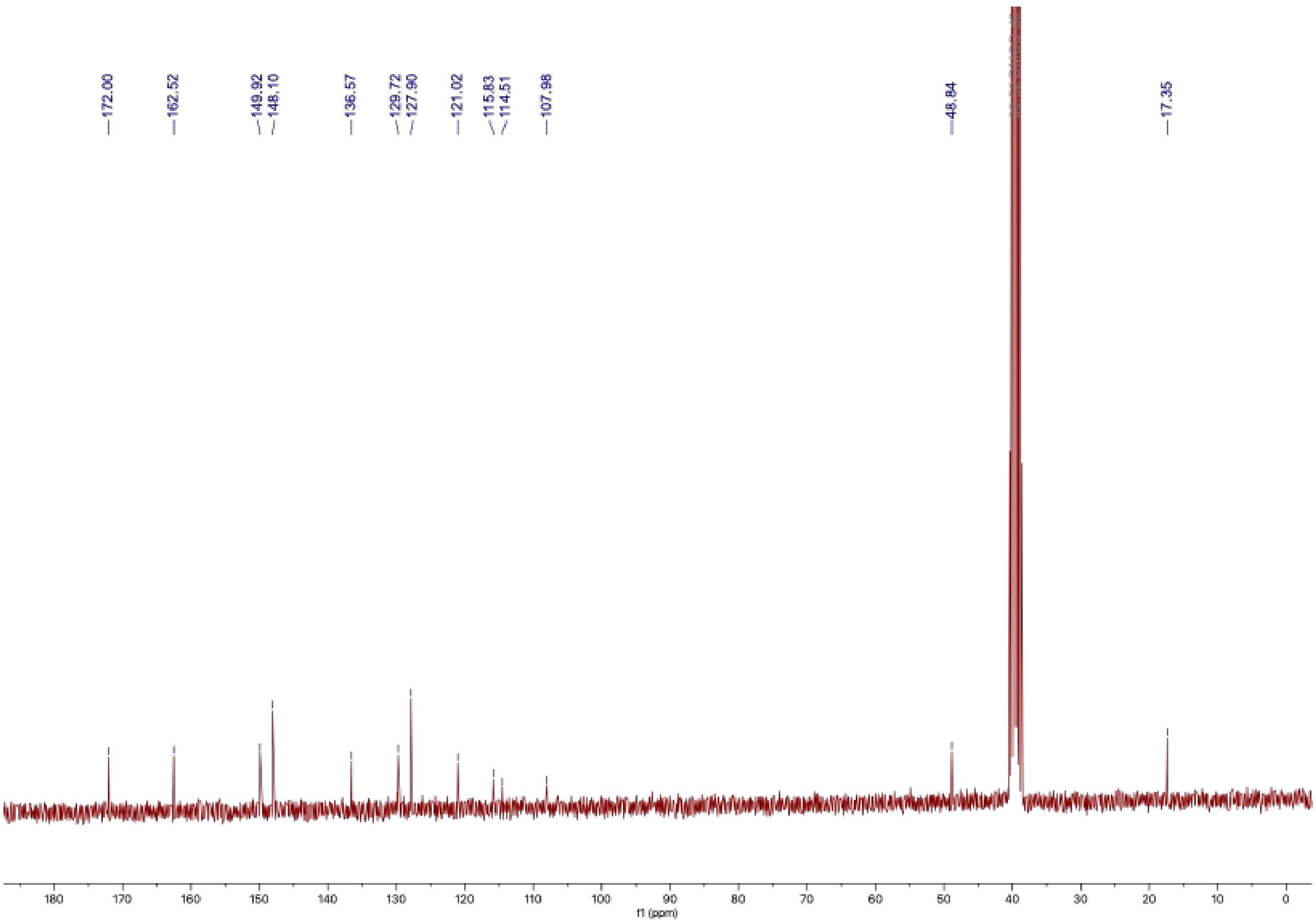

(S)-*N*-(1-(2-(4-chlorophenyl)hydrazineyl)-1-oxopropan-2-yl)-3,5-dinitrobenzamide, 23

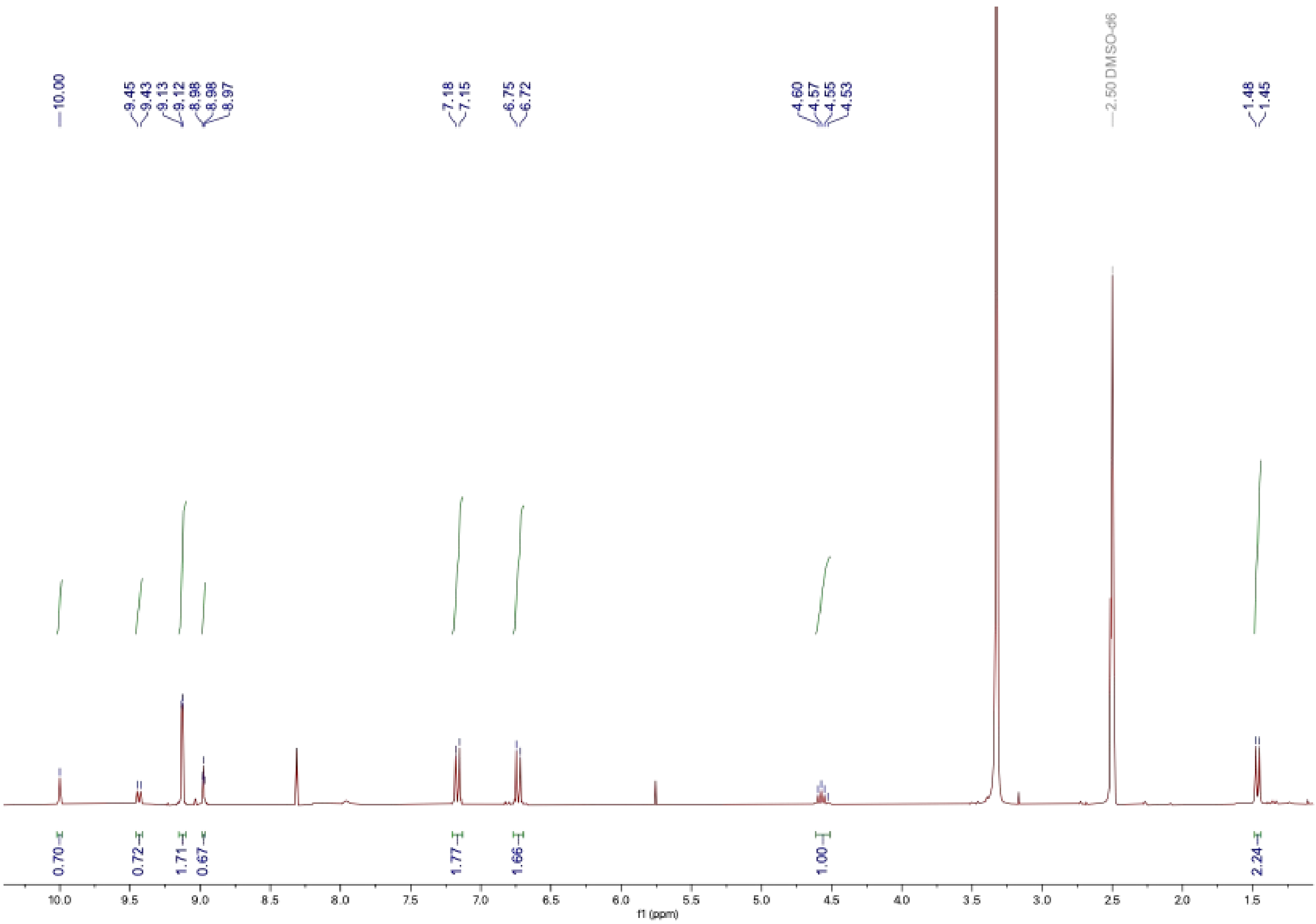

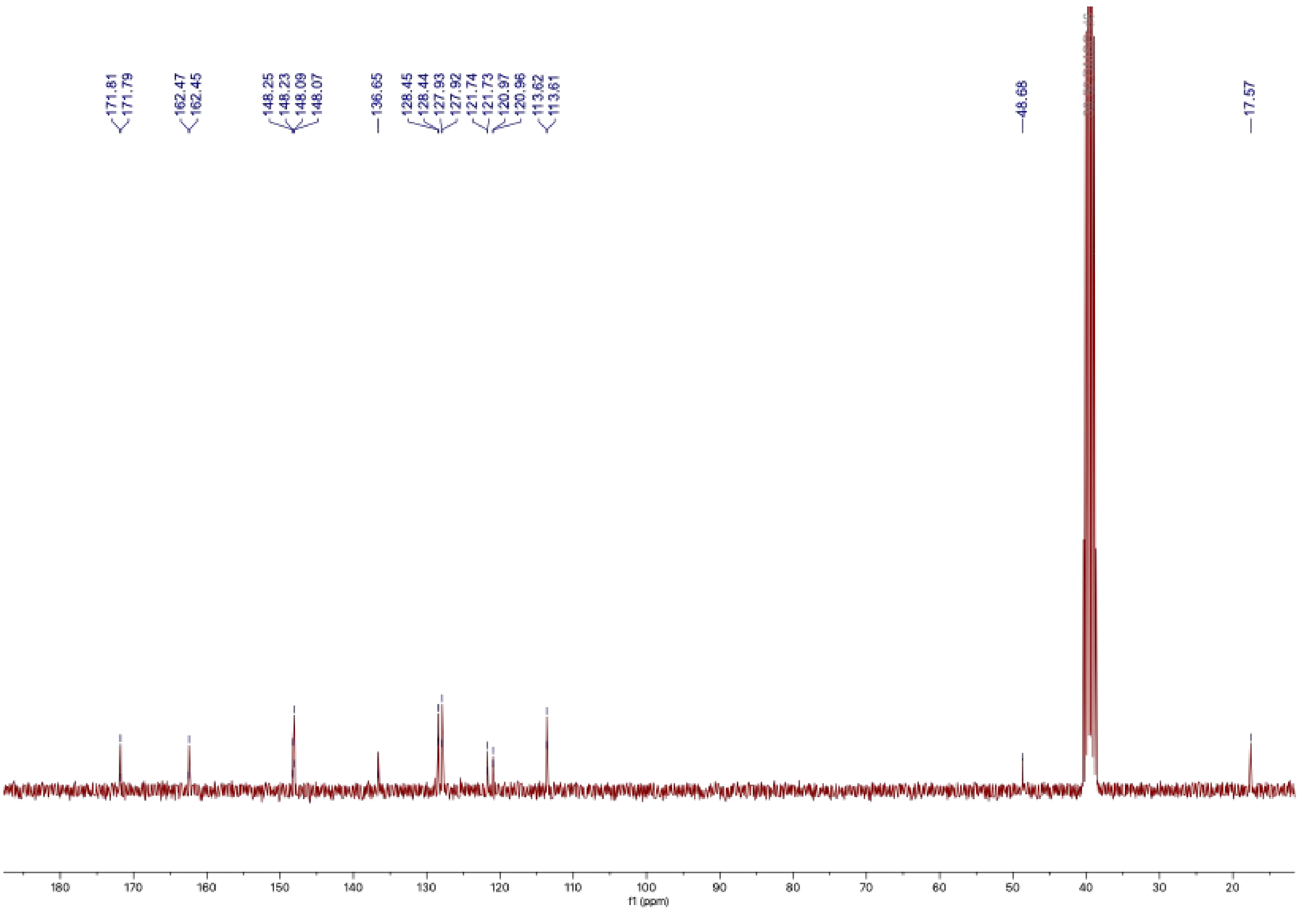

(S)-*N*-(1-(2-(4-bromophenyl)hydrazineyl)-1-oxopropan-2-yl)-3,5-dinitrobenzamide, 24

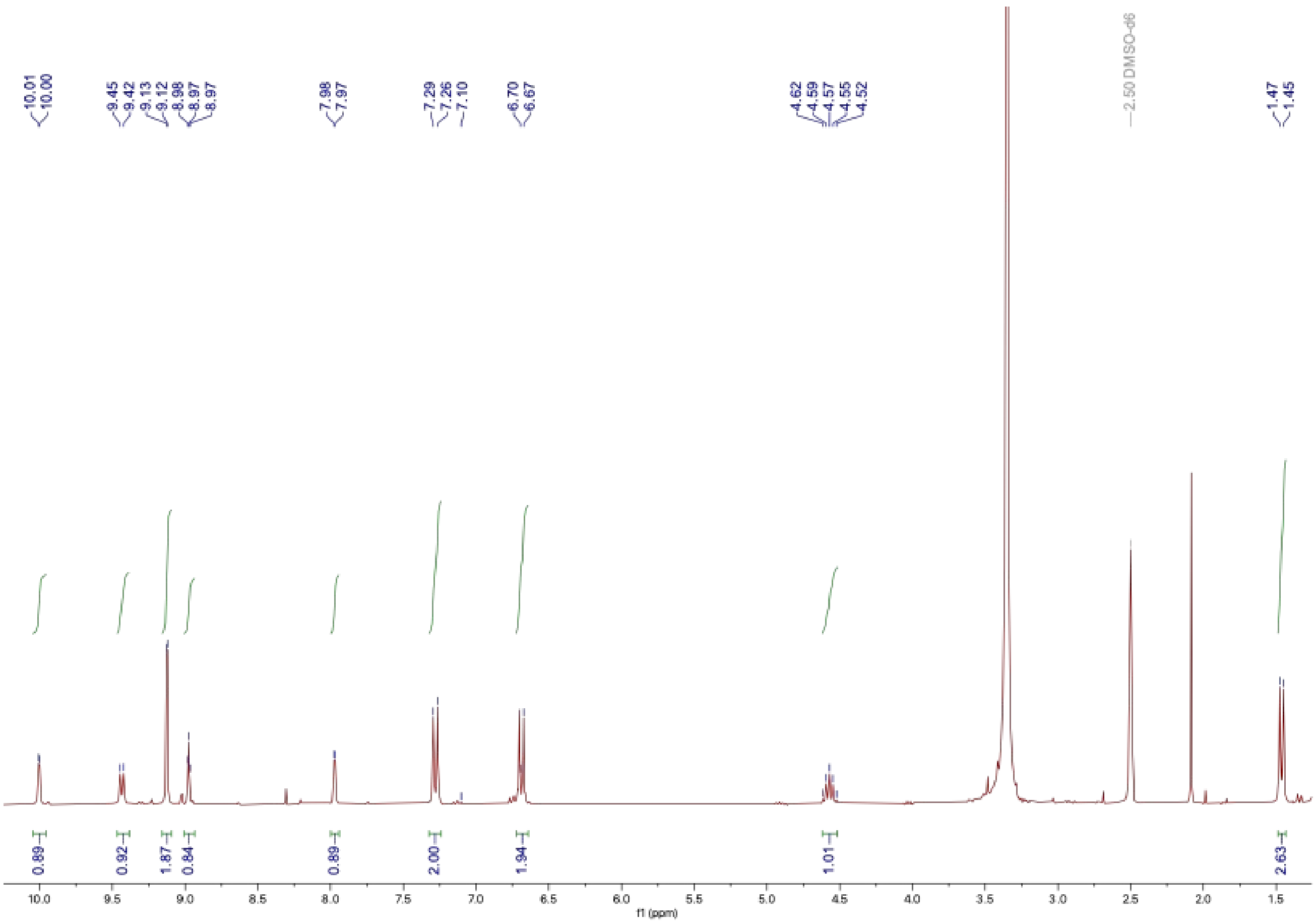

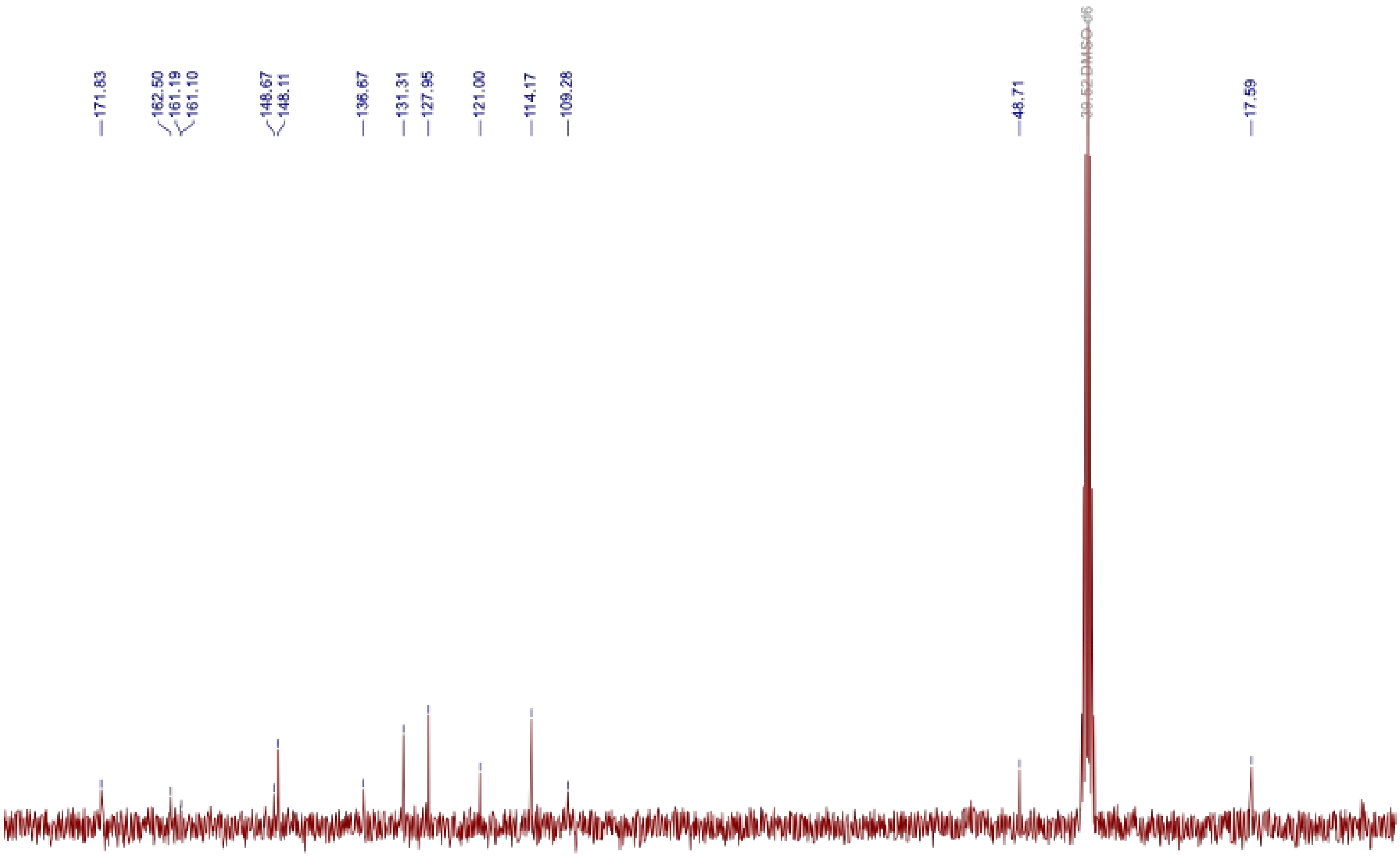

(S)-*N*-(1-(2-(2-bromophenyl)hydrazineyl)-4-methyl-1-oxopentan-2-yl)-3,5-dinitrobenzamide, 25

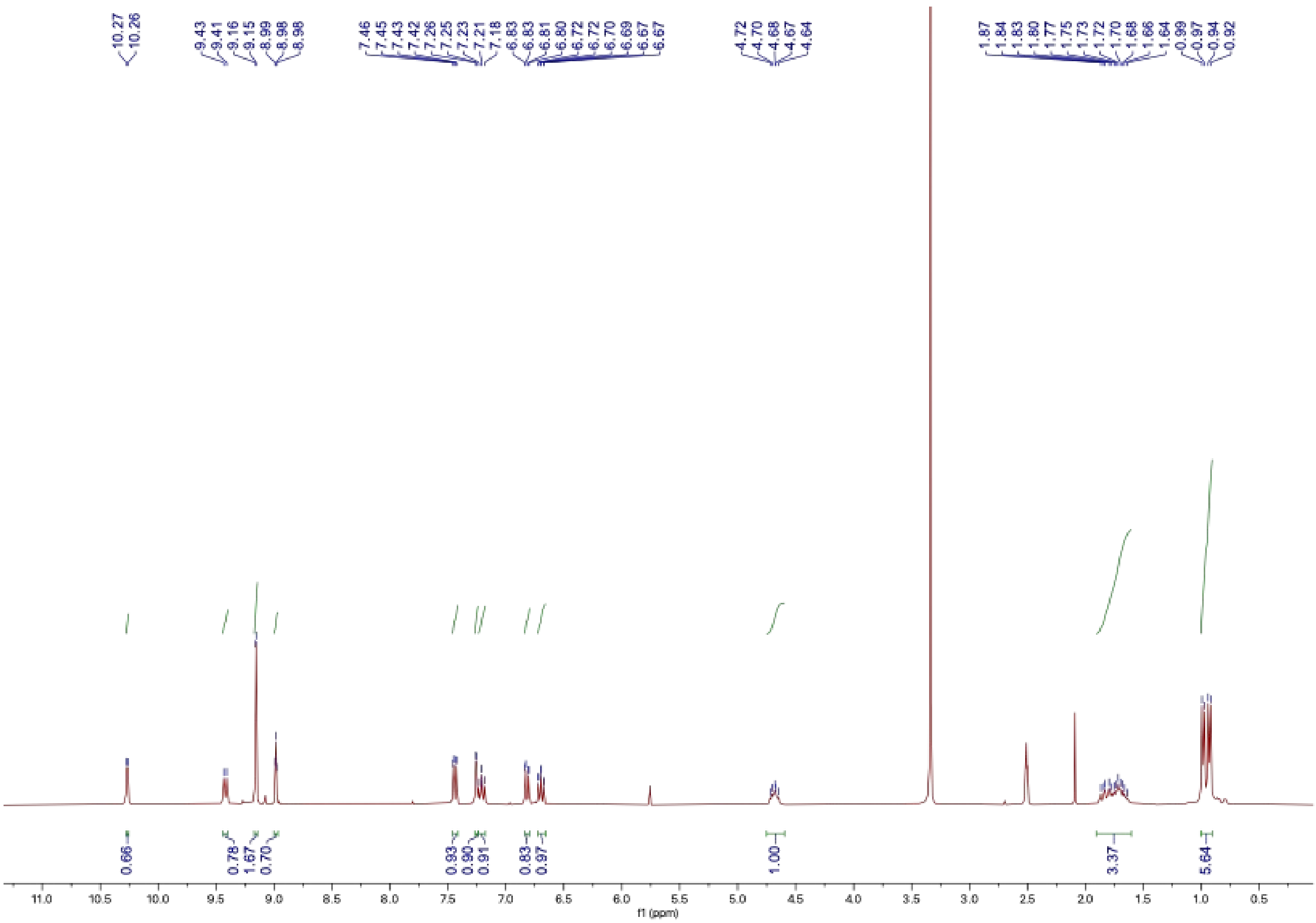

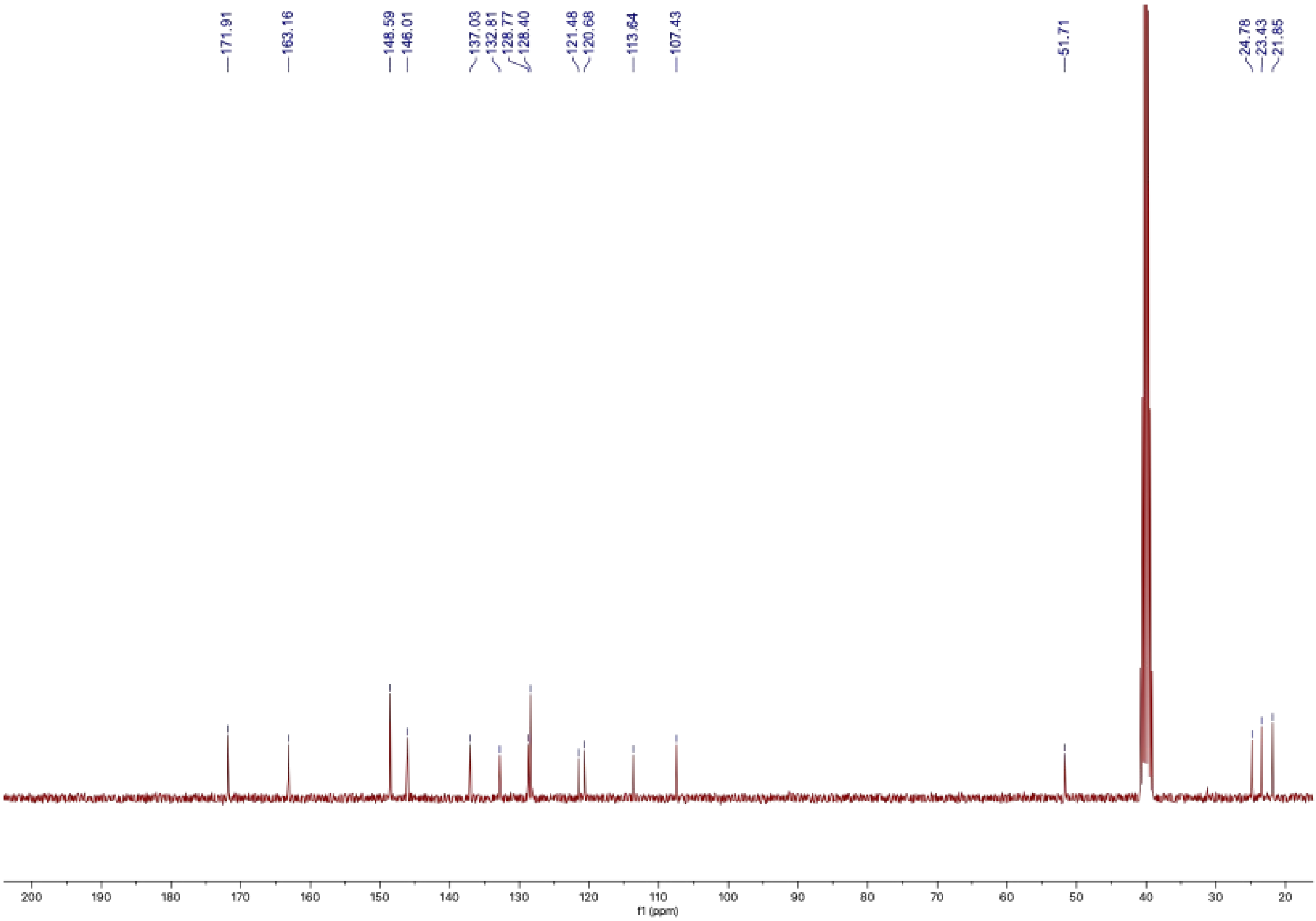

(S)-*N*-(4-methyl-1-oxo-1-(2-(2-(trifluoromethyl)phenyl)hydrazineyl)pentan-2-yl)-3,5-dinitrobenzamide, 26

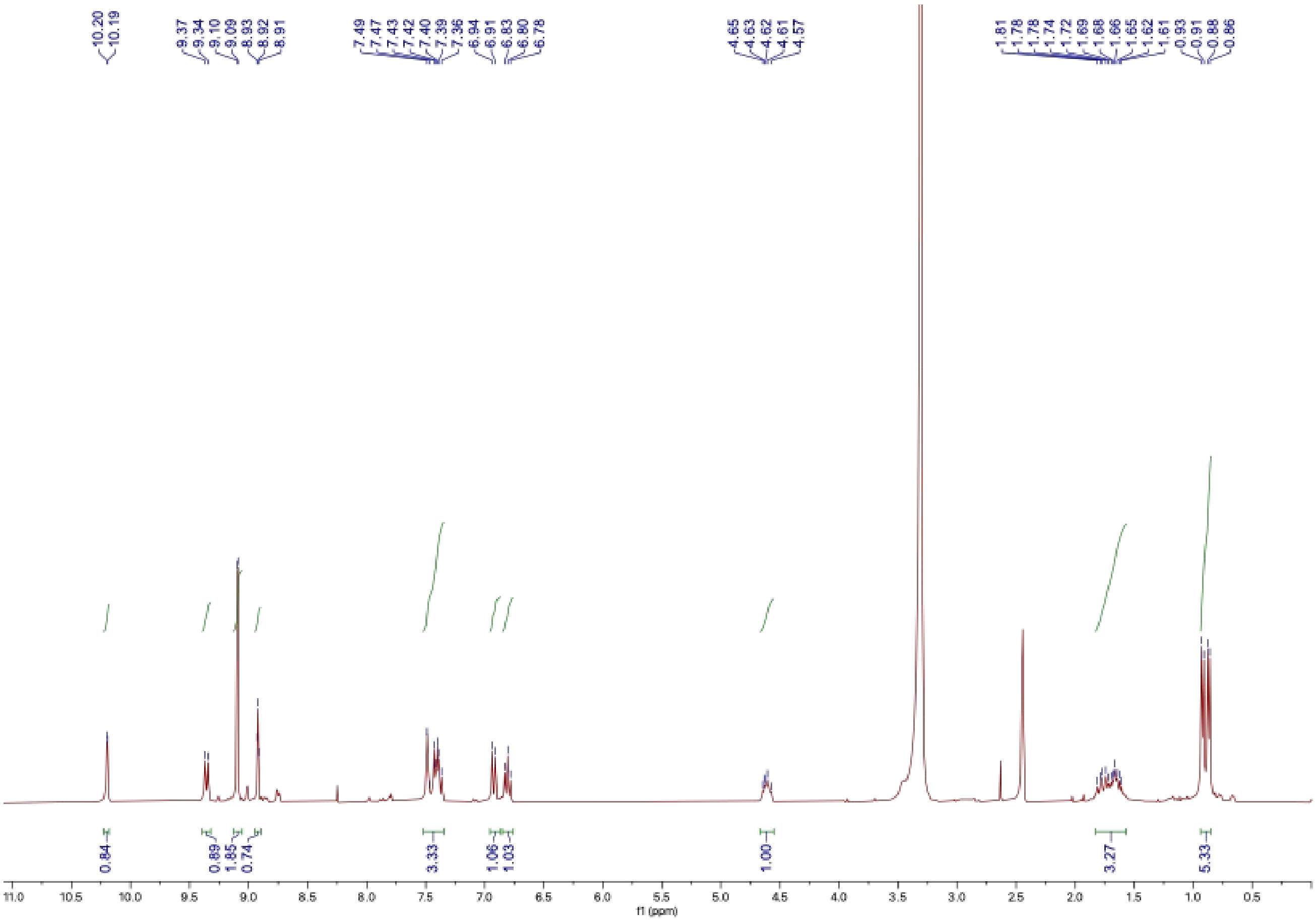

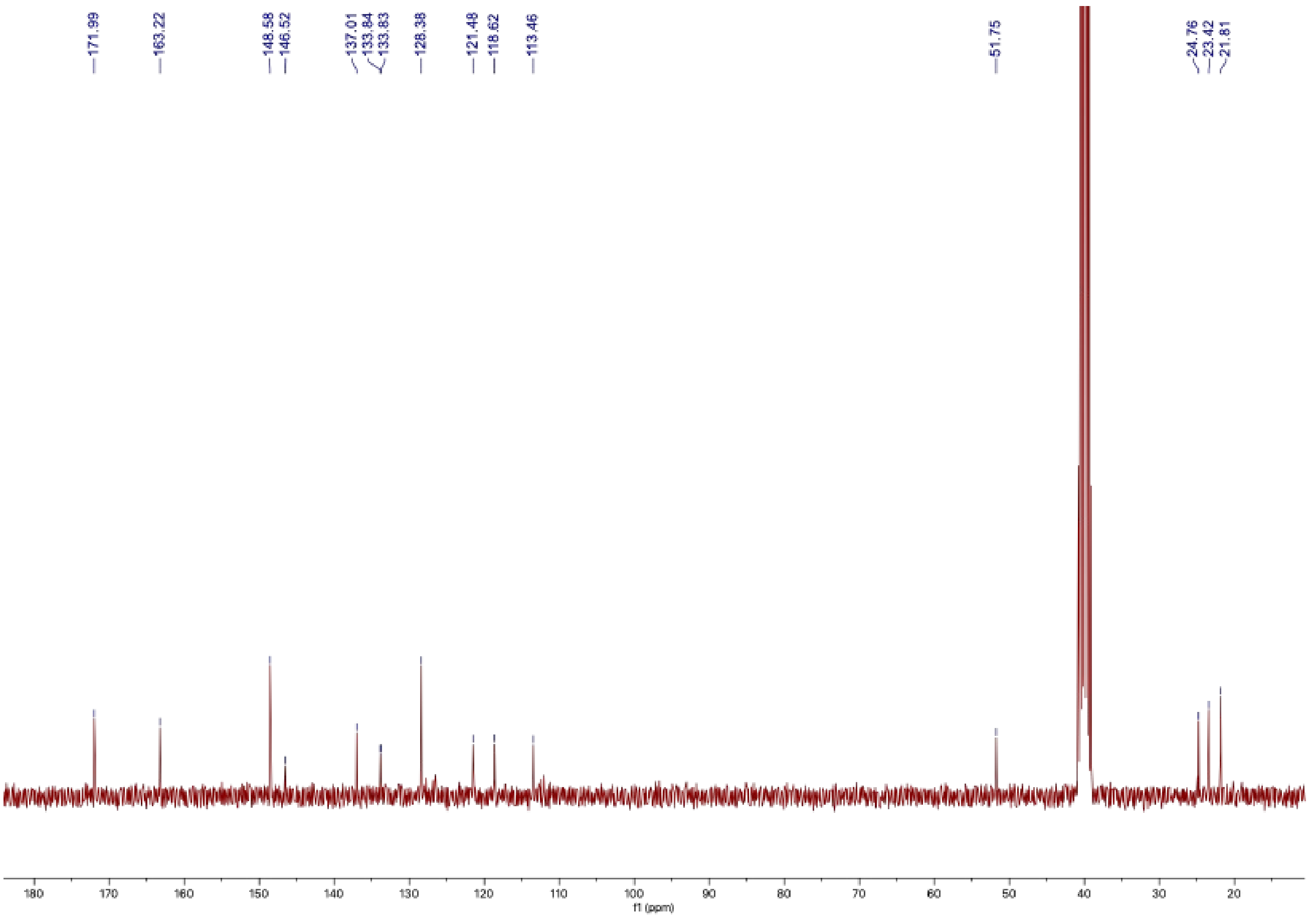

(S)-N-(1-(2-(3-chlorophenyl)hydrazineyl)-4-methyl-1-oxopentan-2-yl)-3,5-dinitrobenzamide, 27

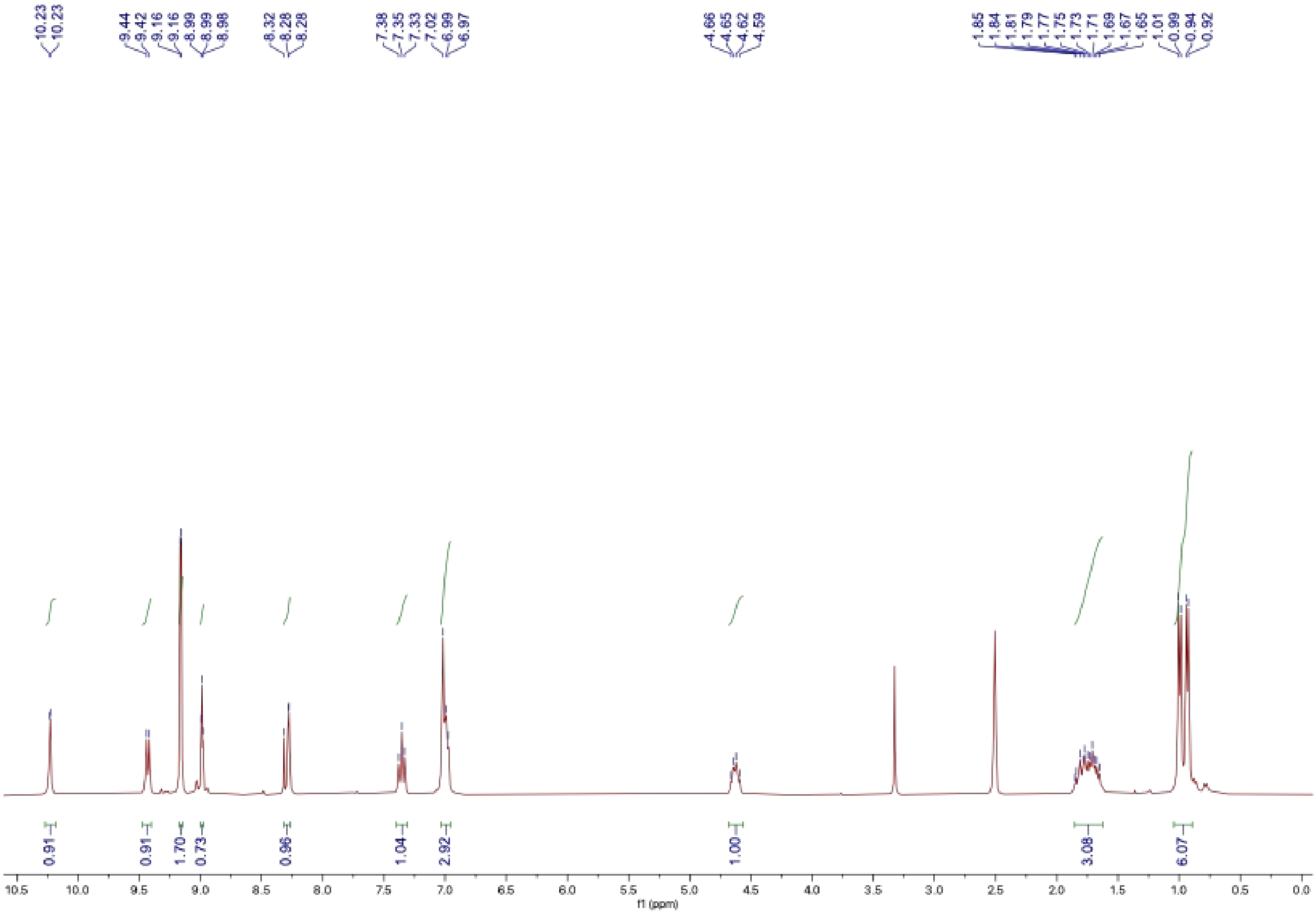

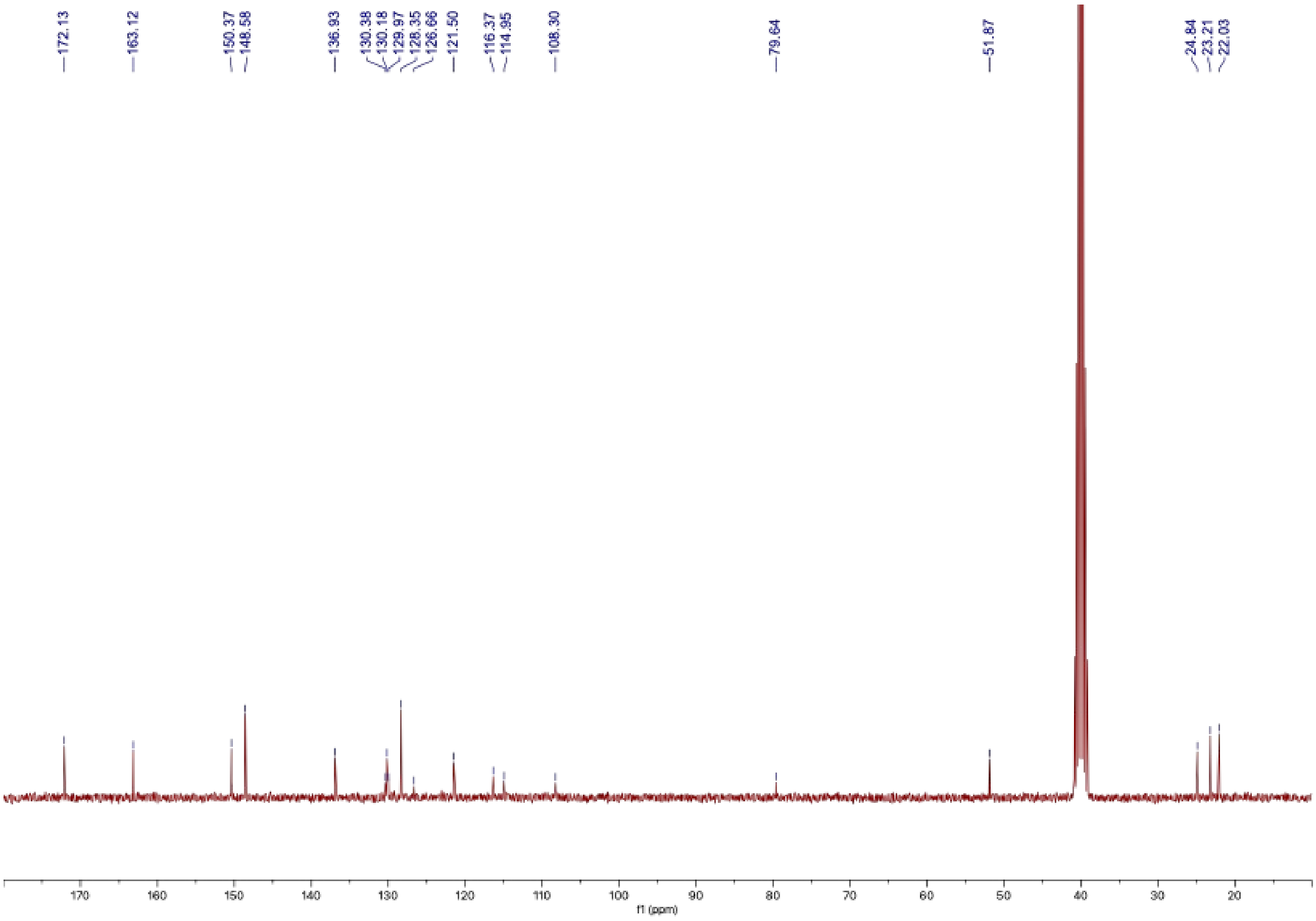

(S)-*N*-(1-(2-(3-bromophenyl)hydrazineyl)-4-methyl-1-oxopentan-2-yl)-3,5-dinitrobenzamide, 28

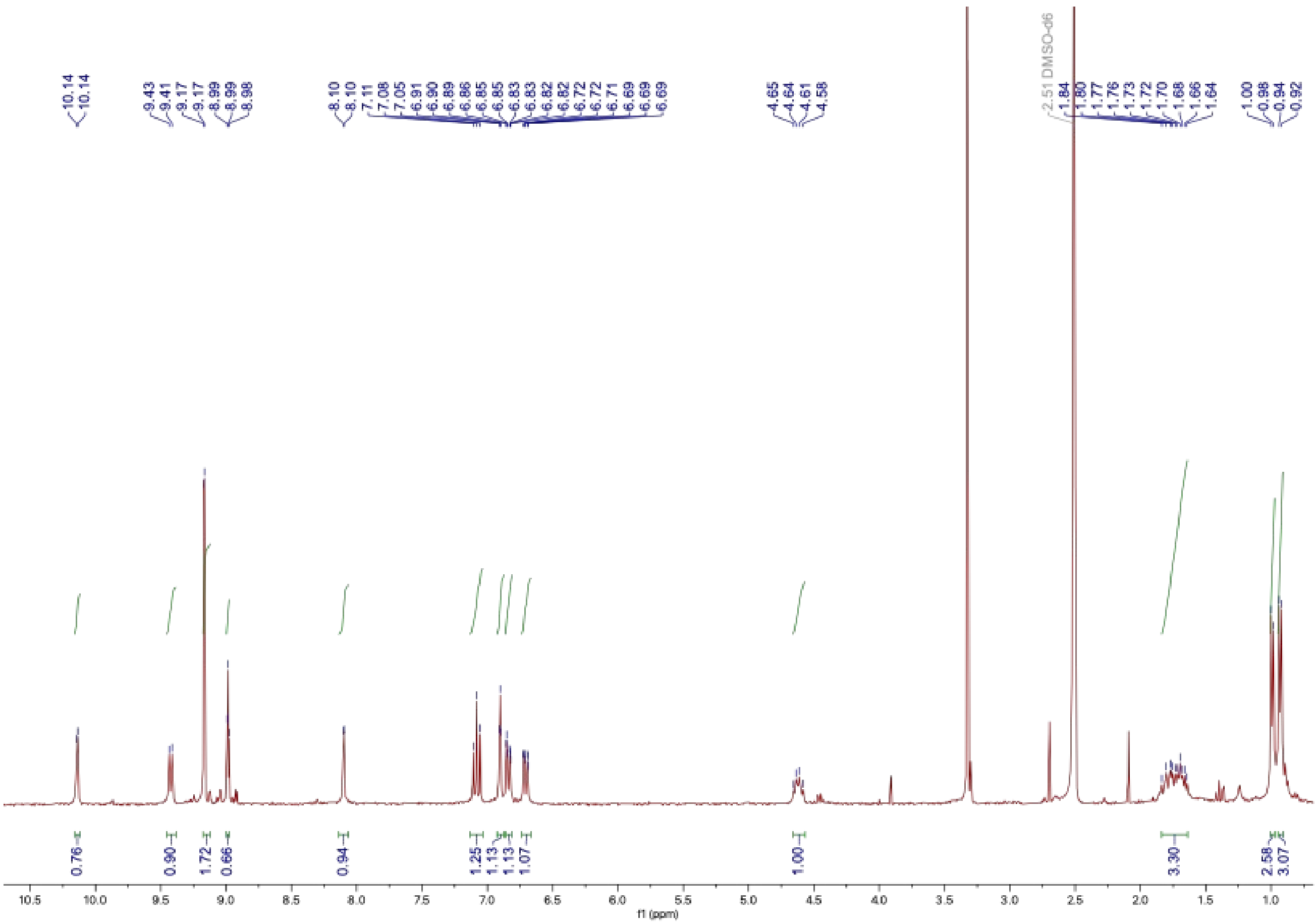

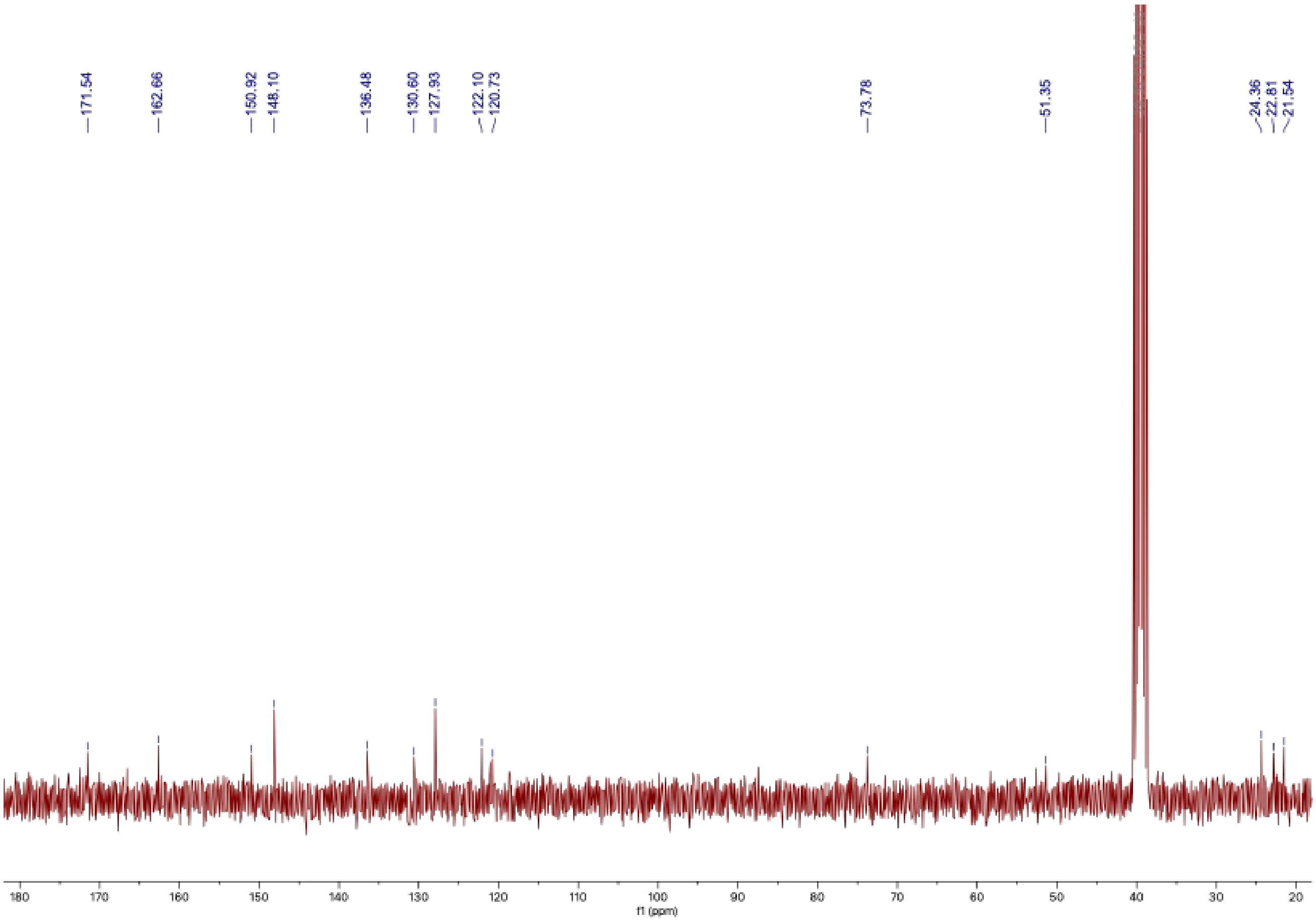

(S)-*N*-(4-methyl-1-oxo-1-(2-(3-(trifluoromethyl)phenyl)hydrazineyl)pentan-2-yl)-3,5-dinitrobenzamide, 29

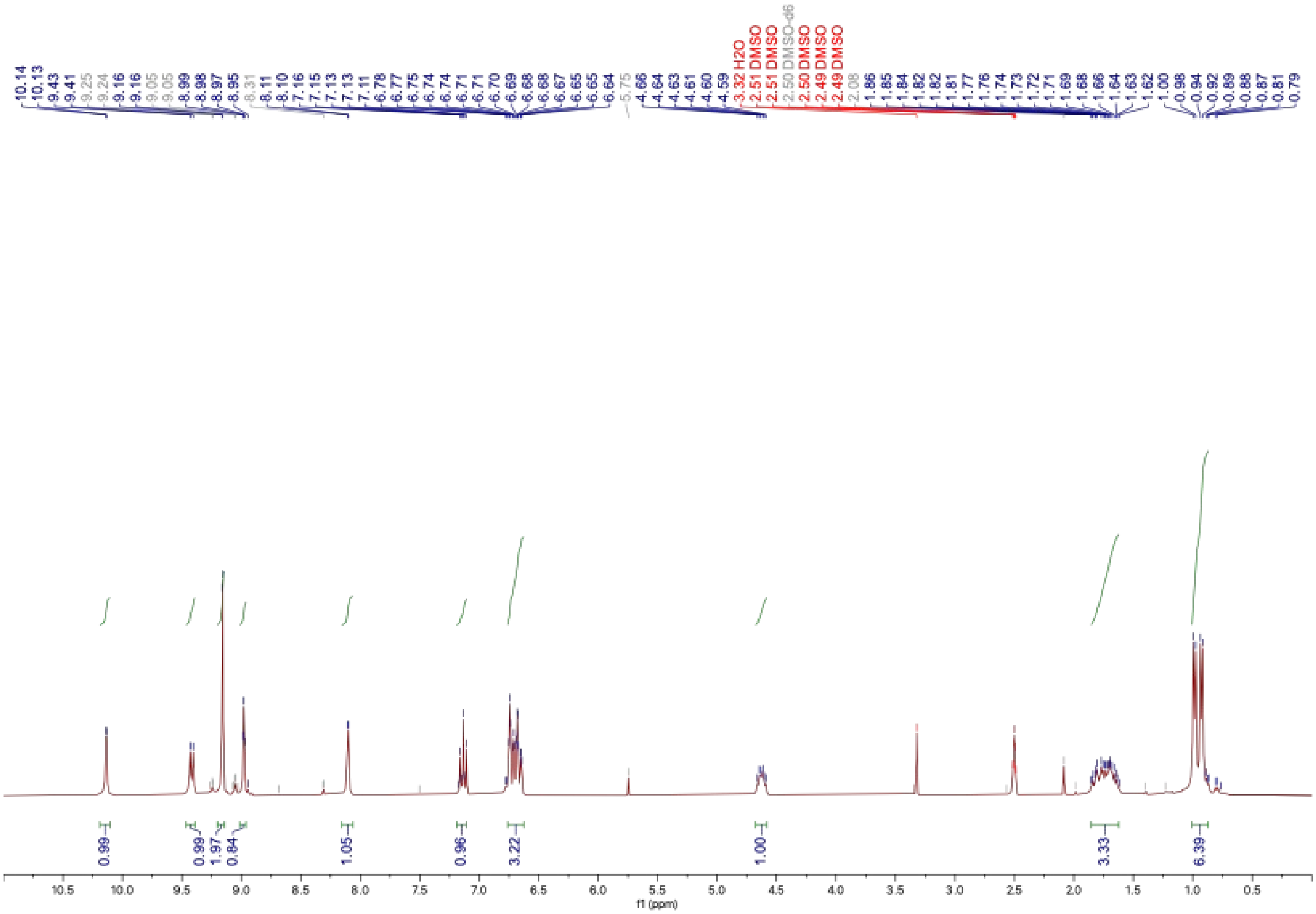

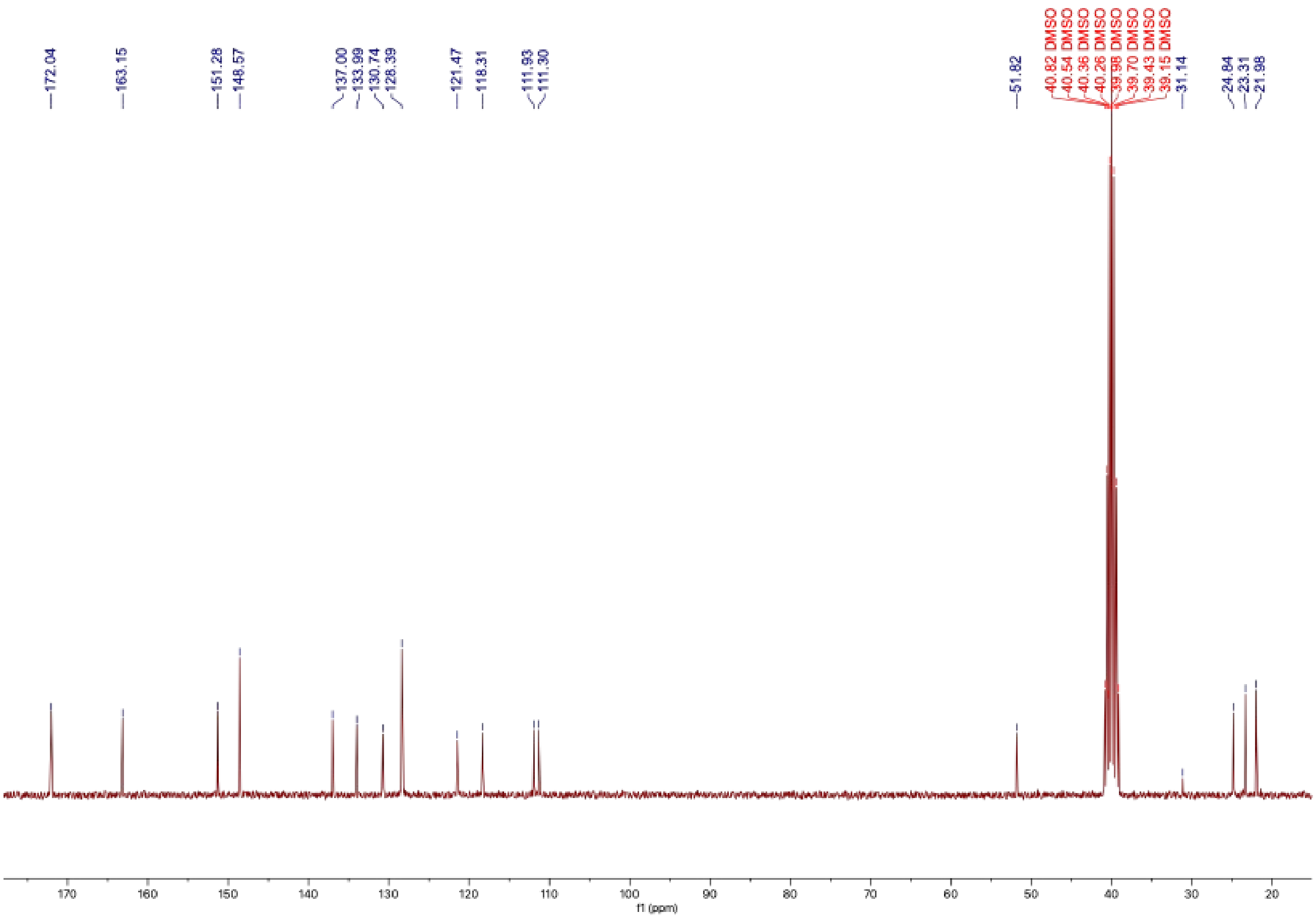

(S)-*N*-(1-(2-(4-chlorophenyl)hydrazineyl)-4-methyl-1-oxopentan-2-yl)-3,5-dinitrobenzamide, 30

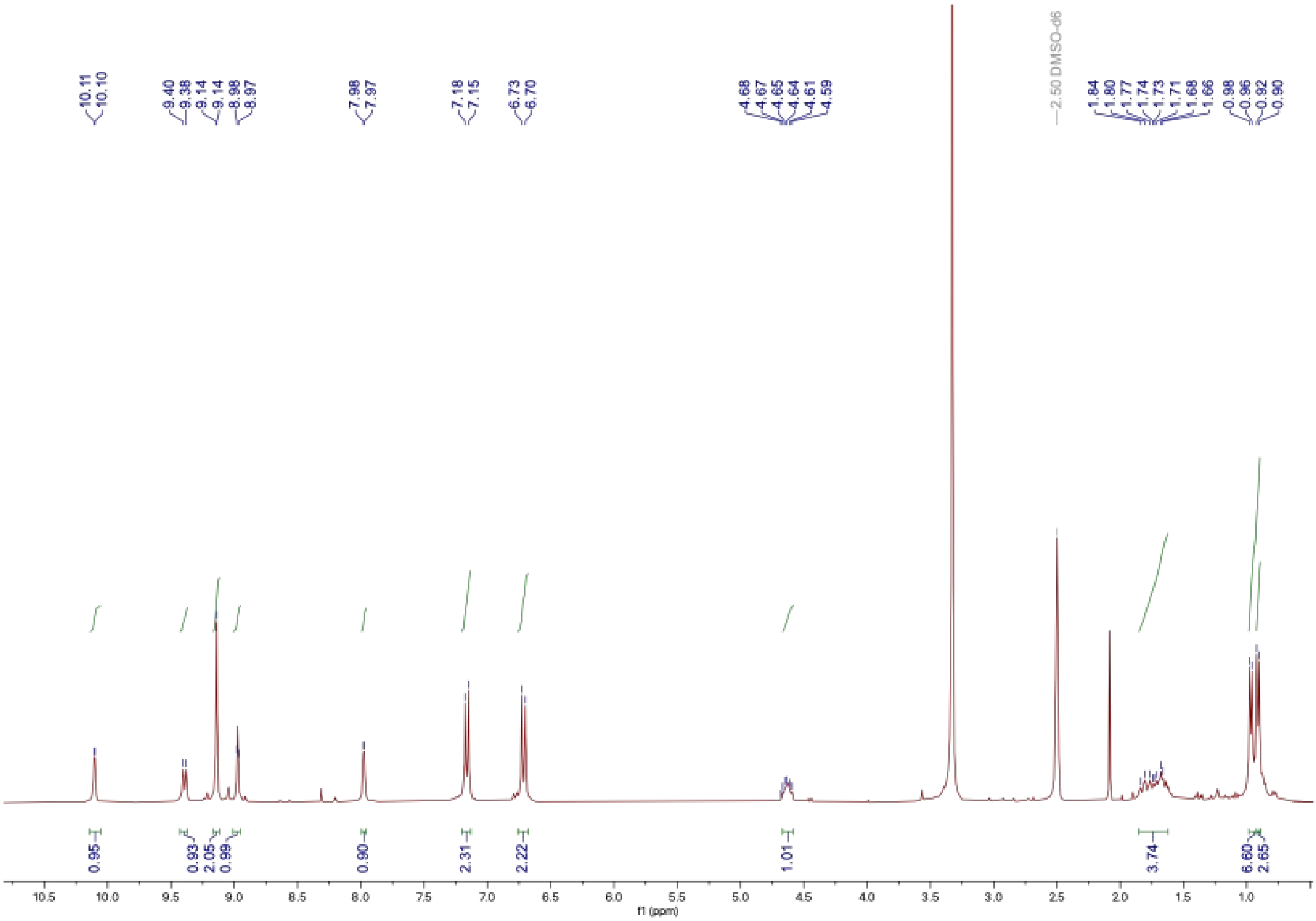

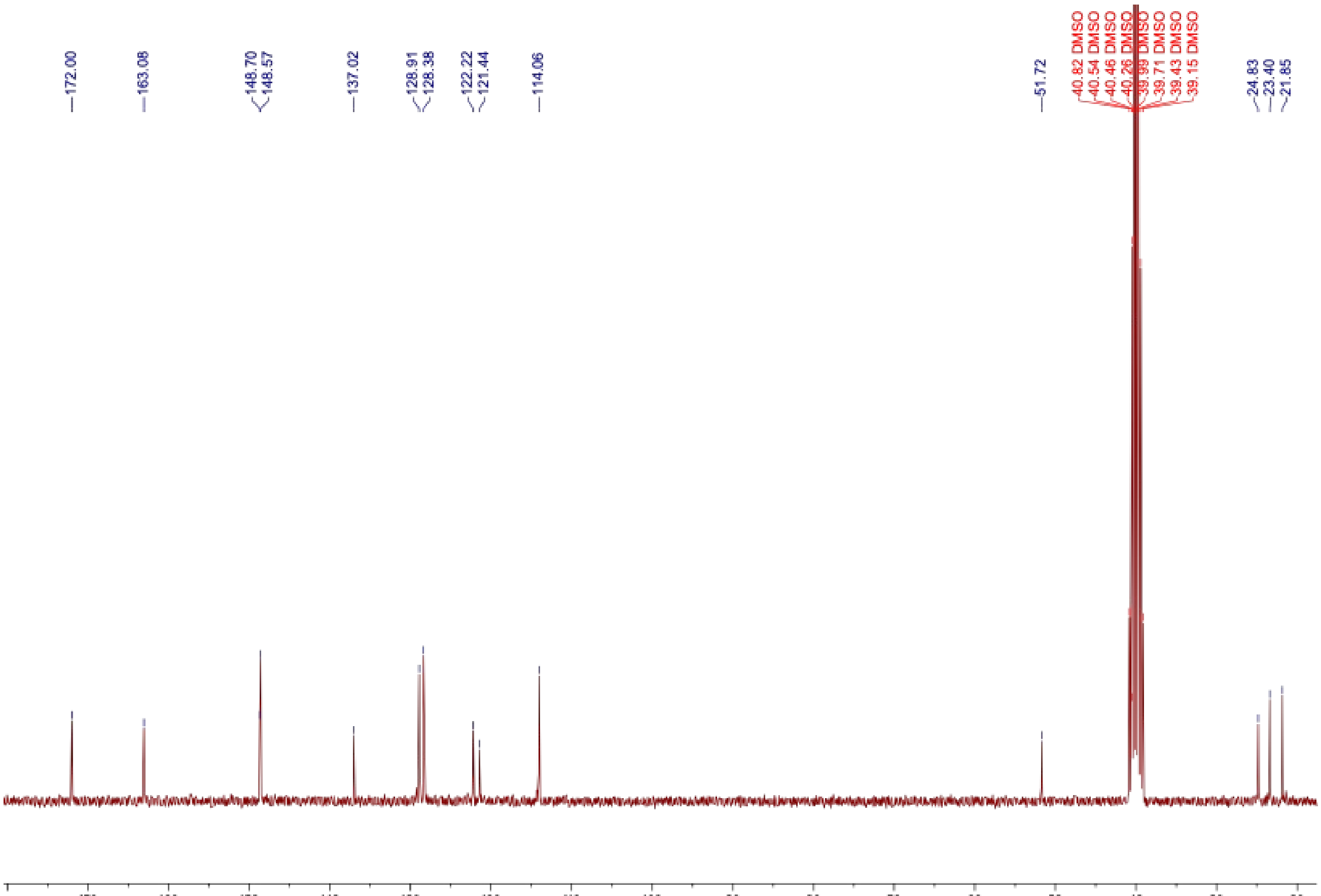

(S)-N-(1-(2-(4-bromophenyl)hydrazineyl)-4-methyl-1-oxopentan-2-yl)-3,5-dinitrobenzamide, 31

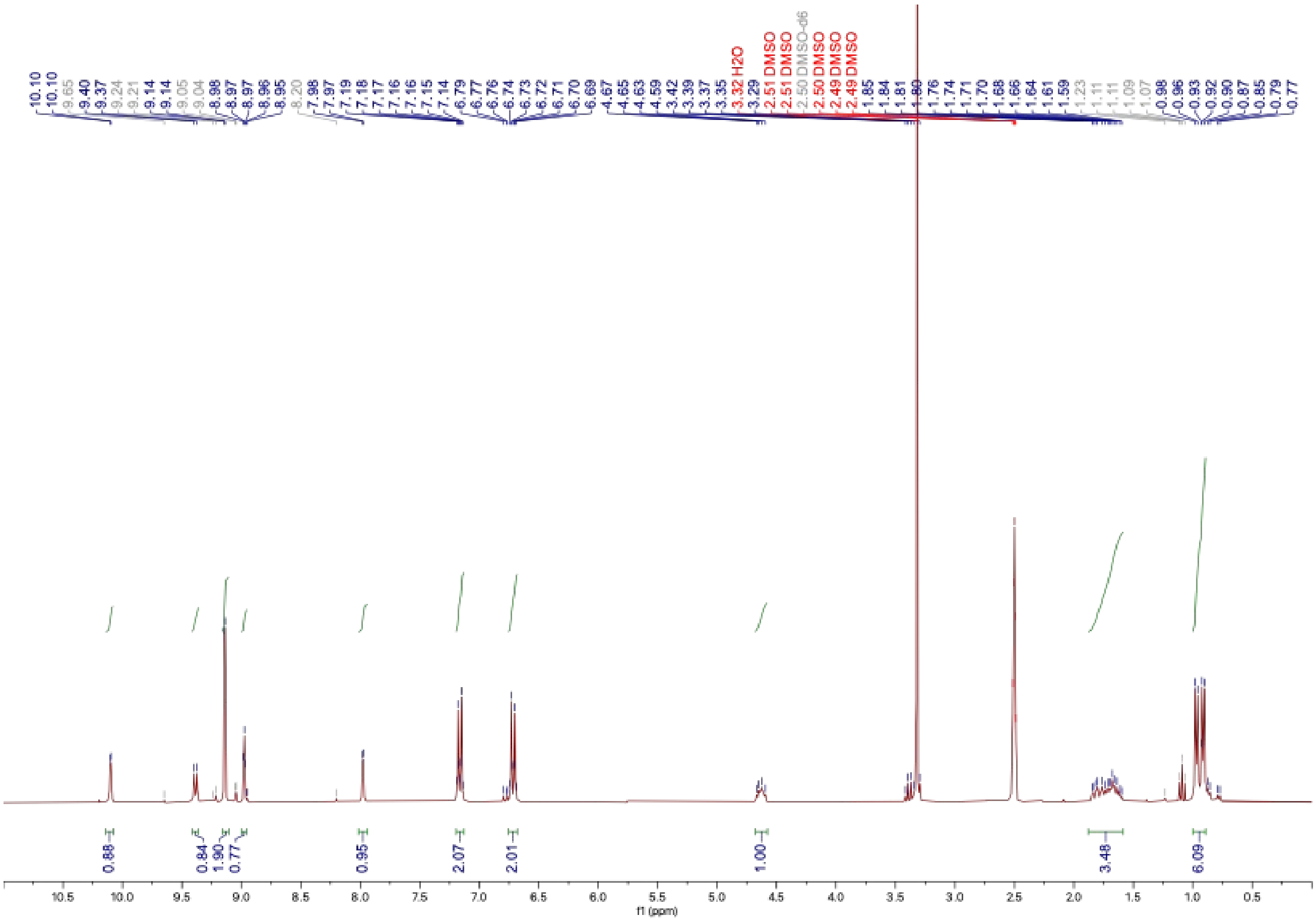

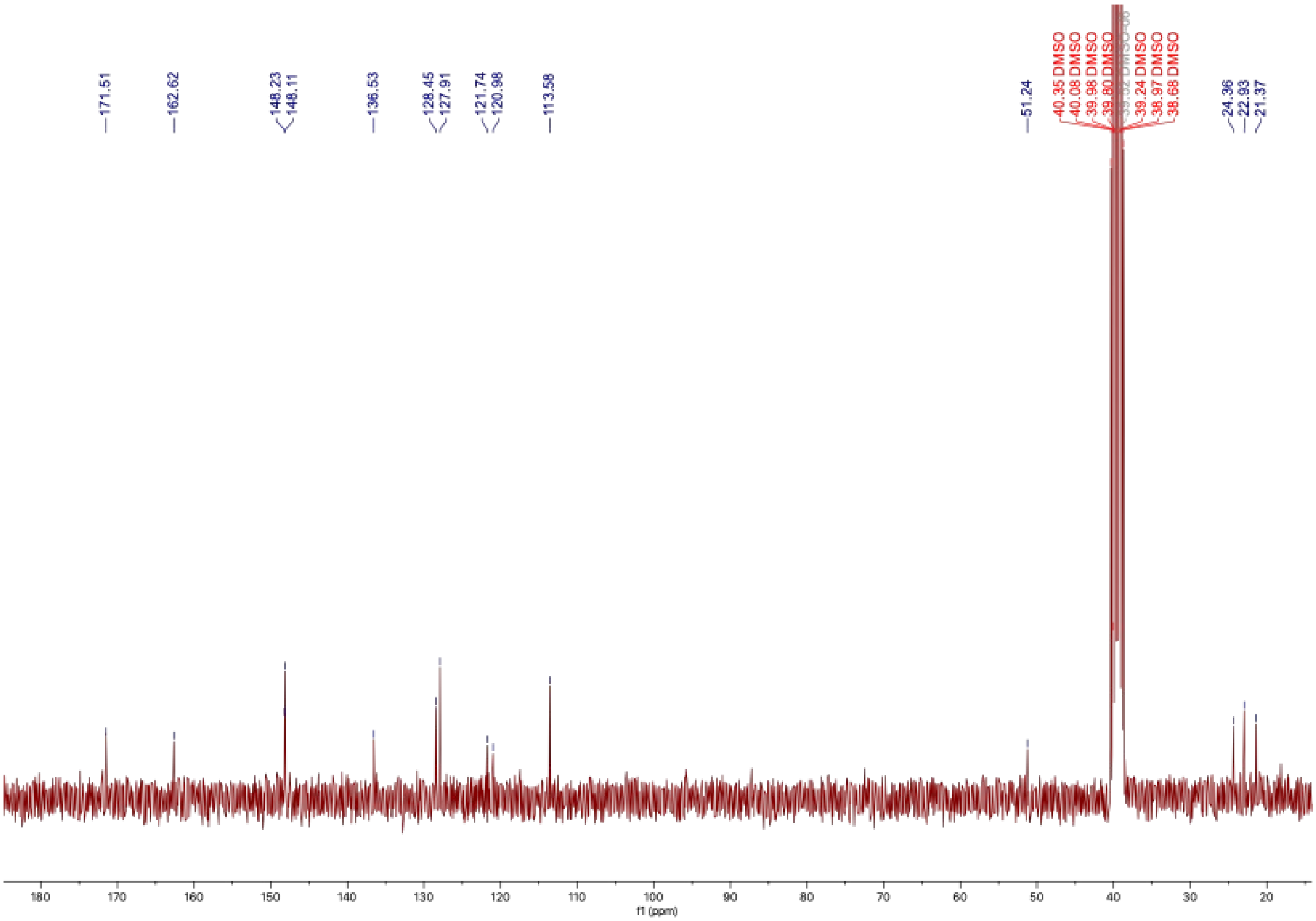

(S)-*N*-(4-methyl-1-oxo-1-(2-(4-(trifluoromethyl)phenyl)hydrazineyl)pentan-2-yl)-3,5-dinitrobenzamide, 32

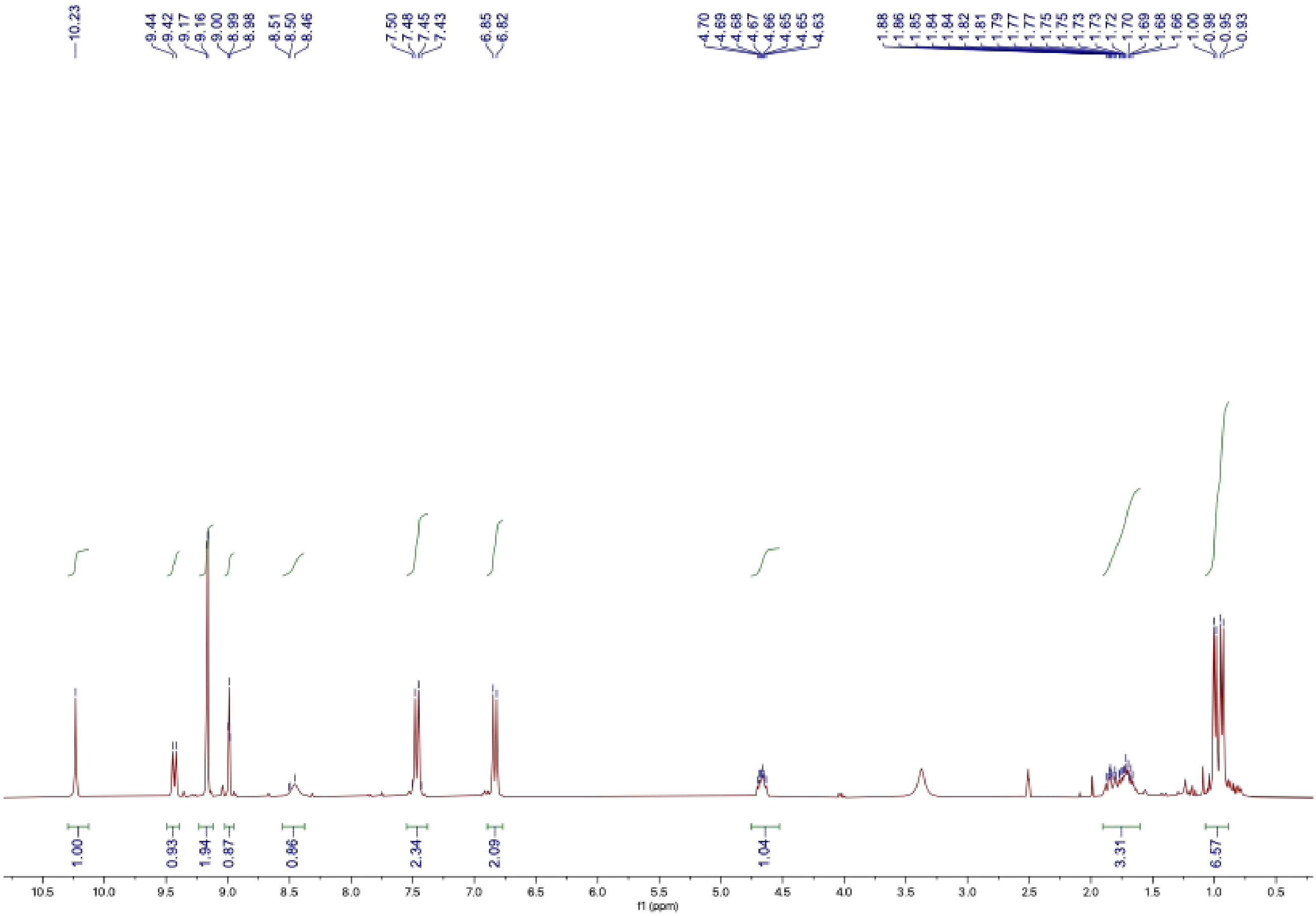

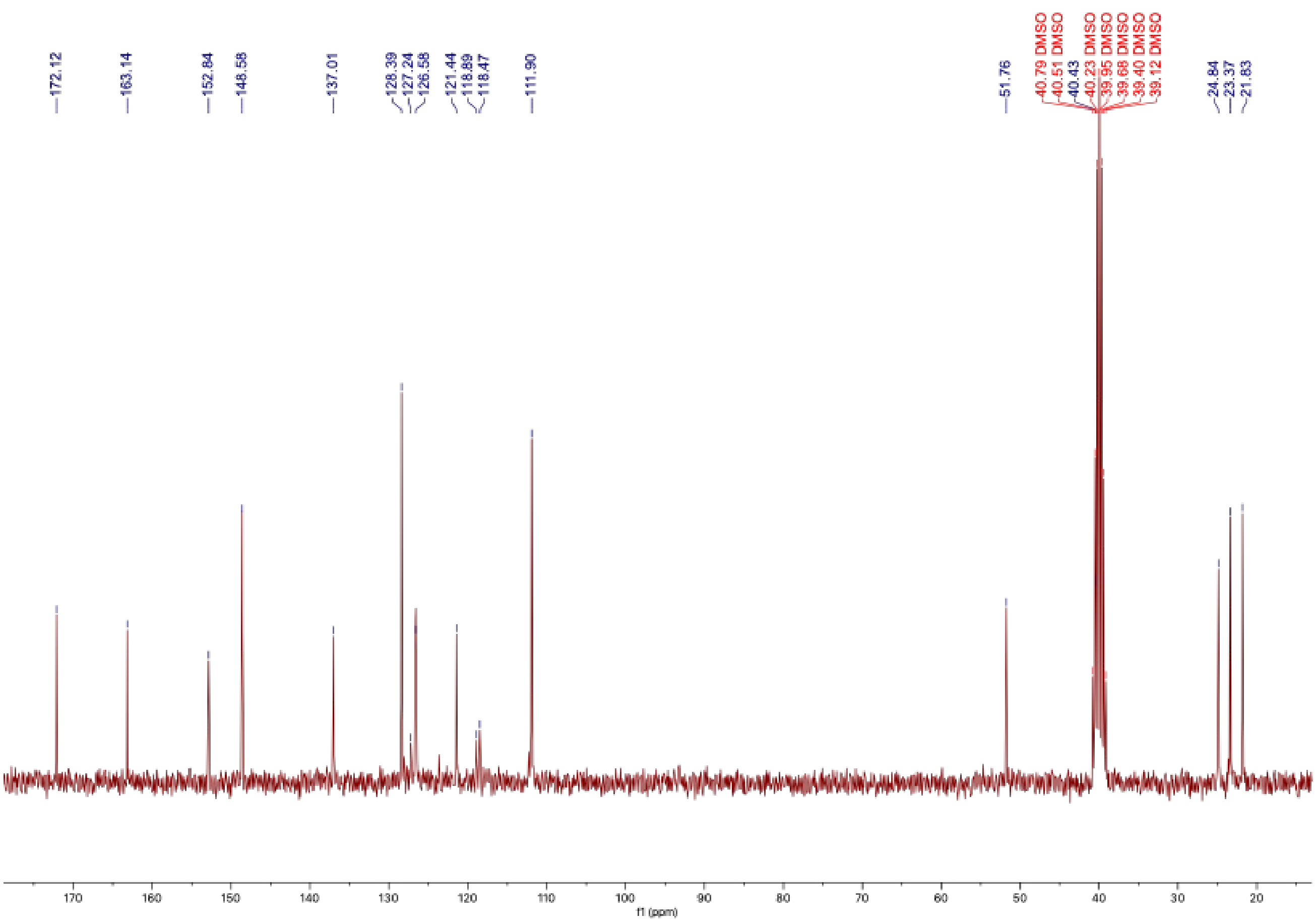

(S)-*N*-(1-(2-(2-bromophenyl)hydrazineyl)-1-oxo-3-phenylpropan-2-yl)-3,5-dinitrobenzamide, 33

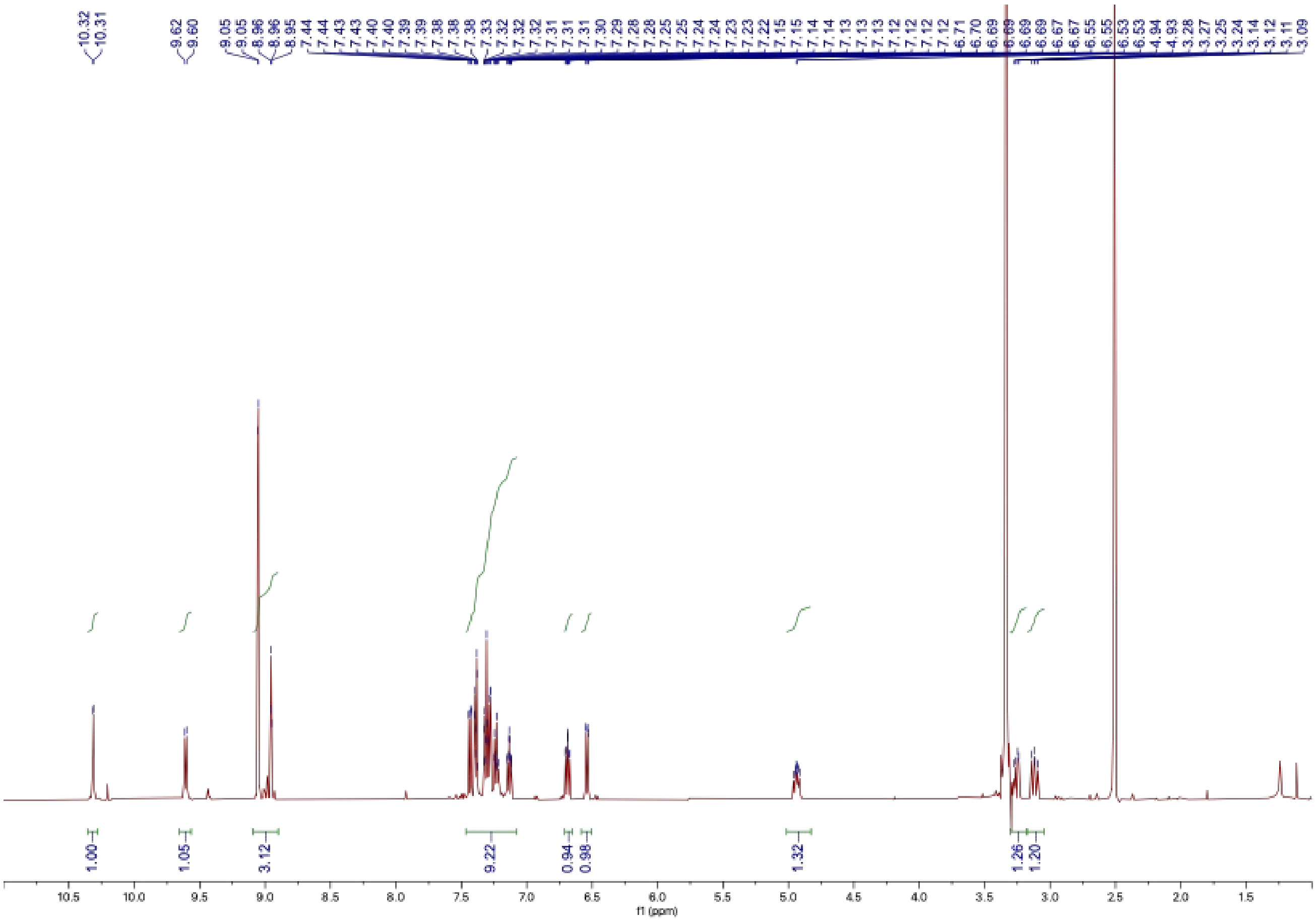

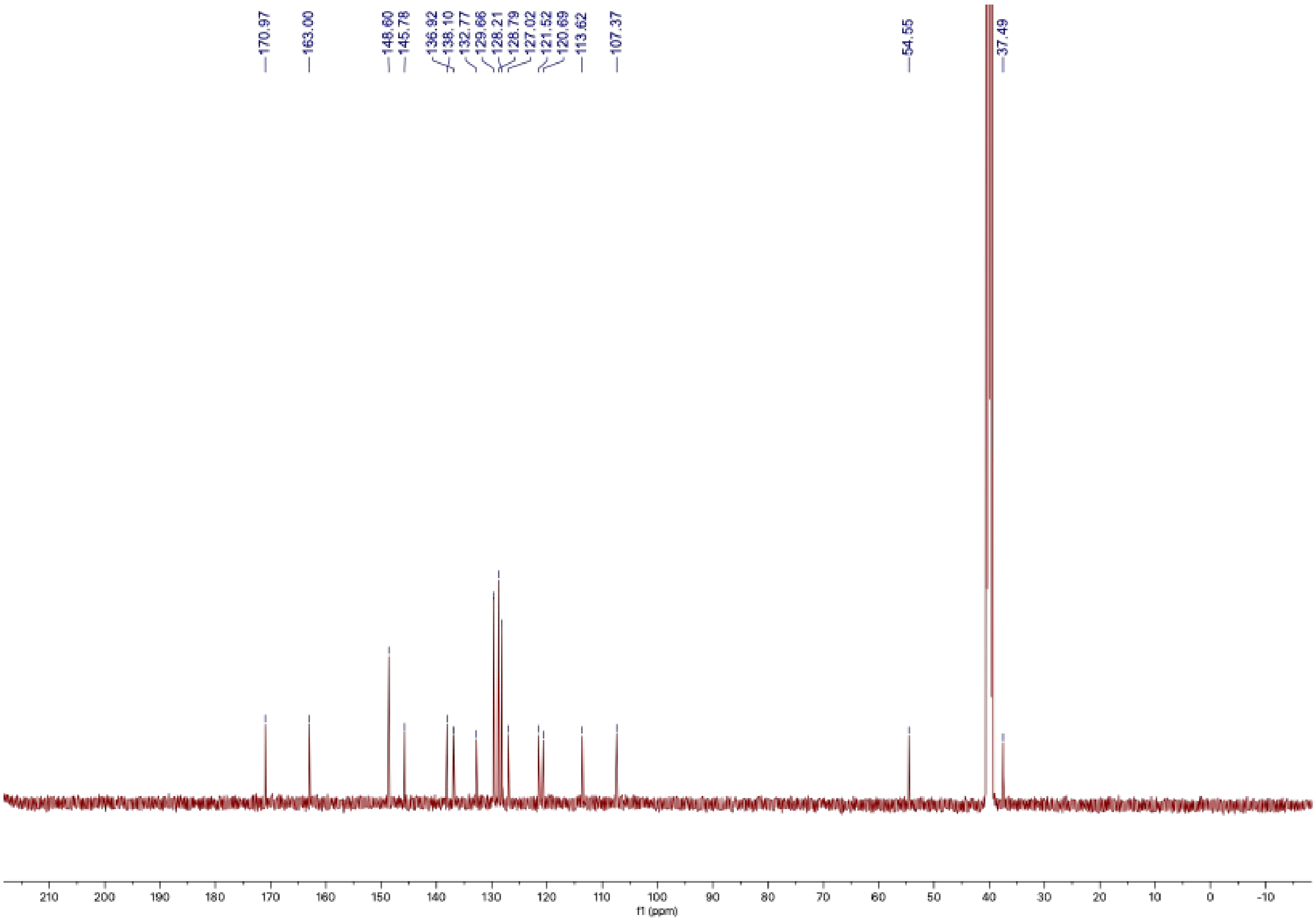

(S)-3,5-dinitro-*N*-(1-oxo-3-phenyl-1-(2-(2-(trifluoromethyl)phenyl)hydrazineyl)propan-2-yl)benzamide, 34

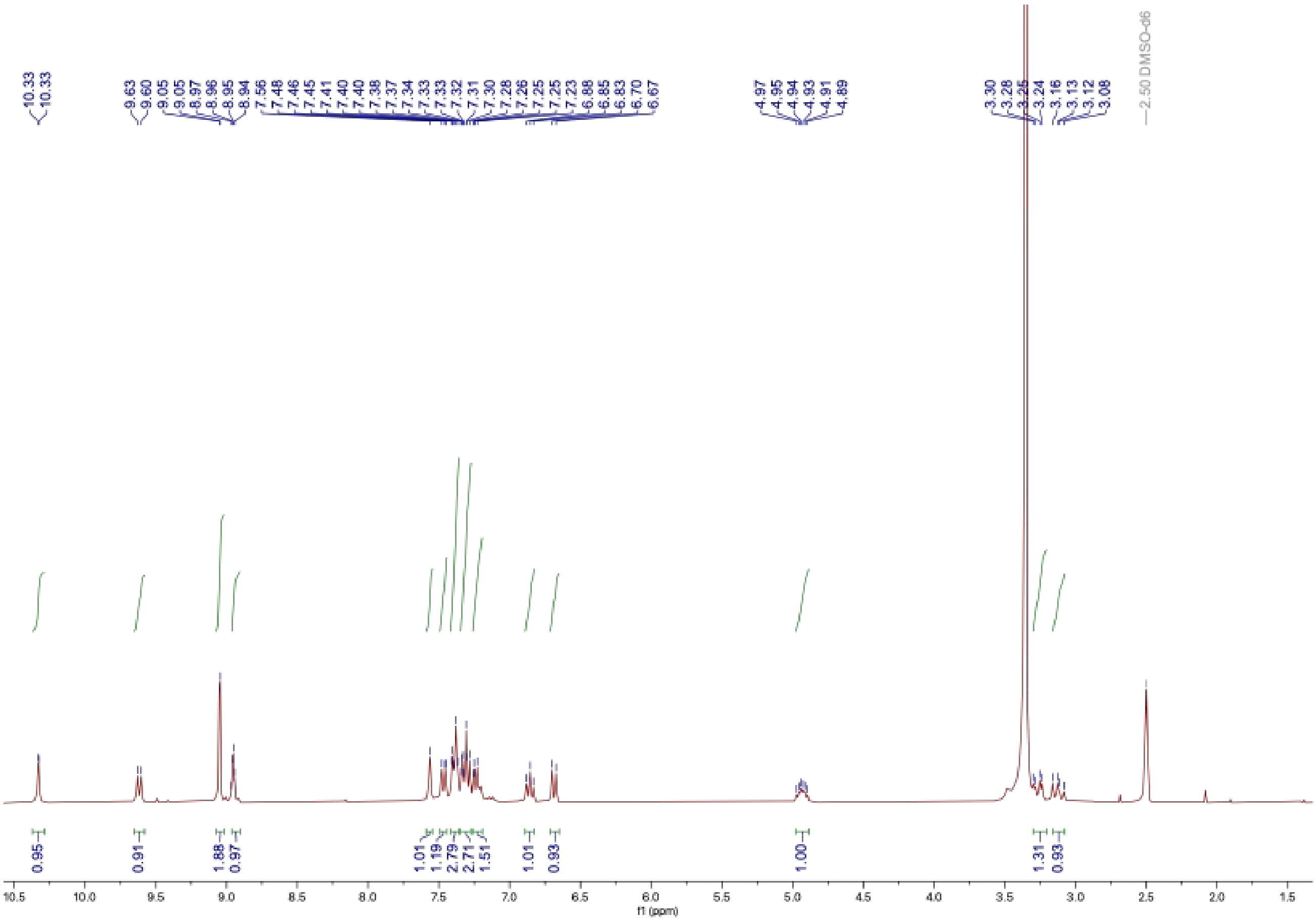

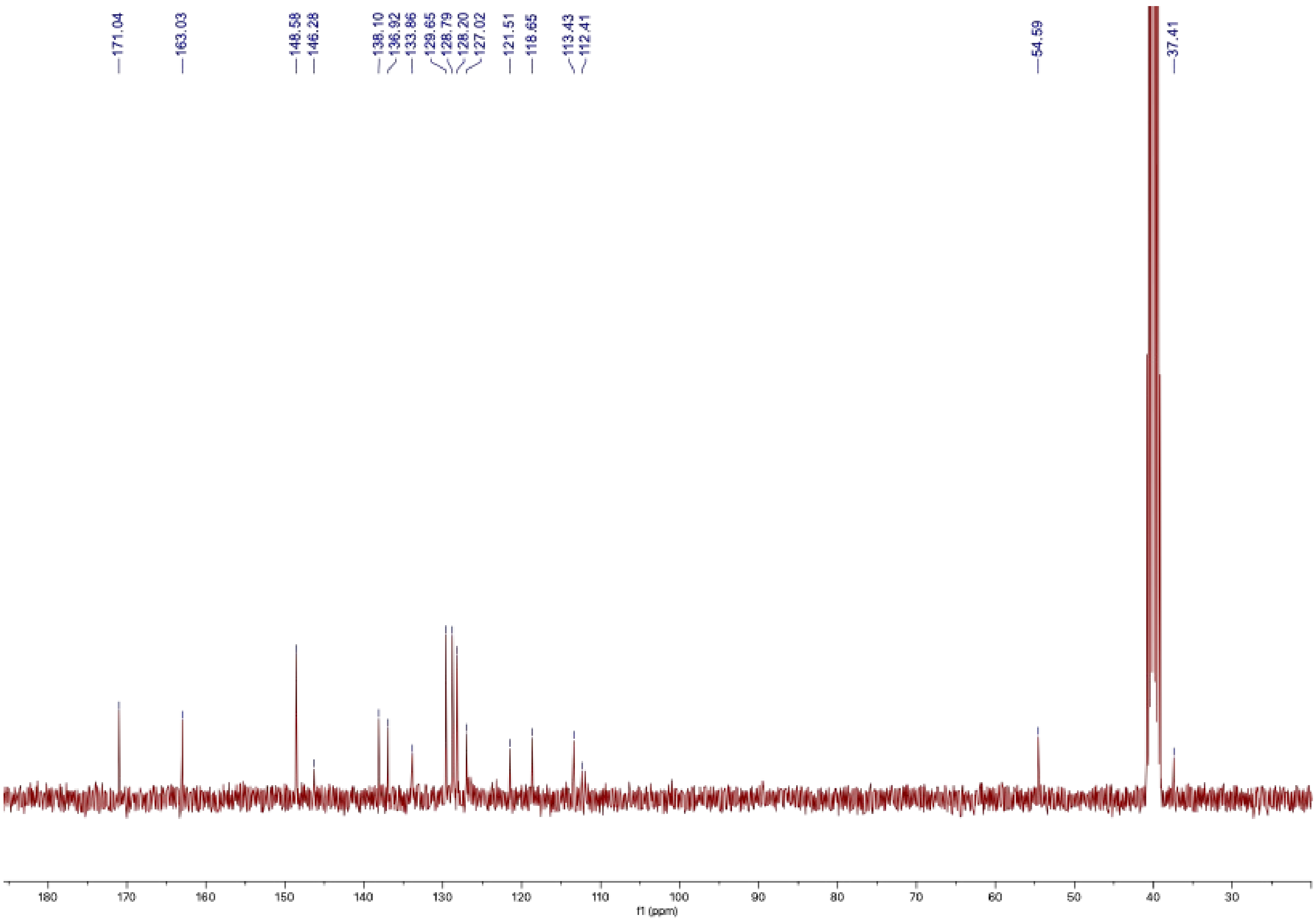

(S)-*N*-(1-(2-(3-chlorophenyl)hydrazineyl)-1-oxo-3-phenylpropan-2-yl)-3,5-dinitrobenzamide, 35

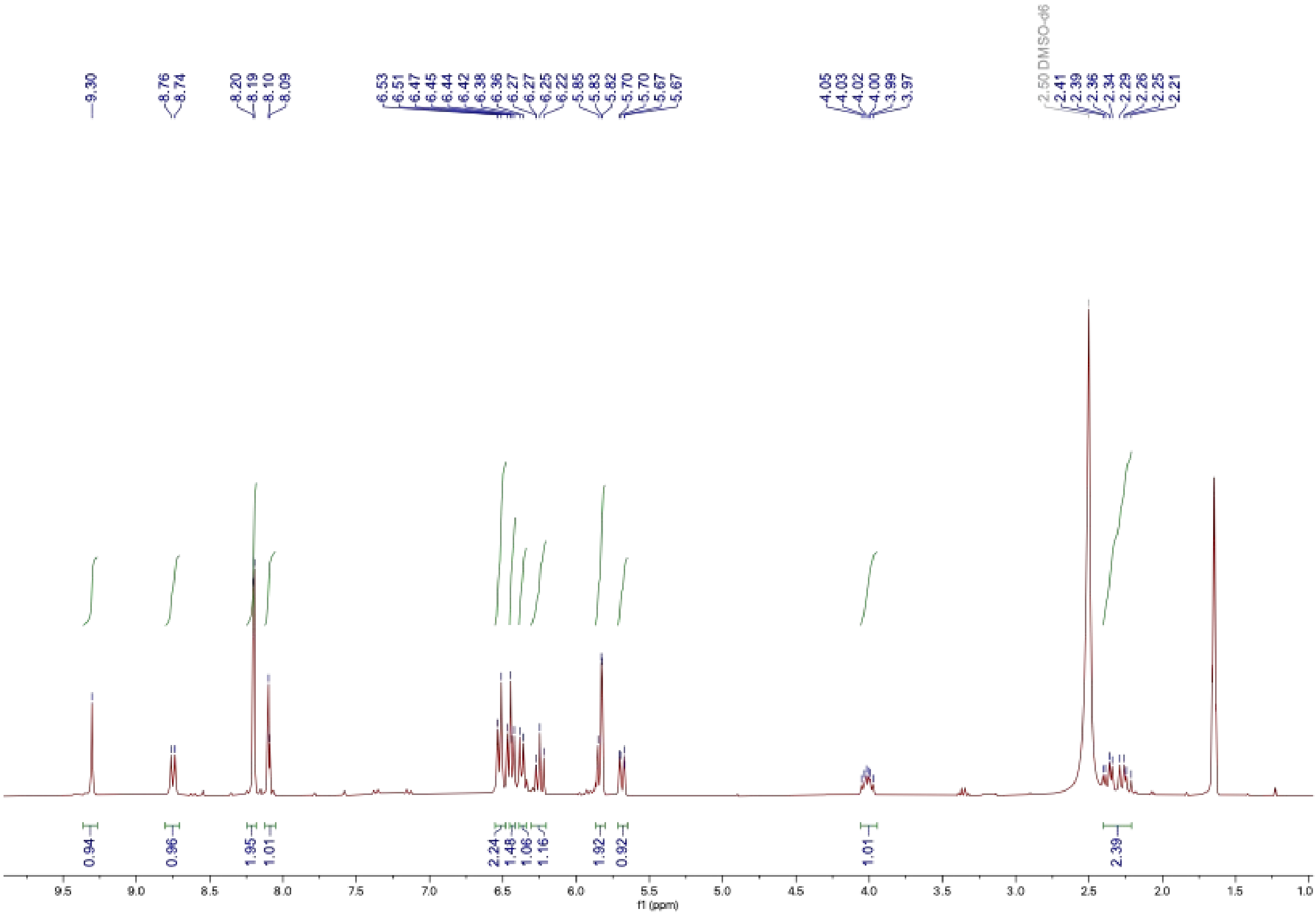

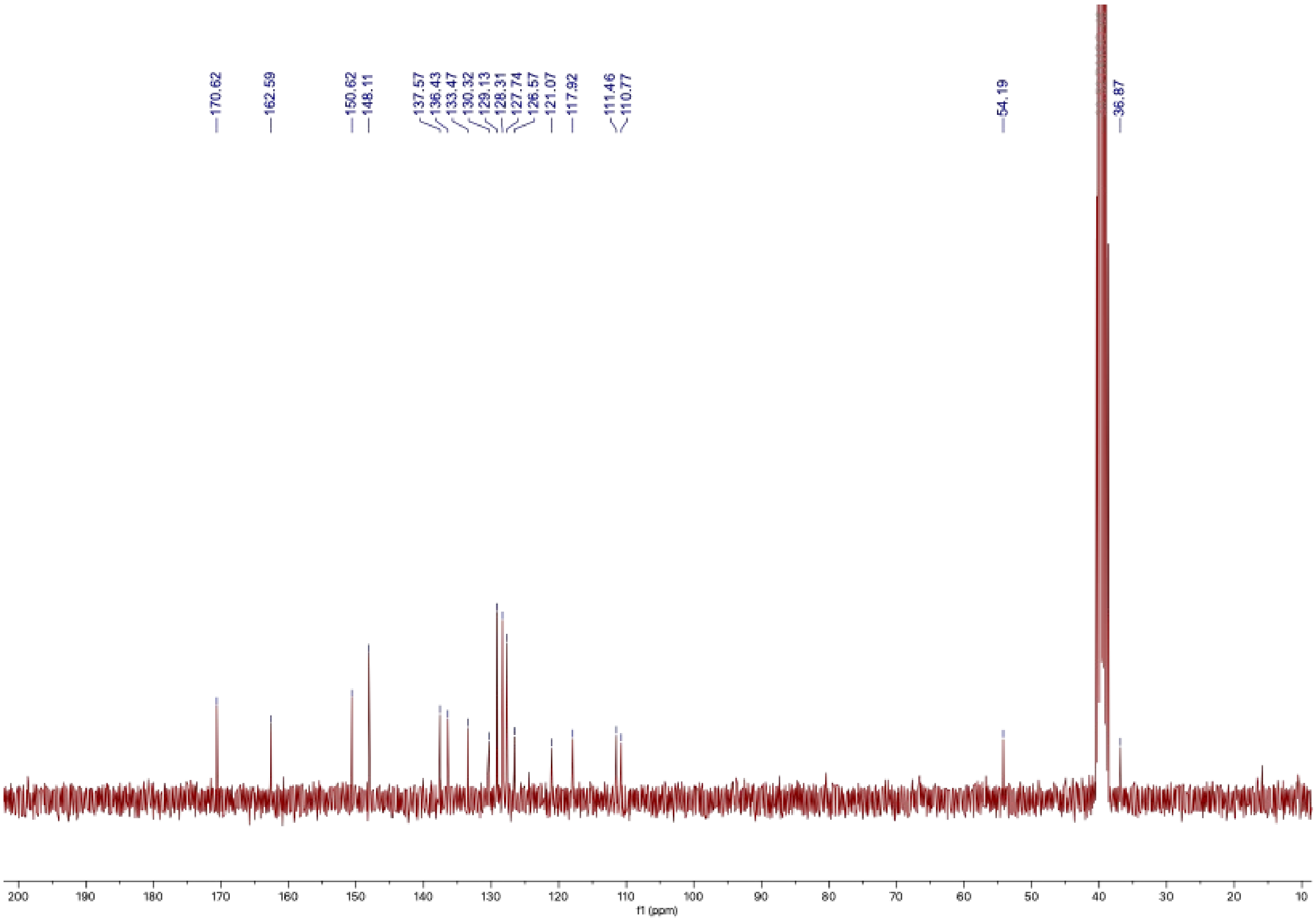

(S)-*N*-(1-(2-(3-bromophenyl)hydrazineyl)-1-oxo-3-phenylpropan-2-yl)-3,5-dinitrobenzamide, 36

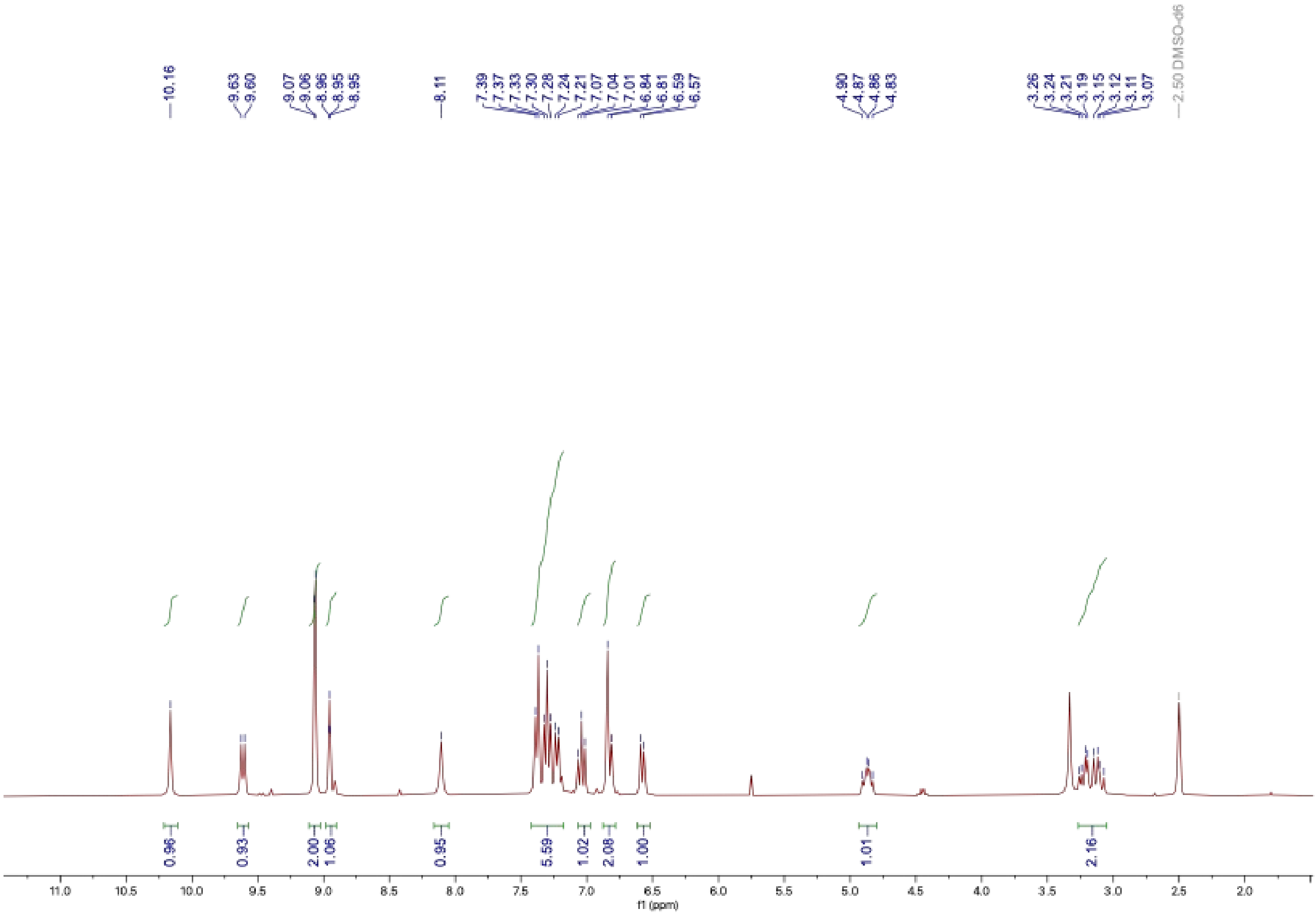

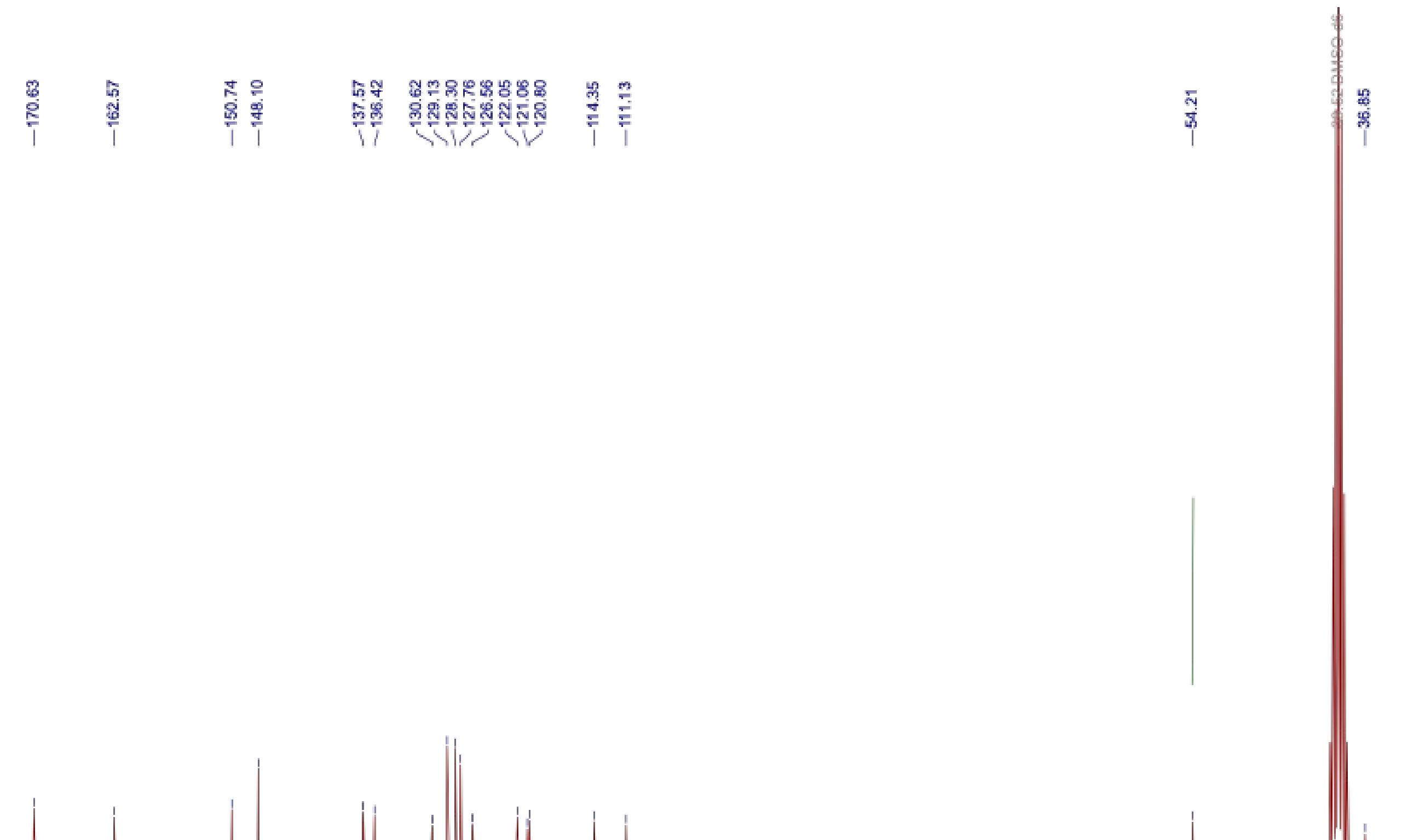

(S)-3,5-dinitro-*N*-(1-oxo-3-phenyl-1-(2-(3-(trifluoromethyl)phenyl)hydrazineyl)propan-2-yl)benzamide, 37

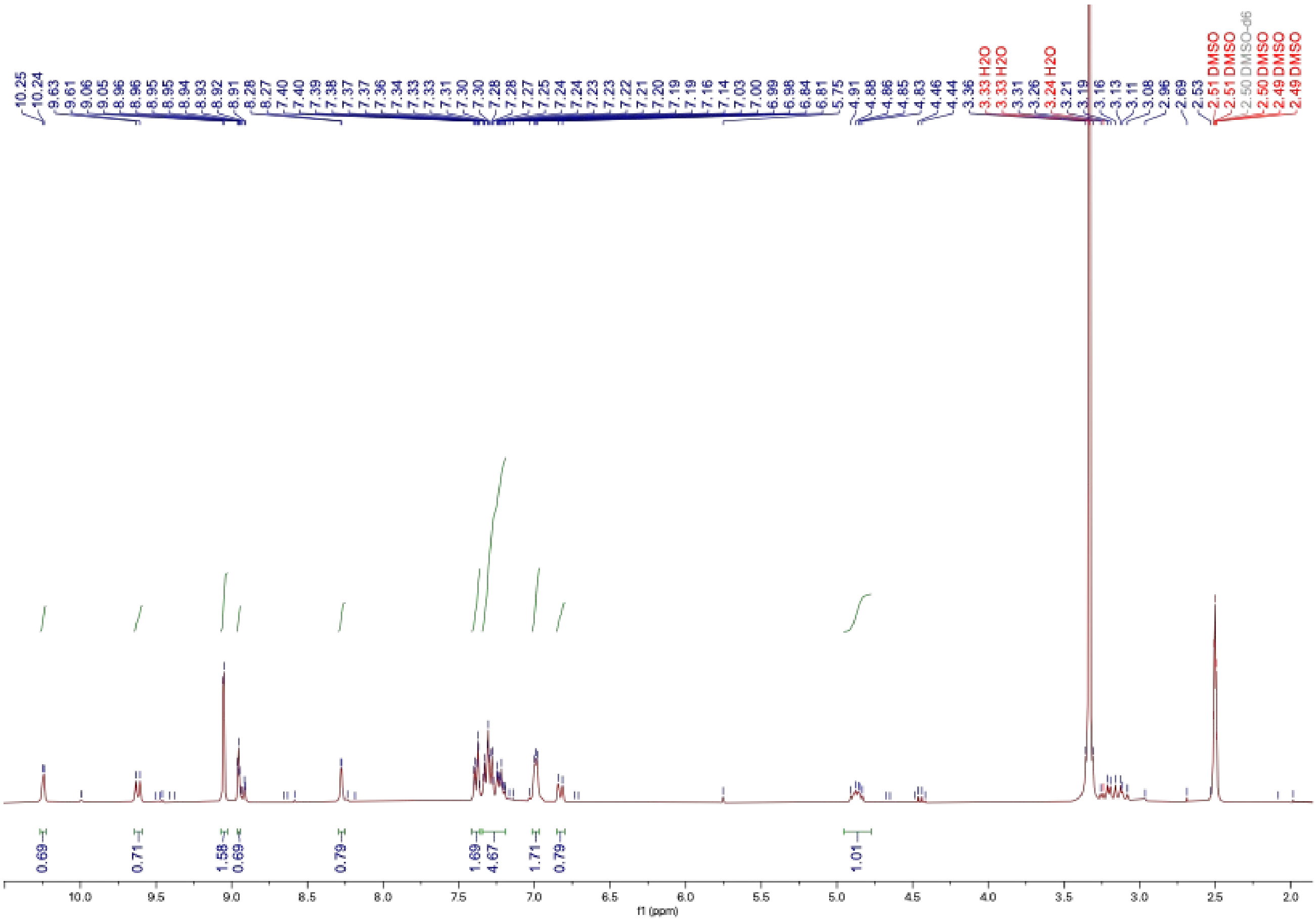

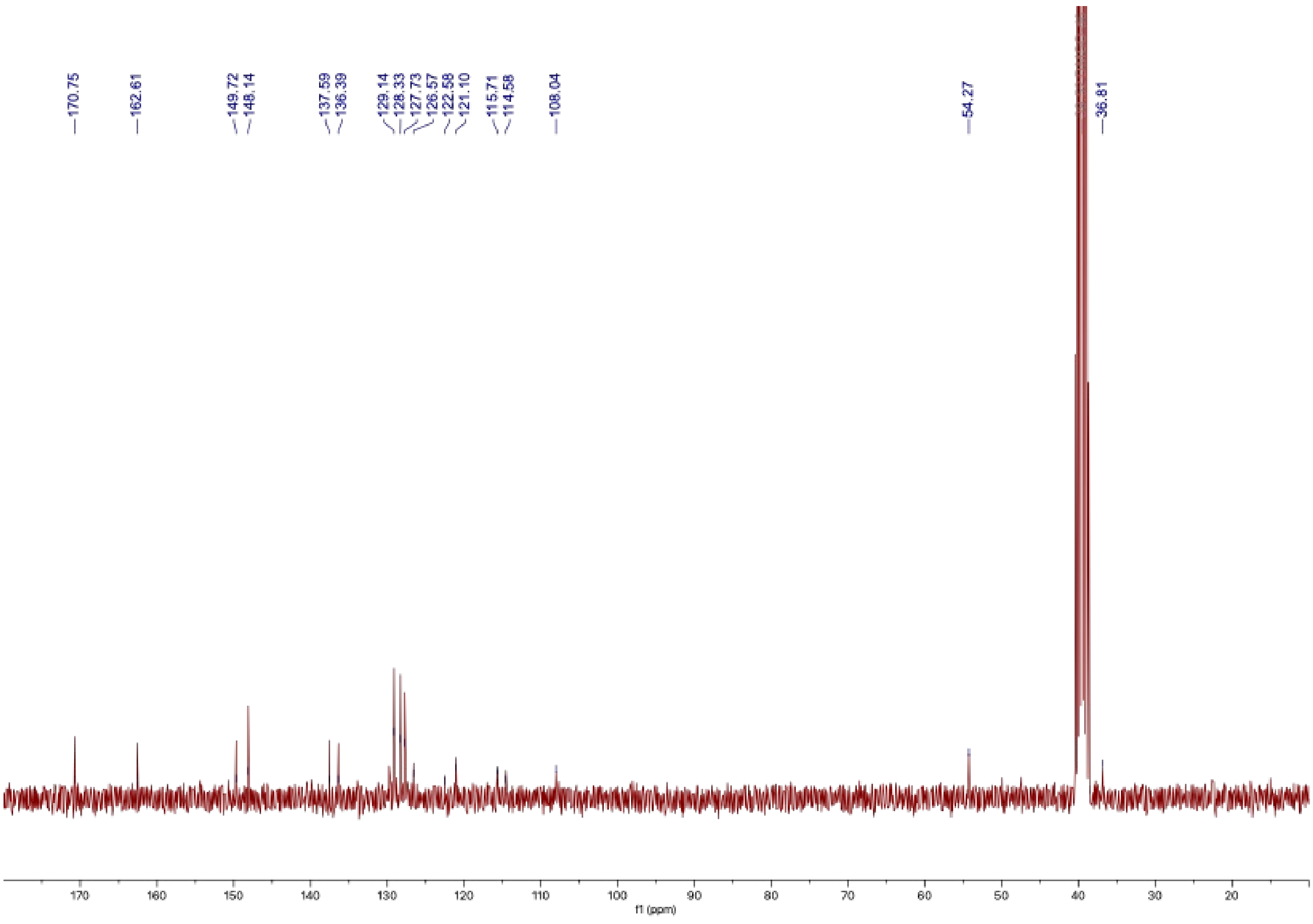

(S)-*N*-(1-(2-(4-chlorophenyl)hydrazineyl)-1-oxo-3-phenylpropan-2-yl)-3,5-dinitrobenzamide, 38

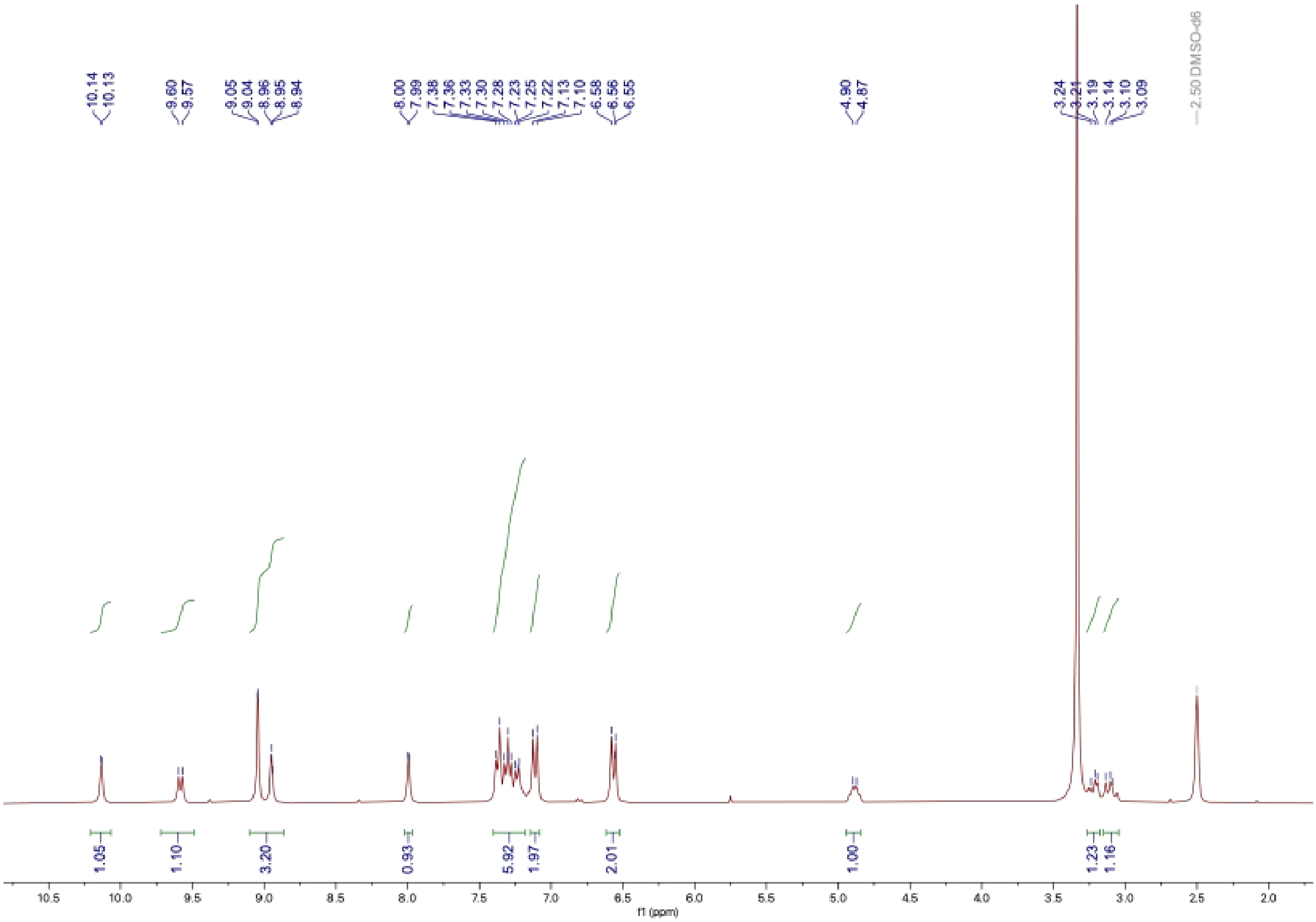

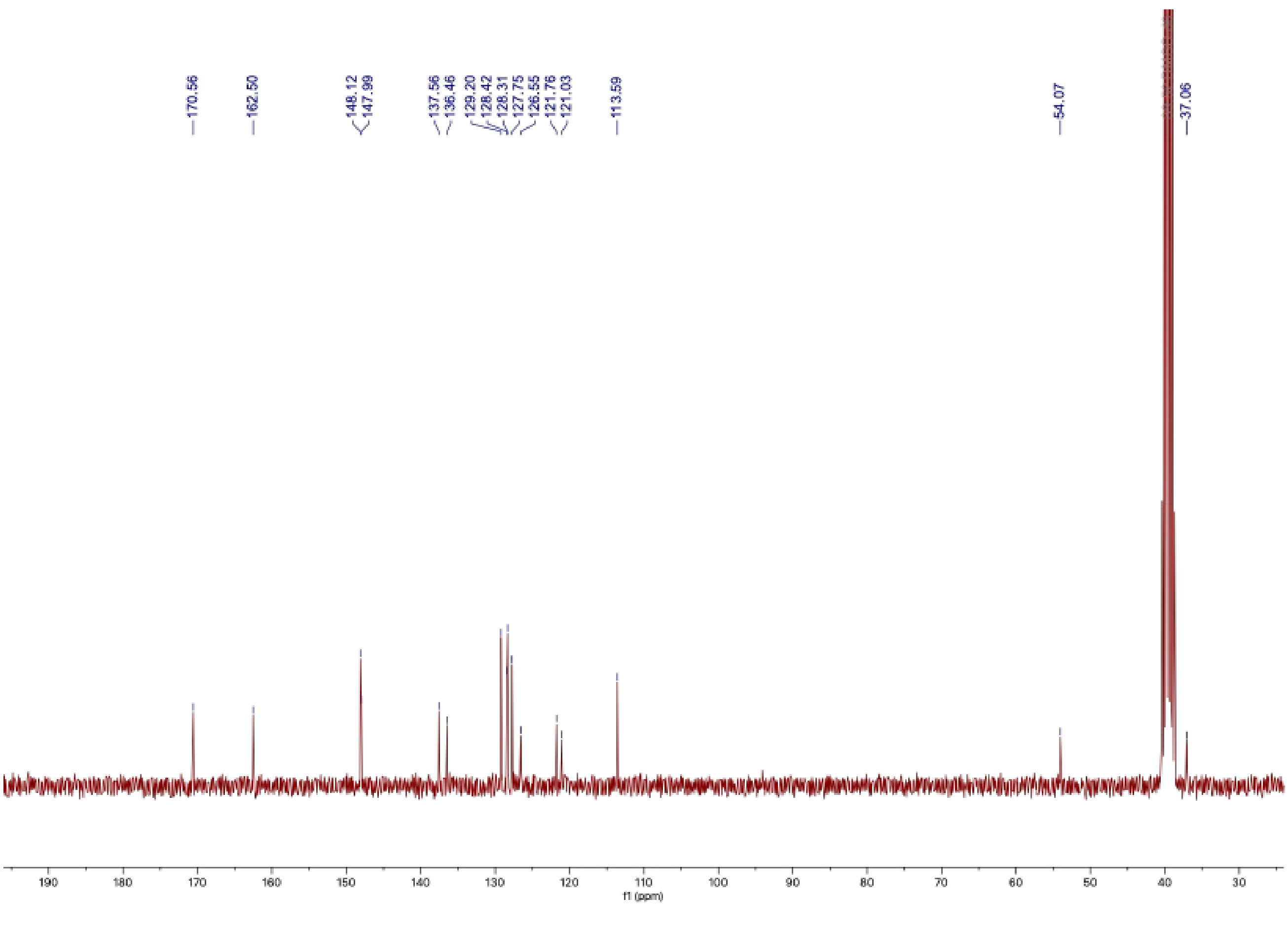

(S)-*N*-(1-(2-(4-bromophenyl)hydrazineyl)-1-oxo-3-phenylpropan-2-yl)-3,5-dinitrobenzamide, 39

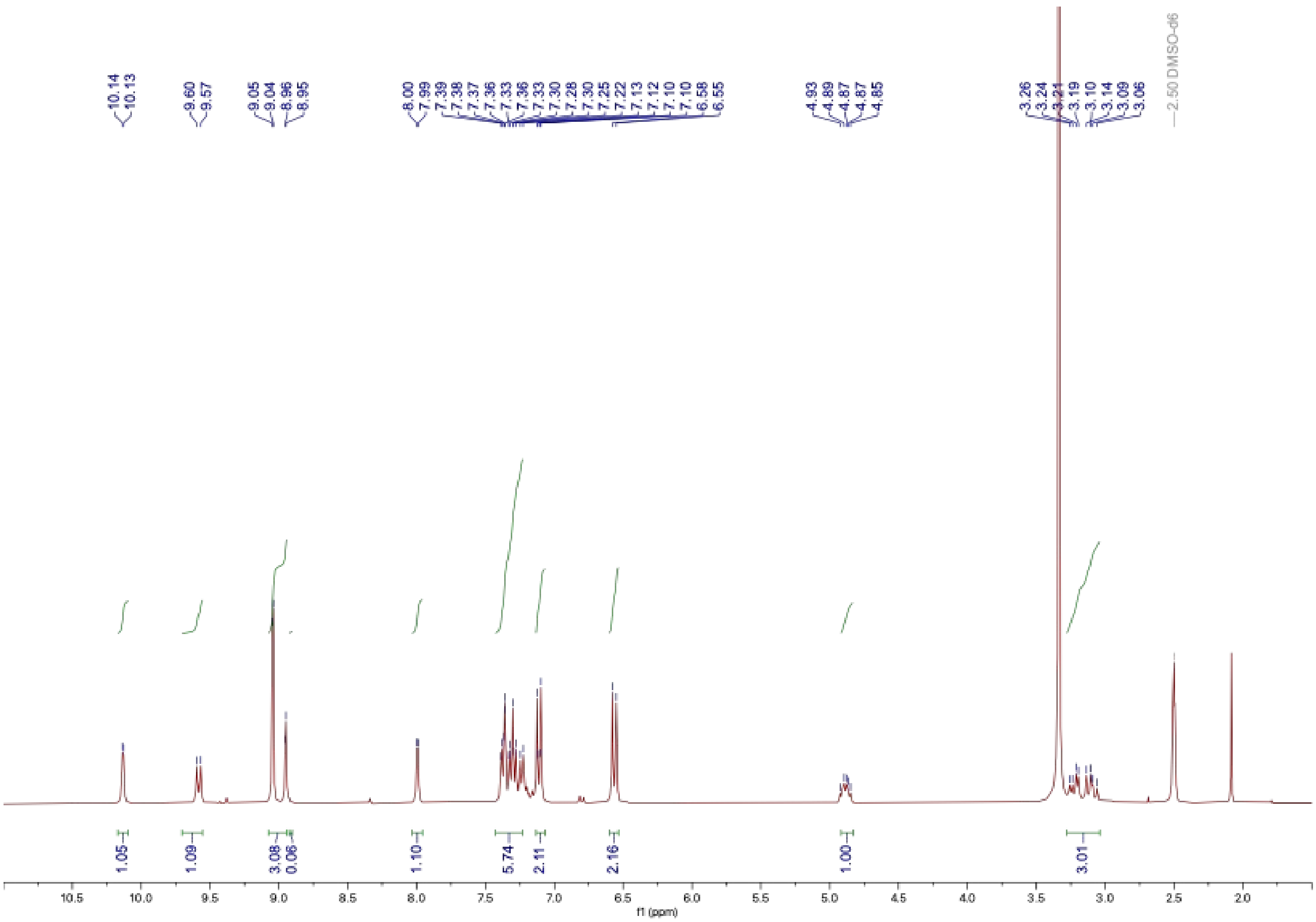

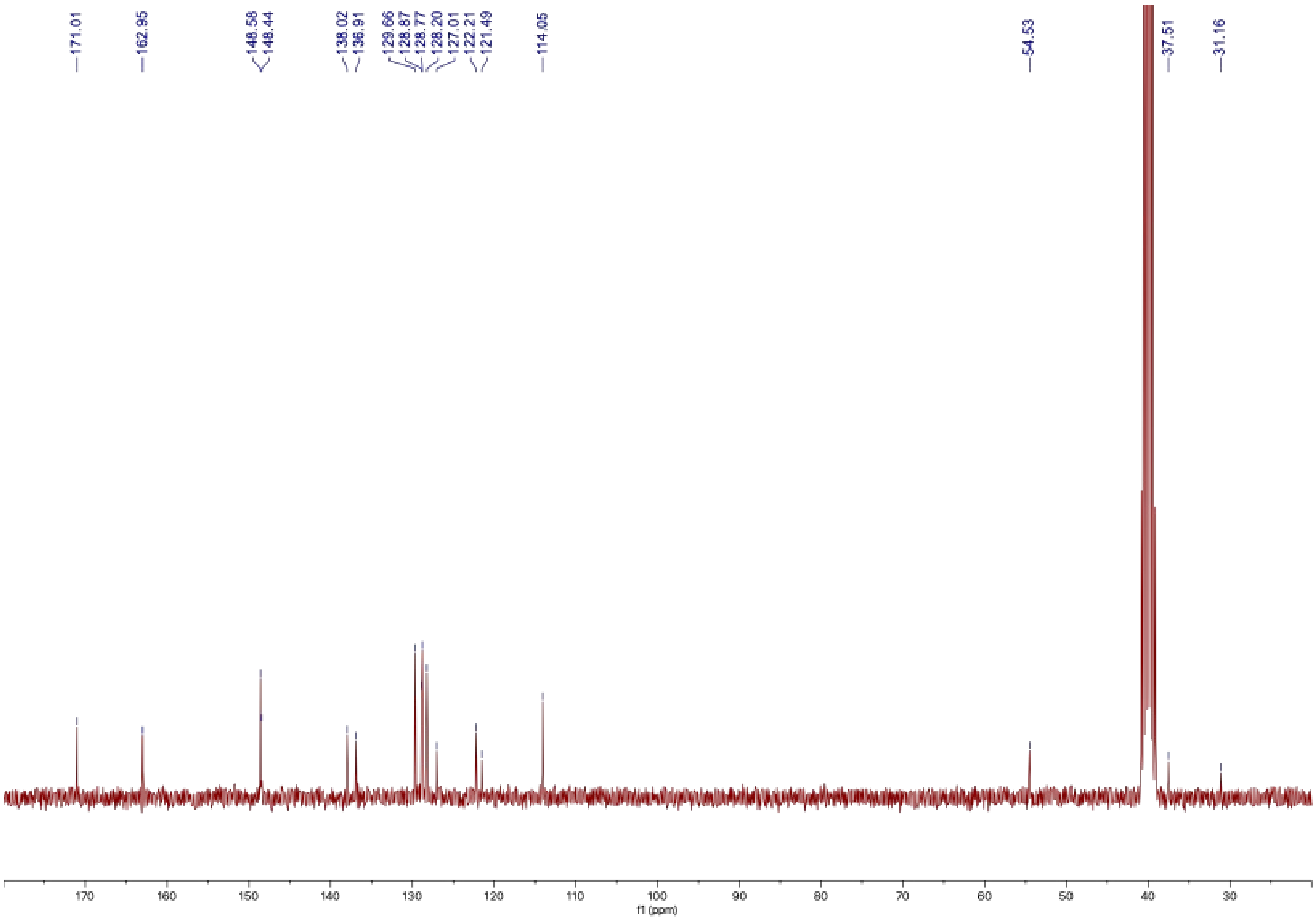

(S)-3,5-dinitro-*N*-(1-oxo-3-phenyl-1-(2-(4-(trifluoromethyl)phenyl)hydrazineyl)propan-2-yl)benzamide, 40

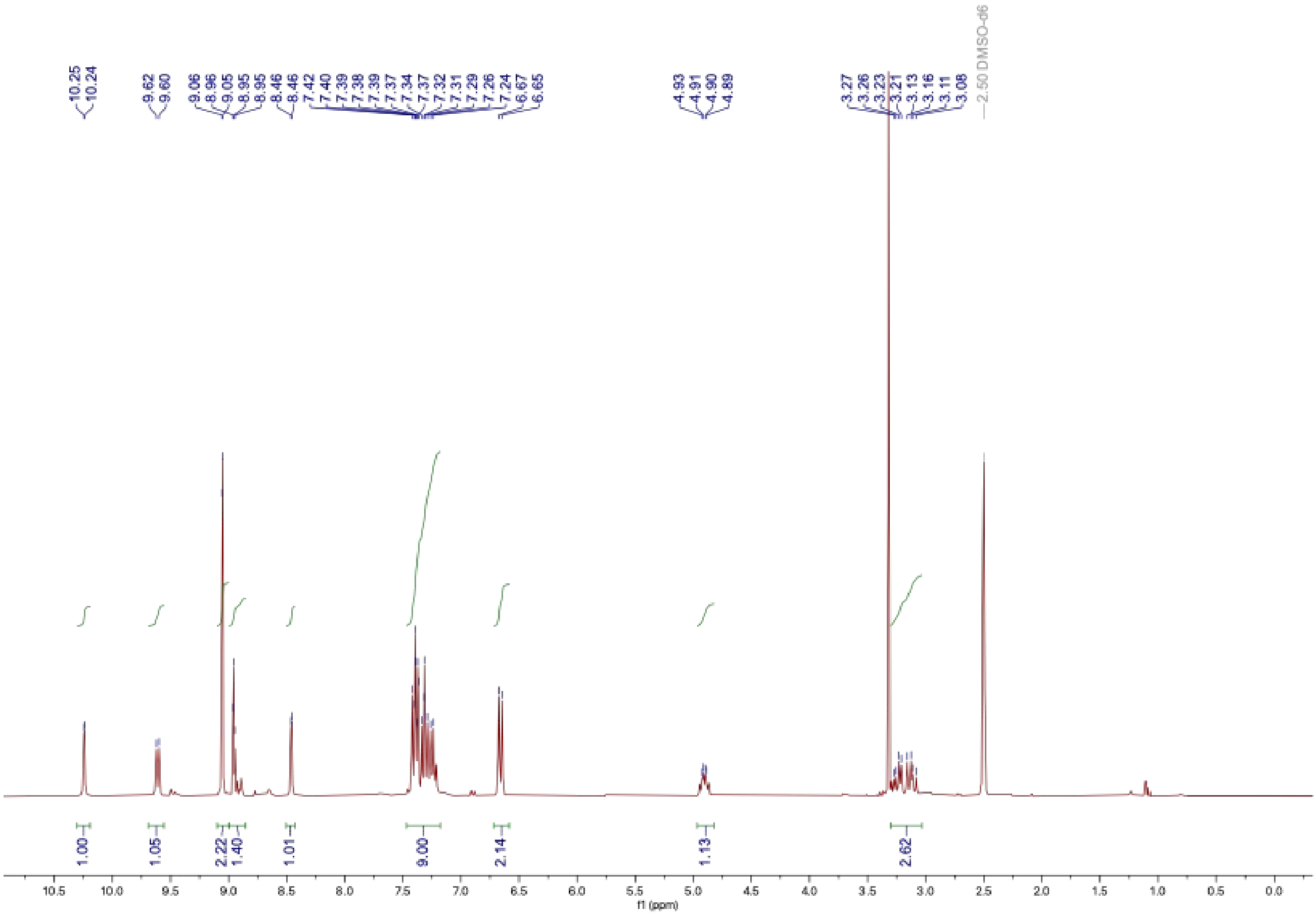

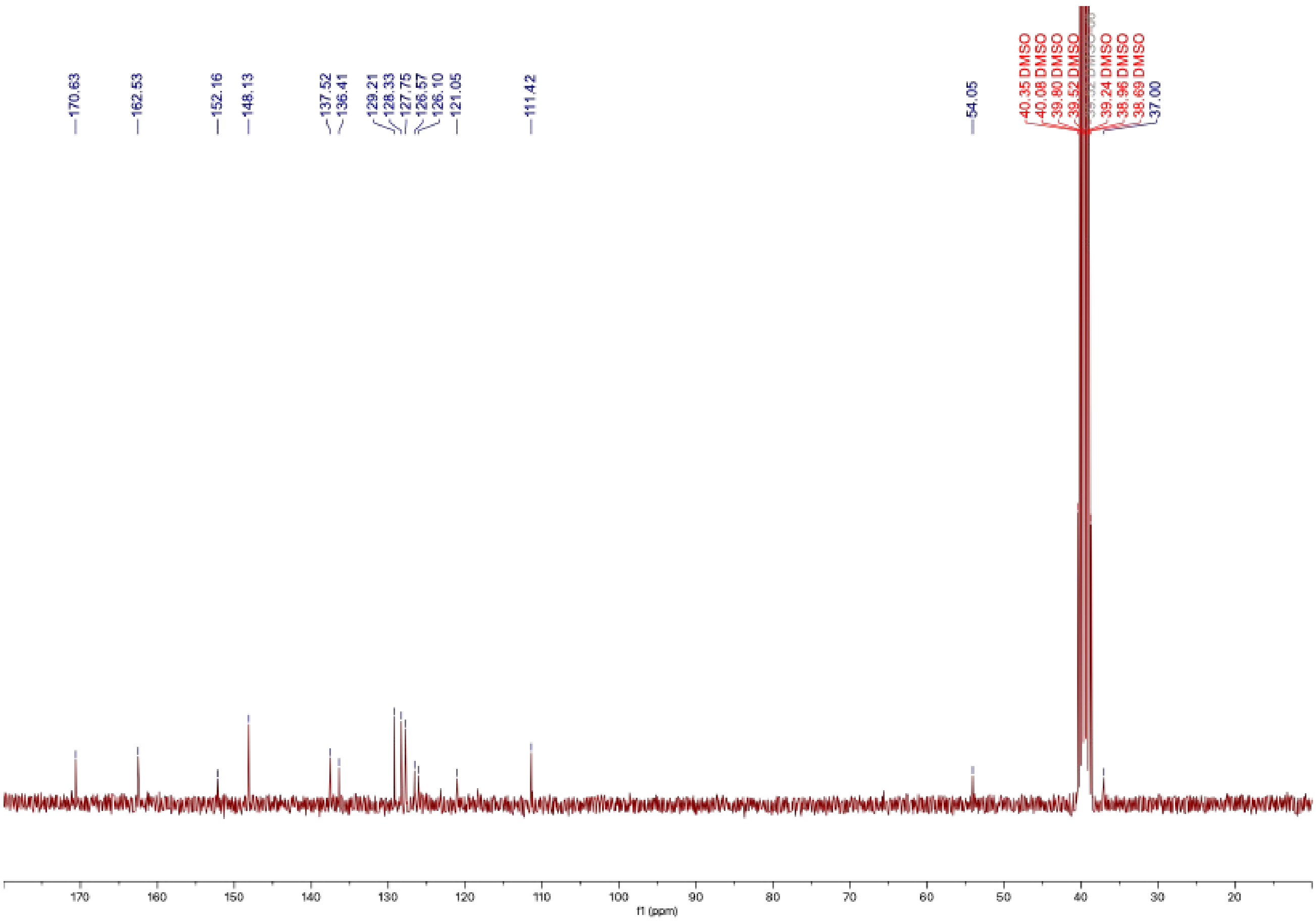

(S)-N-(1-(2-(2-bromophenyl)hydrazineyl)-4-(methylthio)-1-oxobutan-2-yl)-3,5-dinitrobenzamide, 41

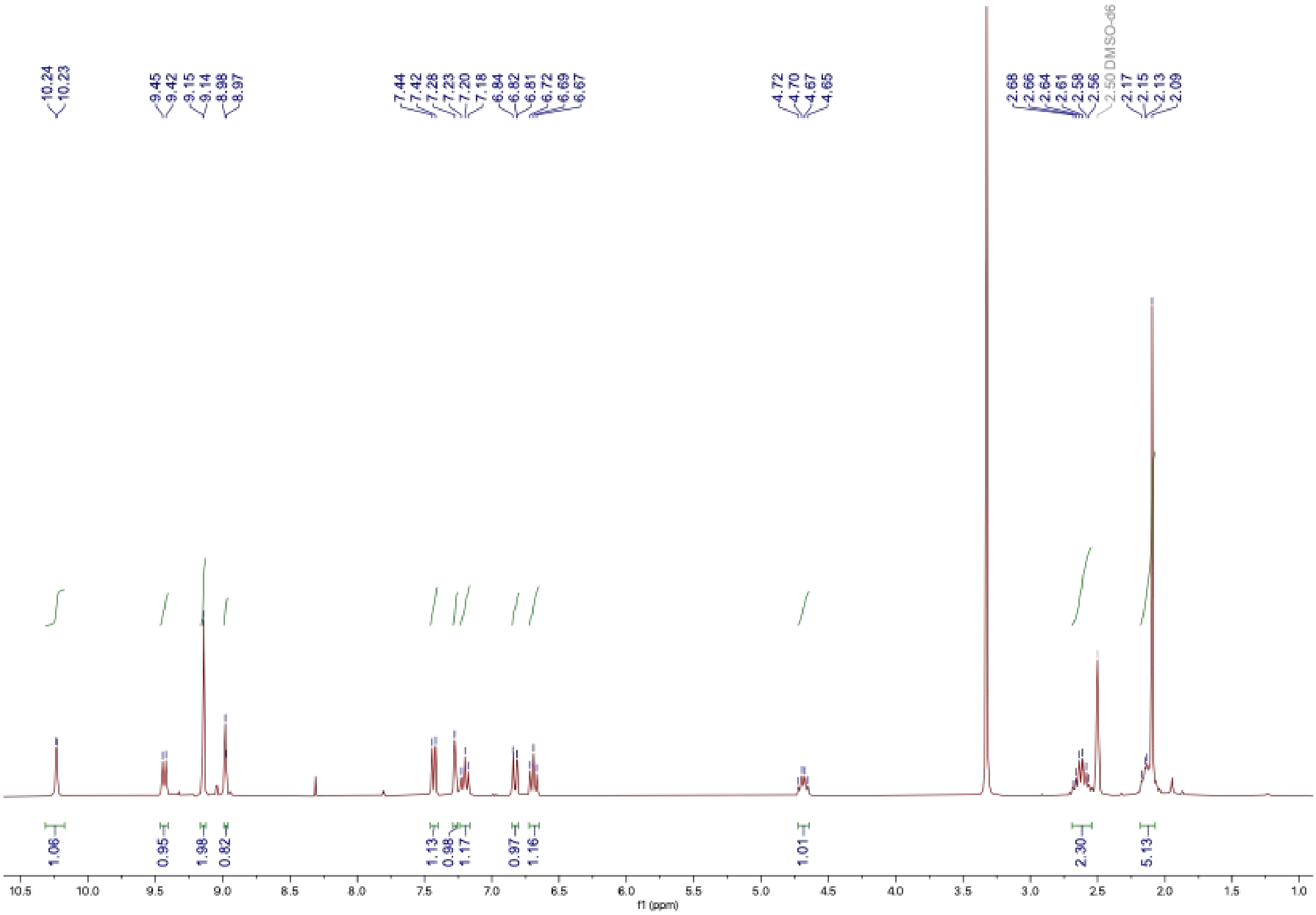

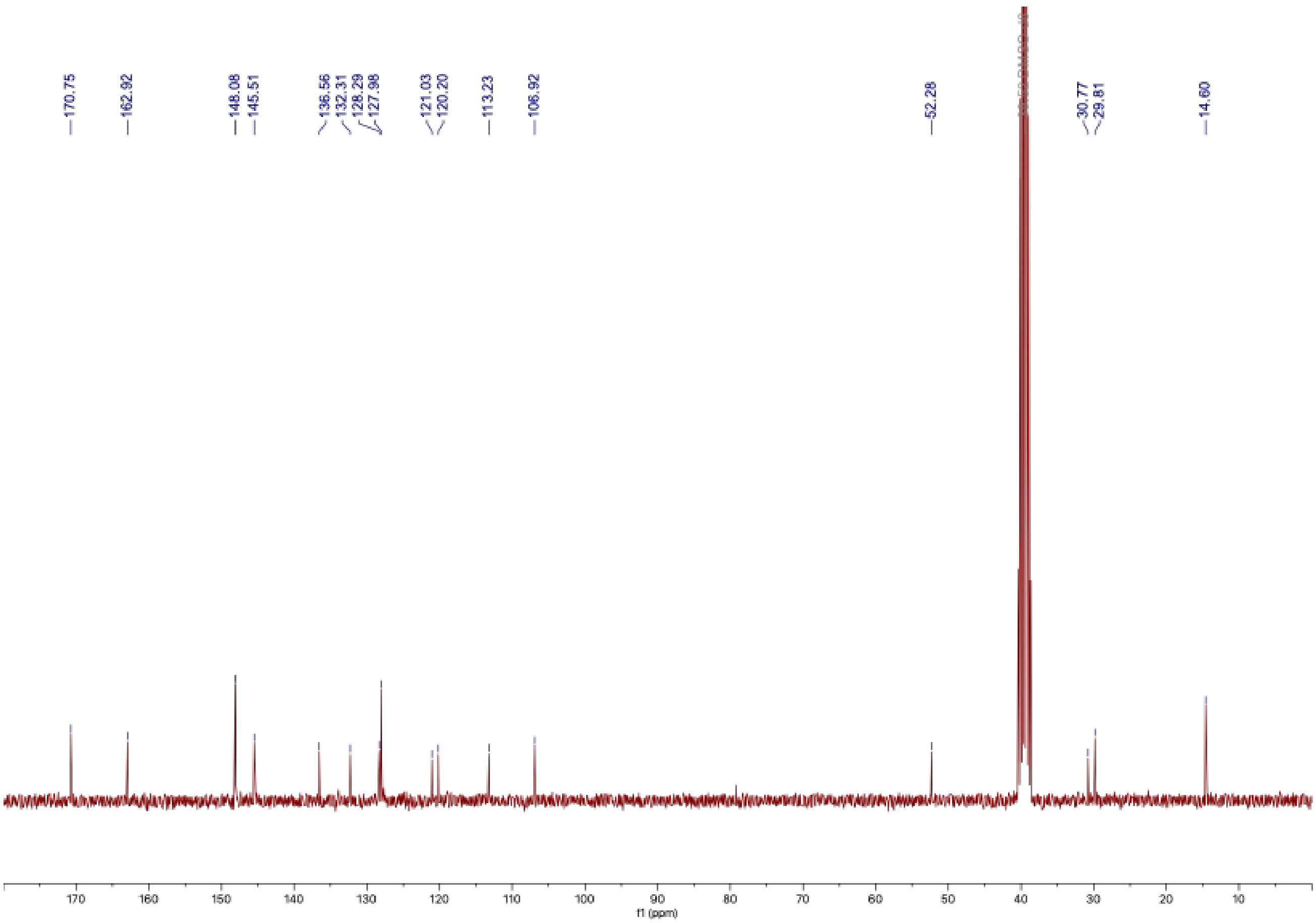

(*S*)-*N*-(4-(methylthio)-1-oxo-1-(2-(2-(trifluoromethyl)phenyl)hydrazineyl)butan-2-yl)-3,5-dinitrobenzamide, 42

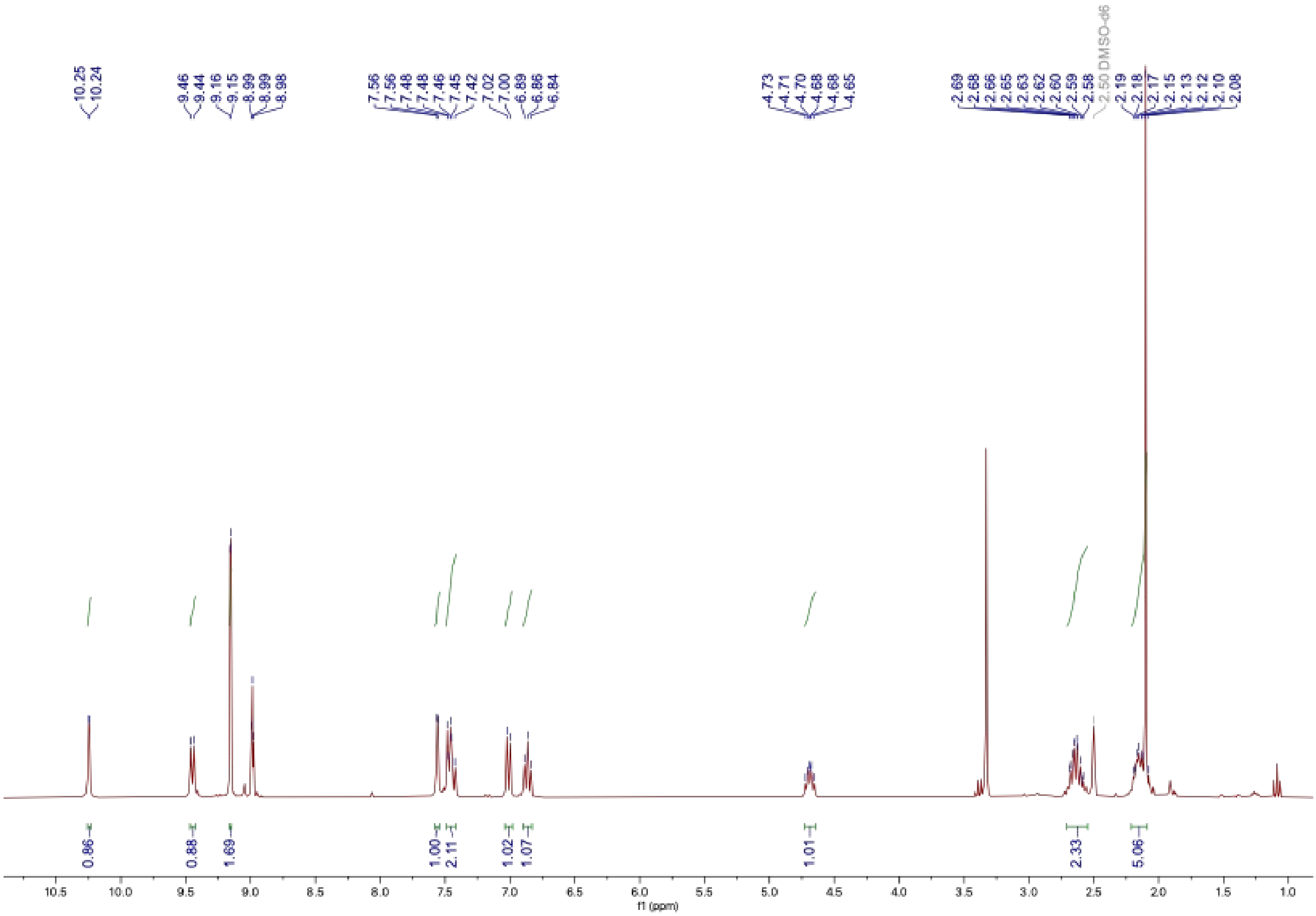

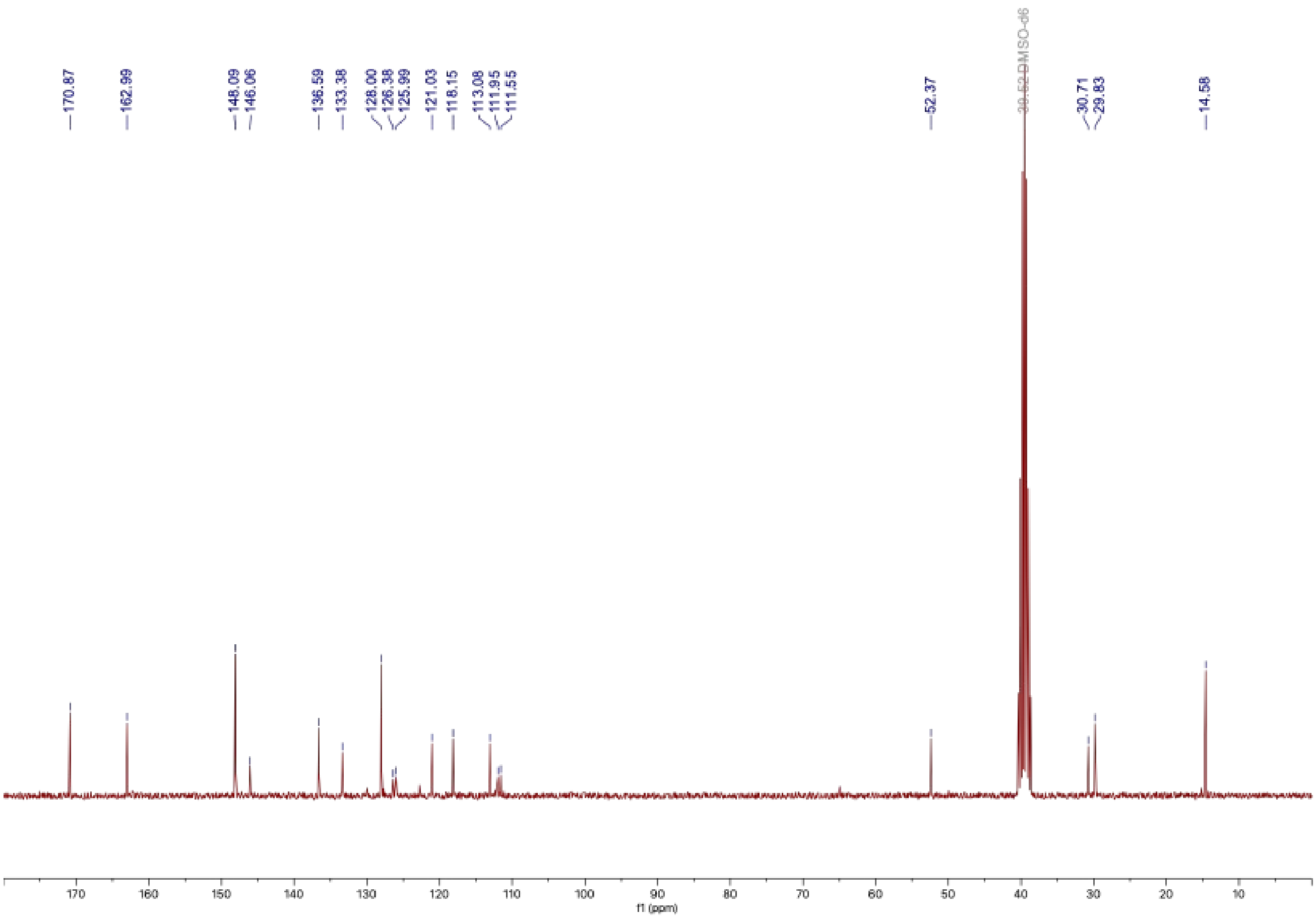

(S)-*N*-(1-(2-(3-bromophenyl)hydrazineyl)-4-(methylthio)-1-oxobutan-2-yl)-3,5-dinitrobenzamide, 43

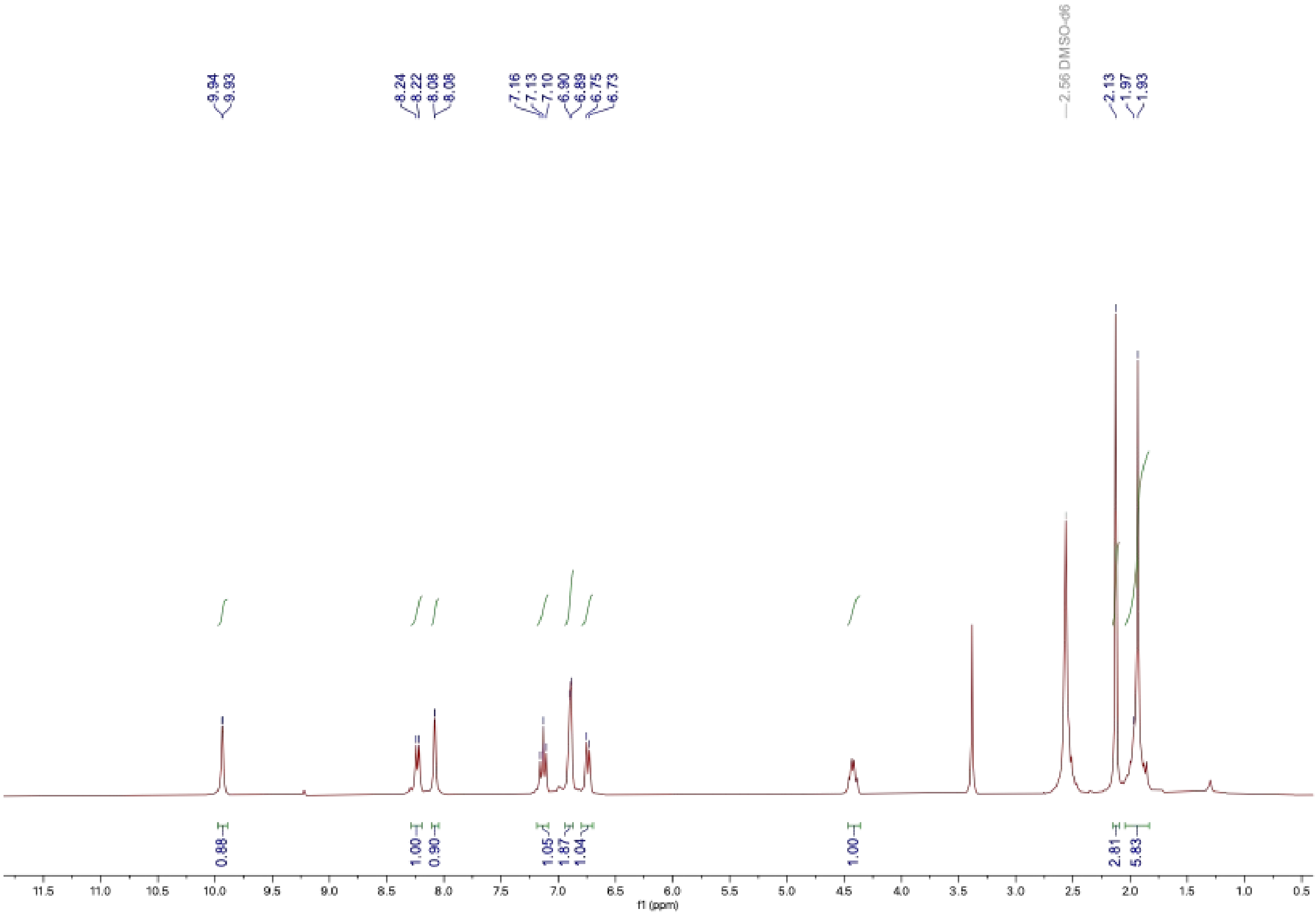

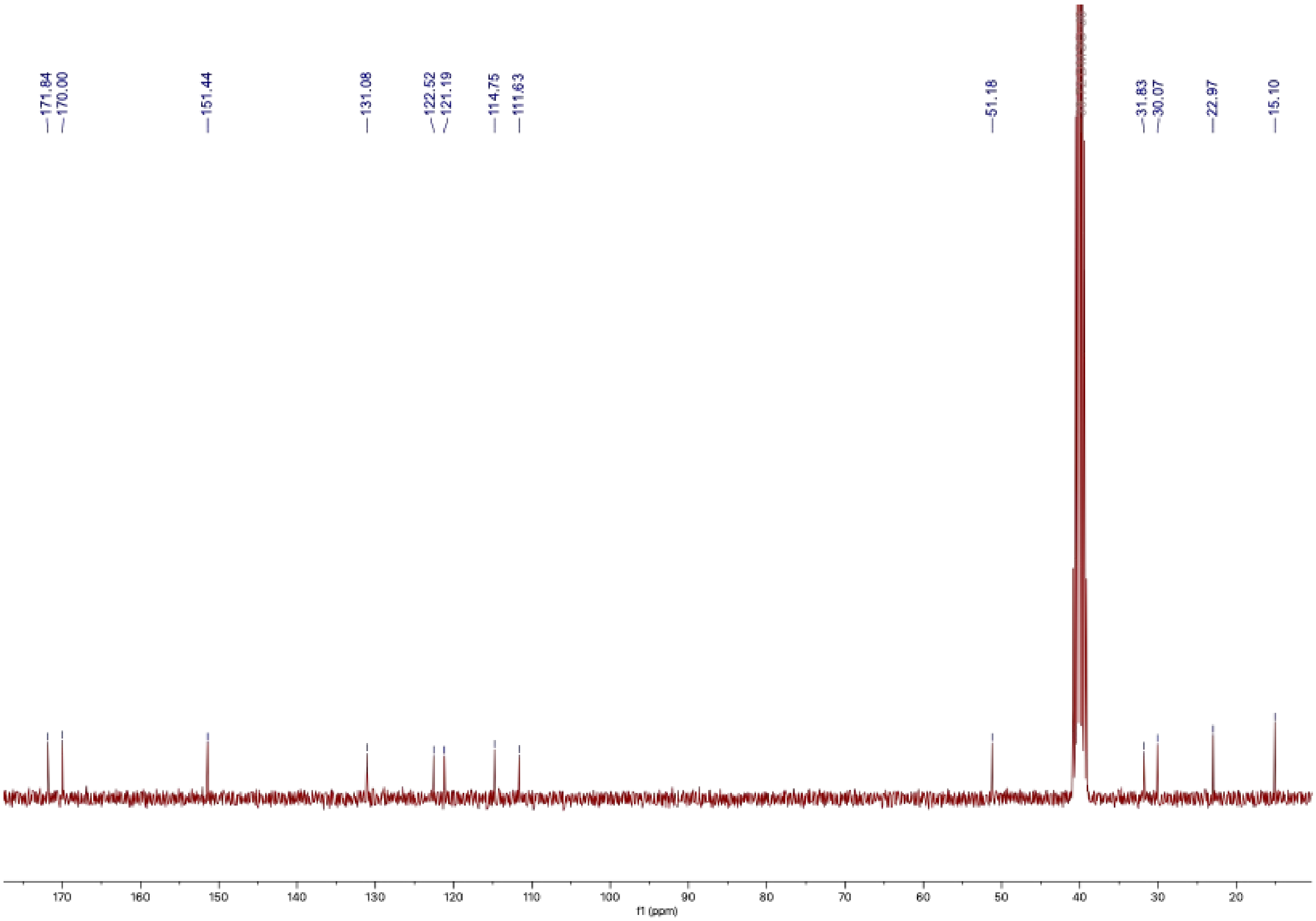

(S)-*N*-(4-(methylthio)-1-oxo-1-(2-(3-(trifluoromethyl)phenyl)hydrazineyl)butan-2-yl)-3,5-dinitrobenzamide, 44

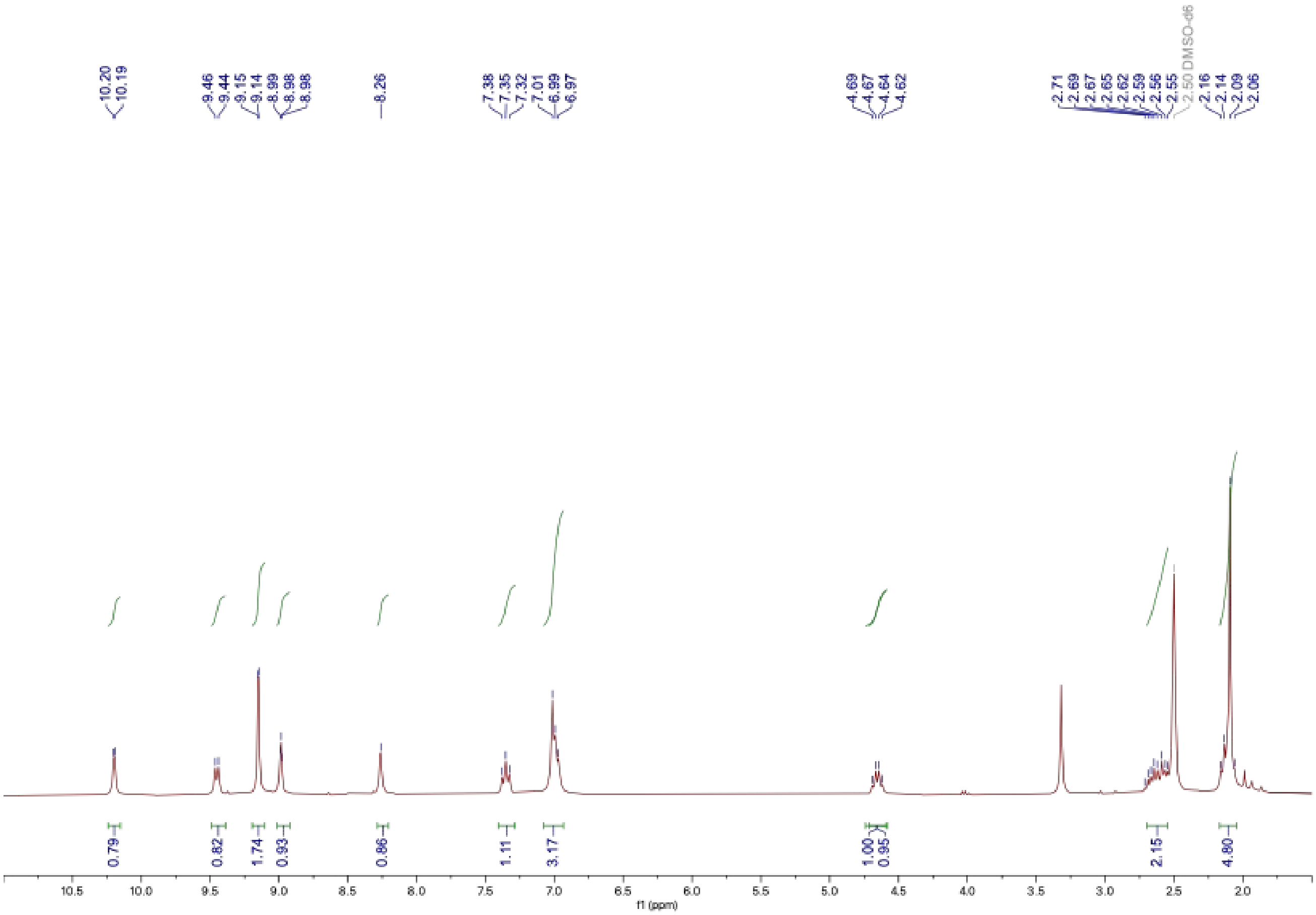

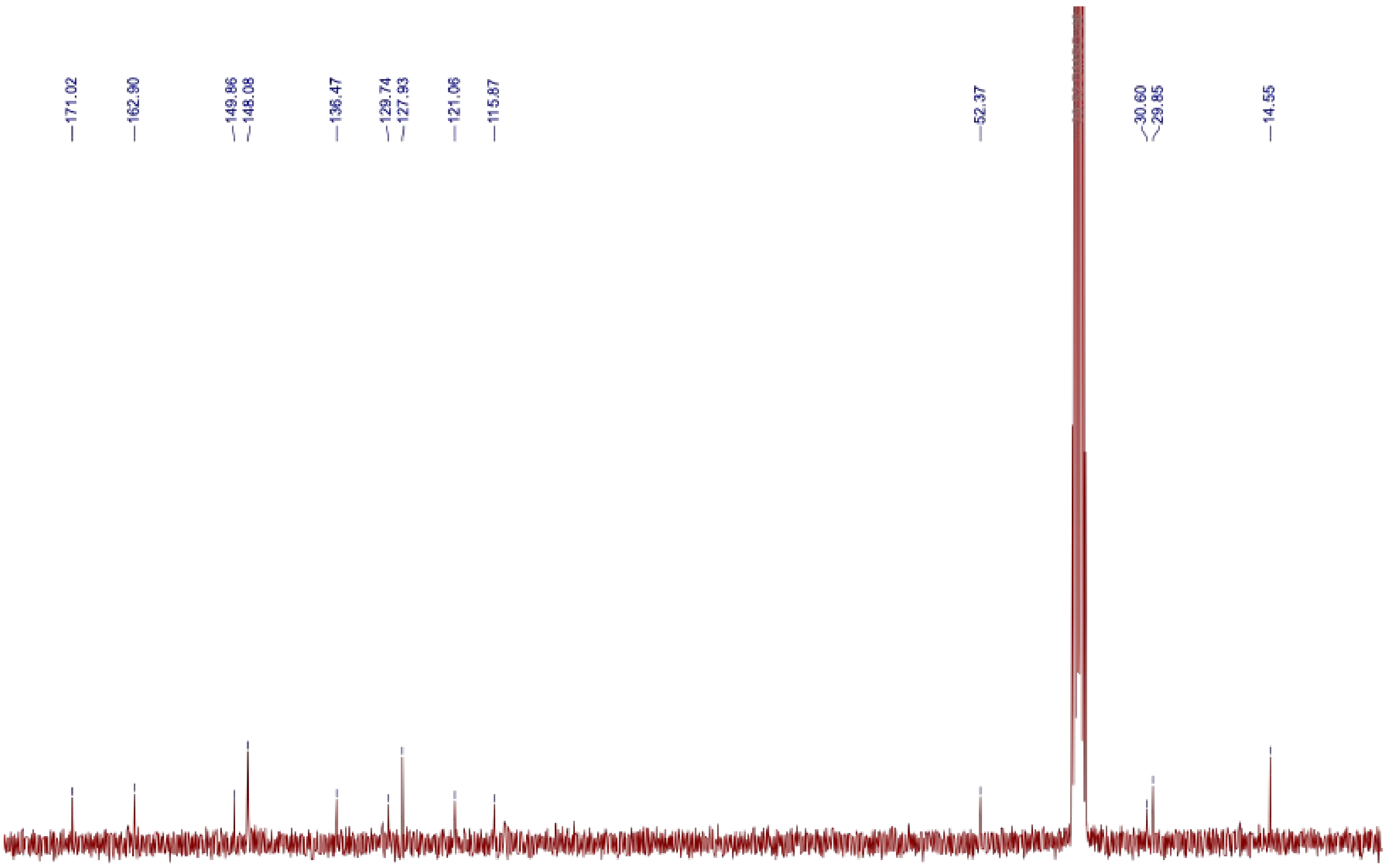

(S)-*N*-(1-(2-(4-chlorophenyl)hydrazineyl)-4-(methylthio)-1-oxobutan-2-yl)-3,5-dinitrobenzamide, 45

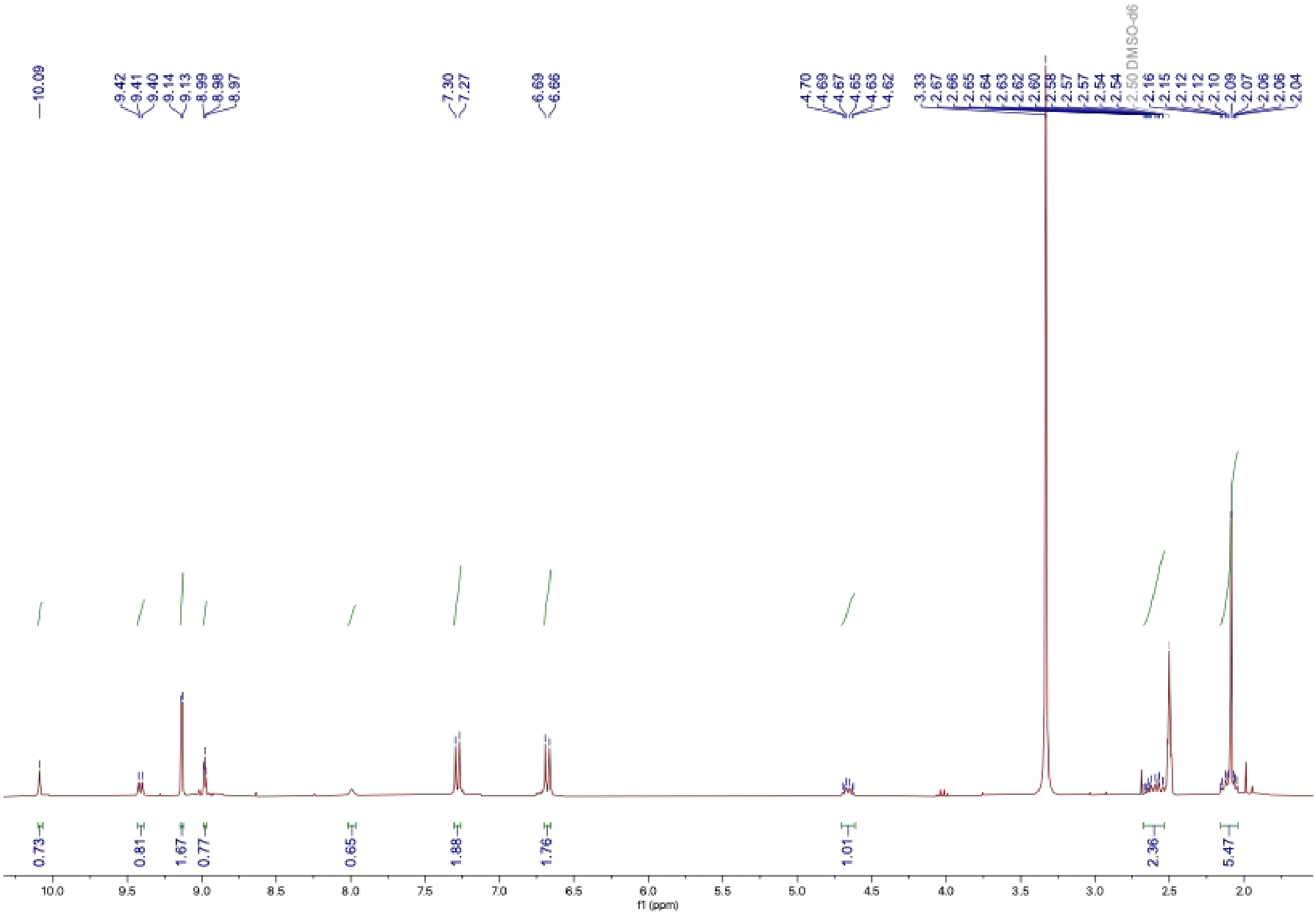

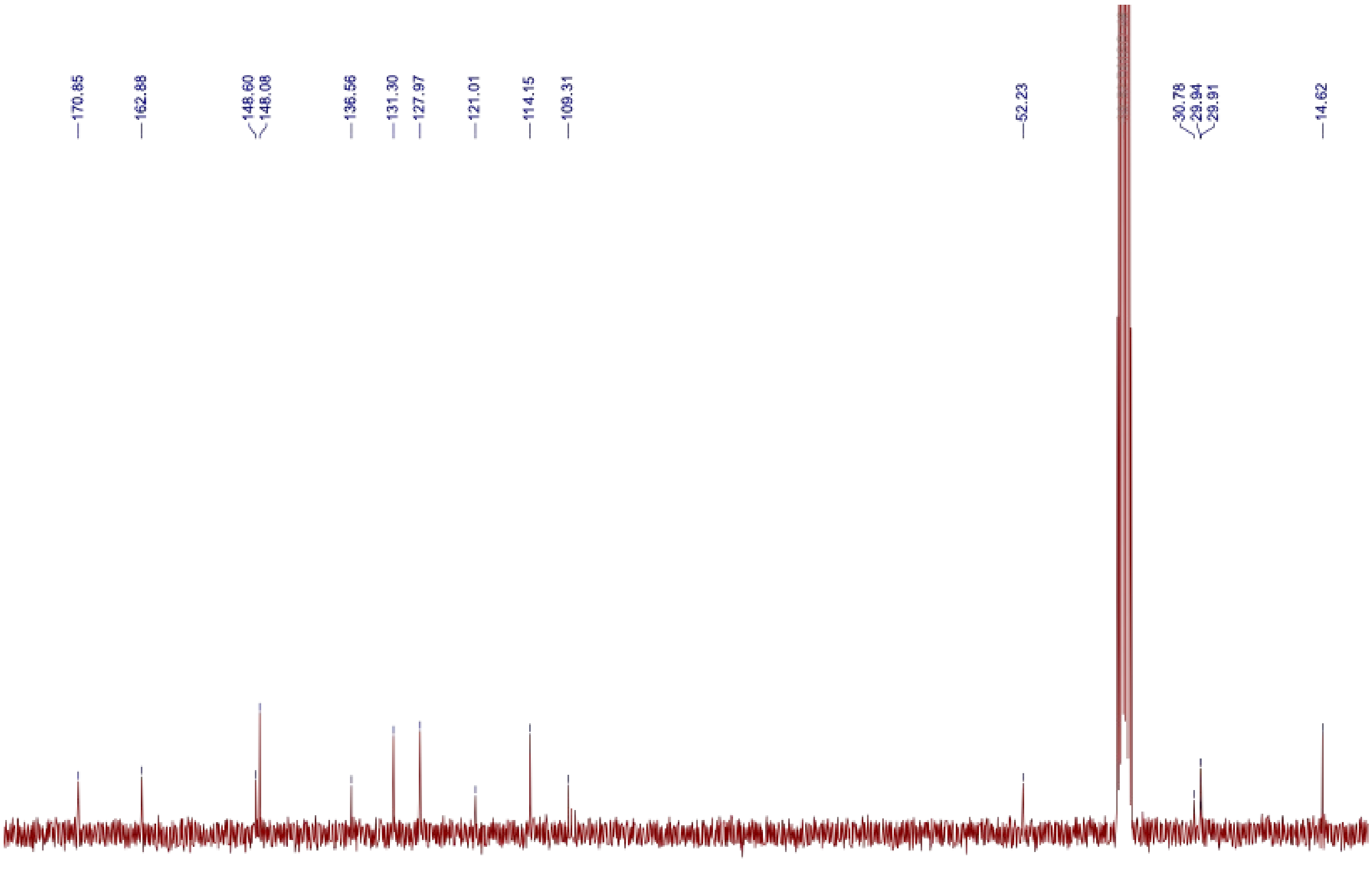

(S)-*N*-(1-(2-(4-bromophenyl)hydrazineyl)-4-(methylthio)-1-oxobutan-2-yl)-3,5-dinitrobenzamide, 46

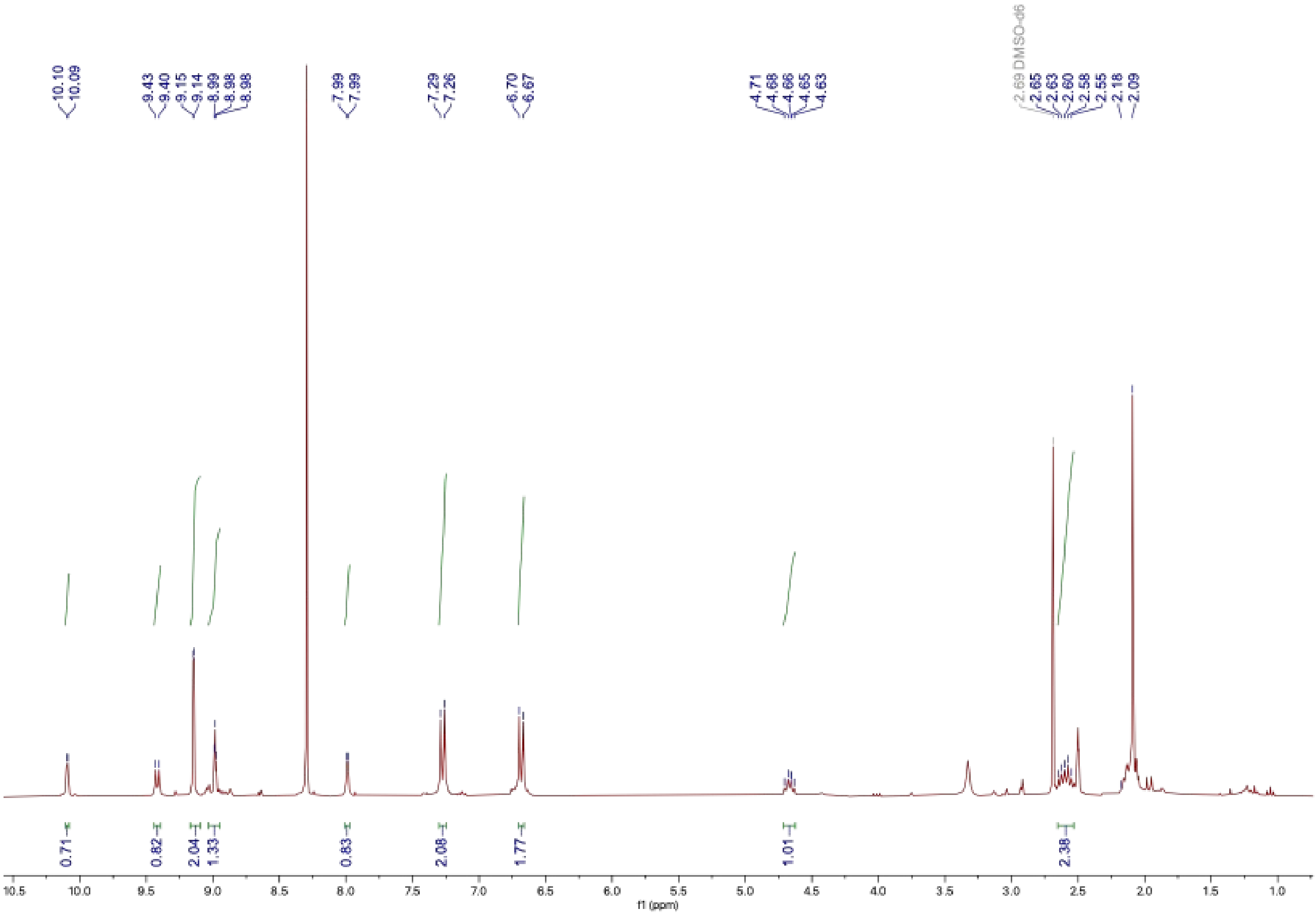

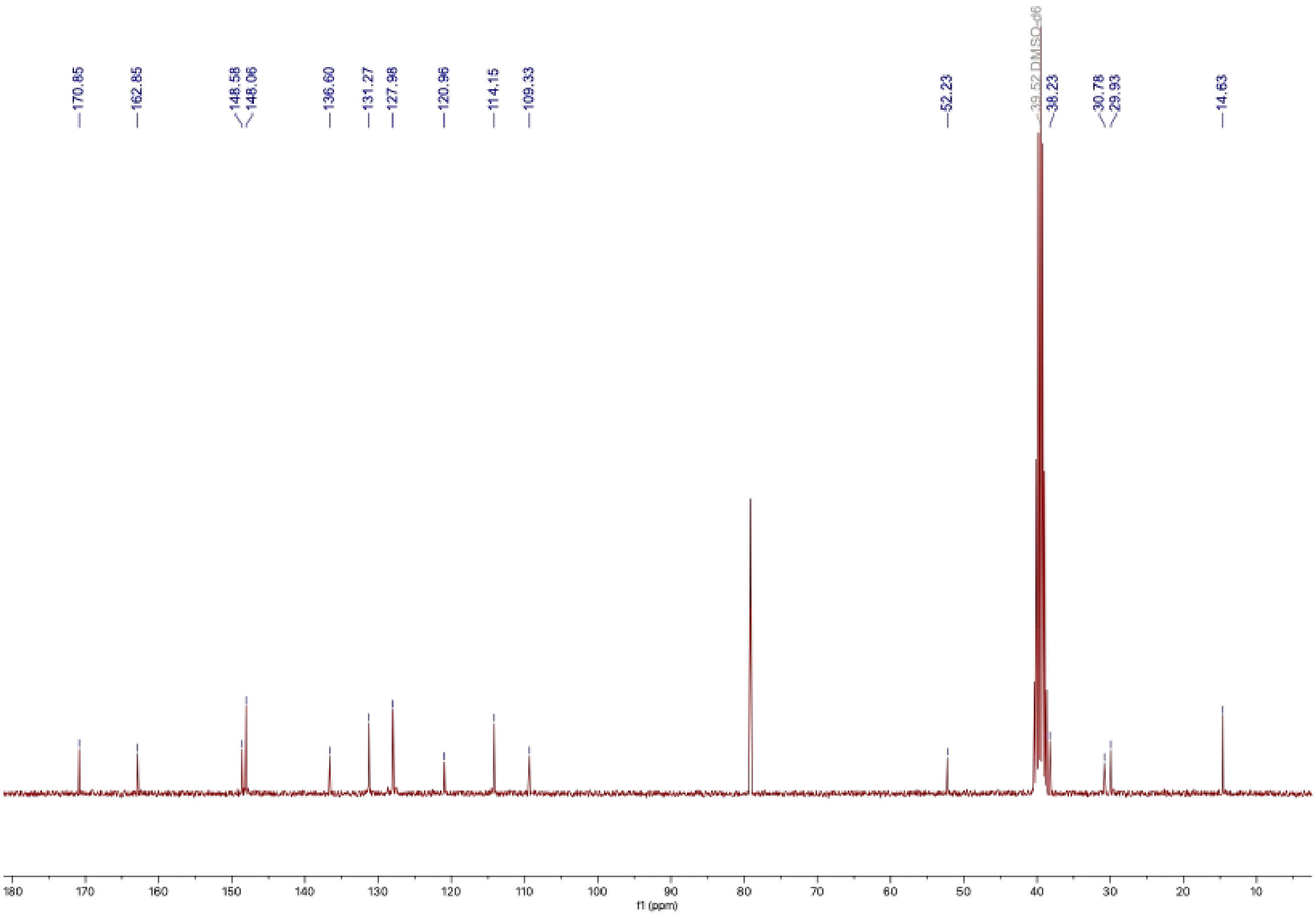

(S)-N-(1-(2-(2-bromophenyl)hydrazineyl)-1-oxo-4-phenylbutan-2-yl)-3,5-dinitrobenzamide, 47

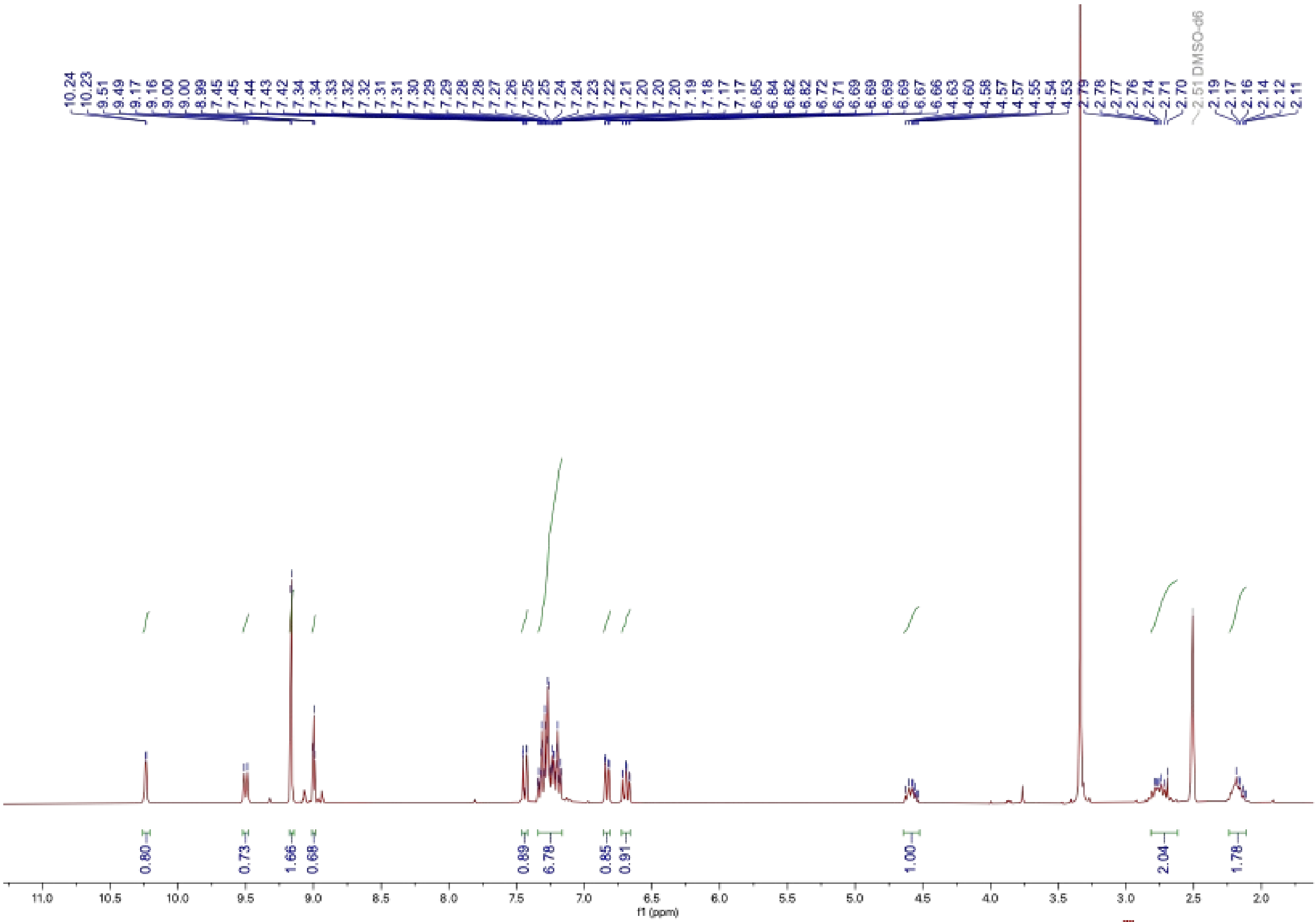

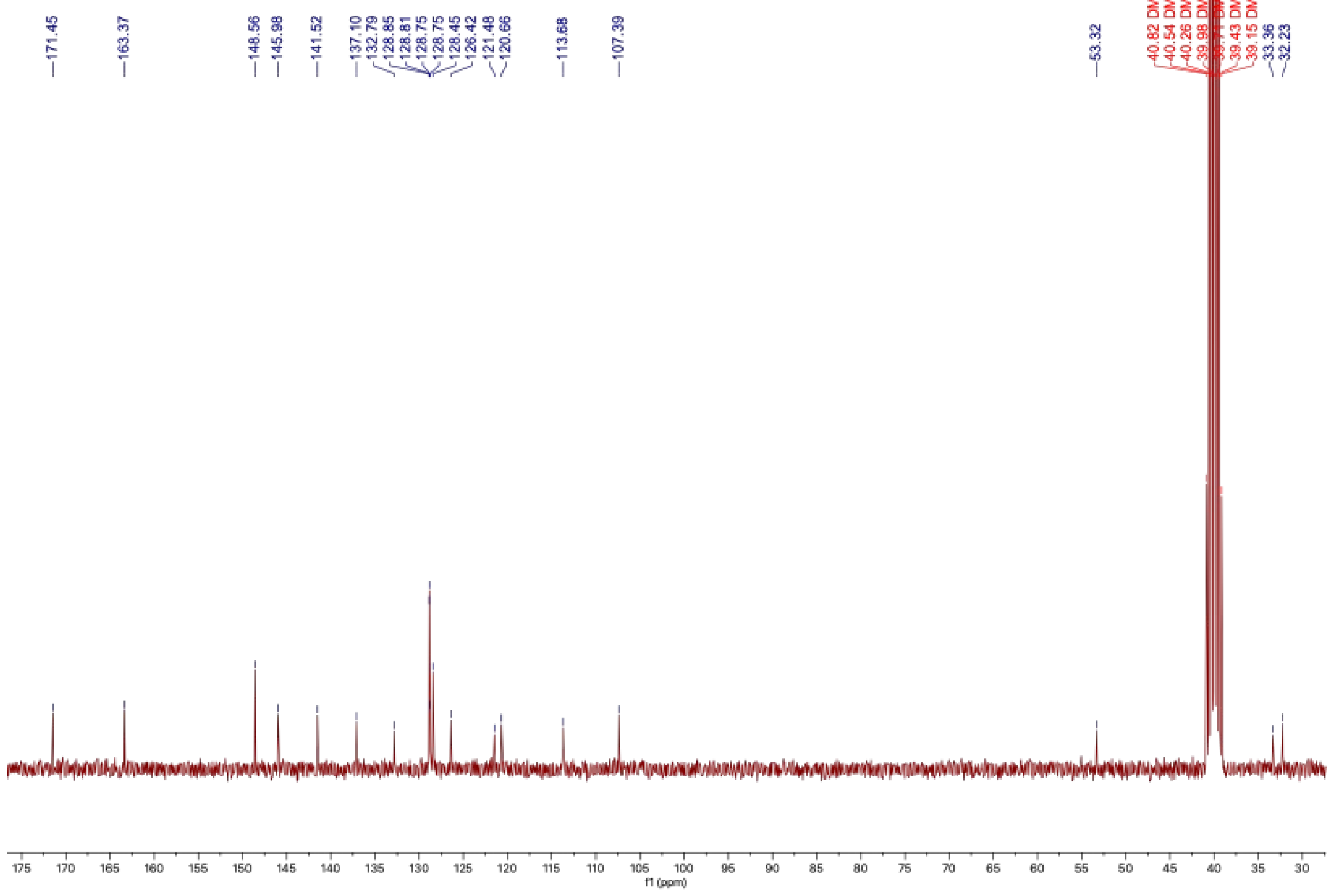

(S)-3,5-dinitro-*N*-(1-oxo-4-phenyl-1-(2-(2-(trifluoromethyl)phenyl)hydrazineyl)butan-2-yl)benzamide, 48

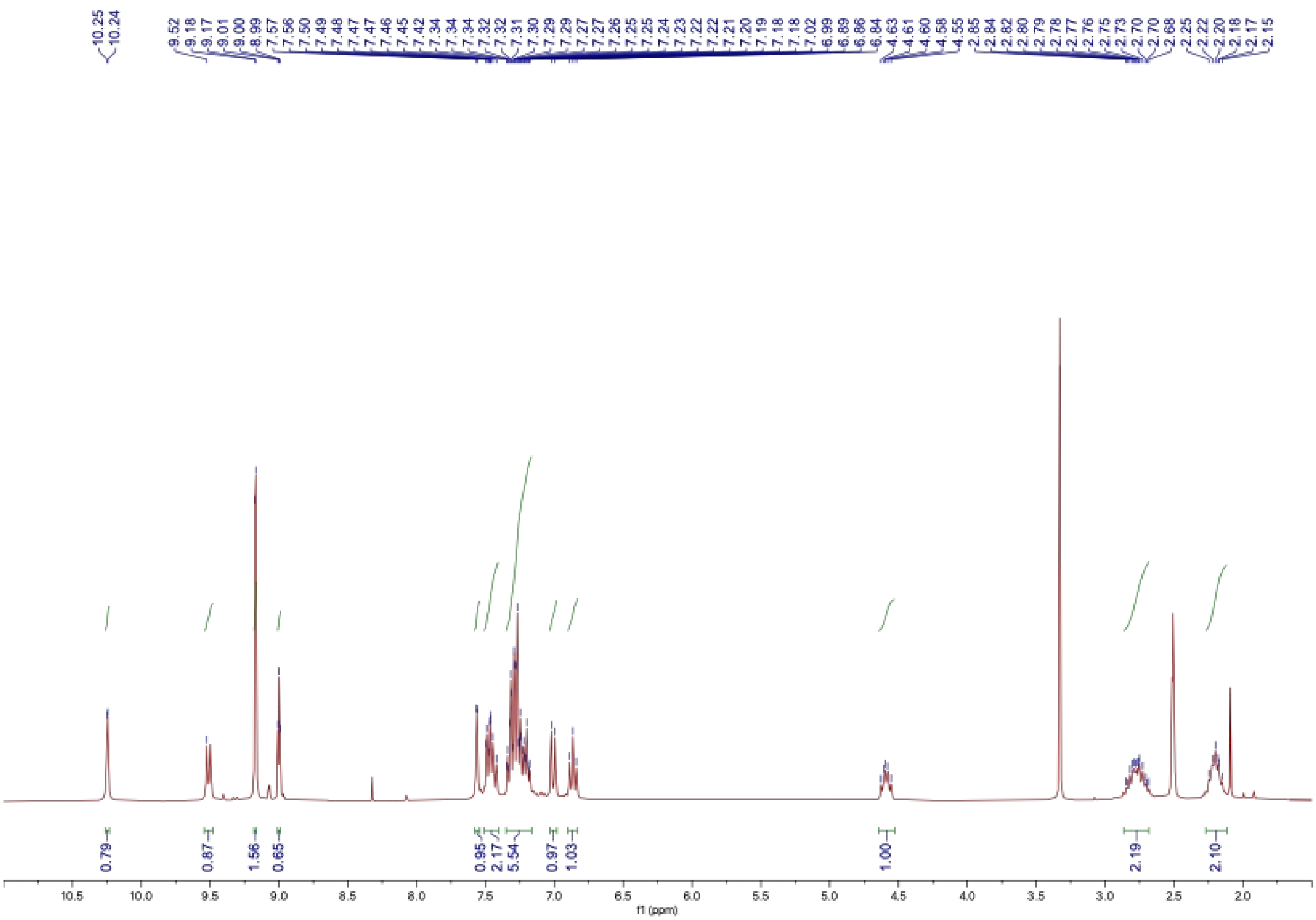

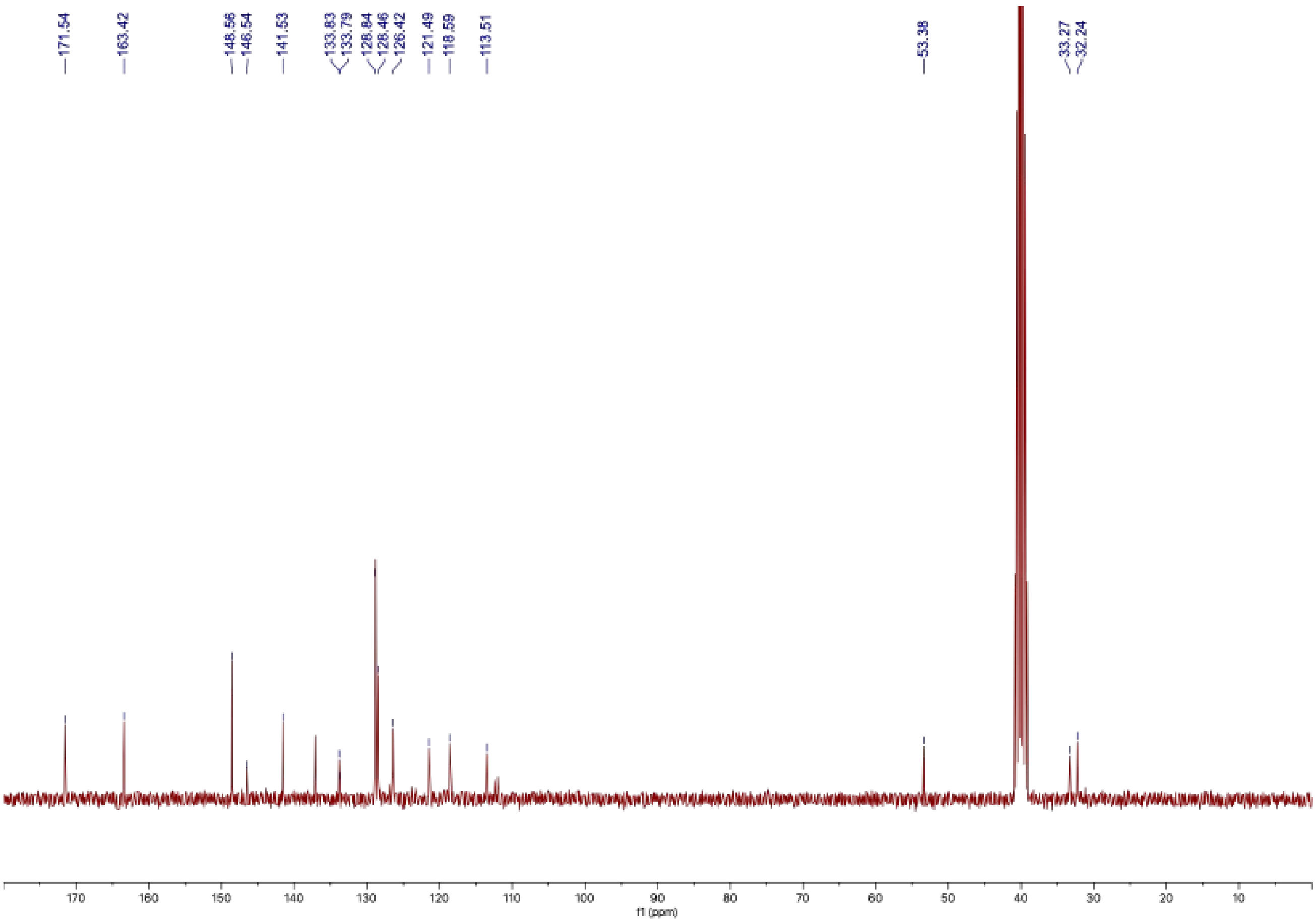

(S)-*N*-(1-(2-(3-chlorophenyl)hydrazineyl)-1-oxo-4-phenylbutan-2-yl)-3,5-dinitrobenzamide, 49

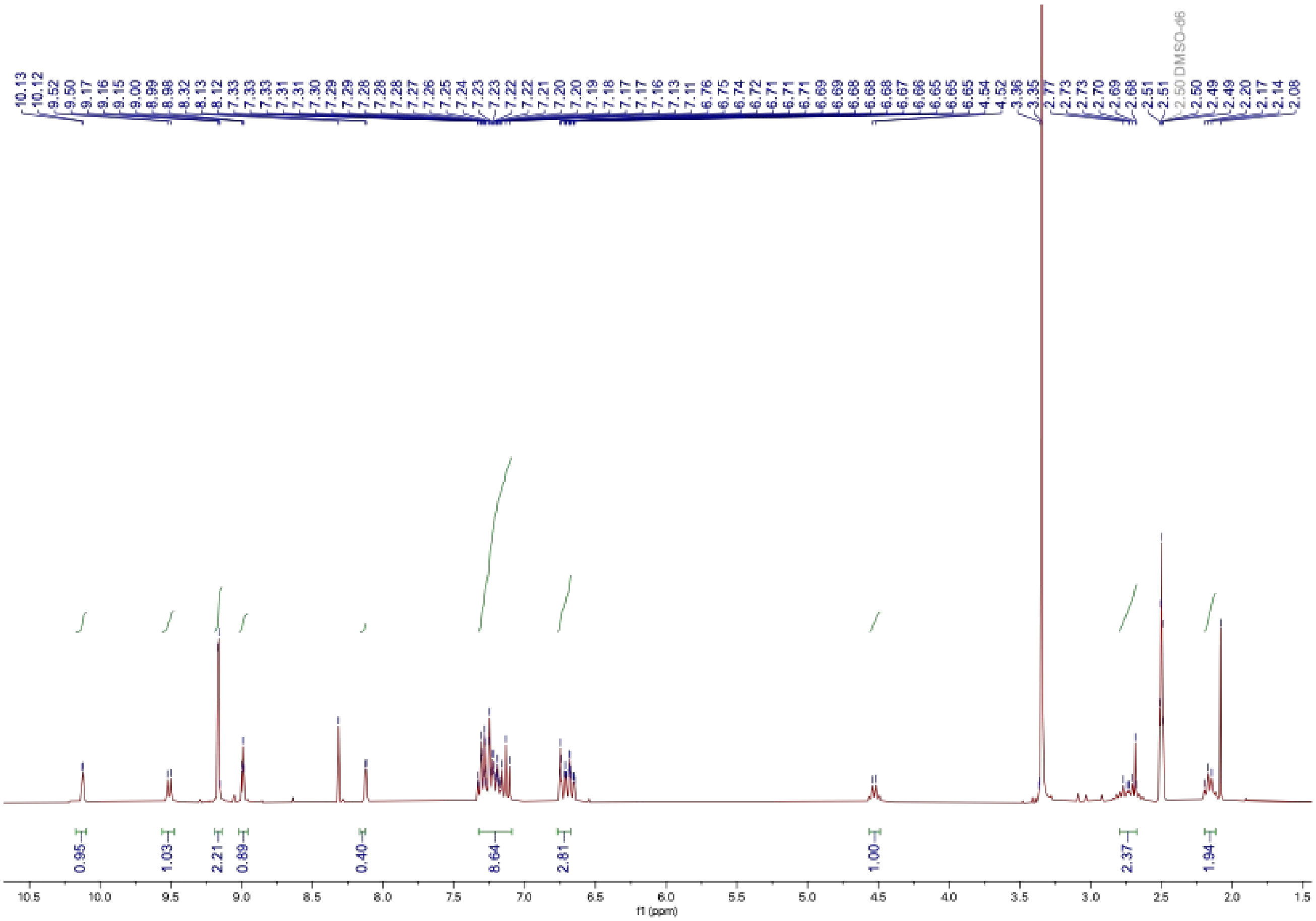

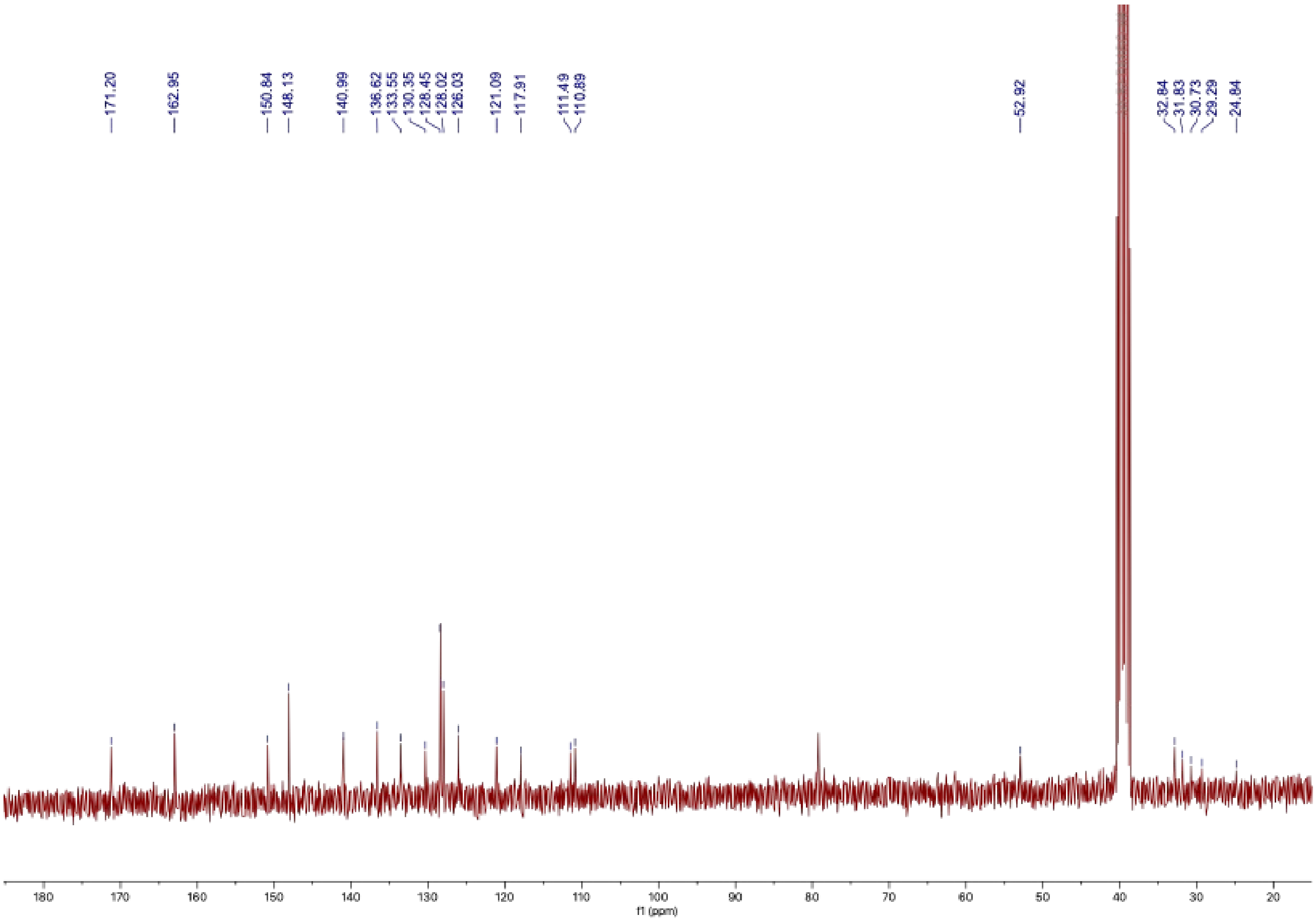

(S)-*N*-(1-(2-(3-bromophenyl)hydrazineyl)-1-oxo-4-phenylbutan-2-yl)-3,5-dinitrobenzamide, 50

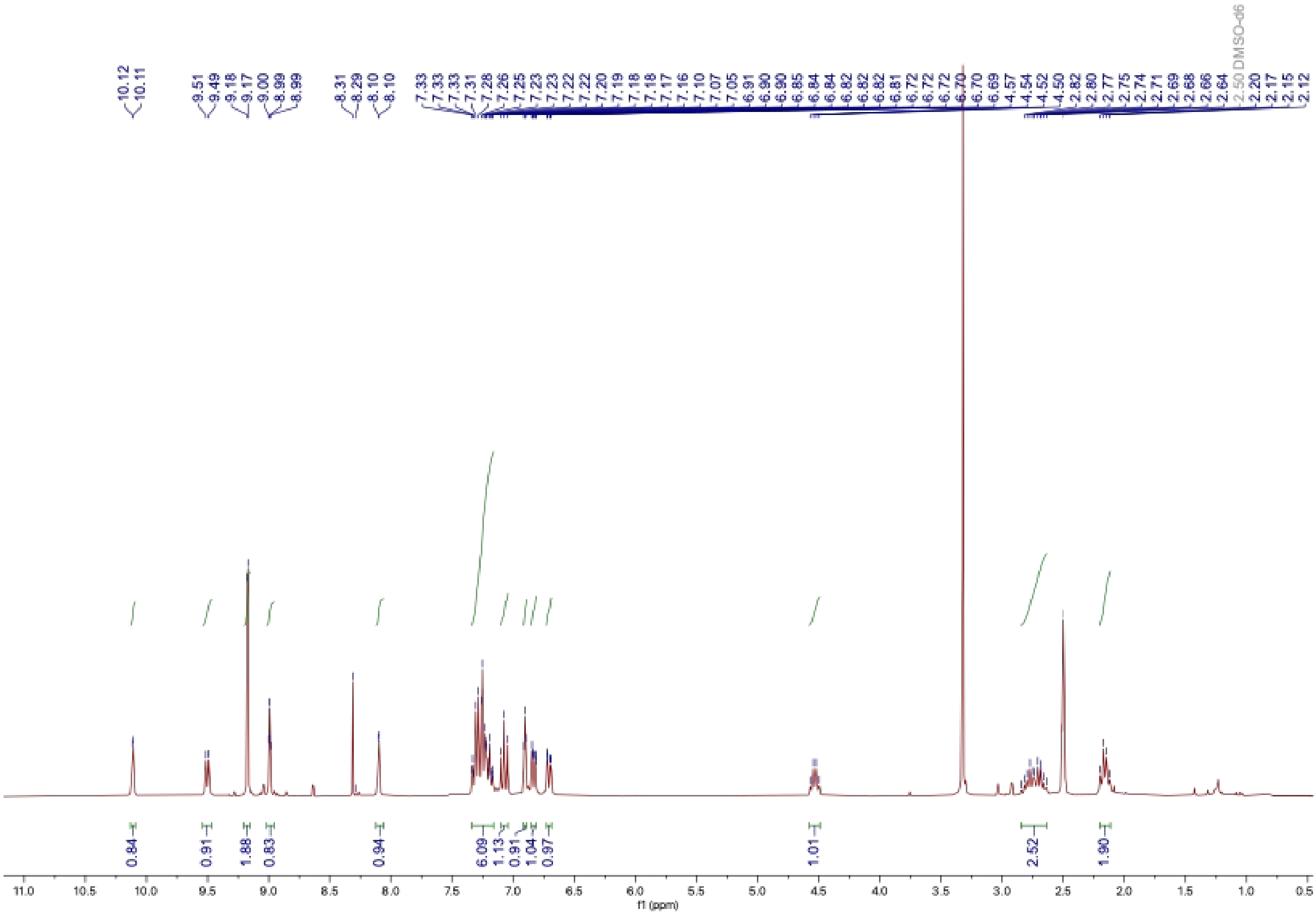

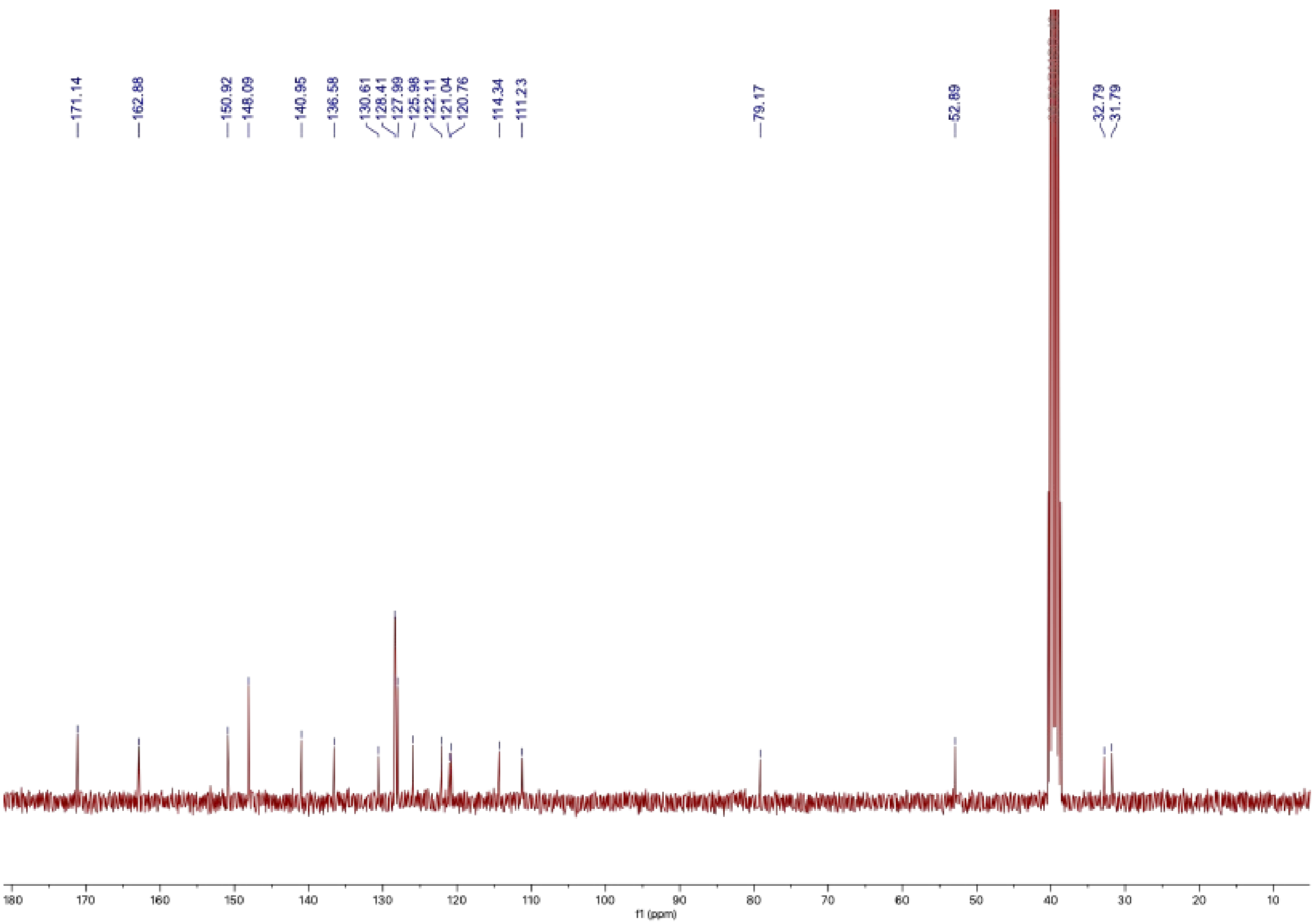

(S)-3,5-dinitro-*N*-(1-oxo-4-phenyl-1-(2-(3-(trifluoromethyl)phenyl)hydrazineyl)butan-2-yl)benzamide, 51

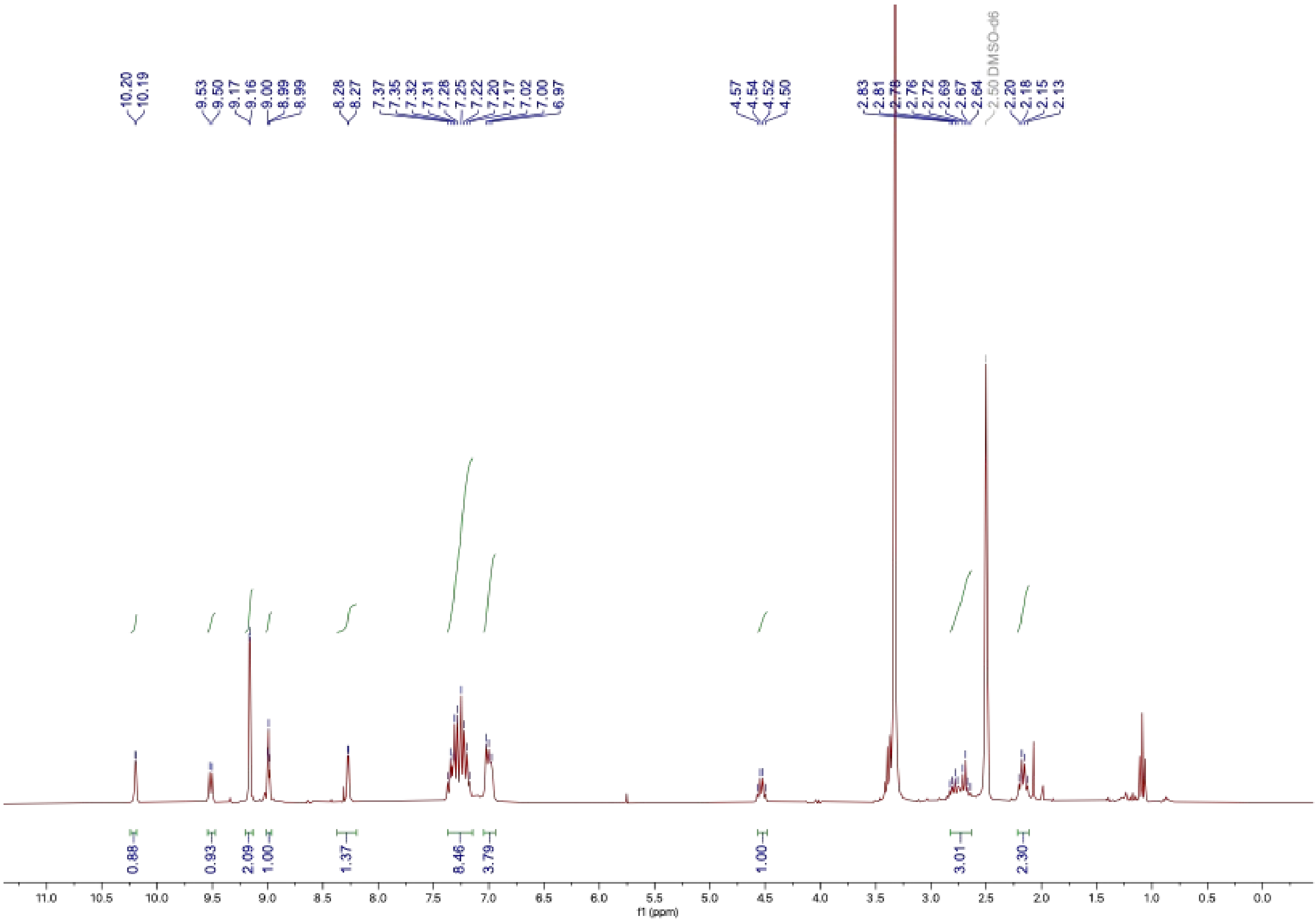

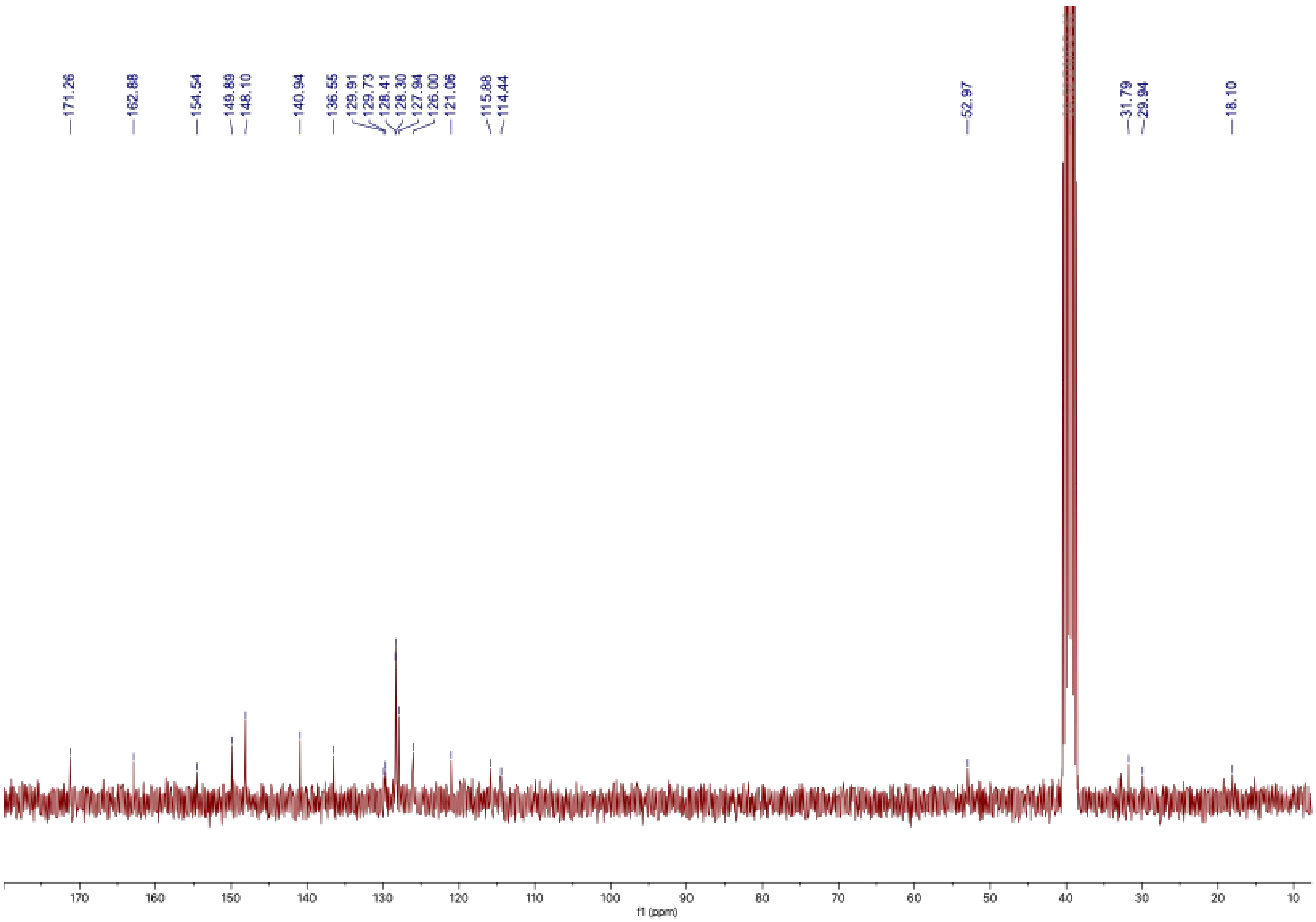

(S)-*N*-(1-(2-(4-chlorophenyl)hydrazineyl)-1-oxo-4-phenylbutan-2-yl)-3,5-dinitrobenzamide, 52

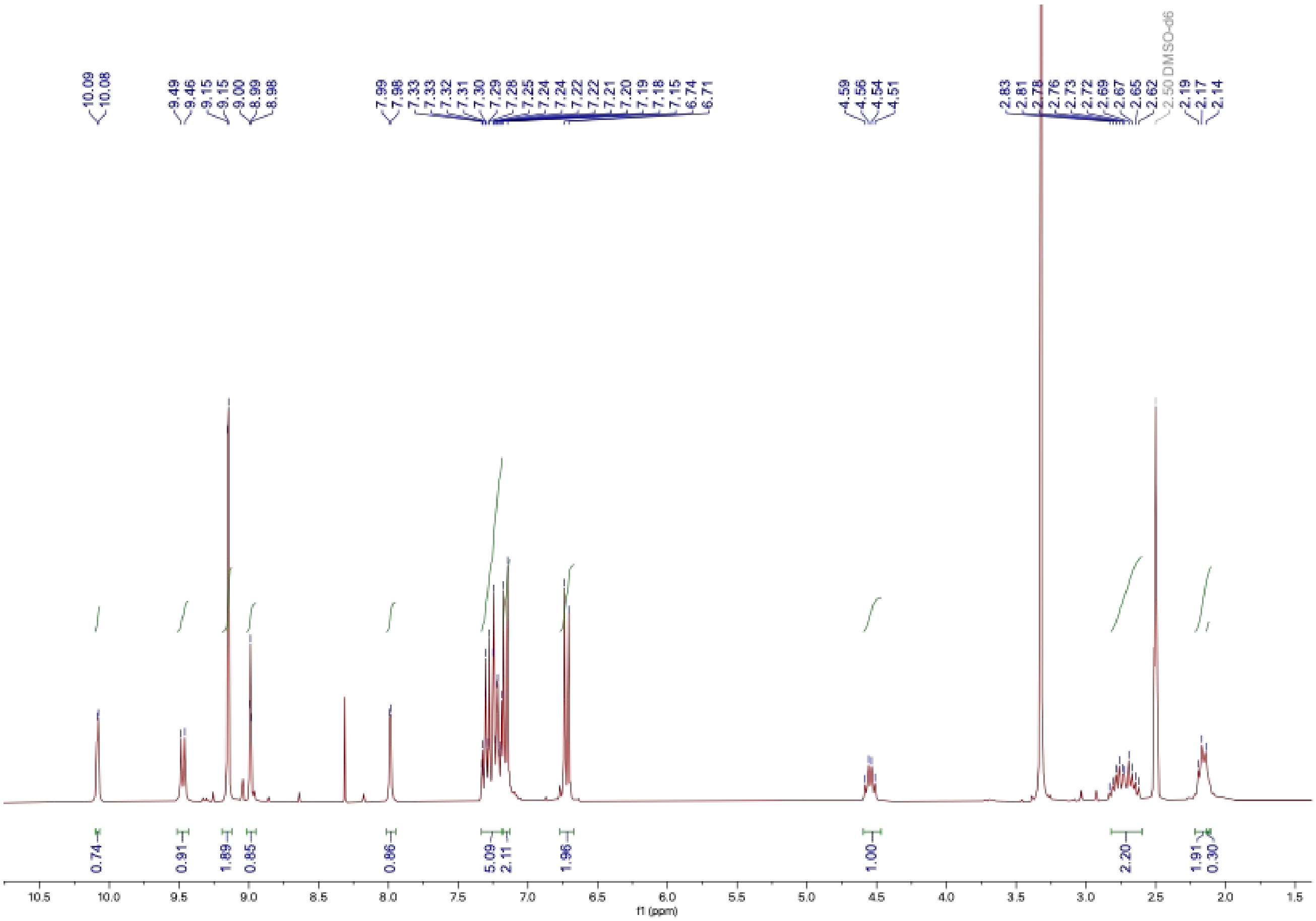

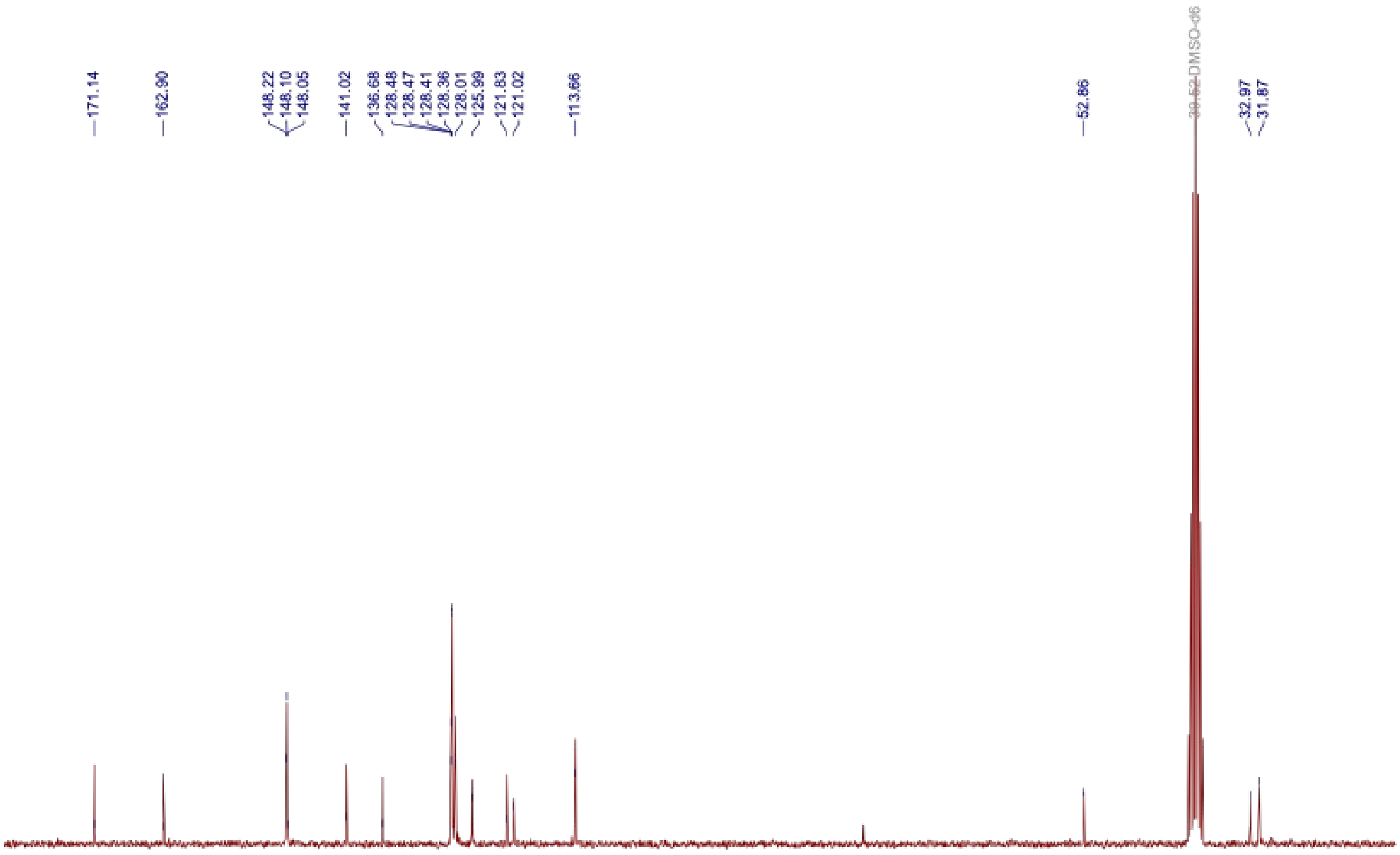

(S)-*N*-(1-(2-(4-bromophenyl)hydrazineyl)-1-oxo-4-phenylbutan-2-yl)-3,5-dinitrobenzamide, 53

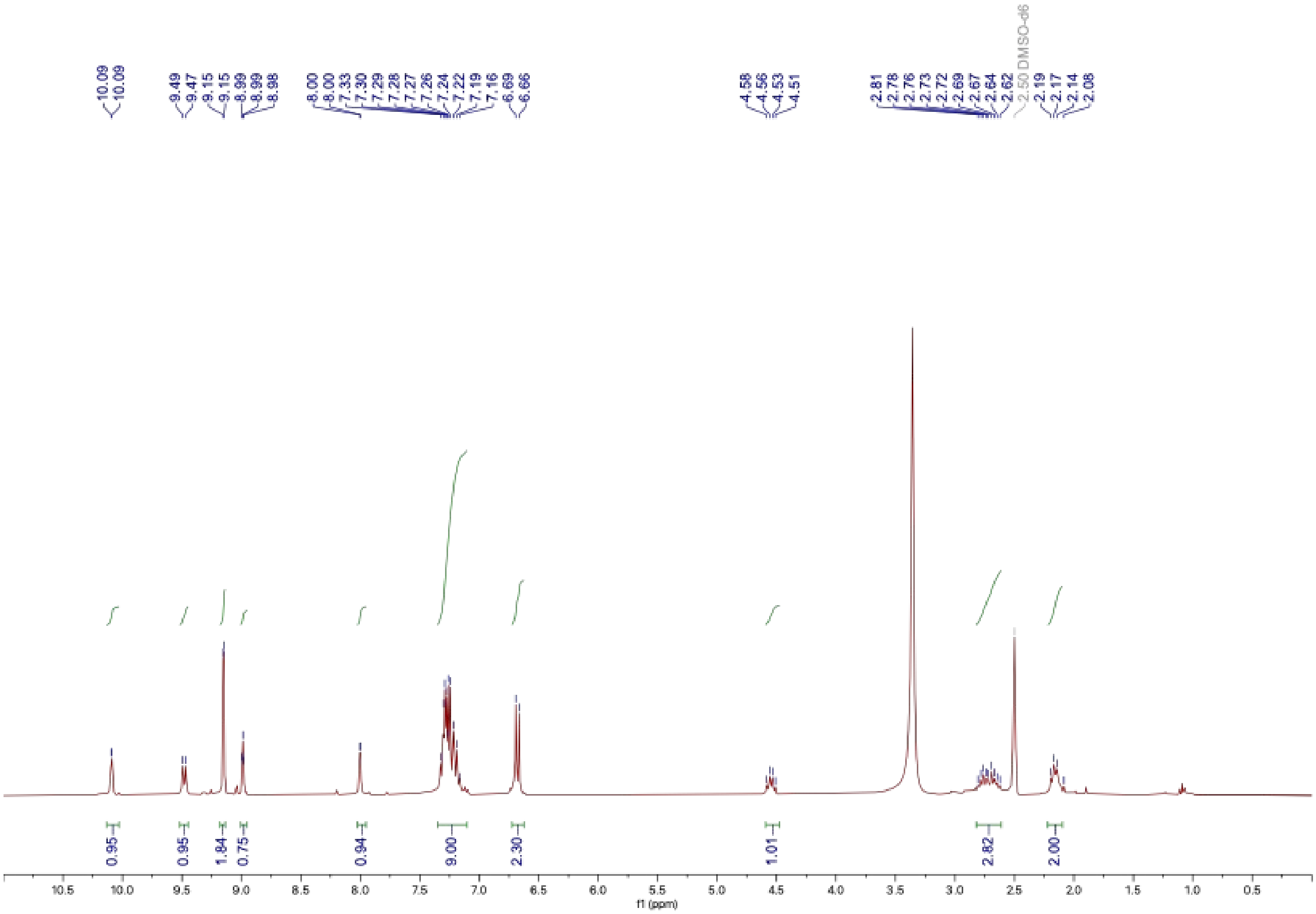

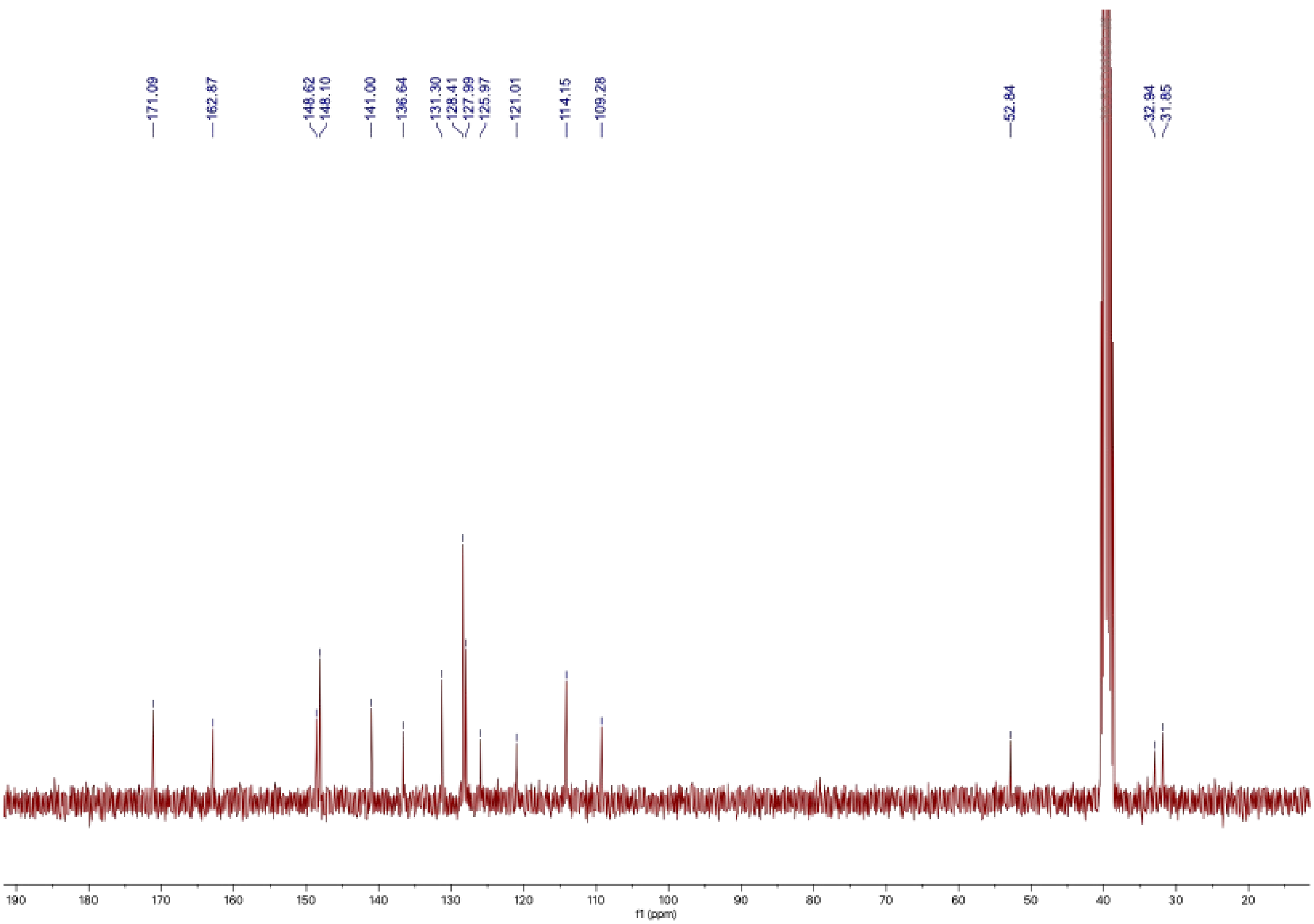

(S)-3,5-dinitro-*N*-(1-oxo-4-phenyl-1-(2-(4-(trifluoromethyl)phenyl)hydrazineyl)butan-2-yl)benzamide, 54

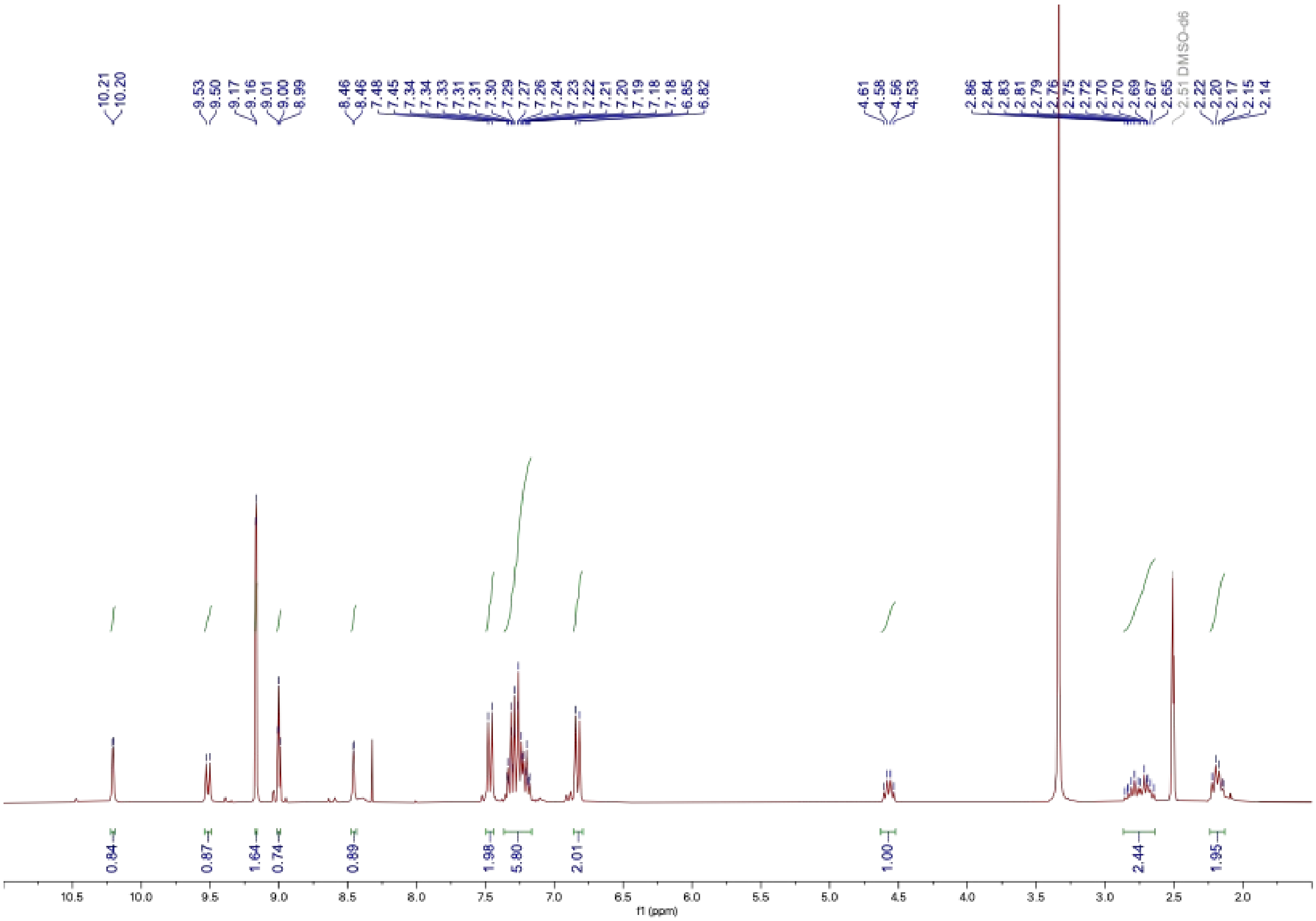

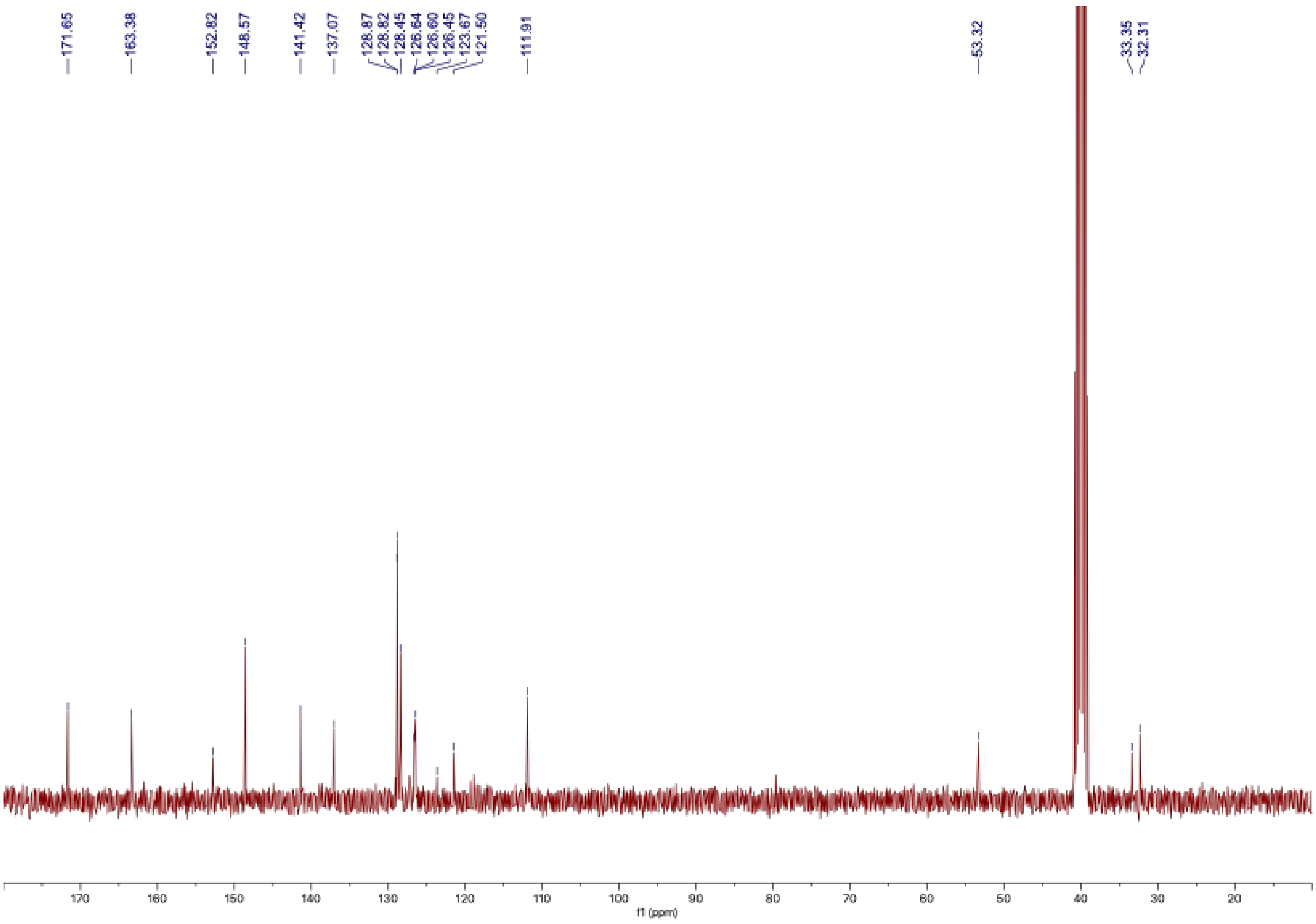

Supplement: Supplementary file 1 [file MD-017-D6MD00338A-s001.pdf]
